# Substitutional Diversity-Oriented Synthesis and In Vitro Anticancer Activity of Framework-Integrated Estradiol-Benzisoxazole Chimeras

**DOI:** 10.3390/molecules27217456

**Published:** 2022-11-02

**Authors:** Ferenc Kovács, Dóra Izabella Adamecz, Ferenc István Nagy, Benedek Papp, Mónika Kiricsi, Éva Frank

**Affiliations:** 1 Department of Organic Chemistry, University of Szeged, Dóm tér 8, H-6720 Szeged, Hungary; 2 Department of Biochemistry and Molecular Biology, Doctoral School of Biology, University of Szeged, Közép fasor 52, H-6726 Szeged, Hungary

**Keywords:** estradiol, benzisoxazole, hybrid, cancer selectivity, apoptosis induction

## Abstract

Hybridization of steroids and other pharmacophores often modifies the bioactivity of the parent compounds, improving selectivity and side effect profile. In this study, estradiol and 3′-(un)substituted benzisoxazole moieties were combined into novel molecules by structural integration of their aromatic rings. Simple estrogen starting materials, such as estrone, estradiol and estradiol-3-methylether were used for the multistep transformations. Some of the heterocyclic derivatives were prepared from the estrane precursor by a formylation or Friedel–Crafts acylation—oximation—cyclization sequence, whereas others were obtained by a functional group interconversion strategy. The antiproliferative activities of the synthesized compounds were assessed on various human cervical, breast and prostate cancer cell lines (HeLa, MCF-7, PC3, DU-145) and non-cancerous MRC-5 fibroblast cells. Based on the primary cytotoxicity screens, the most effective cancer-selective compounds were selected, their IC_50_ values were determined and their apoptosis-inducing potential was evaluated by quantitative real-time PCR. Pharmacological studies revealed a strong structure–function relationship, where derivatives with a hydroxyl group on C-17 exhibited stronger anticancer activity compared to the 17-acetylated counterparts. The present study concludes that novel estradiol-benzisoxazole hybrids exert remarkable cancer cell-specific antiproliferative activity and trigger apoptosis in cancer cells.

## 1. Introduction

**Figure 1:**
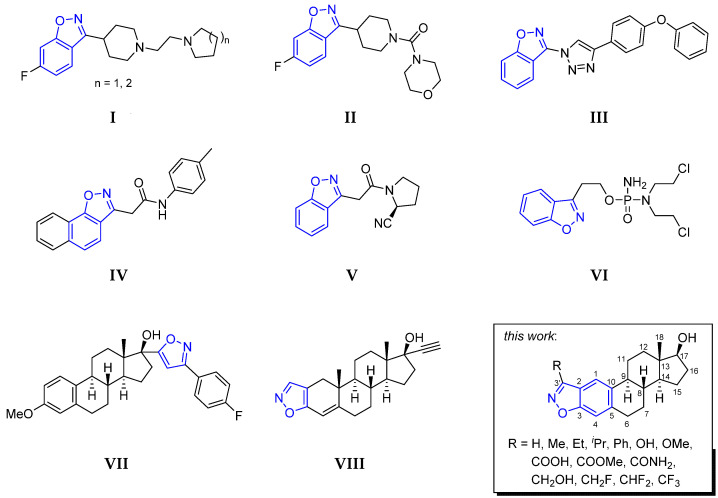
Benzisoxazoles (**I**–**VI**) [3,4,5,6,7,8] and steroidal isoxazoles (**VII**,**VIII**) [13,15] with anticancer activity and the proposed E2-benzisoxazole hybrids.

Naturally occurring and synthetic benzisoxazoles [1], particularly their 3-substituted representatives [2], are important pharmacophores and serve as valuable tools for drug design and discovery, having a high number of positive hits in biological screens. Because of their versatile properties and potential as selective ligands for a variety of macromolecular targets, these bicyclic aromatic ring systems constitute the essential structural motif in a wide range of pharmacologically active compounds, including a number of potential anticancer agents (Figure 1) [3,4,5,6,7,8,9]. Furthermore, the benzisoxazole scaffold is often used as a bioisosteric replacement for the benzoyl group of biologically active molecules [10].

Chemical modification of natural steroids with different heterocycles provides a way to alter the function of the parent compound, and several derivatives have been demonstrated to be effective in the prevention and treatment of many types of cancers [11]. Although there are no examples in the literature for the synthesis of steroidal benzisoxazoles, the incorporation of the five-membered isoxazole ring into a sterane backbone in either a connected [12,13,14] or a condensed manner [15] led to some effective antiproliferative agents (**I**–**VIII**, Figure 1). Nevertheless, the phenolic A-ring of estrogens offers the possibility to synthesize aromatic ring-integrated benzisoxazole hybrids and this modification may have beneficial outcomes in several aspects. First, molecular hybridization of steroids with other potentially active compounds often modulates bioactivity and improves the selectivity and side effect profile of the individual compounds [16,17]. In addition, derivatization of estrogens at the C2–C3 position by the introduction of a fused heteroring while simultaneously eliminating the phenolic OH group makes it likely that the novel derivative will not be able to bind to the estrogen receptor, and will therefore be free of undesired hormonal effects [18]. Even estradiol (E2) derivatives substituted at C-2 with different functional groups, such as 2-methoxyestradiol (2-ME2) and its structural analogues [19], in which the phenolic OH group required for estrogen receptor binding is intact, do not have hormonal effects due to steric and electronic factors induced by the C-2 group [20]. Modification of the OH group, which plays a key role as an H-donor in hormone receptor binding [20], together with the C-2 functionalization through a heteroring formation, can clearly reduce the interaction with the target protein. At the same time, the 3-OH group of 2-ME2 analogs can be held responsible for their rapid metabolic degradation [21], so the incorporation of the oxygen into a heteroring can increase pharmacokinetic stability as well. Although the sterane backbone can be favorable promoting cell membrane penetration and thus the delivery to the site of action, the heteroatoms of the incorporated isoxazole moiety may be involved in H-bonding interactions to the target macromolecule other than a hormone receptor. However, it is important to note that sterane-based structures may have a very complex mode of action in the human body [22], e.g., at least five different aspects of the anticancer mechanism of action of 2-ME2 have been elucidated so far [21].

As a continuation of our ongoing research for designing steroidal A-ring integrated chimeras with anticancer activity [18,23,24,25,26], a benzisoxazole scaffold containing different substituents at the C-3′ position of the heteroring was hybridized with the aromatic ring of E2. According to a comprehensive analysis of the chemical structure of marketed anticancer agents, the most abundant functional groups of these drugs are OH, COOH, COOR, NH_2_ and F; moreover, 43.4% of them contain both aromatic and non-aromatic rings as part of their structure [27]. These structural features were taken into account in designing the synthesis of novel compounds that show diversity in the substitution pattern of the *N*,*O*-heterocyclic ring. Virtual screening of the pharmacokinetic parameters using ChemAxon’s Chemicalize software [28] showed that almost all the molecules designed to be produced meet the drug-like criteria defined by Lipinski and Veber [29,30] (Supplementary Material p50).

Various protocols for the synthesis of 3-substituted benzisoxazoles have been reported so far [31]. Among others, catalytic cyclizations of 2-hydroxyaryl aldoximes and ketoximes [32] leading to the N–O bond formation through intramolecular Mitsunobu reaction [33] or by conversion of the hydroxyl group of the oximes to good, leaving groups followed by base-catalyzed ring-closure, are well-known procedures [3,34]. However, due to the necessity of a strong base or high temperature, these methods often involve the formation of other products; e.g., Beckmann rearrangement of the oxime and subsequent cyclization can lead to isomeric benzoxazoles. A new route using 2,3-dichloro-5,6-dicyano-1,4-benzoquinone (DDQ)/PPh_3_ at room temperature (RT) was reported to overcome these disadvantages [34]. Since the phenolic substructure is present in estrogens, the 2-hydroxyaryl aldehyde or ketone precursors of oximes can be obtained by regioselective formylation or Friedel–Crafts acylation. Although some drawbacks of these approaches could be found, such as the need for 3–4 reaction steps from E2 to benzisoxazoles, we tried to optimize each step in order to achieve high conversions without the formation of undesirable byproducts.

The newly synthesized benzisoxazole derivatives were evaluated for their in vitro antiproliferative activities on DU-145 and PC3 (both prostate cancer), HeLa (cervical cancer) and MCF-7 (breast cancer) cell lines. For comparison, the cytotoxicity of the compounds was tested on MRC-5 non-cancerous lung fibroblast cells. According to the results of the initial screening, the most potent anticancer agents were selected, their IC_50_ values were determined and their apoptosis-inducing potential was examined by reverse transcription quantitative polymerase chain reaction (RT-qPCR) measurements.

## 2. Results and Discussion

### 2.1. Syntheses

Based on the literature background, the synthesis of estradiol-A-ring-integrated unsubstituted benzoisoxazole and those containing diverse functional groups at C-3′ position of the heteroring were designed (Figure 1). For the multistep transformations estrone (E1), estradiol (E2) and estradiol-3-methylether (E2Me) were used as starting materials. Some of the reactions (if R = H, Me, Et, *^i^*Pr, Ph, COOMe, CF_3_, OH, OMe) were initiated by regioselective formylation or Friedel–Crafts acylation at the C-2 position of the corresponding estrane precursor (**1** or E2). The following oxime formation and activation of the oxime-OH offered the possibility of cyclization with the phenolic OH group to isoxazole under appropriate conditions. In other cases (if R = COOH, CONH_2_, CH_2_OH, CH_2_F, CHF_2_), a common 3′-methylcarboxylated benzisoxazole intermediate served as a precursor for additional functional group interconversion (FGI).

**Scheme 1:**
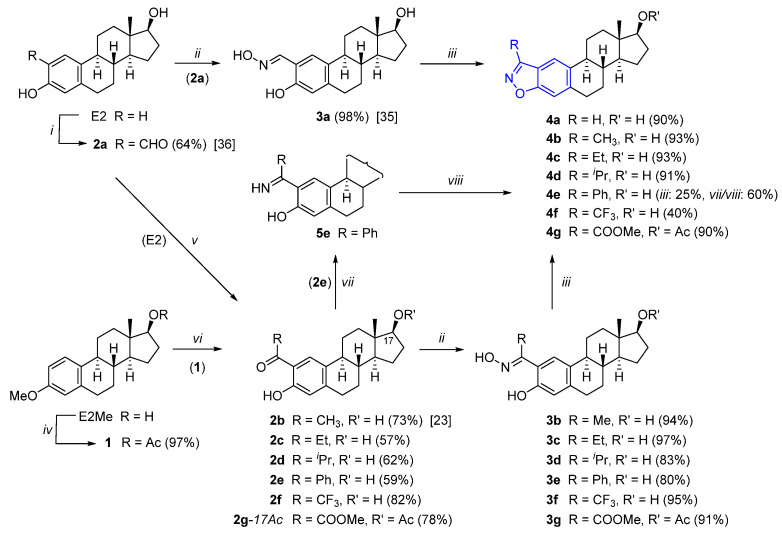
Synthesis of A-ring-condensed isoxazoles from E2 and E2Me. *Reagents and conditions*: (i) EtMgBr, THF, (CH_2_O)_n_, hexamethylphosphoramide (HMPA), benzene, 80 °C, 20 h; KOH, MeOH, RT, 1 h; (ii) NH_2_OH·HCl, NaOAc or pyridine, EtOH, RT (for **3a** and **3g**) or reflux (for **3b**–**f**), 2–24 h; (iii) DDQ, PPh_3_, DCM, RT, 10 min (for **4a**, **4f** and **4g**), with the addition of Et_3_N (for **4b**–**e**); (iv) Ac_2_O, cat. H_2_SO_4_, sonication, RT, 5 min; (v) TFAA (for **2f**), AlCl_3_, DCM, 0 °C, 4 h; (vi) AcCl (for **2b**), EtC(O)Cl (for **2c**), *^i^*PrC(O)Cl (for **2d**), BzCl (for **2e**), Cl(CO)_2_OMe (for **2g**), AlCl_3_, DCM, 0 °C to RT, 4 h, followed by alkaline deacetylation with NaOH in MeOH/DCM (1:9) (for **2b**–**f**); (vii) NH_3_/MeOH (6M), RT, 2 h; (viii) NCS, K_2_CO_3_, THF, RT, overnight.

After a nearly quantitative oximation [35] of 2-formyl-E2 (**2a**), obtained by regioselective Cashiragi *ortho*-formylation of E2 [36], DDQ/PPh_3_-induced ring-closure of the 2-hydroxyaryl aldoxime moiety in **3a** [34] proceeded rapidly to result in an unsubstituted A-ring-condensed isoxazole derivative (**4a**) in high yield (Scheme 1). During the 3-step sequence, only formylation reduced the efficiency, leading to a ca. 60% yield of 2-formyl-E2 (**2a**). The residual E2 and the minor 4-formyl isomer with similar polarity to that of **2a** were removed by column chromatography and subsequent recrystallization. For the similar synthesis of the 3′-methyl- (**4b**), 3′-ethyl (**4c**), 3′-isopropyl (**4d**) and 3′-phenyl-substituted steroidal benzisoxazoles (**4e**), estradiol-3-methylether-17-acetate (**1**) was used as starting material in order to protect the reactive OH groups of E2 during Friedel–Crafts acylation reactions. Regioselective AlCl_3_-induced electrophilic substitution on C-2 with acetyl chloride (AcCl) [23], propionyl chloride, isobutyryl chloride or benzoyl chloride (BzCl) with simultaneous deprotection of 3-OH by the excess Lewis acid, and the following alkaline deprotection of 17-OH led to acylated E2 derivatives **2b**–**e** in good yields. Compound **2g** obtained by the similar conversion of **1** with methyl chlorooxoacetate was not subjected to alkaline treatment due to its diester character but was used for the next reaction step. Analogous CF_3_-substituted isoxazole **2f** could not be synthesized from **1**, since only trifluoroacetic anhydride (TFAA) instead of the more reactive acyl chloride was available as a reagent for the electrophile generation and in this case, the 3-OMe group of **1** did not activate the *ortho* position of the aromatic ring enough for an electrophilic attack. Thus, Friedel–Crafts acylation from E2 was performed under controlled conditions. Low temperature and 6 equiv. of AlCl_3_ were required for high yield of the desired product (**2f**). Although the formation of an unidentified apolar by-product (probably as a result of Wagner-Meerwein rearrangement) was observed with increasing temperature, only *O*-acylation of E2 occurred by using less (2 equiv.) Lewis acid. The structures of the 2-substituted derivatives were determined by ^1^H and ^13^C NMR spectroscopy (Supplementary Material, p2–49). The regioselective entry of the formyl or acyl substituents into C-2 position was supported by the two singlet signals for C-1 and C-4 in the ^1^H NMR spectra of **2a**–**g**. In all cases, the carbonyl-C of the formyl or acyl group as well as the other carbon peaks, if relevant, of the introduced moiety were also observed in the ^13^C NMR (J-mod) spectra. 

Next, condensation reactions of ketones **2b**–**g** were carried out with hydroxylamine to afford the corresponding oximes as a single isomer (**3b**, **3c** and **3g**) [37] or mixtures of *E* and *Z* forms (**3d**–**f**) [38] (Scheme 1). Subsequent DDQ/PPh_3_-initiated cyclization of the oximes in dichloromethane (DCM) under mild conditions afforded the desired E2-benzisoxazole hybrids (**4b**–**g**). It is worth mentioning that lower yields (25% and 40%) were obtained for **4e** and **4f**. Because of stereoelectronic reasons, only one of the oxime isomers was able to cyclize to isoxazole with the bulky adduct formed from DDQ and PPh_3_ [34], whereas the other remained unchanged during the reaction, as confirmed by thin-layer chromatography (TLC) monitoring. Additionally, the presence of the Ph group in **3e** activated the compound towards Beckmann rearrangement, leading to the formation of 2-phenylbenzoxazole as the main product. In order to enhance the yield, an alternative route was carried out for the transformation of **2e**, involving an imine formation (**5e**) with ammonia and the following chlorination/dehydrohalocyclization by *N*-chlorosuccinimide (NCS)/K_2_CO_3_ [39]. In this case, steric factors do not impede the cyclization to **4e**, and a higher yield (60%) was obtained (Scheme 1). Unfortunately, this latter method did not work for the conversion of the trifluoromethyl derivative **2f**. 

**Scheme 2:**
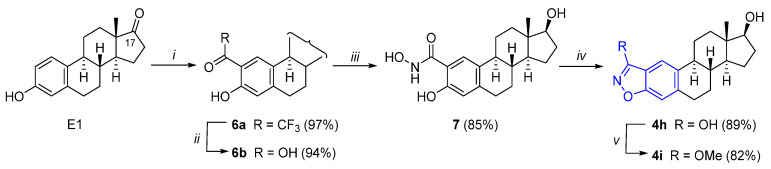
Synthesis of A-ring-condensed isoxazoles from E1. *Reagents and conditions*: (i) TFAA, AlCl_3_, DCM, 0 °C to RT, 4.5 h, (ii) KOH, EtOH/H_2_O, reflux, 4 h; (iii) three steps: NaBH_4_/EtOH, RT, 30 min (**6c**, R = OH, 17β-OH at C-17); MeI, Na_2_CO_3_, DMF, 50 °C, 2 h (**6d**, R = OMe, 17β-OH at C-17); NH_2_OH (50 wt. % aq. solution), KOH, MeOH/dioxane, RT, 30 min; (iv) diisopropyl azodicarboxylate (DIAD), PPh_3_, THF, RT, 2 h; (v) MeI, K_2_CO_3_, DMF, RT, 1 h.

For the synthesis of two additional heterocyclic derivatives (**4h** and **4i**), E1 was used as a starting material (Scheme 2). After regioselective Friedel–Crafts acylation with TFAA and a subsequent haloform reaction of **6a** [40], an E2-salicylic acid domain-integrated hybrid (**6b**) was produced. This compound was next converted to a hydroxamic acid derivative of E2 (**7**) by a three-step sequence involving C-17 ketone reduction, methylester formation of the acid moiety [41] and the final nucleophilic acyl substitution with NH_2_OH [42]. Mitsunobu-triggered heterocyclization in anhydrous THF at RT [43] afforded 3′-hydroxybenzisoxazole **4h** in 89% yield, whereas its *O*-methylation furnished **4i** in 82% yield (Scheme 2). 

**Scheme 3:**
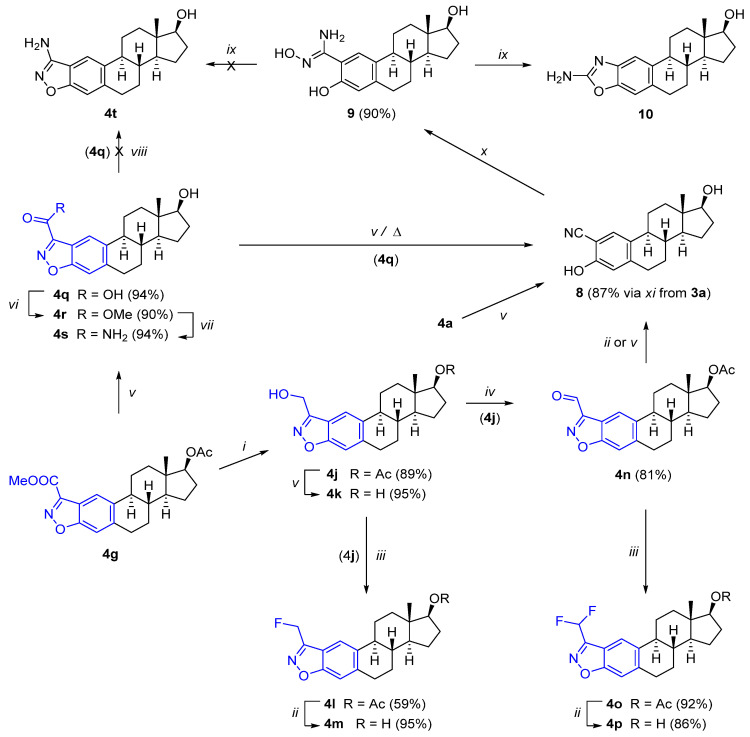
Synthesis of A-ring-condensed 3′-substituted isoxazoles by FGI. *Reagents and conditions*: (i) NaBH_4_, MeOH, RT, 1 h; (ii) LiOH, THF/H_2_O, RT, 2–24 h; (iii) DAST, DCM, RT, 1–2 h; (iv) DMP, DCM, RT, 2 h; (v) NaOH, MeOH/DCM, RT, 1–20 h; (vi) MeOH, H_2_SO_4_, reflux, 36 h; (vii) NH_3_/MeOH (6M), RT, 4 h. (viii) DPPA, toluene, reflux, overnight; (ix) DDQ, PPh_3_, with or without the addition of Et_3_N, DCM, RT, 1 h; (x) NH_2_OH (50 wt. % aq. solution), Na_2_CO_3_, EtOH, reflux, overnight; (xi) DIAD, PPh_3_, ACN, 1 h.

In the following, additional benzisoxazole derivatives were synthesized by FGI of the pre-formed substituted heteroring (Scheme 3). Reduction of the previously obtained 17-*O*-protected 3′-methyl ester (**4g**) with NaBH_4_ in MeOH gave **4j**. Deacetylation of 17-OH led to **4k**, whereas conversion of **4j** with diethylaminosulfur trifluoride (DAST) in DCM and subsequent deprotection afforded the fluoromethylated product **4m** in high yield. On the other hand, **4j** was mildly oxidized with Dess-Martin periodinane (DMP) to **4n**, which was then transformed to **4p** by nucleophilic fluorination followed by deacetylation of **4o**. Interestingly, treatment of **4n** with LiOH or NaOH initiated not only deacetylation but also formic acid elimination accompanied by heteroring opening to give 2-cyano-E2 (**8**). The same product (**8**) was also obtained from **4a** by Kemp elimination [33] or from **4q** by decarboxylation under the influence of heat and/or basic medium. Otherwise, the latter product (**4q**) was prepared by alkaline hydrolysis of the two ester functionalities in **4g** at RT. Starting from **4q**, two additional derivatives (**4r** and **4s**) were also synthesized by FGI. The preparation of the 3′-amino-benzisoxazole-E2 hybrid (**4t**) proved to be the most difficult challenge. Since the DDQ/PPh_3_-induced cyclization of amidoxime **9** obtained from **8** failed and 2-amino-oxazole **10** was formed instead of the desired isoxazole **4t** by Tiemann rearrangement, the Curtius rearrangement of the acyl azide available from **4q** with diphenylphosphorylazide (DPPA) in refluxing toluene was tried to carry out. Nevertheless, unfortunately, this reaction did not lead to success either. The reaction did not proceed even to the formation of the azide, so the preparation of the amino-substituted derivative (**4t**) was discarded.

Structural determination of the novel steroidal A-ring-fused isoxazoles (**4a**–**s**) was accomplished using ^1^H NMR, ^13^C NMR (J-mod) and MS measurements. The fact of the cyclization was confirmed by the disappearance of the proton signal of the phenolic OH group in the proton spectra, and in the case of the 3′-substituted derivatives (**4b**–**s**) by the negative peak of the three hydrogen-free, sp^2^-hybridized carbon atoms (C-2, C-3, and C-3′). For the unsubstituted isoxazole **4a**, the peak of 3′-H was detected at 8.60 ppm, whereas C-3′ was observed as a positive signal at 146.2 ppm. The measured molecular masses were in good agreement with those calculated from the structures.

### 2.2. Pharmacological Studies

**Figure 2:**
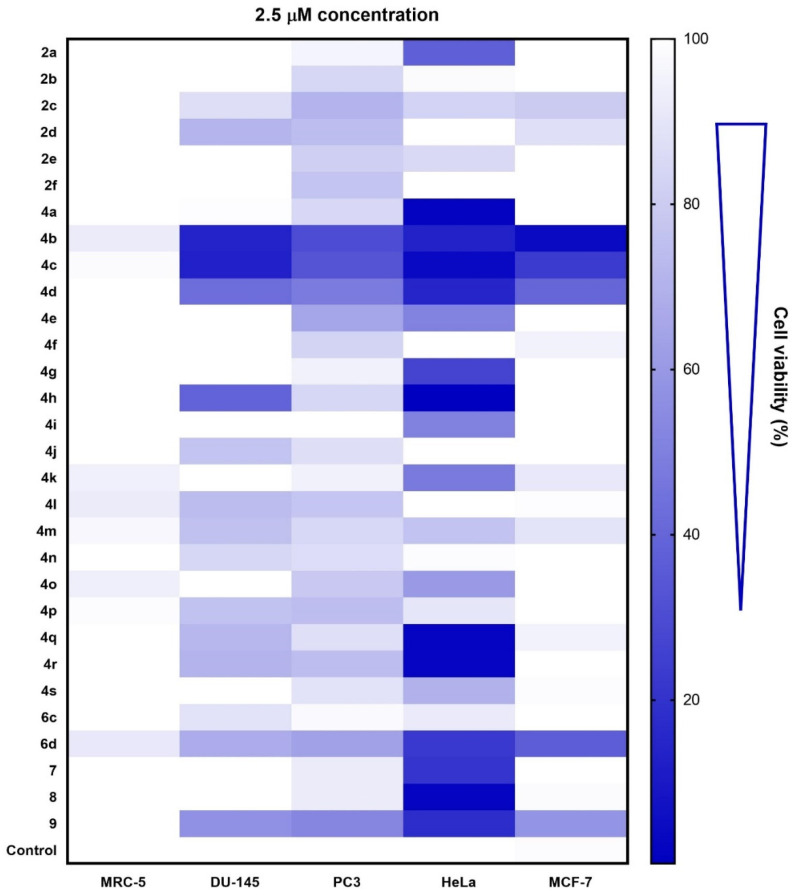
Representative heatmap of the primary cytotoxic performance of estradiol derivatives on cancerous DU-145, PC3, HeLa and MCF-7 cell lines as well as on non-cancerous human fibroblasts (MRC-5) (compound concentration = 2.5 µM; incubation time = 72 h). Control represents the viability of untreated cells.

With the newly synthesized estradiol-A-ring-integrated benzisoxazole derivatives in our hand, we set off to investigate their in vitro anticancer activity. First, all compounds were solubilized in cell culture grade dimethyl sulfoxide (DMSO) at a final concentration of either 2.5, 5 or 10 mM, respectively, depending on the solubility. Then, each compound was subjected to a preliminary toxicity screen on prostate cancer (DU-145, PC3), cervical cancer (HeLa) and MCF-7 breast cancer cell lines. The non-cancerous MRC-5 cells were also incorporated into the tests to determine the cancer-selective antiproliferative effect of the synthesized molecules. Cells of each cell line were incubated for 72 h with the compounds applied at 2.5 μM concentration, then MTT cell viability assays were carried out. The results of the viability screen (Supplementary Material p51) are shown as a heatmap (Figure 2).

The cytotoxicity screen resulted in a great number of positive hits, whereas the estradiol derivatives exhibited strong antiproliferative activity on cancer cells, in particular, HeLa cells were highly sensitive to the treatments. Furthermore, most compounds presented outstanding cancer cell-selective performance by showing remarkable activity on cancerous cell lines, but no or negligible effect on non-cancerous fibroblasts. To validate these findings, we selected the three most promising derivatives, namely **4b**, **4c** and **4d** (3′-methyl, 3′-ethyl, and 3′-isopropyl substituted steroidal benzisoxazoles), and examined their anticancer efficiency more thoroughly. We assessed the minimal inhibitory concentration (IC_50_) of the compounds by treating DU-145, HeLa, MCF-7 and MRC-5 cells with either **4b**, **4c** or **4d** in 1, 2, 3, 4, 5, 6, 8 and 10 μM concentrations for 72 h and for the viability data, dose-response curves were fitted (Supplementary Material, p52–53) and IC_50_ values were calculated accordingly (Table 1). For comparison, the IC_50_ values of cisplatin on the same cell lines are also included [24].

These results agreed with the primary cytotoxicity screen (Figure 2), as the obtained IC_50_ concentrations verified that the tested compounds were selectively effective and very potent on every cancer cell line involved in the examination. The IC_50_ values of the molecules were at least one but sometimes even two magnitudes higher on non-cancerous MRC-5 cells than on malignant cells. Interestingly, each estradiol-benzisoxazole hybrid was more effective on the three cancer cell lines than the classic chemotherapy drug cisplatin. In fact, cisplatin affected the viability of non-cancerous fibroblasts similarly to steroids; nevertheless, it exhibited significantly weaker antiproliferative capacity on cancer cells than the steroidal chimeras. In summary, potent and tumour cell-selective compounds were found on breast, cervical and prostate cancer cell lines among these E2-benzisoxazole hybrids with 3′-methyl (**4b**), 3′-ethyl (**4c**) and 3′-isopropyl (**4d**) substitution, i.e., this alkyl substitution of the heterocycle proved to be favourable in terms of biological effect and selectivity. 

**Figure 3:**
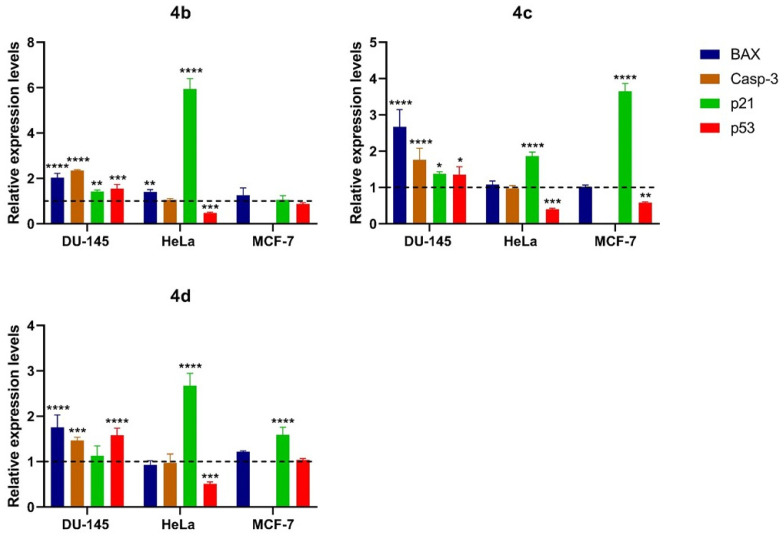
The relative mRNA levels of pro-apoptotic markers BAX, Casp-3, p21, p53 in DU-145, HeLa and MCF-7 cells treated with **4b** (DU-145: 2 μM HeLa: 1 μM, MCF-7 1 μM), **4c** (DU-145: 1 μM HeLa: 1 μM, MCF-7 1 μM) and **4d** (DU-145: 4 μM HeLa: 2 μM, MCF-7 2 μM). Fisher′s LSD test, *: *p* < 0.05, **: *p* < 0.01, ***: *p* < 0.001, ****: *p* < 0.0001.

Lastly, to delineate the possible mechanism of cytotoxicity induced by these novel steroidal heterocycles, we evaluated the apoptosis-inducing potential of the selected three compounds. Most cancer cells try to evade apoptosis and avoid undergoing this form of programmed cell death, which is often the reason behind the ineffectiveness of chemotherapy. Thus, examination of the apoptosis-triggering capacity of newly synthesized anticancer agents is imperative. For this, DU-145, HeLa, and MCF-7 cells were treated with either **4b**, **4c** or **4d** in different concentrations for 72 h. After treatments, total RNA was isolated, reverse transcribed into cDNA and the relative expression levels of some key apoptotic marker genes (BAX, Casp-3, p21, p53) were measured by real-time qPCR (Figure 3). Since MCF-7 cells are known to be deficient in functional caspase-3 due to a deletion in exon 3 of the gene [44], we did not examine the relative transcript levels of this gene in MCF-7 breast cancer cells. 

In accordance with the cytotoxicity data, we found that exposure of cancer cells to any of the three E2-benzisoxazole hybrids induced significant alterations in the expression profile of the examined pro-apoptotic genes (Figure 3). The most sensitive marker was p21 since massive upregulation of p21 expression was observable on each cancer cell line following treatments. Of the three cancerous cell types, DU-145 cells were the most affected, as increased transcript levels of every pro-apoptotic marker, i.e., p21, BAX, Casp-3 as well as p53 were also visible in the case of DU-145 cells.

## 3. Materials and Methods

### 3.1. General

Chemicals, reagents and solvents were purchased from commercial suppliers (Sigma-Aldrich and Alfa Aesar) and used without further purification. Amylene-stabilized dichloromethane was used for the Friedel–Crafts acylations, cyclizations and the Dess–Martin oxidation as solvent. Melting points (Mps) were determined on an SRS Optimelt digital apparatus and are uncorrected. The transformations were monitored by TLC using 0.25 mm thick Kieselgel-G plates (Si 254 F, Merck). The compound spots were detected by spraying with 5% phosphomolybdic acid in 50% aqueous phosphoric acid. Purifications by column chromatograpy (CC) were carried out on silica gel 60, 40–63 µm (Merck) using flash mode. Elementary analysis data were obtained with a PerkinElmer CHN analyzer model 2400. NMR spectra were recorded with a Bruker DRX 500 instrument at RT in CDCl_3_ or DMSO-*d*_6_ using residual solvent signals as an internal reference. Chemical shifts are reported in ppm (*δ* scale) and coupling constants (*J*) are given in Hz. Multiplicities of the ^1^H signals are indicated as a singlet (s), a broad singlet (bs), a doublet (d), a doublet of doublets (dd), a doublet of triplets (dt), a triplet (t), a triplet of doublets (td), a quartet (q) or a multiplet (m). ^13^C NMR spectra are ^1^H-decoupled and the J-MOD pulse sequence was used for multiplicity editing. In this spin-echo type experiment, the signal intensity is modulated by the different coupling constants *J* of carbons depending on the number of attached protons. Both protonated and unprotonated carbons can be detected (CH_3_ and CH carbons appear as positive signals, whereas CH_2_ and C carbons appear as negative signals). The purified derivatives were dissolved in high purity acetonitrile and introduced with an Agilent 1290 Infinity II liquid chromatography pump to an Agilent 6470 tandem mass spectrometer equipped an electrospray ionization chamber. Flow rate was 0.5 mL·min^−1^ and contained 0.1% formic acid or 0.1% ammonium hydroxide to help facilitate ionization. The instrument operated in MS1 scan mode with 135 V fragmentor voltage, and the spectra were recorded from 300 to 500 *m*/*z*, which were corrected with the background.

### 3.2. Chemistry

#### 3.2.1. 3-Methoxyestra-1,3,5(10)-triene-17β-acetate (**1**)

E2Me (2.86 g, 10.0 mmol) was suspended in Ac_2_O (10 mL), and after adding a catalytic amount of H_2_SO_4_ (1 drop), the mixture was sonicated for 5 min. The resulting homogenous solution was poured into ice-cold water (100 mL), and the precipitate was filtered off, washed with water and dried. The crude product was purified by CC (EtOAc/hexane = 5:95). Recrystallized from MeOH/H_2_O. Yield (**1**): 3.12 g (95%, white crystals); Mp: 100–102 °C; Anal. Calcd. for C_21_H_28_O_3_ (328.45) C 76.79; H 8.59. Found C 76.48; H 8.77. ^1^H NMR (500 MHz, CDCl_3_): *δ* 0.83 (3H, s, 18-CH_3_), 1.23–1.64 (7H, overlapping m), 1.70–1.80 (1H, m), 1.89 (2H, m), 2.06 (3H, s, Ac-CH_3_), 2.16–2.27 (2H, m), 2.25–2.33 (1H, m), 2.79–2.93 (2H, m), 3.78 (3H, s), 4.69 (1H, dd, *J* 7.8, 9.2, 17-αH), 6.63 (1H, d, *J* 2.8, 4-H), 6.71 (1H, dd, *J* 2.8, 8.6, 2-H), 7.20 (1H, dd, *J* 1.1, 8.6, 1-H); ^13^C NMR (125 MHz, CDCl_3_) *δ* 12.2 (18-CH_3_), 21.3 (Ac-CH_3_), 23.4 (CH_2_), 26.4 (CH_2_), 27.4 (CH_2_), 27.7 (CH_2_), 29.9 (CH_2_), 37.1 (CH_2_), 38.7 (8-CH), 43.1 (13-C), 44.0 (9-CH), 50.0 (14-CH), 55.3 (3-MeO), 82.9 (17-CH), 111.6 (2-CH), 114.0 (4-CH), 126.5 (1-CH), 132.7 (10-C), 138.0 (5-C), 157.6 (3-C), 171.4 (Ac-C=O); ESI-MS *m*/*z* 329.2 [M + H]^+^, 329.2 calcd. for [C_21_H_29_O_3_]^+^.

#### 3.2.2. General Procedure for the Synthesis of Compounds **2c**–**e** and **2g-17Ac**

Step 1. To a solution of **1** (328 mg, 1.0 mmol) in DCM (10 mL), AlCl_3_ (800 mg, 6 equiv.) and acyl chloride (1.5 equiv.) were added at 0 °C. After 30 min, the ice bath was removed, the reaction mixture was allowed to warm to RT and stirred for another 4 h. The mixture was poured into diluted HCl (1 M, 20 mL), stirred for 10 min and extracted with EtOAc (3 × 20 mL). The combined organic phase was washed with water (30 mL) and brine (30 mL), dried over anhydrous Na_2_SO_4_ and then concentrated in vacuo. The crude product was purified by CC to afford the 17-Ac derivatives of **2c**–**e** or **2g**.

Step 2. Subsequent deacetylation was perfomed (for **2c**-17Ac, **2d**-17Ac and **2d**-17Ac) by the addition of NaOH (3 equiv.) in MeOH/DCM = 1:9 (0.1 M) under stirring at RT for 1 h. After evaporating off the solvents under reduced pressure, HCl (1 M, 10 mL) was added to the residue and extracted with EtOAc (3 × 10 mL). The combined organic phase was washed with water (10 mL) and brine (10 mL), dried over anhydrous Na_2_SO_4_ and concentrated in vacuo. 

2-Propionylestra-1,3,5(10)-triene-3-ol-17β-acetate (**2c**-17Ac) and 2-propionylestra-1,3,5(10)-triene-3,17β-diol (**2c**): According to Section 3.2.2, propionyl chloride (130 µL, 138 mg) was used for Step 1. Eluent (CC): EtOAc/hexane = 10:90. Recrystallized from MeOH. Yield (**2c**-17Ac): 240 mg (65%, white needles); Mp: 169–171 °C; Anal. Calcd. for C_23_H_30_O_4_ (370.49) C 74.56; H 8.16. Found C 74.43; H 8.37. ^1^H NMR (500 MHz, CDCl_3_): *δ* 0.84 (3H, s, 18-CH_3_), 1.23 (3H, t, *J* 7.3, CH_3_ of propionyl), 1.26–1.48 (5H, overlapping m), 1.45–1.55 (1H, m), 1.52–1.62 (1H, m), 1.70–1.79 (1H, m), 1.85–1.95 (2H, m), 2.07 (3H, s, Ac-CH_3_), 2.14–2.26 (2H, m), 2.26–2.34 (1H, m), 2.78–2.92 (2H, m), 2.96–3.05 (2H, m, CH_2_ of propionyl), 4.70 (1H, t, *J* 8.5, 17-αH), 6.69 (1H, s, 4-H), 7.63 (1H, s, 1-H), 12.12 (1H, s, 3-OH); ^13^C NMR (125 MHz, CDCl_3_) *δ* 8.5 (CH_3_ of propionyl), 12.2 (18-CH_3_), 21.3 (Ac-CH_3_), 23.4 (CH_2_), 26.4 (CH_2_), 27.0 (CH_2_), 27.7 (CH_2_), 30.0 (CH_2_), 31.5 (CH_2_), 36.9 (CH_2_), 38.5 (8-CH), 43.0 (13-C), 43.6 (9-CH), 50.0 (14-CH), 82.7 (17-CH), 117.5 (2-C), 117.8 (4-CH), 126.5 (1-CH), 131.4 (C), 146.9 (C), 160.2 (3-C), 171.3 (Ac-C=O), 206.8 (C=O of propionyl); ESI-MS: *m*/*z* 371.2 [M + H]^+^, 371.2 calcd. for [C_23_H_31_O_4_]^+^.

In Step 2, **2c**-17Ac (160 mg, 0.43 mmol) and NaOH (52 mg, 1.30 mmol) were used. The crude product was recrystallized from MeOH. Yield (**2c**): 124 mg (87%, white crystals); Mp: 137–139 °C; Anal. Calcd. for C_21_H_28_O_3_ (328.45) C 76.79; H 8.59. Found C 76.50; H 8.82. ^1^H NMR (500 MHz, CDCl_3_): *δ* 0.80 (3H, s, 18-CH_3_), 1.15–1.27 (1H, m), 1.20–1.28 (3H, m, CH_3_ of propionyl), 1.27–1.62 (7H, overlapping m), 1.66–1.76 (1H, m), 1.85–1.93 (1H, m), 1.95–2.02 (1H, m), 2.08–2.21 (2H, m), 2.29–2.37 (1H, m), 2.85 (2H, m), 2.93–3.05 (2H, m), 3.74 (1H, q, *J* 7.7, 7.7, 7.7), 6.69 (1H, s, 4-H), 7.65 (1H, s, 1-H), 12.12 (1H, s, 3-OH); ^13^C NMR (125 MHz, CDCl_3_) *δ* 8.6 (CH_3_ of propionyl), 11.2 (18-CH_3_), 23.3 (CH_2_), 26.5 (CH_2_), 27.0 (CH_2_), 30.0 (CH_2_), 30.8 (CH_2_), 31.5 (CH_2_), 36.7 (CH_2_), 38.8 (8-CH), 43.3 (13-C), 43.7 (9-CH), 50.2 (14-CH), 82.0 (17-CH), 117.4 (2-C), 117.8 (4-CH), 126.5 (1-CH), 131.6 (C), 147.0 (C), 160.2 (3-C), 206.8 (C=O); ESI-MS: *m*/*z* 329.2 [M + H]^+^, 329.2 calcd. for [C_21_H_29_O_3_]^+^.

2-Isobutyrylestra-1,3,5(10)-triene-3-ol-17β-acetate (**2d**-17Ac) and 2-isobutyrylestra-1,3,5(10)-triene-3,17β-diol (**2d**): According to Section 3.2.2, isobutyryl chloride (157 µL, 160 mg) was used for Step 1. Eluent (CC): EtOAc/hexane = 10:90. Recrystallized from MeOH. Yield (**2d**-17Ac): 258 mg (67%, yellowish white scales); Mp: 167–169 °C; Anal. Calcd. for C_24_H_32_O_4_ (384.52) C 74.97; H 8.39. Found C 74.72; H 8.61. ^1^H NMR (500 MHz, CDCl_3_): *δ* 0.84 (3H, s, 18-CH_3_), 1.24 (6H, dd, *J* 6.4, 6.4, 2 × CH_3_ of isobutyryl), 1.26–1.31 (1H, m), 1.28–1.52 (5H, overlapping m), 1.49–1.62 (1H, m), 1.70–1.80 (1H, m), 1.85–1.95 (2H, m), 2.07 (3H, s, Ac-CH_3_), 2.15–2.29 (2H, m), 2.26–2.34 (1H, m), 2.79–2.93 (2H, m), 3.59 (1H, m, CH of isobutyryl), 4.70 (1H, t, *J* 8.5, 17-αH), 6.71 (1H, s, 4-H), 7.66 (1H, s, 1-H), 12.29 (1H, s, 3-OH); ^13^C NMR (125 MHz, CDCl_3_) *δ* 12.2 (18-CH_3_), 19.5 (one of the CH_3_ of isobutyryl), 19.6 (the other CH_3_ of isobutyryl), 21.3 (Ac-CH_3_), 23.4 (CH_2_), 26.4 (CH_2_), 27.0 (CH_2_), 27.7 (CH_2_), 30.0 (CH_2_), 34.9 (CH), 36.9 (CH_2_), 38.5 (CH), 43.0 (13-C), 43.6 (9-CH), 50.0 (14-CH), 82.7 (17-CH), 116.4 (2-C), 118.0 (4-CH), 126.5 (1-CH), 131.4 (C), 147.0 (C), 160.9 (3-C), 171.3 (Ac-C=O), 210.5 (C=O of isobutyryl); ESI-MS: *m*/*z* 385.2 [M + H]^+^, 385.2 calcd. for [C_24_H_33_O_4_]^+^.

In Step 2, **2d**-17Ac (220 mg, 0.57 mmol) and NaOH (68 mg, 1.70 mmol) were used. Yield (**2d**): 180 mg (92%, white powder); Mp: 130–132 °C; Anal. Calcd. for C_22_H_30_O_3_ (342.48) C 77.16; H 8.83. Found C 76.93; H 8.99. ^1^H NMR (500 MHz, CDCl_3_): *δ* 0.80 (3H, s, 18-CH_3_), 1.16–1.27 (1H, m), 1.24 (6H, dd, *J* 6.8, 6.8, 2 × CH_3_ of isobutyryl), 1.28–1.60 (7H, overlapping m), 1.67–1.77 (1H, m), 1.85–1.93 (1H, m), 1.95–2.03 (1H, m), 2.08–2.23 (2H, m), 2.29–2.38 (1H, m), 2.79–2.93 (2H, m), 3.60 (1H, m, CH of isobutyryl), 3.75 (1H, t, *J* 8.7, 17-αH), 6.71 (1H, s, 4-H), 7.68 (1H, s, 1-H), 12.29 (1H, s, 3-OH); ^13^C NMR (125 MHz, CDCl_3_) *δ* 11.2 (18-CH_3_), 19.5 (one CH_3_ of isobutyryl), 19.6 (the other CH_3_ of isobutyryl), 23.3 (CH_2_), 26.5 (CH_2_), 27.0 (CH_2_), 30.0 (CH_2_), 30.8 (CH_2_), 34.9 (CH), 36.7 (CH_2_), 38.8 (CH), 43.4 (13-C), 43.7 (9-CH), 50.2 (14-CH), 82.0 (17-CH), 116.4 (2-C), 118.0 (4-CH), 126.5 (1-CH), 131.5 (C), 147.1 (C), 160.9 (3-C), 210.5 (C=O); ESI-MS: *m*/*z* 343.2 [M + H]^+^, 343.2 calcd. for [C_22_H_31_O_3_]^+^.

2-Benzoylestra-1,3,5(10)-triene-3-ol-17β-acetate (**2e**-17Ac) and 2-benzoylestra-1,3,5(10)-triene-3,17β-diol (**2e**): According to Section 3.2.2, benzoyl chloride (174 µL, 211 mg) was used for Step 1. Eluent (CC): Et_2_O/hexane = 10:90. Yield (**2e**-17Ac): 272 mg (65%, yellow powder); Mp: 148–150 °C; Anal. Calcd. for C_27_H_30_O_4_ (418.53) C 77.48; H 7.23. Found C 77.40; H 7.57; ^1^H NMR (500 MHz, CDCl_3_): *δ* 0.83 (3H, s, 18-CH_3_), 1.18–1.31 (1H, m), 1.28–1.36 (1H, m), 1.33–1.40 (1H, m), 1.37–1.44 (1H, m), 1.41–1.49 (1H, m), 1.50–1.61 (2H, m), 1.70–1.79 (1H, m), 1.79–1.85 (1H, m), 1.86–1.95 (1H, m), 1.98–2.06 (1H, s), 2.04 (3H, s, Ac-CH_3_), 2.10–2.27 (2H, m), 2.83–2.98 (2H, m), 4.64–4.71 (1H, m, 17-αH), 6.79 (1H, s, 4-H), 7.48 (1H, s, 4-H), 7.46–7.53 (2H, overlapping m, 3′-H and 5′-H), 7.55–7.62 (1H, m, 4′-H), 7.64–7.70 (2H, m, 2′-H and 6′-H), 11.82 (1H, s, 3-OH); ^13^C NMR (125 MHz, CDCl_3_) *δ* 12.2 (18-CH_3_), 21.2 (Ac-CH_3_), 23.4 (CH_2_), 26.2 (CH_2_), 27.0 (CH_2_), 27.7 (CH_2_), 30.1 (CH_2_), 36.9 (CH_2_), 38.6 (8-CH), 43.1 (13-C), 43.6 (9-CH), 50.1 (14-CH), 82.7 (17-CH), 117.4 (2-C), 117.8 (4-CH), 128.4 (3′-CH and 5′-CH), 129.3 (2′-CH and 6′-CH), 130.6 (4′-CH), 131.3 (C), 131.9 (1-CH), 138.5 (C), 147.3 (C), 161.2 (3-C), 171.2 (Ac-C=O), 201.3 (Bz-C=O); ESI-MS: *m*/*z* 419.2 [M + H]^+^, 419.2 calcd. for [C_27_H_31_O_4_]^+^.

In Step 2, **2e**-17Ac (230 mg, 0.55 mmol) and NaOH (66 mg, 1.65 mmol) were used. Yield (**2e**): 186 mg (90%, white powder); Mp: 126–128 °C; Anal. Calcd. for C_25_H_28_O_3_ (376.50) C 79.76; H 7.50. Found C 79.56; H 7.84. ^1^H NMR (500 MHz, CDCl_3_): *δ* 0.77 (3H, s, 18-CH_3_), 1.12–1.54 (8H, overlapping m), 1.65–1.75 (1H, m), 1.84–1.94 (2H, m), 2.00–2.08 (1H, m), 2.05–2.17 (2H, m), 2.79–2.98 (2H, m), 3.66–3.75 (1H, m, 17-αH), 6.79 (1H, s, 4-H), 7.49 (1H, s, 1-H), 7.48–7.55 (2H, m, 3′-H and 5′-H), 7.55–7.62 (1H, m, 4′-H), 7.64–7.70 (2H, m, 2′-H and 6′-H), 11.85 (1H, s, 3-OH); ^13^C NMR (125 MHz, CDCl_3_) *δ* 11.2 (18-CH_3_), 23.2 (CH_2_), 26.2 (CH_2_), 27.0 (CH_2_), 30.2 (CH_2_), 30.7 (CH_2_), 36.6 (CH_2_), 38.8 (8-CH), 43.3 (13-C), 43.7 (9-CH), 50.2 (14-CH), 81.9 (17-CH), 117.3 (2-C), 117.8 (4-CH), 128.5 (2′-CH and 6′-CH), 129.2 (3′-CH and 5′-CH), 130.6 (CH), 131.4 (C), 131.9 (CH), 138.4 (C), 147.4 (C), 161.1 (3-C), 201.4 (C=O); ESI-MS: *m*/*z* 377.2 [M + H]^+^, 377.2 calcd. for [C_25_H_29_O_3_]^+^.

2-(Methyloxoacetyl)-estra-1,3,5(10)-triene-3-ol-17β-acetate (**2g**-17Ac): The synthesis was carried out according to Section 3.2.2, but on a larger scale using E2Me (1.64 g, 5 mmol) and methyl chlorooxoacetate (690 µL, 920 mg) for Step 1. Eluent (CC): EtOAc/hexane = 20:80. Yield (**2g**-17Ac): 1.56 g (78%, yellow crystals); Mp: 134–136 °C; Anal. Calcd. for C_23_H_28_O_6_ (400.47) C 68.98; H 7.05. Found C 68.75; H 7.22. ^1^H NMR (500 MHz, CDCl_3_): *δ* 0.83 (3H, s, 18-CH_3_), 1.22–1.61 (7H, overlapping m), 1.70–1.79 (1H, m), 1.86–1.95 (2H, m), 2.06 (3H, s, Ac-CH_3_), 2.12–2.28 (3H, m), 2.81–2.97 (2H, m), 3.99 (3H, s, COOCH_3_), 4.65–4.72 (1H, m, 17-αH), 6.75 (1H, s, 4-H), 7.58 (1H, s, 1-H), 10.98 (1H, s, 3-OH); ^13^C NMR (125 MHz, CDCl_3_) *δ* 12.2 (18-CH_3_), 21.3 (Ac-CH_3_), 23.4 (CH_2_), 26.1 (CH_2_), 26.8 (CH_2_), 27.7 (CH_2_), 30.3 (CH_2_), 36.7 (CH_2_), 38.3 (8-CH), 43.0 (13-C), 43.4 (9-CH), 50.0 (14-CH), 53.1 (COOCH_3_), 82.7 (17-CH), 114.4 (2-C), 117.9 (4-CH), 128.9 (1-CH), 132.7 (C), 150.1 (C), 161.7 (3-C), 163.2 (COOCH_3_), 171.3 (Ac-C=O), 189.8 (C=O of ketone); ESI-MS: *m*/*z* 399.0 [M − H]^−^, 399.2 calcd. for [C_23_H_27_O_6_]^−^.

**2g**-17Ac was not deacetylated in Step 2.

#### 3.2.3. 2-Trifluoracetylestra-1,3,5(10)-triene-3,17β-diol (**2f**)

To a solution of E2 (272 mg, 1.0 mmol) in DCM (10 mL), AlCl_3_ (800 mg, 6 equiv.) and TFAA (1.5 mmol, 1.5 equiv.) were added and the mixture was stirred at 0 °C for 4 h. After completion, it was poured into HCl solution (1 M, 20 mL), stirred for 10 min and extracted with EtOAc (3 × 20 mL). The combined organic phase was washed with water (30 mL) and brine (30 mL), then dried over anhydrous Na_2_SO_4_ and concentrated in vacuo. The crude product was purified by CC (DCM). Recrystallized from dioxane/H_2_O. Yield (**2f**): 302 mg (82%, yellow crystals); Mp: 104–106 °C; Anal. Calcd. for C_20_H_23_F_3_O_3_ (368.40) C 65.21; H 6.29. Found C 65.12; H 6.48. ^1^H NMR (500 MHz, CDCl_3_): *δ* 0.79 (3H, s, 18-CH_3_), 1.15–1.24 (1H, m), 1.24–1.33 (1H, m), 1.30–1.48 (4H, m), 1.45–1.60 (2H, m), 1.66–1.76 (1H, m), 1.87–1.95 (1H, m), 1.96–2.05 (1H, m), 2.08–2.25 (2H, m), 2.25–2.34 (1H, m), 2.82–2.98 (2H, m), 3.71–3.78 (1H, t, *J* 8.5, 17-αH), 6.79 (1H, s, 4-CH), 7.69 (1H, s, 1-H), 10.86 (1H, s, 3-OH); ^13^C NMR (125 MHz, CDCl_3_) *δ* 11.2 (18-CH_3_), 23.2 (CH_2_), 26.1 (CH_2_), 26.7 (CH_2_), 30.4 (CH_2_), 30.7 (CH_2_), 36.5 (CH_2_), 38.5 (8-CH), 43.3 (13-C), 43.6 (9-CH), 50.2 (14-CH), 81.9 (17-CH), 112.2 (2-C), 116.8 (1C, q, *J* 290.2, CF_3_), 118.3 (4-CH), 127.2 (1C, q, *J* 3.9, 1-CH), 133.2 (C), 151.1 (C), 162.4 (C), 183.9 (1C, q, *J* 34.8, C=O); ESI-MS: *m*/*z* 367.0 [M − H]^−^, 367.2 calcd. for [C_20_H_22_F_3_O_3_]^−^.

#### 3.2.4. General Procedure for the Synthesis of Oximes **3a**–**g**

To a solution of 2-substituted estradiol derivative (**2a**–**g**, 1.0 mmol) in EtOH (10 mL), hydroxylamine hydrochloride and a base were added in excess and the mixture was stirred at ambient temperature for a certain period. The solvent was removed under reduced pressure, and the residue was suspended in water (10 mL) and extracted with EtOAc (3 × 10 mL). The combined organic phase was washed with NH_4_Cl (1 M, 10 mL), water (10 mL) and brine (10 mL), and then dried over anhydrous Na_2_SO_4_ and concentrated *in vacuo*. The crude product was purified by CC.

Estra-1,3,5(10)-triene-3,17β-diol-2-carbaldehyde oxime (**3a**): According to Section 3.2.4, 2-formyl-estradiol (**2a**, 300 mg), hydroxylamine hydrochloride (104 mg, 1.5 equiv.) and sodium acetate (164 mg, 2 equiv.) were used. Conditions: RT, 1 h. CC (EtOAc/DCM = 5:95). Yield (**3a**): 309 mg (98%, white powder); Mp > 180 °C (decomp.); Anal. Calcd. for C_19_H_25_NO_3_ (315.41) C 72.35; H 7.99; N 4.44. Found C 72.30; H 8.17; N 4.33. ^1^H NMR (500 MHz, DMSO-*d*_6_): *δ* 0.67 (3H, s, 18-CH_3_), 1.05–1.44 (7H, overlapping m), 1.53–1.63 (1H, m), 1.74–1.82 (1H, m), 1.82–1.94 (2H, m), 2.03–2.12 (1H, m), 2.20–2.28 (1H, m), 2.71–2.77 (2H, m), 3.53 (1H, td, *J* 8.5, 3.9, 17-αH), 4.41 (1H, d, *J* 4.7, 17-OH), 6.56 (1H, s, 4-H), 7.34 (1H, s, 1-H), 8.27 (1H, s, HC=N), 9.75 (1H, s, NOH), 11.11 (1H, s, 3-OH); ^13^C NMR (125 MHz, DMSO-*d*_6_): *δ* 11.1 (18-CH_3_), 22.6 (CH_2_), 25.9 (CH_2_), 26.6 (CH_2_), 28.9 (CH_2_), 29.8 (CH_2_), 36.4 (CH_2_), 38.4 (8-CH), 42.7 (13-C), 43.2 (9-CH), 49.5 (14-CH), 79.9 (17-CH), 115.4 (4-CH), 115.5 (2-C), 125.0 (1-CH), 131.3 (10-C), 139.2 (5-C), 148.6 (HC=N), 153.7 (3-C); ESI-MS: *m*/*z* 314.0 [M − H]^−^, 314.2 calcd. for [C_19_H_24_NO_3_]^−^.

(Estra-1,3,5(10)-triene-3,17β-diol-2-yl)ethan-1-one oxime (**3b**): According to Section 3.2.4, 2-acetyl-estradiol (**2b**, 314 mg, 1.0 mmol), hydroxylamine hydrochloride (104 mg, 1.5 equiv.) and sodium acetate (164 mg, 2 equiv.) were used. Conditions: reflux, 2 h. CC (EtOAc/DCM = 5:95). Yield: 309 mg (94%, white crystals); Mp: 240–242 °C; Anal. Calcd. for C_20_H_27_NO_3_ (C_20_H_27_NO_3_) C 72.92; H 8.26; N 4.25. Found C 72.86; H 8.58; N 4.03. ^1^H NMR (500 MHz, CDCl_3_): *δ* 0.79 (3H, s, 18-CH_3_), 1.15–1.58 (8H, overlapping m), 1.66–1.76 (1H, m), 1.84–1.93 (1H, m), 1.93–2.06 (1H, m), 2.07–2.25 (2H, m), 2.29–2.37 (1H, m), 2.32–2.36 (3H, s, CH_3_ of acetoxime), 2.80–2.87 (2H, m), 3.74 (1H, t, *J* 8.5, 17-αH), 6.68 (1H, s, 4-H), 7.32 (1H, s, 1-H), 7.65 (1H, s, NOH), 10.94 (1H, s, 3-OH); ^13^C NMR (125 MHz, CDCl_3_) *δ* 10.9 (CH_3_), 11.2 (CH_3_), 23.3 (CH_2_), 26.6 (CH_2_), 27.3 (CH_2_), 29.5 (CH_2_), 30.8 (CH_2_), 36.8 (CH_2_), 39.0 (8-CH), 43.4 (13-C), 44.0 (9-CH), 50.2 (14-CH), 82.1 (17-CH), 116.4 (2-C), 117.0 (4-CH), 124.5 (1-CH), 131.2 (10-C), 140.3 (5-C), 155.5 (C), 159.8 (C); ESI-MS: *m*/*z* 328.0 [M − H]^−^, 328.2 calcd. for [C_20_H_26_NO_3_]^−^.

(Estra-1,3,5(10)-triene-3,17β-diol-2-yl)propan-1-one oxime (**3c**): According to Section 3.2.4, 2-propanoyl-estradiol (**2c**, 328 mg), hydroxylamine hydrochloride (104 mg, 1.5 equiv.) and sodium acetate (164 mg, 2 equiv.) were used. Conditions: reflux, overnight. CC (MeOH/DCM = 2:98). Yield (**3c**): 333 mg (97%, white crystals); Mp: 240–242 °C; Anal. Calcd. for C_21_H_29_NO_3_ (343.47) C 73.44; H 8.51; N 4.08. Found C 73.10; H 8.84; N 3.97. ^1^H NMR (500 MHz, DMSO-*d*_6_): *δ* 0.67 (3H, s, 18-CH_3_), 1.08 (3H, t, *J* 7.5, CH_3_ of propionyl oxime), 1.07–1.43 (7H, overlapping m), 1.54–1.63 (1H, m), 1.75–1.82 (1H, m), 1.83–1.94 (2H, m), 2.07–2.16 (1H, m), 2.28–2.35 (1H, m), 2.71–2.86 (4H, overlapping m, 6-H_2_ and CH_2_ of propionyl oxime), 3.53 (1H, td, *J* 8.5, 4.8, 17-αH), 4.45–4.50 (1H, m, 17-OH), 6.55 (1H, s, 4-H), 7.27 (1H, s, 1-H), 11.27 (1H, s), 11.31 (1H, s); ^13^C NMR (125 MHz, DMSO-*d*_6_): *δ* 11.0 (CH_3_), 11.2 (CH_3_), 17.6 (CH_2_), 22.7 (CH_2_), 26.0 (CH_2_), 26.7 (CH_2_), 28.8 (CH_2_), 29.9 (CH_2_), 36.5 (CH_2_), 38.5 (8-CH), 42.8 (13-C), 43.4 (9-CH), 49.5 (14-CH), 80.0 (17-CH), 115.5 (2-C), 116.2 (4-CH), 124.1 (1-CH), 130.6 (C), 138.8 (C), 155.1 (3-C), 162.2 (C=N); ESI-MS: *m*/*z* 344.2 [M + H]^+^, 344.2 calcd. for [C_21_H_30_NO_3_]^+^.

(Estra-1,3,5(10)-triene-3,17β-diol-2-yl)-2-methylpropan-1-one oxime isomers: According to Section 3.2.4, 2-isobutyryl-estradiol (**2d**, 342 mg), hydroxylamine hydrochloride (347 mg, 5 equiv.) and pyridine (1 mL) were used. Conditions: reflux, overnight. CC (MeOH/DCM = 2:98). Yield of the faster eluting isomer (**3d**-*E*): 175 mg (49%, white crystals); Mp: 211–213 °C; Anal. Calcd. for C_22_H_31_NO_3_ (357.49) C 73.92; H 8.74; N 3.92. Found C 73.80; H 9.10; N 3.86. ^1^H NMR (500 MHz, DMSO-*d*_6_): *δ* 0.66 (3H, s, 18-CH_3_), 1.14 (6H, dd, *J* 6.9, 6.9, 2 × CH_3_ of isobutyryl oxime), 1.06–1.25 (2H, m), 1.24 (1H, s), 1.24–1.35 (3H, m), 1.35–1.43 (1H, m), 1.53–1.63 (1H, m), 1.74–1.81 (1H, m), 1.81–1.94 (2H, m), 2.04–2.13 (1H, m), 2.19–2.26 (1H, m), 2.66–2.75 (2H, m), 3.38 (1H, m, CH of isobutyryl oxime), 3.52 (1H, td, *J* 8.5, 4.8, 17-αH), 4.47 (1H, d, *J* 4.8, 17-OH), 6.51 (1H, s, 4-H), 7.03 (1H, s, 1-H), 10.00 (1H, s), 10.85 (1H, s); ^13^C NMR (125 MHz, DMSO-*d*_6_): *δ* 11.2 (18-CH_3_), 18.7 (2 × CH_3_ of isobutyryl oxime), 22.7 (CH_2_), 26.1 (CH_2_), 26.8 (CH_2_), 27.3 (CH of isobutyryl oxime), 28.8 (CH_2_), 29.9 (CH_2_), 36.5 (CH_2_), 38.6 (8-CH), 42.8 (13-C), 43.3 (9-CH), 49.5 (14-CH), 80.0 (17-CH), 115.6 (4-CH), 119.2 (2-C), 126.0 (1-CH), 130.0 (C), 137.6 (C), 153.8 (C), 162.9 (C); ESI-MS: *m*/*z* 358.2 [M + H]^+^, 358.2 calcd. for [C_22_H_32_NO_3_]^+^.

Yield of the slower eluting isomer (**3d**-*Z*): 121 mg (34%, white crystals); Mp: 214–216 °C; Anal. Calcd. for C_22_H_31_NO_3_ (357.49) C 73.92; H 8.74; N 3.92. Found C 73.71; H 9.03; N 3.68. ^1^H NMR (500 MHz, DMSO-*d*_6_): *δ* 0.66 (3H, s, 18-CH_3_), 1.02 and 1.03 (6H, overlapping d, 2 × CH_3_ of isobutyryl oxime), 1.06–1.43 (7H, overlapping m), 1.53–1.63 (1H, m), 1.74–1.93 (3H, m), 2.03–2.12 (1H, m), 2.15–2.22 (1H, m), 2.65–2.76 (3H, m), 3.48–3.56 (1H, m, 17-αH), 4.45–4.49 (1H, d, *J* 4.8, 17-OH), 6.49 (1H, s, 4-H), 6.78 (1H, s, 1-H), 8.74 (1H, bs), 10.26 (1H, bs); ^13^C NMR (125 MHz, DMSO-*d*_6_): *δ* 11.2 (18-CH_3_), 20.2 (one CH_3_ of isobutyryl oxime), 20.2 (the other CH_3_ of isobutyryl oxime), 22.7 (CH_2_), 26.1 (CH_2_), 26.9 (CH_2_), 28.9 (CH_2_), 29.9 (CH_2_), 33.4 (CH of isobutyryl oxime), 36.5 (CH_2_), 38.6 (8-CH), 42.8 (13-C), 43.4 (9-CH), 49.5 (14-CH), 80.0 (17-CH), 115.3 (4-CH), 120.0 (2-C), 125.5 (1-CH), 130.0 (C), 136.8 (C), 151.4 (C), 159.7 (C); ESI-MS: *m*/*z* 358.2 [M + H]^+^, 358.2 calcd. for [C_22_H_32_NO_3_]^+^.

(Estra-1,3,5(10)-triene-3,17β-diol-2-yl)(phenyl)methanone oxime isomers (**3e**): According to Section 3.2.4, 2-benzoyl-estradiol (**2e**, 377 mg), hydroxylamine hydrochloride (347 mg, 5 equiv.) and pyridine (1 mL) were used. Conditions: reflux, 48 h. CC (EtOAc/DCM = 2:98 to 5:95). Yield of the faster eluting isomer (**3e**-*E*): Yield: 172 mg (44%, white powder); Mp: 280–282 °C; Anal. Calcd. for C_25_H_29_NO_3_ (391.51) C 76.70; H 7.47; N 3.58. Found C 76.38; H 7.73; N 3.49. ^1^H NMR (500 MHz, DMSO-*d*_6_): *δ* 0.61 (3H, s, 18-CH_3_), 0.99–1.40 (7H, overlapping m), 1.50–1.60 (1H, m), 1.61–1.71 (2H, m), 1.72–1.80 (1H, m), 1.80–1.91 (1H, m), 1.92–2.01 (1H, m), 2.71–2.77 (2H, m), 3.42–3.50 (1H, td, *J* 8.5, 4.8, 17-αH), 4.41–4.46 (1H, d, *J* 4.9, 17-OH), 6.62 (1H, s), 6.67 (1H, s), 7.25–7.31 (2H, m), 7.42–7.53 (3H, m), 11.00 (1H, s), 11.40 (1H, s); ^13^C NMR (125 MHz, DMSO-*d*_6_): *δ* 11.1 (18-CH_3_), 22.7 (CH_2_), 25.7 (CH_2_), 26.6 (CH_2_), 28.8 (CH_2_), 29.9 (CH_2_), 36.2 (CH_2_), 38.4 (8-CH), 42.7 (13-C), 43.1 (9-CH), 49.4 (14-CH), 79.9 (17-CH), 116.1 (4-CH), 117.0 (2-C), 126.5 (1-CH), 128.2 (2′-CH and 6′-CH), 128.4 (3′-CH and 5′-CH), 128.6 (4′-CH), 130.4 (C), 132.1 (C), 139.1 (C), 155.0 (C), 159.1 (C); ESI-MS: *m*/*z* 390.1 [M − H]^−^, 390.2 calcd. for [C_25_H_28_NO_3_]^−^.

Yield of the slower eluting isomer (**3e**-*Z*): 141 mg (36%, white powder); Mp: 277–279 °C; Anal. Calcd. for C_25_H_29_NO_3_ (391.51) C 76.70; H 7.47; N 3.58. Found C 76.47; H 7.69; N 3.20. ^1^H NMR (500 MHz, DMSO-*d*_6_): *δ* 0.67 (3H, s, 18-CH_3_), 1.02–1.43 (7H, overlapping m), 1.54–1.64 (1H, m), 1.75–1.84 (2H, m), 1.83–1.94 (1H, m), 2.06–2.18 (2H, m), 2.74–2.81 (2H, m), 3.51 (1H, td, *J* 8.5, 4.8, 17-αH), 4.46 (1H, d, *J* 4.8, 17-OH), 6.57 (1H, s), 6.83 (1H, s), 7.29–7.33 (3H, m), 7.37–7.42 (2H, m), 8.91 (1H, s), 11.04 (1H, s); ^13^C NMR (125 MHz, DMSO-*d*_6_): *δ* 11.2 (18-CH_3_), 22.7 (CH_2_), 26.1 (CH_2_), 26.9 (CH_2_), 29.0 (CH_2_), 29.9 (CH_2_), 36.5 (CH_2_), 38.6 (8-CH), 42.8 (13-C), 43.4 (9-CH), 49.5 (14-CH), 80.0 (17-CH), 115.4 (4-CH), 118.6 (2-CH), 126.2 (1-CH), 126.3 (2′-CH and 6′-CH), 128.1 (3′-CH and 5′-CH), 128.3 (4′-CH), 130.3 (C), 136.8 (C), 137.4 (C), 151.9 (C), 153.9 (C); ESI-MS: *m*/*z* 390.1 [M − H]^−^, 390.2 calcd. for [C_25_H_28_NO_3_]^−^.

(Estra-1,3,5(10)-triene-3,17β-diol-2-yl)-2,2,2-trifluoroethan-1-one oxime isomers (**3f**): According to Section 3.2.4, 2-trifluoracetyl-estradiol (**2f**, 356 mg), hydroxylamine hydrochloride (695 mg, 10 equiv.) and pyridine (1.5 mL) were used. Conditions: reflux, overnight. CC (EtOAc/hexane = 20:80) yielded an inseparable mixture of *Z*- and *E*-isomers (1:2). Yield (**3f**): 364 mg (95%, white powder); Mp: 180–182 °C; Anal. Calcd. for C_20_H_24_F_3_NO_3_ (383.41) C 62.65; H 6.32; N 3.29. Found C 62.60; H 6.57; N 3.04. ^1^H NMR (500 MHz, DMSO-*d*_6_): *δ* 0.66 (3H, s, 18-CH_3_), 1.05–1.43 (7H, overlapping m), 1.53–1.63 (1H, m), 1.75–1.94 (3H, m), 2.04–2.13 (1H, td, *J* 11.2, 3.9), 2.14–2.25 (1H, m), 2.68–2.81 (2H, dd, *J* 10.0, 5.9), 3.48–3.58 (1H, td, *J* 8.5, 4.7, 17-αH), 4.45–4.49 (1H, d, *J* 4.8, 17-OH), 6.56 and 6.58 (1H, s, 4-H of *E*- and *Z*-isomers), 6.88 and 6.98 (1H, s, 1-H of *E*- and *Z*-isomers), 9.45 and 9.61 (1H, bs and s, 3-OH of *E*- and *Z*-isomers), 12.21 and 12.53 (1H, bs and s, NOH of *E*- and *Z*-isomers); ^13^C NMR (125 MHz, DMSO-*d*_6_): *δ* 11.2 (18-CH_3_), 22.7 (CH_2_), 26.0 (CH_2_), 26.7 (CH_2_), 29.0 (6-CH_2_ of *Z*-isomer), 29.0 (6-CH_2_ of *E*-isomer), 29.9 (CH_2_), 36.5 (12-CH_2_ of *E*-isomer), 36.5 (12-CH_2_ of *Z*-isomer) 38.4 (8-CH), 42.8 (13-C), 43.2 (9-CH of *Z*-isomer), 43.3 (9-CH of *E*-isomer), 49.5 (14-CH), 80.0 (17-CH), 112.3 (2-C), 115.1 (1-CH of *Z*-isomer), 115.4 (4-CH of *E*-isomer), 116.0 (2-C), 118.2. (1C, q, *J* 282.2, CF_3_ of *Z*-isomer), 121.2 (1C, q, *J* 273.7, CF_3_ of *E*-isomer), 125.8 (1-CH of *E*-isomer), 127.4 (1-CH of *Z*-isomer), 130.6 (10-C of *E*-isomer), 130.7 (10-C of *Z*-isomer, 139.5 (5-C of *E*-isomer), 139.8 (5-C of *Z*-isomer), 144.5 (1C, q, *J* 32.1, C=N of *E*-isomer), 145.1 (1C, q, *J* 30.3, C=N of *Z*-isomer), 152.7 (3-C of *E*-isomer), 153.9 (3-C of *Z*-isomer); ESI-MS: *m*/*z* 382.0 [M − H]^−^, 382.2 calcd. for [C_20_H_23_F_3_NO_3_]^−^.

Methyl-2-(estra-1,3,5(10)-triene-3-hydroxy-17β-acetoxy-2-yl)-2-hydroxyimino acetate (**3g**): According to Section 3.2.4, 2-(methyloxoacetyl)estradiol-17β-acetate (**2g***-17Ac*, 400 mg), hydroxylamine hydrochloride (104 mg, 1.5 equiv.) and pyridine (1 mL) were used. The synthesis was repeated in duplicate. Conditions: RT, overnight. CC (EtOAc/hexane = 20:80). Yield (**3g**): 378 mg (91%, white powder, as a single isomer); Mp: 188–189 °C. Anal. Calcd. for C_23_H_29_NO_6_ (415.49) C 66.49; H 7.04; N 3.37. Found C 66.35; H 7.42; N 3.24. ^1^H NMR (500 MHz, DMSO-*d*_6_): *δ* 0.77 (3H, s, 17-CH_3_), 1.22–1.41 (6H, overlapping m), 1.44–1.55 (1H, m), 1.63–1.72 (1H, m), 1.74–1.84 (2H, m), 2.01 (3H, s, Ac-CH_3_), 2.04–2.20 (3H, m), 2.71–2.78 (2H, m), 3.77 (3H, s, COOCH_3_), 4.57–4.64 (1H, t, *J* 8.5, 17-αH), 6.56 (1H, s, 4-H), 7.22 (1H, s, 1-H), 9.74 (1H, s, 3-OH), 11.72 (1H, s, NOH); ^13^C NMR (125 MHz, DMSO-*d*_6_): *δ* 11.8 (18-CH_3_), 20.8 (Ac-CH_3_), 22.7 (CH_2_), 25.7 (CH_2_), 26.5 (CH_2_), 27.1 (CH_2_), 28.8 (CH_2_), 36.3 (CH_2_), 37.9 (8-CH), 42.4 (13-C), 42.8 (9-CH), 49.0 (14-CH), 51.8 (COOCH_3_), 81.8 (17-CH), 114.7 (2-C), 116.0 (4-CH), 124.0 (1-CH), 131.1 (10-C), 140.0 (5-C), 149.6 (C=N), 153.3 (3-C), 163.9 (COOCH_3_), 170.3 (Ac-C=O); ESI-MS: *m*/*z* 414.1 [M − H]^−^, 414.2 calcd. for [C_23_H_28_NO_6_]^−^.

#### 3.2.5. General Procedure for the Synthesis of Compounds **4a**–**g**

DDQ (1.5 equiv.) was added slowly to a solution of PPh_3_ (1.5 equiv.) in DCM (0.5 M), and stirred for 1 min. This suspension was added to a solution of oxime (**3a**–**g**, 1.0 equiv.) and Et_3_N (2.0 equiv.) in DCM (0.2 M), and stirred for 10 min at RT. The solvent was removed under reduced pressure. The residue was dissolved in EtOAc/MeOH, and Celite^®^ was added (~10× weight of the crude sample), which was then concentrated in vacuo and purified by CC. 

Isoxazolo[4′,5′:2,3]estra-1,3,5(10)-triene-17β-ol (**4a**): According to Section 3.2.5, **3a** (158 mg, 0.50 mmol) was used for the reaction. Eluent (CC): EtOAc/hexane = 30:70. Yield (**4a**): 134 mg (90%, white powder); Mp: 176–178 °C; Anal. Calcd. for C_19_H_23_NO_2_ (297.40) C 76.74; H 7.80; N 4.71. Found C 76.55; H 7.96; N 4.33. ^1^H NMR (500 MHz, CDCl_3_): *δ* 0.80 (3H, s, 18-CH_3_), 1.18–1.57 (7H, overlapping m), 1.58–1.68 (1H, m), 1.70–1.78 (1H, m), 1.89–2.08 (2H, m), 2.09–2.20 (1H, m), 2.27–2.35 (1H, m), 2.35–2.44 (1H, m), 3.00–3.06 (2H, m), 3.76 (1H, t, *J* 8.5, 17-αH), 7.32 (1H, s, 4-H), 7.61 (1H, s, 1-H), 8.60 (1H, d, *J* 1.1, 3′-H); ^13^C NMR (125 MHz, CDCl_3_) *δ* 11.2 (18-CH_3_), 23.4 (CH_2_), 26.5 (CH_2_), 27.0 (CH_2_), 30.4 (CH_2_), 30.7 (CH_2_), 36.7 (CH_2_), 38.6 (8-CH), 43.3 (13-C), 44.2 (9-CH), 50.5 (14-CH), 81.9 (17-CH), 108.8 (4-CH), 117.8 (2-CH), 119.7 (1-C), 137.6 (10-C), 141.0 (5-C), 146.2 (3′-CH), 161.1 (3-C); ESI-MS: *m*/*z* 296.0 [M − H]^−^, 296.2 calcd. for [C_19_H_22_NO_2_]^−^.

3′-Methylisoxazolo[4′,5′:2,3]estra-1,3,5(10)-triene-17β-ol (**4b**): According to Section 3.2.5, **3b** (150 mg, 0.46 mmol) was used for the reaction. Eluent (CC): EtOAc/hexane = 30:70. Yield (**4b**): 132 mg (93%, white crystals); Mp: 168–170 °C; Anal. Calcd. for C_20_H_25_NO_2_ (311.42) C 77.14; H 8.09; N 4.50. Found C 77.10; H 8.34; N 4.26. ^1^H NMR (500 MHz, CDCl_3_): *δ* 0.80 (3H, s 18-CH_3_), 1.19–1.32 (1H, m), 1.31–1.56 (6H, overlapping m), 1.59–1.79 (2H, m), 1.89–1.99 (1H, m), 2.02 (1H, m), 2.09–2.20 (1H, m), 2.27–2.36 (1H, m), 2.39–2.47 (1H, m), 2.54 (3H, s, 3′-CH_3_), 2.99–3.05 (2H, m), 3.72–3.80 (1H, m, 17-αH), 7.24 (1H, s, 4-H), 7.50 (1H, s, 1-H); ^13^C NMR (125 MHz, CDCl_3_) *δ* 10.2 (CH_3_), 11.2 (CH_3_), 23.4 (CH_2_), 26.6 (CH_2_), 27.1 (CH_2_), 30.4 (CH_2_), 30.8 (CH_2_), 36.7 (CH_2_), 38.7 (8-CH), 43.3 (13-C), 44.2 (9-CH), 50.5 (14-CH), 82.0 (17-CH), 109.0 (4-CH), 116.9 (1-CH), 120.6 (2-C), 136.9 (10-C), 140.5 (5-C), 154.9 (C=N), 161.6 (3-C); ESI-MS: *m*/*z* 312.2 [M − H]^+^, 312.2 calcd. for [C_20_H_26_NO_2_]^+^.

3′-Ethylisoxazolo[4′,5′:2,3]estra-1,3,5(10)-triene-17β-ol (**4c**): According to Section 3.2.5, 3**c** (50 mg, 0.15 mmol) was used for the reaction. Eluent (CC): DCM. Yield (**4c**): 43 mg (93%, white crystals); Mp: 166–168 °C; Anal. Calcd. for C_21_H_27_NO_2_ (325.45) C 77.50; H 8.36; N 4.30. Found C 77.38; H 8.67; N 4.16. ^1^H NMR (500 MHz, CDCl_3_): *δ* 0.82 (3H, s, 18-CH_3_), 1.21–1.32 (1H, m), 1.31–1.56 (6H, overlapping m), 1.45 (3H, t, *J* 7.4, 7.4, CH_3_ of ethyl), 1.60–1.73 (1H, m), 1.70–1.80 (1H, m), 1.90–1.98 (1H, m), 1.99–2.07 (1H, m), 2.10–2.21 (1H, m), 2.28–2.37 (1H, m), 2.39–2.48 (1H, m), 2.95–3.06 (4H, m), 3.73–3.81 (1H, m, 17-αH), 7.27 (1H, s, 4-CH), 7.54 (1H, s, 1-CH); ^13^C NMR (125 MHz, CDCl_3_) *δ* 11.2 (18-CH_3_), 12.4 (CH_3_ of ethyl), 19.1 (CH_2_), 23.4 (CH_2_), 26.6 (CH_2_), 27.1 (CH_2_), 30.4 (CH_2_), 30.8 (CH_2_), 36.7 (CH_2_), 38.7 (8-CH), 43.4 (13-C), 44.3 (9-CH), 50.5 (14-CH), 82.0 (17-CH), 109.1 (4-CH), 117.0 (1-CH), 119.9 (C), 136.8 (C), 140.4 (C), 159.6 (C), 161.8 (C); ESI-MS: *m*/*z* 326.2 [M + H]^+^, 326.2 calcd. for [C_21_H_28_NO_2_]^+^.

3′-Isopropylisoxazolo[4′,5′:2,3]estra-1,3,5(10)-triene-17β-ol (**4d**): According to Section 3.2.5, **3d** (80 mg, 0.22 mmol, 1:1 mixture of *E* and *Z*-oximes) was used for the reaction. Eluent (CC): EtOAc/hexane = 40:60. Yield (**4d**): 68 mg (91%, white crystals); Mp: 168–169 °C; Anal. Calcd. for C_22_H_29_NO_2_ (339.48) C 77.84; H 8.61; N 4.13. Found C 77.58; H 8.74; N 4.02. ^1^H NMR (500 MHz, CDCl_3_): *δ* 0.81 (3H, s, 18-CH_3_), 1.21–1.30 (1H, m), 1.31–1.47 (4H, overlapping m), 1.49 (6H, d, *J* 7.1, 2 × CH_3_ of *^i^*Pr), 1.47–1.56 (2H, m), 1.59–1.79 (2H, m), 1.90–1.97 (1H, m), 1.99–2.06 (1H, m), 2.10–2.21 (1H, m), 2.28–2.37 (1H, m), 2.39–2.46 (1H, m), 2.99–3.05 (2H, m), 3.38 (1H, m, CH of *^i^*Pr), 3.73–3.81 (1H, m, 17-αH), 7.27 (1H, s, 4-H), 7.58 (1H, s, 1-H); ^13^C NMR (125 MHz, CDCl_3_) *δ* 11.2 (18-CH_3_), 21.3 (one CH_3_ of *^i^*Pr), 21.4 (the other CH_3_ of *^i^*Pr), 23.4 (CH_2_), 26.6 (CH_2_), 27.0 (CH of *^i^*Pr), 27.1 (CH_2_), 30.4 (CH_2_), 30.8 (CH_2_), 36.7 (CH_2_), 38.7 (8-CH), 43.4 (13-C), 44.3 (9-CH), 50.5 (14-CH), 82.0 (17-CH), 109.2 (4-CH), 117.4 (1-CH), 119.2 (2-C), 136.6 (C), 140.3 (C), 162.0 (C), 163.1 (C); ESI-MS: *m*/*z* 340.2 [M + H]^+^, 340.2 calcd. for [C_22_H_30_NO_2_]^+^.

3′-Phenylisoxazolo[4′,5′:2,3]estra-1,3,5(10)-triene-17β-ol (**4e**): Method A (Scheme 1*,* iii)*:* According to Section 3.2.5, **3e** (100 mg, 0.26 mmol, 1:1 mixture of *E* and *Z*-oximes) was used for the reaction. Eluent (CC): EtOAc/DCM = 2:98. Yield (**4e**): 24 mg (25%, yellowish white solid); Method B (Scheme 1, vii/viii): **2e** (62 mg, 0.16 mmol) was dissolved in NH_3_ (6 M in MeOH, 0.5 mL) and stirred for 2 h at RT. The solvent was then removed under reduced pressure, and the residue (**5e**) was redissolved in THF (1 mL). NCS (33 mg, 1.5 equiv.) and K_2_CO_3_ (69 mg, 3.0 equiv.) were added to the solution, and the resulting suspension was stirred at RT overnight. After evaporating off the solvent, the residue was suspended in water (10 mL) and extracted with EtOAc (3 × 10 mL). The combined organic phase was washed with HCl (1M, 10 mL), water (10 mL) and brine (10 mL), dried over anhydrous Na_2_SO_4_ and concentrated in vacuo. The crude product was purified by CC (DCM). Yield (**4e**): 37 mg (60%, as yellowish white solid); Mp: 196–198 °C; Anal. Calcd. for C_25_H_27_NO_2_ (373.50) C 80.40; H 7.29; N 3.75. Found C 80.02; H 7.56; N 3.55. ^1^H NMR (500 MHz, CDCl_3_): *δ* 0.81 (3H, s, 18-CH_3_), 1.20–1.53 (7H, overlapping m), 1.61–1.80 (2H, m), 1.86–1.99 (1H, m), 1.99–2.09 (1H, m), 2.09–2.21 (1H, m), 2.31–2.39 (1H, m), 2.40–2.47 (1H, m), 3.03–3.09 (2H, m), 3.74–3.80 (1H, t, *J* 8.5, 17-αH), 7.35 (1H, s, 4-H), 7.47–7.60 (3H, overlapping m, 3″-H, 4″-H and 5″-H), 7.78 (1H, s, 1-H), 7.90–7.97 (2H, m, 2″-H and 6″-H); ^13^C NMR (125 MHz, CDCl_3_) *δ* 11.2 (18-CH_3_), 23.4 (CH_2_), 26.7 (CH_2_), 27.1 (CH_2_), 30.4 (CH_2_), 30.8 (CH_2_), 36.8 (CH_2_), 38.8 (8-CH), 43.4 (13-C), 44.4 (9-CH), 50.6 (14-CH), 82.0 (17-CH), 109.3 (4-CH), 117.9 (1-CH), 119.0 (2-C), 128.2 (2″-CH and 6″-CH), 129.2 (3″-CH and 5″-CH), 129.6 (1″-C), 130.2 (4″-CH), 137.7 (C), 140.7 (C), 157.3 (C), 162.8 (C); ESI-MS: *m*/*z* 374.2 [M + H]^+^, 374.2 calcd. for [C_25_H_28_NO_2_]^+^.

3′-Trifluoromethylisoxazolo[4′,5′:2,3]estra-1,3,5(10)-triene-17β-ol (**4f**): According to Section 3.2.5, **3f** (115 mg, 0.30 mmol, mixture of *E* and *Z*-oximes) was used for the reaction. Eluent (CC): EtOAc/hexane = 20:80. Yield (**4f**): 44 mg (40%, white crystals); Mp: 106–108 °C; Anal. Calcd. for C_20_H_22_F_3_NO_2_ (365.40) C 65.74; H 6.07; N 3.83. Found C 65.60; H 6.41; N 3.75. ^1^H NMR (500 MHz, CDCl_3_): *δ* 0.81 (3H, s, 18-CH_3_), 1.21–1.32 (1H, m), 1.31–1.43 (2H, m), 1.40–1.49 (3H, m), 1.48–1.57 (1H, m), 1.61–1.79 (2H, m), 1.91–1.99 (1H, m), 2.00–2.09 (1H, m), 2.09–2.20 (1H, m), 2.29–2.38 (1H, m), 2.38–2.47 (1H, m), 2.99–3.11 (2H, m), 3.73–3.80 (1H, t, *J* 8.6, 17-αH), 7.39 (1H, s, 4-H), 7.66 (1H, s, 1-H); ^13^C NMR (125 MHz, CDCl_3_) *δ* 11.1 (18-CH_3_), 23.4 (CH_2_), 26.4 (CH_2_), 26.8 (CH_2_), 30.4 (CH_2_), 30.7 (CH_2_), 36.6 (CH_2_), 38.4 (8-CH), 43.3 (13-C), 44.2 (9-CH), 50.5 (14-CH), 81.9 (17-CH), 109.4 (4-CH), 115.7 (2-C), 116.8 (1-CH), 120.5 (1C, q, *J* 271.3, CF_3_), 139.6 (C), 142.8 (C), 149.8 (1C, q, *J* 38.2, 3′-C), 163.2 (3-C); ESI-MS: *m*/*z* 368.3 [M+3H]^+^, 366.2 calcd. for [C_20_H_23_F_3_NO_2_]^+^.

3′-Carboxymethylisoxazolo[4′,5′:2,3]estra-1,3,5(10)-triene-17β-acetate (**4g**): According to Section 3.2.5, **3g** (700 mg, 1.68 mmol) was used for the reaction. Eluent (CC): EtOAc/hexane = 20:80. Yield (**4g**): 601 mg (90%, yellowish white crystals); Mp: 176–178 °C; Anal. Calcd. for C_23_H_27_NO_5_ (397.47) C 69.50; H 6.85; N 3.52. Found C 69.28; H 7.03; N 3.39. ^1^H NMR (500 MHz, CDCl_3_): *δ* 0.85 (3H, s, 18-CH_3_), 1.27–1.42 (1H, m), 1.39–1.52 (4H, overlapping m), 1.50–1.71 (2H, m), 1.73–1.83 (1H, m), 1.90–2.00 (2H, m), 2.07 (3H, s, Ac-CH_3_), 2.20–2.30 (1H, m), 2.30–2.39 (1H, m), 2.40–2.49 (1H, m), 2.98–3.11 (2H, m), 4.08 (3H, s, COOCH_3_), 4.68–4.75 (1H, m, 17-αH), 7.36 (1H, s, 4-H), 7.99 (1H, s, 1-H); ^13^C NMR (125 MHz, CDCl_3_) *δ* 12.2 (18-CH_3_), 21.3 (Ac-CH_3_), 23.5 (CH_2_), 26.4 (CH_2_), 26.9 (CH_2_), 27.7 (CH_2_), 30.4 (CH_2_), 36.8 (CH_2_), 38.2 (8-CH), 43.0 (13-C), 44.1 (9-CH), 50.3 (14-CH), 53.0 (COOCH_3_), 82.7 (17-CH), 109.1 (4-CH), 118.2 (2-C), 118.7 (1-CH), 139.2 (C), 141.8 (C), 150.0 (3′-C), 161.0 (3-C), 163.3 (COOCH_3_), 171.3 (Ac-C=O); ESI-MS: *m*/*z* 398.2 [M + H]^+^, 398.2 calcd. for [C_23_H_28_NO_5_]^+^.

#### 3.2.6. 3-Hydroxy-2-trifluoracetylestra-1,3,5(10)-triene-17-one (**6a**)

To a suspension of E1 (1.08 g, 4.0 mmol) in DCM (30 mL), AlCl_3_ (3.2 g, 6 equiv.) and TFAA (670 µL, 1.2 equiv.) were added at 0 °C. After 30 min, the ice bath was removed, the reaction was gradually warmed to RT and stirred for 4 h. The reaction was poured into ice-cold HCl (1 M, 100 mL) and stirred for 10 min. The layers were separated and the aqueous phase was extracted with EtOAc (3 × 30 mL). The combined organic phase was washed with aq. NaHCO_3_ (10 wt. %, 40 mL), water (40 mL) and brine (40 mL), then dried over anhydrous Na_2_SO_4_ and concentrated in vacuo. The crude product was purified by CC (EtOAc/hexane = 10:90). Yield (**6a**): 1.42 g (97%, yellow crystals); Mp: 179–181 °C; Anal. Calcd. for C_20_H_21_F_3_O_3_ (366.38) C 65.57; H 5.78. Found C 65.46; H 5.97. ^1^H NMR (500 MHz, CDCl_3_): *δ* 0.92 (3H, s, 18-CH_3_), 1.41–1.69 (6H, overlapping m), 1.96–2.11 (3H, m), 2.11–2.21 (1H, m), 2.21–2.33 (1H, m), 2.34–2.42 (1H, m), 2.48–2.57 (1H, m), 2.87–3.03 (2H, m), 6.82 (1H, s, 4-H), 7.69 (1H, s, 1-H), 10.87 (1H, s, 3-OH); ^13^C NMR (125 MHz, CDCl_3_) *δ* 13.9 (18-CH_3_), 21.7 (CH_2_), 25.7 (CH_2_), 26.1 (CH_2_), 30.2 (CH_2_), 31.5 (CH_2_), 35.9 (CH_2_), 38.0 (8-CH), 43.6 (13-C), 48.0 (9-CH), 50.5 (14-CH), 112.3 (2-C), 116.7 (1C, q, *J* 290.0, CF_3_), 118.4 (4-CH), 127.3 (1C, q, *J* 3.9, 1-CH), 132.6 (C), 150.7 (C), 162.5 (3-C), 183.9 (1C, q, *J* 35.0, C=O), 220.3 (17-C=O); ESI-MS: *m*/*z* 365.0 [M − H]^−^, 365.1 calcd. for [C_20_H_20_F_3_O_3_]^−^.

#### 3.2.7. 3-Hydroxy-17-oxoestra-1,3,5(10)-triene-2-carboxylic Acid (**6b**)

To a solution of **6a** (1.35 g, 3.68 mmol) in EtOH (20 mL), KOH (2.06 g, 10 equiv.) in water (3 mL) was added, and the mixture was kept at reflux temperature for 3 h. Then, it was acidified with HCl (6M) and the EtOH was removed under reduced pressure. To the residue HCl (1 M, 10 mL) was added and extracted with EtOAc (3 × 10 mL). The combined organic phase was washed with brine (20 mL), dried over anhydrous Na_2_SO_4_ and concentrated in vacuo. The crude product was purified by CC (EtOAc/DCM = 10:90 with 1% AcOH additive to reduce tailing). Yield (**6b**): 1.09 g (94%, white powder); Mp > 190 °C (decomp.); Anal. Calcd. for C_19_H_22_O_4_ (314.38) C 72.59; H 7.05. Found C 72.62; H 7.24. ^1^H NMR (500 MHz, DMSO-*d*_6_): *δ* 0.82 (3H, s, 18-CH_3_), 1.29–1.52 (5H, overlapping m), 1.52–1.61 (1H, m), 1.73–1.80 (1H, m), 1.88–1.99 (2H, m), 2.01–2.11 (1H, m), 2.12–2.20 (1H, m), 2.26–2.34 (1H, m), 2.39–2.48 (1H, m), 2.77–2.90 (2H, m), 6.66 (1H, s, 4-H), 7.66 (1H, s, 1-H), 11.08 (1H, bs, 3-OH), 13.54 (1H, bs, COOH); ^13^C NMR (125 MHz, DMSO-*d*_6_): *δ* 13.4 (18-CH_3_), 21.1 (CH_2_), 25.4 (CH_2_), 25.6 (CH_2_), 29.0 (CH_2_), 31.2 (CH_2_), 35.3 (CH_2_), 37.4 (8-CH), 42.9 (13-C), 47.2 (9-CH), 49.5 (14-CH), 110.4 (2-C), 116.4 (4-CH), 126.5 (1-CH), 130.9 (C), 145.4 (C), 158.8 (3-C), 171.9 (C=O of carboxyl), 219.5 (C=O of ketone); ESI-MS: *m*/*z* 313.0 [M − H]^−^, 313.1 calcd. for [C_19_H_21_O_4_]^−^.

#### 3.2.8. Three-Step Synthesis of Compound **7**

Synthesis of 3,17β-dihydroxyestra-1,3,5(10)-triene-2-carboxylic acid (**6c**) by the reduction of **6b**: To a solution of **6b** (1.05 g, 3.34 mmol) in EtOH (30 mL), NaBH_4_ (631 mg, 5 equiv.) was added in small portions over a 10 min period and stirring was continued for 30 min at RT. The mixture was neutralized with HCl (6 M) and the EtOH was removed under reduced pressure. To the residue HCl (1 M, 10 mL) was added and extracted with EtOAc (3 × 10 mL). The combined organic phase was washed with brine (20 mL), dried over anhydrous Na_2_SO_4_ and concentrated in vacuo. The crude product was purified by CC (EtOAc/DCM = 20:80 with 1% AcOH additive to reduce tailing). Yield (**6c**): 1.01 g (96%, white powder); Mp > 240 °C (decomp.); Anal. Calcd. for C_19_H_24_O_4_ (316.40) C 72.13; H 7.65. Found C 72.04; H 7.95. ^1^H NMR (500 MHz, DMSO-*d*_6_): *δ* 0.65 (3H, s, 18-CH_3_), 1.02–1.43 (7H, overlapping m), 1.52–1.62 (1H, m), 1.74–1.81 (1H, m), 1.82–1.93 (2H, m), 2.03–2.12 (1H, m), 2.18–2.26 (1H, m), 2.71–2.86 (2H, m), 3.48–3.55 (1H, t, *J* 8.5, 17-αH), 4.29–4.66 (1H, bs, 17-OH), 6.63 (1H, s, 4-H), 7.65 (1H, s, 1-H), 10.97 (1H, bs, 3-OH), 13.64 (1H, bs, COOH); ^13^C NMR (125 MHz, DMSO-*d*_6_): *δ* 11.1 (18-CH_3_), 22.7 (CH_2_), 25.9 (CH_2_), 26.4 (CH_2_), 29.1 (CH_2_), 29.8 (CH_2_), 36.4 (CH_2_), 38.2 (8-CH), 42.7 (13-C), 43.0 (9-CH), 49.5 (14-CH), 80.0 (17-CH), 110.3 (2-C), 116.3 (4-CH), 126.5 (1-CH), 131.4 (C), 145.5 (C), 158.7 (3-C), 172.0 (C=O of carboxyl); ESI-MS: *m*/*z* 315.0 [M − H]^−^, 315.2 calcd. for [C_19_H_23_O_4_]^−^.

Synthesis of 3,17β-dihydroxyestra-1,3,5(10)-triene-2-carboxylic acid methyl ester (**6d**) by esterification of **6c**: To a solution of **6c** (950 mg, 3.00 mmol) in DMF (10 mL), Na_2_CO_3_ (382 mg, 1.2 equiv.) and MeI (280 µL, 1.5 equiv.) were added and the mixture was stirred at 50 °C for 2 h. Then, it was repeatedly concentrated under vacuum with the addition of toluene, and then water (10 mL) was added and extracted with EtOAc (3 × 10 mL). The combined organic phase was washed with aq. NaHCO_3_ (10 wt. %, 2 × 10 mL), water (10 mL) and brine (10 mL), and then dried over anhydrous Na_2_SO_4_ and concentrated *in vacuo*. The crude product was purified by CC (EtOAc/DCM = 5:95). Yield (**6d**): 912 mg (92%, white crystals); Mp: 154–156 °C; Anal. Calcd. for C_20_H_26_O_4_ (330.42) C 72.70; H 7.93. Found C 72.46; H 8.27. ^1^H NMR (500 MHz, CDCl_3_): *δ* 0.78 (3H, s, 18-CH_3_), 1.14–1.24 (1H, m), 1.24–1.58 (7H, overlapping m), 1.64–1.75 (1H, m), 1.84–1.92 (1H, m), 1.93–2.01 (1H, m), 2.09–2.19 (2H, m), 2.31–2.39 (1H, m), 2.82–2.92 (2H, m), 3.70–3.77 (1H, t, *J* 8.5, 17-αH), 3.92 (3H, s, COOCH_3_), 6.70 (1H, s, 4-H), 7.73 (1H, s, 1-H), 10.48 (1H, s, 3-OH); ^13^C NMR (125 MHz, CDCl_3_) *δ* 11.2 (18-CH_3_), 23.3 (CH_2_), 26.4 (CH_2_), 27.0 (CH_2_), 30.0 (CH_2_), 30.7 (CH_2_), 36.7 (CH_2_), 38.8 (8-CH), 43.4 (13-C), 43.8 (9-CH), 50.2 (14-CH), 52.2 (COOCH_3_), 82.0 (17-CH), 110.1 (2-C), 117.1 (4-CH), 126.6 (1-CH), 132.0 (C), 146.3 (C), 159.3 (3-C), 170.8 (COOCH_3_); ESI-MS: *m*/*z* 331.2 [M + H]^+^, 331.2 calcd. for [C_20_H_27_O_4_]^+^.

Synthesis of 2-hydroxycarbamoylestra-1,3,5(10)-triene-3,17β-diol (**7**) by hydroxamation of **6d**: To a solution of **6d** (898 mg, 2.72 mmol) in MeOH/THF = 2:1 (27 mL), aq. NH_2_OH (50 wt. %, 2.50 mL, 15 equiv.) and KOH (1.15 g, 7.5 equiv.) were added at 0 °C and the mixture was stirred at RT for 30 min. Then, it was poured into ice-cold HCl (2 M, 50 mL), and the precipitate was filtered off and dried. The crude product was used in the next step without further purification. Yield (**7**): 874 mg (97%, pale tan powder); Mp > 200 °C (decomp.); Anal. Calcd. for C_19_H_25_NO_4_ (331.41) C 68.86; H 7.60; N 4.23. Found C 68.98; H 7.87; N 4.04. ^1^H NMR (500 MHz, DMSO-*d*_6_): *δ* 0.68 (3H, s, 18-CH_3_), 1.04–1.14 (1H, m), 1.12–1.44 (6H, overlapping m), 1.53–1.63 (1H, m), 1.74–1.82 (1H, m), 1.83–1.94 (2H, m), 2.03–2.11 (1H, m, 2.33–2.41 (1H, m), 2.72–2.79 (2H, m), 3.49–3.57 (1H, td, *J* 8.6, 4.7, 17-αH), 4.39–4.44 (1H, d, *J* 4.8, 17-OH), 6.57 (1H, s, 4-H), 7.57 (1H, s, 1-H), 9.14 (1H, s, NOH), 11.35 (1H, s, NH), 12.03 (1H, s, 3-OH); ^13^C NMR (125 MHz, DMSO-*d*_6_): *δ* 11.1 (18-CH_3_), 22.6 (CH_2_), 25.8 (CH_2_), 26.4 (CH_2_), 28.9 (CH_2_), 29.8 (CH_2_), 36.3 (CH_2_), 38.3 (8-CH), 42.7 (13-C), 43.3 (9-CH), 49.5 (14-CH), 79.9 (17-CH), 111.0 (2-C), 116.4 (4-CH), 123.3 (1-CH), 130.9 (C), 142.6 (C), 157.2 (3-C), 166.7 (C=O); ESI-MS: *m*/*z* 332.2 [M + H]^+^, 332.2 calcd. for [C_19_H_26_NO_4_]^+^.

#### 3.2.9. 3′-Hydroxyisoxazolo[4′,5′:2,3]estra-1,3,5(10)-triene-17β-ol (**4h**)

DIAD (615 µL, 3.12 mmol) was added to a solution of PPh_3_ (820 mg, 3.12 mmol) in THF (25 mL), followed by the addition of **7** (828 mg, 2.5 mmol) and the mixture was stirred at RT for 2 h. Then, it was concentrated under reduced pressure and purified by CC (isopropanol/DCM = 2:98 to 5:95). Yield (**4h**): 697 mg (89%, white powder); Mp > 220 °C (decomp.); Anal. Calcd. for C_19_H_23_NO_3_ (313.40) C 72.82; H 7.40; N 4.47. Found C 72.99; H 7.72; N 4.18. ^1^H NMR (500 MHz, DMSO-*d*_6_): *δ* 0.67 (3H, s, 18-CH_3_), 1.05–1.45 (7H, overlapping m), 1.54–1.64 (1H, m), 1.75–1.83 (1H, m), 1.82–1.94 (2H, m), 2.12–2.28 (2H, m), 2.77–2.83 (2H, m), 3.49–3.56 (1H, t, *J* 8.5, 17-αH), 4.49 (1H, bs, 17-OH), 6.92 (1H, s), 6.94 (1H, s), 11.37 (1H, bs, 3′-OH); ^13^C NMR (125 MHz, DMSO-*d*_6_): *δ* 11.2 (18-CH_3_), 22.7 (CH_2_), 26.2 (CH_2_), 26.7 (CH_2_), 29.1 (CH_2_), 29.9 (CH_2_), 36.5 (CH_2_), 38.2 (8-CH), 42.7 (13-C), 43.8 (9-CH), 49.6 (14-CH), 80.0 (17-CH), 106.3 (4-CH), 109.1 (1-CH), 128.2 (C), 130.1 (C), 135.7 (C), 141.6 (C), 154.7 (C); ESI-MS: *m*/*z* 314.2 [M + H]^+^, 314.2 calcd. for [C_19_H_24_NO_3_]^+^.

#### 3.2.10. 3′-Methoxyisoxazolo[4′,5′:2,3]estra-1,3,5(10)-triene-17β-ol (**4i**)

To a solution of 4h (157 mg, 0.50 mmol) in DMF (1 mL), K_2_CO_3_ (207 mg, 3 equiv.) and MeI (47 µL, 1.5 equiv.) were added, and the mixture was stirred at RT for 1 h. Then, it was repeatedly concentrated under reduced pressure with the addition of toluene, and then water (10 mL) was added to the residue and extracted with EtOAc (3 × 10 mL). The combined organic phase was washed with aq. NaHCO_3_ (10 wt. %, 2 × 10 mL), water (10 mL) and brine (10 mL), and then dried over anhydrous Na_2_SO_4_ and concentrated in vacuo. The crude product was purified by CC (EtOAc/DCM = 10:90). Yield (4i): 134 mg (82%, white crystals); Mp: 220–222 °C; Anal. Calcd. for C_20_H_25_NO_3_ (327.42) C 73.37; H 7.70; N 4.28. Found C 73.65; H 7.89; N 4.11. ^1^H NMR (500 MHz, CDCl_3_): δ 0.80 (3H, s, 18-CH_3_), 1.17–1.63 (8H, overlapping m), 1.67–1.77 (1H, m), 1.86–1.94 (1H, m), 1.96–2.03 (1H, m), 2.08–2.19 (1H, m), 2.22–2.38 (2H, m), 2.82–2.96 (2H, m), 3.37 (3H, s, 3′-OMe), 3.75 (1H, t, J 8.5, 17-αH), 6.87 (1H, s, 4-H), 6.90 (1H, s, 1-H); ^13^C NMR (125 MHz, CDCl_3_) δ 11.2 (18-CH_3_), 23.3 (CH_2_), 26.9 (CH_2_), 27.2 (CH_2_), 28.2 (3′-OMe), 29.8 (CH_2_), 30.8 (CH_2_), 36.8 (CH_2_), 38.7 (8-CH), 43.3 (13-C), 44.5 (9-CH), 50.3 (14-CH), 82.0 (17-CH), 105.1 (4-CH), 110.1 (1-CH), 129.9 (C), 131.5 (C), 136.4 (C), 141.1 (C), 155.3 (C); ESI-MS: m/z 328.2 [M + H]^+^, 328.2 calcd. for [C_20_H_26_NO_3_]^+^.

#### 3.2.11. 3′-Hydroxymethylisoxazolo[4′,5′:2,3]estra-1,3,5(10)-triene-17β-acetate (**4j**)

To a solution of **4g** (397 mg, 1.0 mmol) in EtOH (10 mL), NaBH_4_ (151 mg, 4 equiv.) was added in small portions and stirred at RT for 1 h. The solution was acidified with HCl (6 M) and EtOH was evaporated. The residue was suspended in water (10 mL), extracted with EtOAc (3 × 10 mL). The combined organic phase was washed with water (10 mL), brine (10 mL), dried over anhydrous Na_2_SO_4_ and concentrated in vacuo. The crude product was purified by CC (MeOH/DCM = 2:98). Yield (**4j**): 327 mg (89%, greenish white crystals); Mp: 114–116 °C; Anal. Calcd. for C_22_H_27_NO_4_ (369.46) C 71.52; H 7.37; N 3.79. Found C 71.43; H 7.69; N 3.46. ^1^H NMR (500 MHz, CDCl_3_): *δ* 0.84 (3H, s, 18-CH_3_), 1.22–1.68 (7H, overlapping m), 1.72–1.82 (1H, m), 1.89–1.97 (2H, m), 2.07 (3H, s, Ac-CH_3_), 2.11–2.18 (1H, t, *J* 6.3, CH_2_-OH), 2.18–2.29 (1H, m), 2.29–2.44 (2H, m), 2.96–3.08 (2H, m), 4.67–4.74 (1H, m, 17-αH), 5.06 (2H, d, *J* 6.2, CH_2_-OH), 7.28 (1H, s, 4-H), 7.67 (1H, d, *J* 1.4, 1-H); ^13^C NMR (125 MHz, CDCl_3_) *δ* 12.2 (18-CH_3_), 21.3 (Ac-CH_3_), 23.5 (CH_2_), 26.4 (CH_2_), 27.0 (CH_2_), 27.7 (CH_2_), 30.4 (CH_2_), 36.9 (CH_2_), 38.3 (8-CH), 43.0 (13-C), 44.1 (9-CH), 50.3 (14-CH), 57.1 (CH_2_-OH), 82.7 (17-H), 109.0 (4-CH), 117.5 (1-CH), 118.8 (2-C), 137.4 (10-C), 141.0 (5-C), 157.6 (3′-C), 162.3 (3-C), 171.4 (Ac-C=O); ESI-MS: *m*/*z* 370.2 [M + H]^+^, 370.2 calcd. for [C_22_H_28_NO_4_]^+^.

#### 3.2.12. 3′-Hydroxymethylisoxazolo[4′,5′:2,3]estra-1,3,5(10)-triene-17β-ol (**4k**)

To a solution of **4j** (74 mg, 0.20 mmol) in MeOH/DCM = 1:9 (2 mL), NaOH (24 mg, 3 equiv.) was added and the mixture was stirred at RT for 1 h. Then, it was concentrated under reduced pressure and the crude product was purified by CC (MeOH/DCM = 2:98). Yield (**4k**): 62 mg (95%, white crystals); Mp: 218–220 °C; Anal. Calcd. for C_20_H_25_NO_3_ (327.42) C 73.37; H 7.70; N 4.28. Found C 73.20; H 8.08; N 4.09. ^1^H NMR (500 MHz, DMSO-*d*_6_): *δ* 0.68 (3H, s, 18-CH_3_), 1.12–1.44 (6H, overlapping m), 1.44–1.57 (1H, m), 1.58–1.66 (1H, m), 1.78–1.96 (3H, m), 2.22–2.30 (1H, m), 2.33–2.41 (1H, m), 2.90–3.03 (2H, m), 3.55 (1H, td, *J* 8.5, 4.5, 17-αH), 4.50 (1H, d, *J* 4.7, 17-OH), 4.82 (2H, d, *J* 4.5, CH_2_-OH), 5.65–5.71 (1H, m, CH_2_-OH), 7.38 (1H, s, 4-H), 7.82 (1H, s, 1-H); ^13^C NMR (125 MHz, DMSO-*d*_6_): *δ* 11.1 (18-CH_3_), 22.8 (CH_2_), 26.1 (CH_2_), 26.4 (CH_2_), 29.5 (CH_2_), 29.9 (CH_2_), 36.3 (CH_2_), 38.1 (8-CH), 42.7 (13-C), 43.6 (9-CH), 49.8 (14-CH), 54.7 (CH_2_-OH), 79.9 (17-CH), 108.2 (4-CH), 118.1 (1-CH), 118.9 (2-C), 136.8 (10-C), 140.5 (5-C), 158.4 (3′-C), 161.0 (3-C); ESI-MS: *m*/*z* 328.2 [M + H]^+^, 328.2 calcd. for [C_20_H_26_NO_3_]^+^.

#### 3.2.13. 3′-Fluoromethylisoxazolo[4′,5′:2,3]estra-1,3,5(10)-triene-17β-acetate (**4l**)

To a solution of **4j** (150 mg, 0.41 mmol) in DCM (2.5 mL), DAST (85 µL, 1.6 equiv.) was added under a nitrogen atmosphere and stirred at RT for 1 h. The reaction was quenched with the addition of aq. NaHCO_3_ (10 wt. %, 10 mL) and extracted with DCM (3 × 10 mL). The combined organic phase was washed with water (10 mL), brine (10 mL), dried over anhydrous Na_2_SO_4_ and concentrated in vacuo. The crude product was purified by CC (EtOAc/hexane = 10:90). Yield (**4l**): 89 mg (59%, white crystals); Mp: 141–143 °C; Anal. Calcd. for C_22_H_26_FNO_3_ (371.45) C 71.14; H 7.06; N 3.77. Found C 71.01; H 7.43; N 3.68. ^1^H NMR (500 MHz, CDCl_3_): *δ* 0.85 (3H, s, 18-CH_3_), 1.24–1.69 (7H, overlapping m), 1.73–1.83 (1H, m), 1.90–2.00 (2H, m), 2.07 (3H, s, Ac-CH_3_), 2.18–2.30 (1H, m), 2.30–2.44 (2H, m), 2.97–3.09 (2H, m), 4.68–4.75 (1H, m, 17-αH), 5.66–5.84 (2H, d, *J* 47.0, CH_2_F), 7.32 (1H, s, 4-H), 7.68 (1H, s, 1-H); ^13^C NMR (125 MHz, CDCl_3_) *δ* 12.2 (18-CH_3_), 21.3 (Ac-CH_3_), 23.5 (CH_2_), 26.4 (CH_2_), 27.0 (CH_2_), 27.7 (CH_2_), 30.4 (CH_2_), 36.9 (CH_2_), 38.3 (8-CH), 43.0 (13-C), 44.1 (9-CH), 50.3 (14-CH), 76.2 (1C, d, *J* 166.7, CH_2_F), 82.7 (17-CH), 109.1 (4-CH), 117.4 (1-CH), 118.7 (2-C), 137.9 (C), 141.4 (C), 154.2 (1C, d, *J* 22.9, 3′-C), 162.4 (3-C), 171.4 (Ac-C=O); ESI-MS: *m*/*z* 372.2 [M + H]^+^, 372.2 calcd. for [C_22_H_27_FNO_3_]^+^.

#### 3.2.14. 3′-Fluoromethylisoxazolo[4′,5′:2,3]estra-1,3,5(10)-triene-17β-ol (**4m**)

To a solution of **4l** (62 mg, 0.17 mmol) in THF (1.7 mL), LiOH (1 M, 510 µL, 3 equiv.) was added and the mixture was stirred at RT overnight. Then, it was concentrated under reduced pressure and the crude product was purified by CC (EtOAc/DCM = 5:95). Yield (**4m**): 52 mg (95%, white crystals); Mp: 184–186 °C; Anal. Calcd. for C_20_H_24_FNO_2_ (329.42) C 72.92; H 7.34; N 4.25. Found C 72.98; H 7.46; N 4.03. ^1^H NMR (500 MHz, CDCl_3_): *δ* 0.80 (3H, s, 18-CH_3_), 1.18–1.31 (1H, m), 1.31–1.58 (6H, overlapping m), 1.61–1.79 (2H, m), 1.90–1.99 (1H, m), 1.99–2.06 (1H, m), 2.09–2.20 (1H, m), 2.28–2.37 (1H, m), 2.39–2.47 (1H, m), 2.97–3.09 (2H, m), 3.72–3.79 (1H, t, *J* 8.5, 17-αH), 5.69–5.84 (2H, d, *J* 47.0, CH_2_F), 7.31 (1H, s, 4-H), 7.69 (1H, s, 1-H); ^13^C NMR (125 MHz, CDCl_3_) *δ* 11.2 (18-CH_3_), 23.4 (CH_2_), 26.5 (CH_2_), 27.0 (CH_2_), 30.4 (CH_2_), 30.7 (CH_2_), 36.7 (CH_2_), 38.6 (8-CH), 43.3 (13-C), 44.2 (9-CH), 50.5 (14-CH), 76.2 (1C, d, *J* 166.8, CH_2_F) 81.9 (17-CH), 109.1 (4-CH), 117.4 (1-CH), 118.7 (2-C), 138.1 (C), 141.5 (C), 154.2 (1C, d, *J* 22.6, 3′-C), 162.4 (3-C). ESI-MS: *m*/*z* 330.2 [M + H]^+^, 330.2 calcd. for [C_20_H_25_FNO_2_]^+^.

#### 3.2.15. 3′-Formylisoxazolo[4′,5′:2,3]estra-1,3,5(10)-triene-17β-acetate (**4n**)

To a solution of **4j** (148 mg, 0.40 mmol) in DCM (5 mL), Dess–Martin periodinane (255 mg, 1.5 equiv.) was added under a nitrogen atmosphere and the mixture was stirred at RT for 2 h. The reaction was filtered into a separating funnel through cotton wool, and then water was added (10 mL) and extracted with DCM (3 × 10 mL). The combined organic phase was washed with aq. NaHCO_3_ (10 wt. %, 10 mL), water (10 mL) and brine (10 mL), and then dried over anhydrous Na_2_SO_4_ and concentrated in vacuo. The crude product was purified by CC (hexane/DCM = 20:80). Yield (**4n**): 119 mg (81%, yellowish white crystals); Mp: 159–160 °C; Anal. Calcd. for C_22_H_25_NO_4_ (367.44) C 71.91; H 6.86; N 3.81. Found C 71.74; H 7.15; N 3.59. ^1^H NMR (500 MHz, CDCl_3_): *δ* 0.85 (3H, s, 18-CH_3_), 1.24–1.70 (7H, overlapping m), 1.72–1.82 (1H, m), 1.90–2.00 (2H, m), 2.07 (3H, s, Ac-CH_3_), 2.19–2.30 (1H, m), 2.30–2.40 (1H, m), 2.40–2.49 (1H, m), 2.98–3.10 (2H, m), 4.67–4.74 (1H, m, 17-αH), 7.39 (1H, s 4-H), 8.05 (1H, s, 1-H), 10.39 (1H, s, CHO); ^13^C NMR (125 MHz, CDCl_3_) *δ* 12.2 (18-CH_3_), 21.3 (Ac-CH_3_), 23.5 (CH_2_), 26.3 (CH_2_), 26.8 (CH_2_), 27.7 (CH_2_), 30.4 (CH_2_), 36.8 (CH_2_), 38.2 (8-CH), 43.0 (13-C), 44.1 (9-CH), 50.3 (14-CH), 82.7 (17-CH), 109.0 (4-CH), 116.3 (2-C), 118.8 (1-CH), 139.8 (C), 142.1 (C), 155.5 (C), 163.4 (C), 171.4 (Ac-C=O), 186.0 (CHO); ESI-MS: *m*/*z* 368.2 [M + H]^+^, 368.2 calcd. for [C_22_H_26_NO_4_]^+^.

#### 3.2.16. 3′-Difluoromethylisoxazolo[4′,5′:2,3]estra-1,3,5(10)-triene-17β-acetate (**4o**)

To a solution of **4n** (62 mg, 0.17 mmol) in DCM (2.0 mL), DAST (90 µL, 4.0 equiv.) was added at 0 °C under a nitrogen atmosphere and stirred at RT for 2 h. The reaction was quenched with the addition of aq. NaHCO_3_ (10 wt. %, 10 mL) and extracted with DCM (3 × 10 mL). The combined organic phase was washed with water (10 mL), brine (10 mL), dried over anhydrous Na_2_SO_4_ and concentrated in vacuo. The crude product was purified by CC (EtOAc/hexane = 3:97). Yield (**4o**): 61 mg (92%, white crystals); Mp: 137–139 °C; Anal. Calcd. for C_22_H_25_F_2_NO_3_ (389.44) C 67.85; H 6.47; N 3.60. Found C 67.55; H 6.80; N 3.49. ^1^H NMR (500 MHz, CDCl_3_): *δ* 0.86 (3H, s, 18-CH_3_), 1.27–1.71 (7H, overlapping m), 1.72–1.83 (1 H, m), 1.91–2.00 (2H, m), 2.07 (3H, s, Ac-CH_3_), 2.19–2.30 (1H, m), 2.30–2.37 (1H, m), 2.37–2.45 (1H, m), 3.01–3.08 (2H, m), 4.67–4.76 (1H, t, *J* 8.4, 17-αH), 6.83–7.17 (1H, t, *J* 53.5, CHF_2_), 7.36 (1H, s, 4-H), 7.74 (1H, s, 1-H); ^13^C NMR (125 MHz, CDCl_3_) *δ* 12.2 (18-CH_3_), 21.3 (Ac-CH_3_), 23.5 (CH_2_), 26.3 (CH_2_), 26.9 (CH_2_), 27.8 (CH_2_), 30.4 (CH_2_), 36.9 (CH_2_), 38.3 (8-CH), 43.0 (13-C), 44.1 (9-CH), 50.4 (14-CH), 82.7 (17-H), 109.2 (4-CH), 110.5 (1C, t, *J* 236.5, CHF_2_), 116.1 (2-C), 117.5 (1-CH), 138.8 (C), 142.2 (C), 153.0 (1C, t, *J* 30.6, C=N), 162.9 (3-C), 171.3 (Ac-C=O); ESI-MS: *m*/*z* 390.2 [M + H]^+^, 390.2 calcd. for [C_22_H_26_F_2_NO_3_]^+^.

#### 3.2.17. 3′-Difluoromethylisoxazolo[4′,5′:2,3]estra-1,3,5(10)-triene-17β-ol (**4p**)

To a solution of **4o** (47 mg, 0.12 mmol) in THF (1.2 mL), LiOH (1 M, 360 µL, 3 equiv.) was added and the mixture was stirred at RT overnight. Then, it was concentrated under reduced pressure and the crude product was purified by CC (DCM). Yield (**4p**): 36 mg (86%, white crystals); Mp: 148–150 °C; Anal. Calcd. for C_20_H_23_F_2_NO_2_ (347.41) C 69.15; H 6.67; N 4.03. Found C 69.10; H 6.84; N 3.88. ^1^H NMR (500 MHz, CDCl_3_): *δ* 0.81 (3H, s, 18-CH_3_), 1.20–1.57 (7H, overlapping m), 1.62–1.79 (2H, m), 1.90–1.99 (1H, m), 1.99–2.07 (1H, m), 2.09–2.20 (1H, m), 2.28–2.37 (1H, m), 2.39–2.48 (1H, m), 2.98–3.08 (2H, m), 3.73–3.80 (1H, t, *J* 8.5, 17-αH), 6.82–7.14 (1H, t, *J* 53.5, CHF_2_), 7.35 (1H, s, 4-CH), 7.75 (1H, s, 1-CH); ^13^C NMR (125 MHz, CDCl_3_) *δ* 11.2 (18-CH_3_), 23.4 (CH_2_), 26.5 (CH_2_), 26.9 (CH_2_), 30.5 (CH_2_), 30.8 (CH_2_), 36.7 (CH_2_), 38.6 (8-CH), 43.4 (13-C), 44.3 (9-CH), 50.6 (14-CH), 81.9 (17-CH), 109.2 (4-CH), 110.5 (1C, t, *J* 236.5, CHF_2_), 116.1 (2-C), 117.5 (1-CH), 138.9 (C), 142.2 (C), 153.0 (1C, t, *J* 30.8, 3′-C), 162.9 (3-C); ESI-MS: *m*/*z* 348.2 [M + H]^+^, 348.2 calcd. for [C_20_H_24_F_2_NO_2_]^+^.

#### 3.2.18. 17β-Hydroxy-isoxazolo[4′,5′:2,3]estra-1,3,5(10)-triene-3′-carboxylic Acid (**4q**)

To a solution of **4g** (180 mg, 0.45 mmol) in MeOH/DCM = 1:9 (4.5 mL), NaOH (108 mg, 6 equiv.) was added and the mixture was stirred at RT for 1 h. Then, it was concentrated under reduced pressure, redissolved in MeOH and poured into ice-cold HCl (2M, 20 mL). The precipitate was filtered off and dried under vacuum. Yield (**4q**): 144 mg (94%, white powder); Mp > 150 °C (decomp.); Anal. Calcd. for C_20_H_23_NO_4_ (341.41) C 70.36; H 6.79; N 4.10. Found C 70.53; H 7.01; N 3.84. ^1^H NMR (500 MHz, DMSO-*d*_6_): *δ* 0.65 (3H, s, 18-CH_3_), 1.03–1.42 (7H, overlapping m), 1.52–1.62 (1H, m), 1.72–1.79 (1H, m), 1.79–1.86 (1H, m), 1.83–1.93 (1H, m), 1.99–2.08 (1H, m), 2.21–2.29 (1H, m), 2.72–2.78 (2H, m), 3.47–3.54 (1H, t, *J* 8.5, 17-αH), 4.49 (1H, bs, 17-OH), 6.66 (1H, s, 4-H), 7.36 (1H, s, 1-H), 10.99 (1H, bs, COOH); ^13^C NMR (125 MHz, DMSO-*d*_6_): *δ* 11.2 (18-CH_3_), 22.7 (CH_2_), 25.7 (CH_2_), 26.4 (CH_2_), 29.3 (CH_2_), 29.8 (CH_2_), 36.4 (CH_2_), 38.1 (8-CH), 42.7 (13-C), 43.0 (9-CH), 49.4 (14-CH), 80.0 (17-CH), 96.3 (C), 115.9 (4-CH), 117.8 (C), 124.2 (C), 129.5 (1-CH), 131.4 (C), 144.1 (C), 158.4 (C); ESI-MS: *m*/*z* 342.2 [M + H]^+^, 342.2 calcd. for [C_20_H_24_NO_4_]^+^.

#### 3.2.19. 17β-Hydroxy-isoxazolo[4′,5′:2,3]estra-1,3,5(10)-triene-3′-carboxylic Acid Methyl Ester (**4r**)

To a solution of **4q** (100 mg, 0.29 mmol) in MeOH (3 mL), cc. H_2_SO_4_ (1 drop) was added and kept at reflux temperature for 36 h. Then, it was neutralized with NaHCO_3_ and concentrated in vacuo. The crude product was purified by CC (EtOAc/DCM = 10:90). Yield (**4r**): 94 mg (90%, white powder); Mp: 201–203 °C; Anal. Calcd. for C_21_H_25_NO_4_ (355.43) C 70.96; H 7.09; N 3.94. Found C 70.73; H 7.34; N 3.65. ^1^H NMR (500 MHz, CDCl_3_): *δ* 0.80 (3H, s, 18-CH_3_), 1.21–1.58 (7H, overlapping m), 1.62–1.79 (2H, m), 1.90–1.99 (1H, m), 1.99–2.06 (1H, m), 2.09–2.23 (1H, m), 2.29–2.38 (1H, m), 2.43–2.52 (1H, m), 2.98–3.10 (2H, m), 3.72–3.80 (1H, td, *J* 8.5, 3.3, 17-αH), 4.08 (3H, s, COOCH_3_), 7.36 (1H, s, 4-CH), 7.99 (1H, s, 1-H); ^13^C NMR (125 MHz, CDCl_3_) *δ* 11.2 (18-CH_3_), 23.4 (CH_2_), 26.5 (CH_2_), 26.9 (CH_2_), 30.4 (CH_2_), 30.7 (CH_2_), 36.7 (CH_2_), 38.5 (8-CH), 43.3 (13-C), 44.3 (9-CH), 50.5 (14-CH), 53.0 (COOCH_3_), 81.9 (17-CH), 109.1 (4-CH), 118.2 (2-C), 118.7 (1-CH), 139.3 (C), 141.8 (C), 150.0 (C), 161.0 (C), 163.3 (COOCH_3_); ESI-MS: *m*/*z* 356.2 [M + H]^+^, 356.2 calcd. for [C_21_H_26_NO_4_]^+^.

#### 3.2.20. 17β-Hydroxy-isoxazolo[4′,5′:2,3]estra-1,3,5(10)-triene-3′-carboxamide (**4s**)

To a solution of **4r** (62 mg, 0.17 mmol) in NH_3_ (6 M in MeOH, 2 mL), cat. KCN was added and the mixture was stirred at RT for 4 h, and then it was concentrated in vacuo. The crude product was purified by CC (EtOAc/DCM = 50:50). Yield (**4s**): 56 mg (94%, white crystals); Mp: 250–252 °C; Anal. Calcd. for C_20_H_24_N_2_O_3_ (340.42) C 70.57; H 7.11; N 8.23. Found C 70.37; H 7.44; N 8.06. ^1^H NMR (500 MHz, DMSO-*d*_6_): *δ* 0.67 (3H, s, 18-CH_3_), 1.11–1.44 (6H, overlapping m), 1.47–1.66 (2H, m), 1.78–1.95 (3H, m), 2.23–2.38 (2H, m), 2.91–3.05 (2H, m), 3.50–3.58 (1H, td, *J* 8.5, 4.8, 17-αH), 4.48–4.53 (1H, d, *J* 4.8, 17-OH), 7.51 (1H, s, 4-CH), 7.94 (1H, s, 1-H), 7.98 (1H, s, one H of NH_2_), 8.29 (1H, s, the other H of NH_2_); ^13^C NMR (125 MHz, DMSO-*d*_6_): *δ* 11.1 (18-CH_3_), 22.8 (CH_2_), 26.0 (CH_2_), 26.2 (CH_2_), 29.5 (CH_2_), 29.8 (CH_2_), 36.3 (CH_2_), 37.9 (8-CH), 42.7 (13-C), 43.4 (9-CH), 49.8 (14-H), 79.9 (17-CH), 108.5 (4-CH), 117.7 (2-C), 118.5 (1-CH), 138.4 (C), 141.3 (C), 151.9 (3′-C), 160.7 (3-C), 161.9 (CONH_2_); ESI-MS: *m*/*z* 341.2 [M + H]^+^, 341.2 calcd. for [C_20_H_25_N_2_O_3_]^+^.

#### 3.2.21. 2-Cyanoestra-1,3,5(10)-triene-3,17β-ol (**8**) by Cyclization Followed by Kemp Elimination from **3a** (Scheme 3, xi)

DIAD (490 µL, 2.50 mmol) was added to a solution of PPh_3_ (656 mg, 2.50 mmol) in ACN (10 mL), followed by the addition of **3a** (315 mg, 1.00 mmol). The reaction mixture was stirred at RT for 1 h, and then it was concentrated under reduced presure. The crude product was purified by CC (EtOAc/hexane = 10:90 to 40:60). Yield (**8**): 258 mg (87%, white powder); Mp > 200 °C (decomp.); Anal. Calcd. for C_19_H_23_NO_2_ (297.40) C 76.74; H 7.80; N 4.71. Found C 76.50; H 8.14; N 4.66. ^1^H NMR (500 MHz, DMSO-*d*_6_): *δ* 0.65 (3H, s, 18-CH_3_), 1.02–1.45 (7H, overlapping m), 1.52–1.62 (1H, m), 1.71–1.93 (3H, m), 1.99–2.10 (1H, m), 2.22–2.30 (1H, m), 2.73–2.80 (2H, m), 3.51 (1 H, t, *J* 8.5, 17-αH), 4.48 (1H, s, 17-OH), 6.67 (1H, s, 4-H), 7.39 (1H, s, 1-H), 10.63 (1H, s, 3-OH); ^13^C NMR (125 MHz, DMSO-*d*_6_): *δ* 11.1 (18-CH_3_), 22.7 (CH_2_), 25.6 (CH_2_), 26.3 (CH_2_), 29.3 (CH_2_), 29.8 (CH_2_), 36.3 (CH_2_), 38.1 (8-CH), 42.7 (13-C), 42.9 (9-CH), 49.4 (14-CH), 79.9 (17-CH), 96.2 (2-C), 115.7 (4-CH), 117.5 (CN), 129.6 (1-CH), 132.0 (C), 144.2 (C), 157.6 (3-C); ESI-MS: *m*/*z* 298.2 [M + H]^+^, 298.2 calcd. for [C_19_H_24_NO_2_]^+^.

#### 3.2.22. Estra-1,3,5(10)-triene-3,17β-diol-2-carboxamidoxime (**9**)

To a solution of 8 (48 mg, 0.16 mmol) in EtOH (2 mL), Na_2_CO_3_ (34 mg, 2 equiv.) and aq. NH_2_OH (50 wt. %, 49 µL, 5 equiv.) were added and the mixture was refluxed overnight. After being concentrated under reduced presure, water (10 mL) was added and extracted with EtOAc (3 × 10 mL). The combined organic phase was washed with water (10 mL) and brine (10 mL), dried over anhydrous Na_2_SO_4_ and concentrated in vacuo. The crude product was purified by CC (EtOAc/DCM = 20:80). Yield (9): 48 mg (90%, white powder); Mp > 210 °C (decomp.); Anal. Calcd. for C_19_H_26_N_2_O_3_ (330.43) C 69.06; H 7.93; N 8.48. Found C 69.13; H 8.15; N 8.29. ^1^H NMR (500 MHz, DMSO-*d*_6_): *δ* 0.67 (3H, s, 18-CH_3_), 1.07–1.44 (8H, overlapping m), 1.54–1.62 (1H, m), 1.75–1.81 (1H, m), 1.83–1.94 (2H, m), 2.04–2.13 (1H, m), 2.70–2.76 (2H, m), 3.49–3.57 (1H, td, *J* 8.3, 4.7, 17-αH), 4.47 (1H, d, *J* 4.8, 17-OH), 6.28 (2H, s, -NH_2_), 6.49 (1H, s, 4-H), 7.47 (1H, s, 1-H), 9.85 (1H, s, -OH), 11.89 (1H, s, -OH); ^13^C NMR (125 MHz, DMSO-*d*_6_): *δ* 11.3 (18-CH_3_), 22.8 (CH_2_), 25.9 (CH_2_), 26.7 (CH_2_), 28.9 (CH_2_), 29.9 (CH_2_), 36.5 (CH_2_), 38.6 (8-CH), 42.8 (13-C), 43.7 (9-CH), 49.6 (14-CH), 80.0 (17-CH), 112.2 (2-C), 115.9 (4-CH), 122.6 (1-CH), 130.2 (C), 138.7 (C), 153.7 (C), 154.9 (C); ESI-MS: *m*/*z* 331.2 [M + H]^+^, 331.2 calcd. for [C_19_H_27_N_2_O_3_]^+^.

#### 3.2.23. 2′-Aminooxazolo[4′,5′:2,3]estra-1,3,5(10)-triene-17β-ol (**10**)

DDQ (52 mg, 0.23 mmol) was added slowly to the solution of PPh_3_ (59 mg, 0.23 mmol) in DCM (1 mL), and the mixture was stirred for 1 min. This suspension was added to a solution of **9** (50 mg, 0.15 mmol) and Et_3_N (45 µL, 0.30 mmol) in DCM (1 mL), and the mixture was stirred at RT for 30 min. After 30 min, another portion of DDQ/PPh_3_ was added and stirred until TLC indicated the complete conversion of the starting material. The solvent was removed under vacuum; the residue was dissolved in EtOAc/MeOH, and then Celite^®^ was added (~10 × weight of the crude sample) and the solvent was removed *in vacuo*. The crude product was purified by CC (EtOAc/hexane = 70:30). Yield (**10**): 33 mg (70%, white powder); Mp > 210 °C (decomp.); Anal. Calcd. for C_19_H_24_N_2_O_2_ (312.41) C 73.05; H 7.74; N 8.97. Found C 73.26; H 7.98; N 8.75. ^1^H NMR (500 MHz, DMSO-*d*_6_): *δ* 0.67 (3H, s, 18-CH_3_), 1.08–1.46 (7H, overlapping m), 1.54–1.64 (1H, m), 1.76–1.82 (1H, m), 1.83–1.94 (2H, m), 2.13–2.22 (1H, m), 2.30 (1H, m), 2.76–2.89 (2H, m, 6-H_2_), 3.53 (1H, td, *J* 8.5, 4.6, 17-αH), 4.48 (1H, d, *J* 4.8, 17-OH), 6.96 (1H, s, 4-H), 7.08 (1H, s, 1-H), 7.15 (2H, s, NH_2_); ^13^C NMR (125 MHz, DMSO-*d*_6_): *δ* 11.2 (18-CH_3_), 22.8 (CH_2_), 26.3 (CH_2_), 27.0 (CH_2_), 29.3 (CH_2_), 29.9 (CH_2_), 36.6 (CH_2_), 38.5 (8-CH), 42.7 (13-C), 44.1 (9-CH), 49.7 (14-CH), 80.0 (17-CH), 107.8 (4-CH), 111.8 (3-CH), 128.3 (5-C), 135.3 (10-C), 141.6 (2-C), 146.2 (3-C), 162.3 (2′-C); ESI-MS: *m*/*z* 313.2 [M + H]^+^, 313.2 calcd. for [C_19_H_25_N_2_O_2_]^+^.

### 3.3. Pharmacology

#### 3.3.1. Cell Culture

All cell lines were obtained from the American Type Culture Collection and maintained in a standard incubator under normal cell culture conditions (37 °C, 5% CO_2_, 95% humidity). DU-145, PC3 (both prostate cancer) HeLa (cervical cancer) and MCF-7 (breast cancer) cell lines were maintained in RPMI-1640 medium (Biosera), whereas MRC-5 (non-cancerous fibroblast) cells were cultured in EMEM medium (Biosera). Both RPMI-1640 and EMEM media were supplemented with 10% fetal bovine serum (FBS), 2 mM glutamine and 1% penicillin-streptomycin.

#### 3.3.2. Cell Viability Assay

For the pharmacological studies, each compound was dissolved in cell culture grade DMSO (Sigma) at a final concentration of 2.5, 5 or 10 mM (depending on solubility). For the initial screening, cells were seeded at 3 × 10^3^ cells/well density in 96-well plates and left to adhere overnight. The next day, cells were treated with each of the steroid derivatives at 2.5 μM concentration for 72 h. Control cells received DMSO treatment (solvent of the steroid derivatives). For IC_50_ measurements, cells were seeded in the same way as above. The next day cells were treated with either **4b**, **4c** or **4d** in 1, 2, 3, 4, 5, 6, 8 and 10 μM concentration for 72 h to obtain the dose-response curves. After 72-h treatments in both cases (initial screening, IC_50_ measurements), MTT assays were conducted. For this, treatment media were replaced with fresh media containing 0.5 mg/mL 3-(4,5-dimethylthiazol-2-yl)-2,5-diphenyl tetrazolium bromide (MTT) reagent (Sigma-Aldrich). Cells were incubated with MTT reagent-containing media for 1–3 h, and then the formed formazan crystals were solubilized in DMSO, and the absorbance of the samples was measured at 570 nm with Synergy HTX microplate reader (BIOTEK^®^). The average viability of the DMSO-treated cells was considered 100%. On the viability data, dose-response curves were fitted (Supplementary Material p52–53), and IC_50_ values were calculated accordingly. The experiments were repeated three times using three biological replicates.

#### 3.3.3. Reverse Transcription Quantitative Polymerase Chain Reaction (RT-qPCR)

For total RNA isolation, cells were seeded in 60 mm dishes at 0.8 × 10^6^ cells/dish density and left to adhere overnight. The next day, cells were treated with either **4b**, **4c** or **4d** in different concentrations for 72 h (**4b** (DU-145: 2 μM HeLa: 1 μM, MCF-7 1 μM), **4c** (DU-145: 1 μM HeLa: 1 μM, MCF-7 1 μM) and **4d** (DU-145: 4 μM HeLa: 2 μM, MCF-7 2 μM)). After the treatments, total RNA was isolated with the RNeasy^®^ Mini Kit (QIAGEN, Hilden, Germany) according to the manufacturer′s recommendation. The concentration and purity of the isolated total RNA were measured with the NanoDrop ND 1000 Spectrophotometer (Thermo Fisher Scientific, Waltham, MA, USA). From each sample, 1 μg RNA was reverse transcribed in a 20 μL reaction with the TaqMan^®^ Reverse Transcription kit (Applied Biosystems, Thermo Fisher Scientific, Waltham, MA, USA) following the manufacturer′s instructions. cDNA was diluted 5X to a final volume of 100 μL. qPCR reactions were performed with PicoReal™ Real-time PCR (Thermo Fisher Scientific, Waltham, MA, USA) using SYBR Green qPCR Master Mix (Thermo Fisher Scientific, Waltham, MA, USA). Reactions were carried out in a 10 μL reaction volume (5 μL SYBR Green, 3 μL RNase-free H_2_O, 1 μL cDNA, 1 μL primer-mix). The sequence and final concentration of the used primers are found within the Supplementary Material p54. Relative transcript levels were determined by the ΔΔCt method, using GAPDH as the reference gene. Experiments were repeated twice with three biological replicates.

#### 3.3.4. Statistical Analysis

Data analysis, IC_50_ value calculations, graphical representation of data (heatmap, dose–response curves and qPCR diagrams) and statistical analysis (non-linear regression, two-way ANOVA) were carried out using GraphPad Prism 8.0.1 software. Differences between the control and treated samples were considered statistically significant, if *p* < 0.05.

## 4. Conclusions

In summary, a compound library containing novel estradiol-benzisoxazole chimeras, differing only in their C-3′ substituent of the heteroring, was created via multistep pathways. The majority of the 19 new heterocyclic compounds were obtained from appropriately synthesized 2-substituted E1 or E2 precursors, and subsequent ring closure involving the 3-OH functionality was performed by high conversion. However, some products were obtained from a 3′-methoxycarbonyl-substituted isoxazole derivative by further modification of its ester group. The steroid products, including all intermediates, were structurally characterized and subjected to in vitro pharmacological studies. The primary screen to test the antiproliferative activity of the obtained compounds provided numerous positive hits, several derivatives exhibited strong anticancer performance, and most estradiol-benzisoxazole hybrids showed remarkable cancer cell selectivity. The three most promising compounds, the 3′-methyl, 3′-ethyl and 3′-isopropyl substituted steroidal benzisoxazoles showed a high degree of cytotoxicity on all tested cancerous cell lines, whereas treatment of non-cancerous cells with these derivatives resulted in no, or minimal change in cell viability. The minimal inhibitory concentrations (IC_50_) of the three compounds were determined on cancerous and non-cancerous cell lines. Interestingly, the IC_50_ values of each molecule were one or two magnitudes lower for the cancerous cell lines compared to the values obtained on non-cancerous fibroblasts. The IC_50_ values of the most potent derivatives were compared to that of cisplatin, a clinically available drug, where we found that unlike cisplatin, estradiol-benzisoxazole hybrids show an outstanding cancer cell specificity. Lastly, we found that each of the three compounds exhibit strong apoptosis inducing potential, which could be the underlying cause of their impressive anticancer performance. Based on our findings, estradiol-benzisoxazole hybridization seems to be exceptionally advantageous for securing excellent anticancer activity; therefore, this structural motif should be considered in rational drug design and future synthesis approaches for clinical cancer therapy. The mechanism of action of the most potent steroidal hybrids as well as their hormone receptor binding will be further investigated.

## Figures and Tables

**Table 1:** IC_50_ values (µM ± SD) of the selected compounds and cisplatin determined on various cancer cell lines and on non-cancerous MRC-5 cells.

Cell Lines	IC_50_ Values (µM ± SD)			
	4b	4c	4d	Cisplatin
MRC-5	27.08 ± 4.06	18.39 ± 2.02	32.36 ± 3.23	32 ± 1.2
DU-145	1.09 ± 0.05	0.56 ± 0.06	2.87 ± 0.2	118 ± 1.1
HeLa	0.46 ± 0.03	0.28 ± 0.03	1.1 ± 0.08	>330
MCF-7	0.34 ± 0.02	0.25 ± 0.03	1.2 ± 0.1	296 ± 1.1

Cisplatin values according to our publication [24].

## Data Availability

Not applicable.

## Supplementary Materials

The following supporting information can be downloaded at: https://www.mdpi.com/article/10.3390/molecules27217456/s1, p2–49: ^1^H NMR and ^13^C NMR spectra of the synthesized compounds, Table in p50: Predicted pharmacokinetic parameters of synthesized compounds, Table in p51: Mean ± SD values of primary growth inhibitory screen (given as cell viability) used to construct the heatmap, Figure in p52–53: Dose response curves used to evaluate IC_50_ concentrations of the selected compounds and Table in p54: List, sequence and final concentrations of primers used in qRT-PCR.

## Sample Availability

Samples of the compounds are not available from the authors.
