# Supplementary material for: Substitutional Diversity-Oriented Synthesis and In Vitro Anticancer Activity of Framework-Integrated Estradiol-Benzisoxazole Chimeras
Source: Molecules. 2022 Nov 2;27(21):7456. doi: 10.3390/molecules27217456 (PMC9654004; doi:10.3390/molecules27217456)
Supplement: Supplementary file 1 [file molecules-27-07456-s001.zip › molecules-1984378-supplementary.pdf]

*Supplementary Material*

# Substitutional Diversity-Oriented Synthesis and In Vitro Anticancer Activity of Framework-Integrated Estradiol-Benzisoxazole Chimeras

**Ferenc Kovács [1], Dóra Izabella Adamecz [2], Ferenc István Nagy [2], Benedek Papp [2], Mónika Kiricsi [2] and Éva Frank [1,*]**

[1] Department of Organic Chemistry, University of Szeged, Dóm tér 8, H-6720 Szeged, Hungary
[2] Department of Biochemistry and Molecular Biology, Doctoral School of Biology, University of Szeged, Közép fasor 52, H-6726 Szeged, Hungary
* Correspondence: frank@chem.u-szeged.hu; Tel.: +36-62-544-275

**Contents**

*$^{1}$H and $^{13}$C NMR spectra of the synthesized compounds* ........ 2-49
*Predicted pharmacokinetic parameters of the synthesized compounds* ........ 50
*Mean values of primary growth inhibitory screen used for heat map construction* ........ 51
*Dose-response curves* ........ 52-53
*List, sequence and appropriate concentrations of primers used in qRT-PCR* ........ 54

*$^{1}$H and $^{13}$C NMR spectra of the synthesized compounds*

$^{1}$H NMR spectrum of compound **1** ($CDCl_3$, 500 MHz)

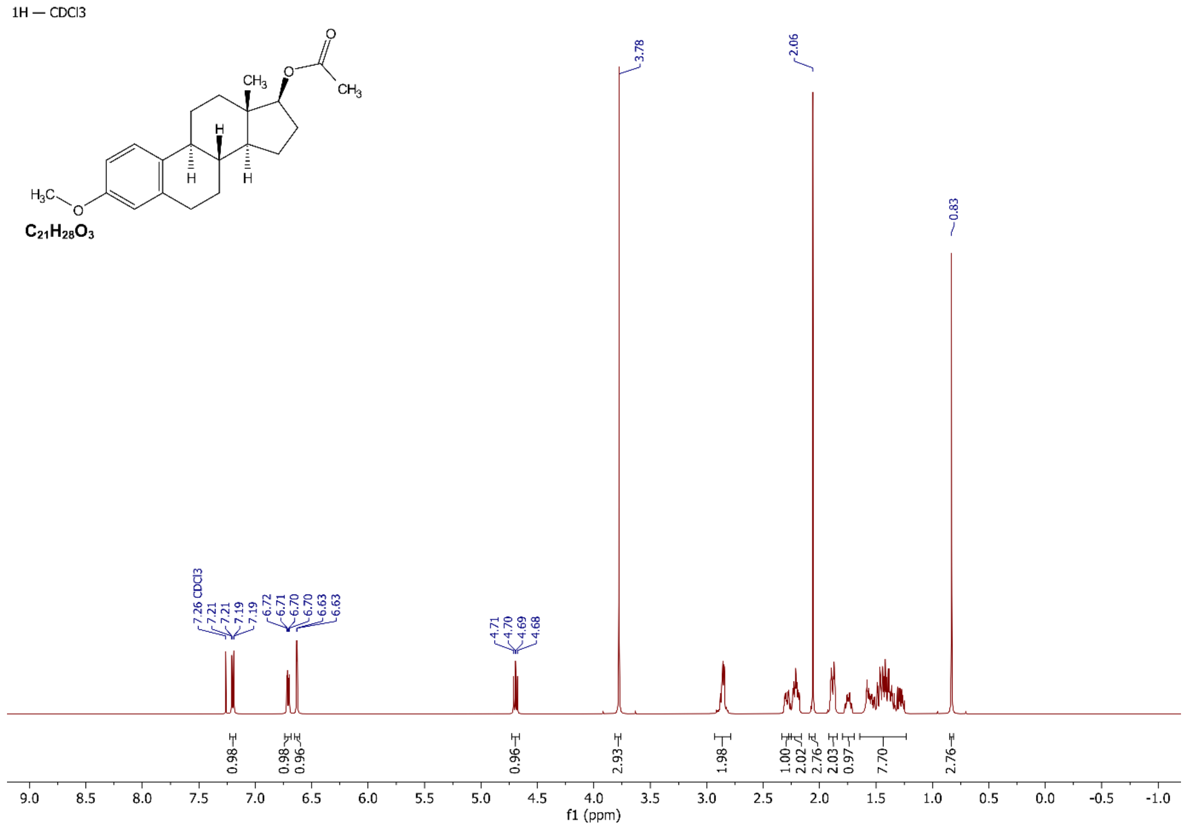


$^{13}$C NMR spectrum of compound **1** ($CDCl_3$, 125 MHz)

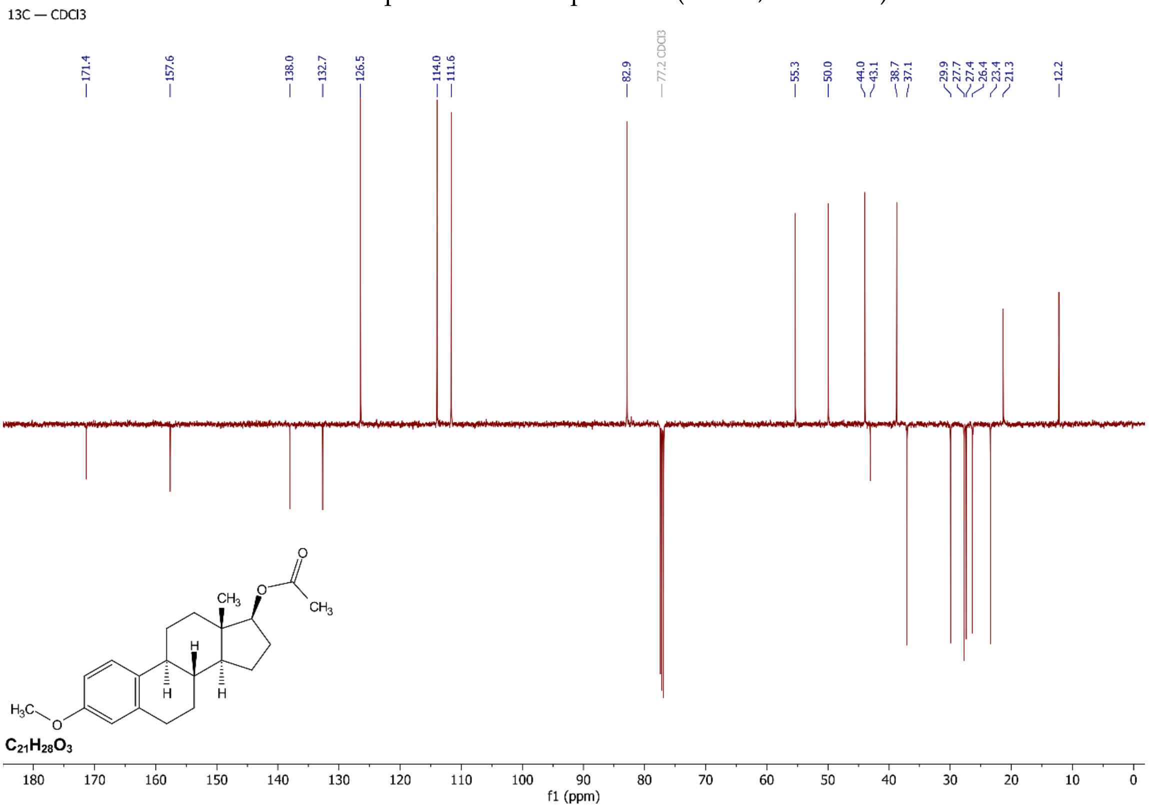

$^{1}H$ NMR spectrum of compound **2a** ($CDCl_3$, 500 MHz)

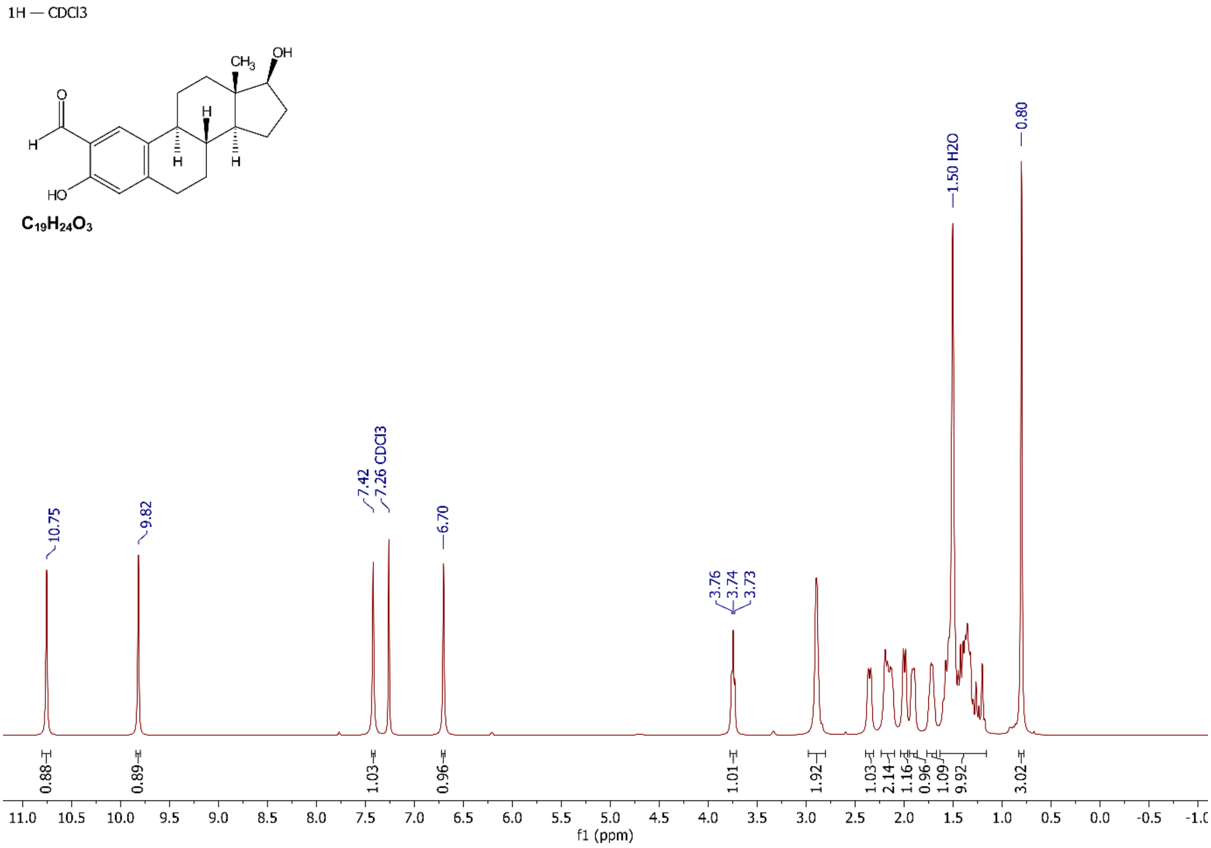


$^{13}C$ NMR spectrum of compound **2a** ($CDCl_3$, 125 MHz)

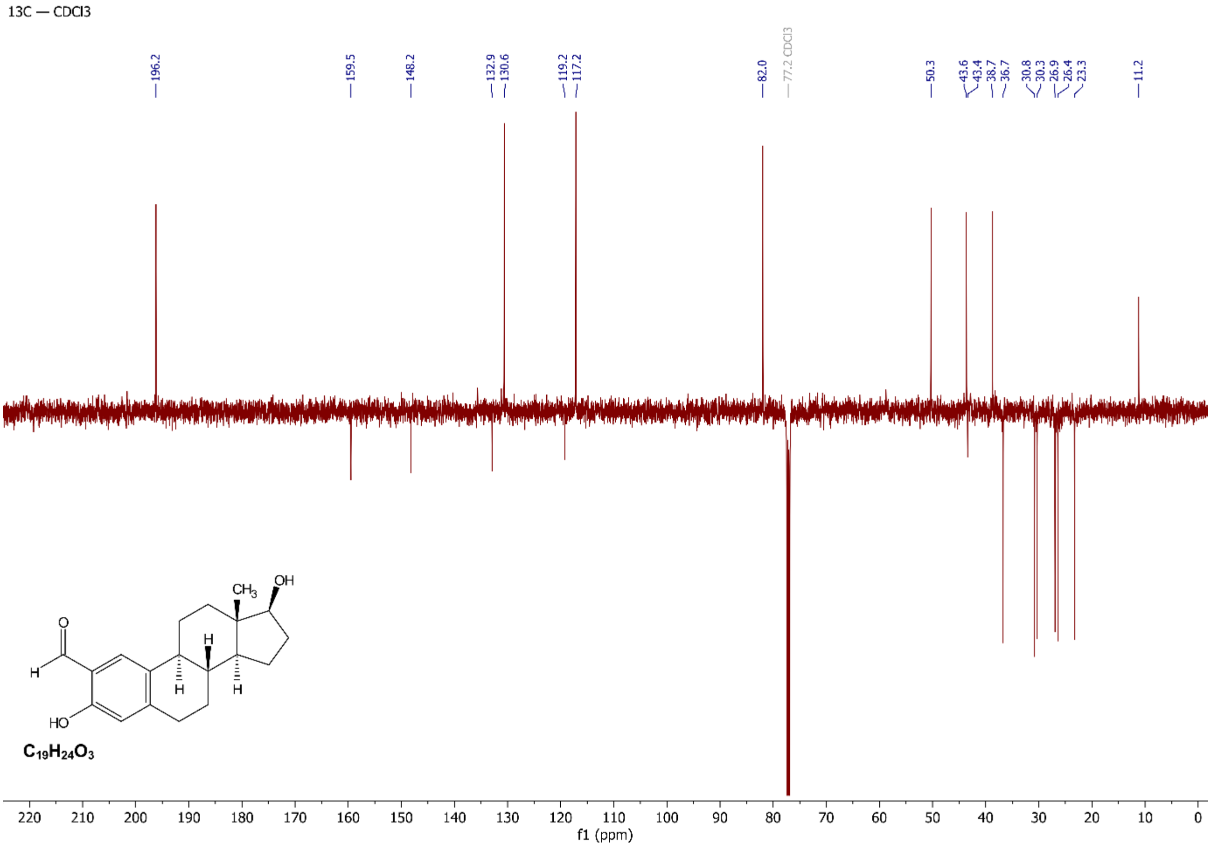

$^{1}H$ NMR spectrum of compound **2b**-17Ac ($CDCl_3$, 500 MHz)

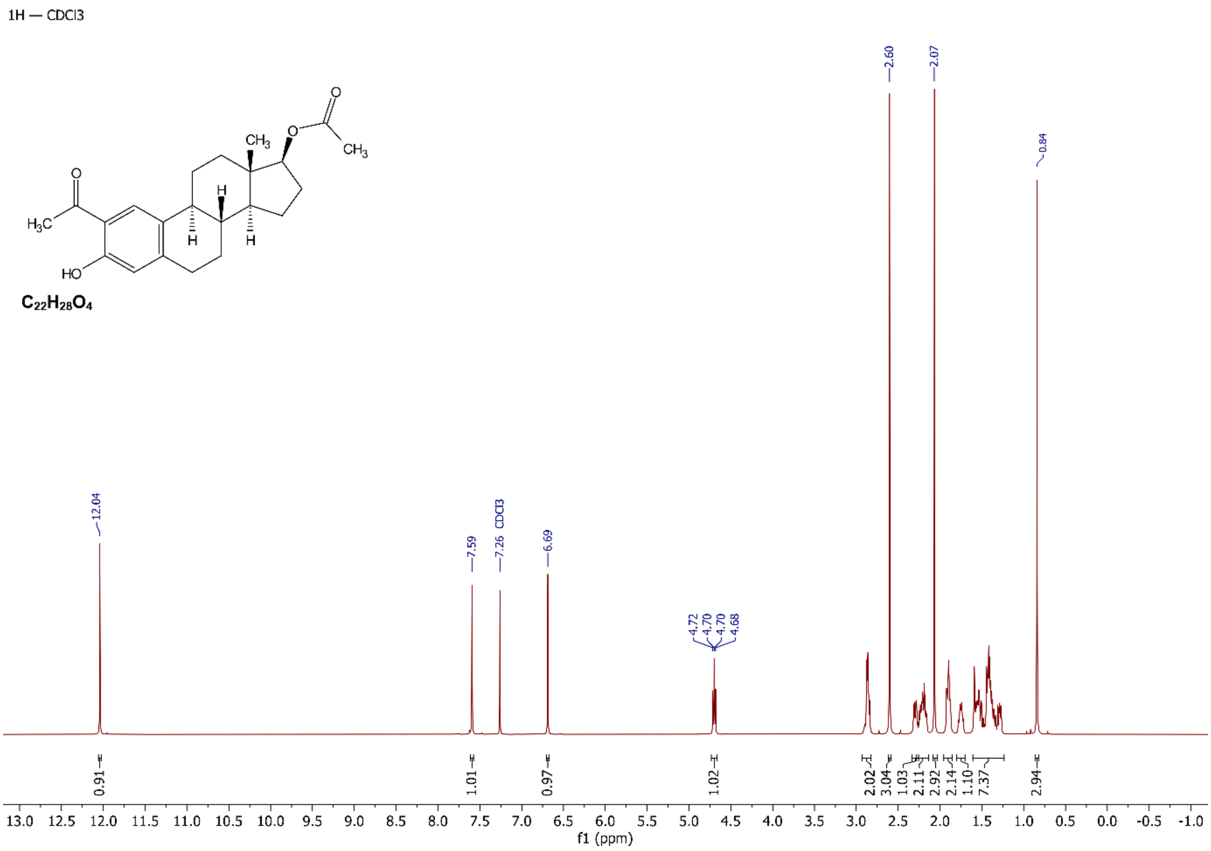


$^{13}C$ NMR spectrum of compound **2b**-17Ac ($CDCl_3$, 125 MHz)

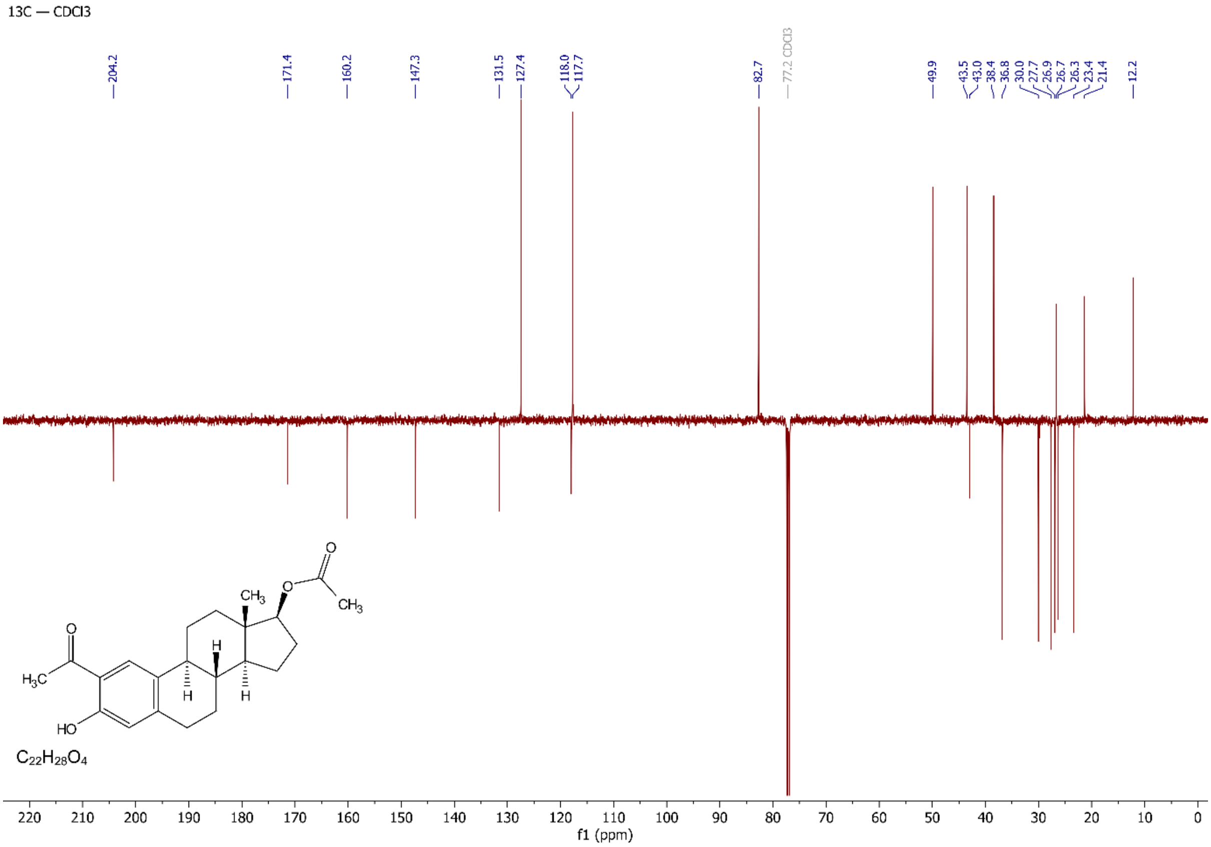

$^{1}H$ NMR spectrum of compound **2b** ($CDCl_3$, 500 MHz)

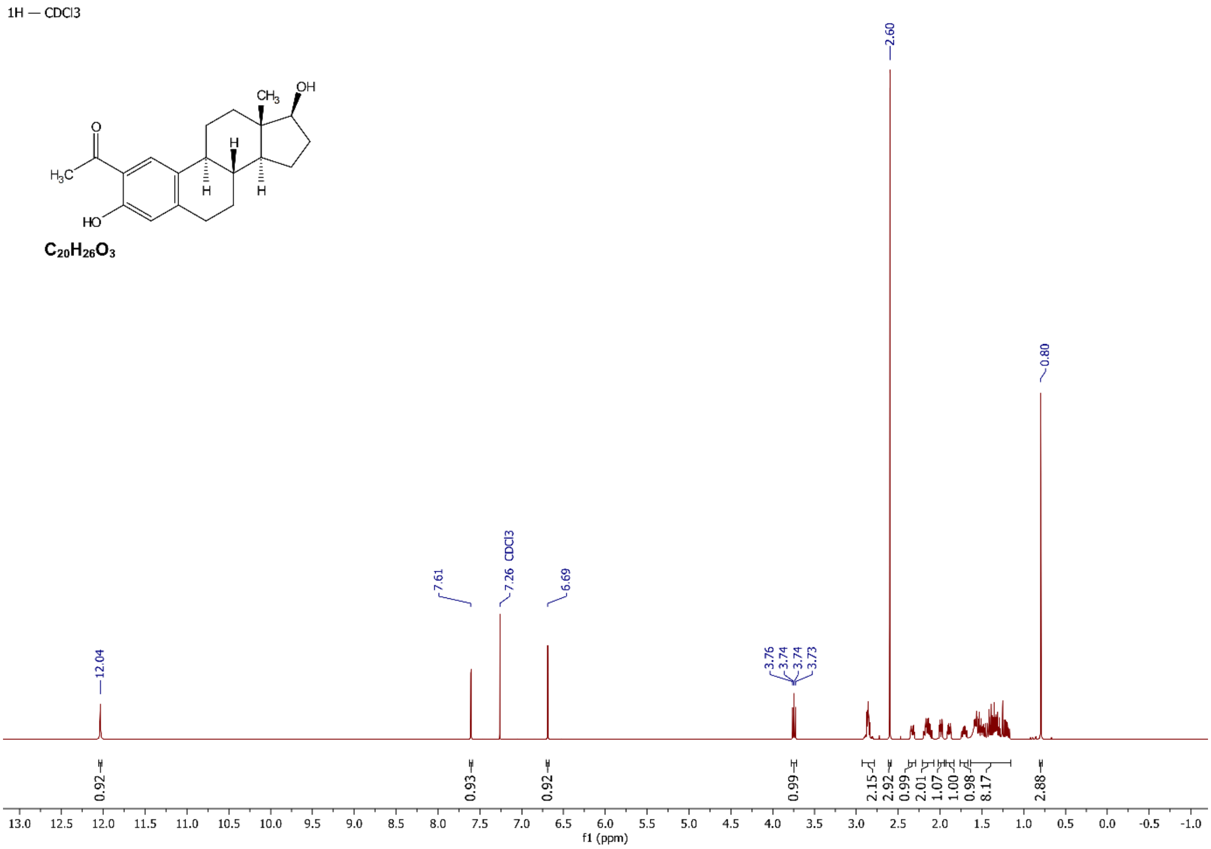


$^{13}C$ NMR spectrum of compound **2b** ($CDCl_3$, 125 MHz)

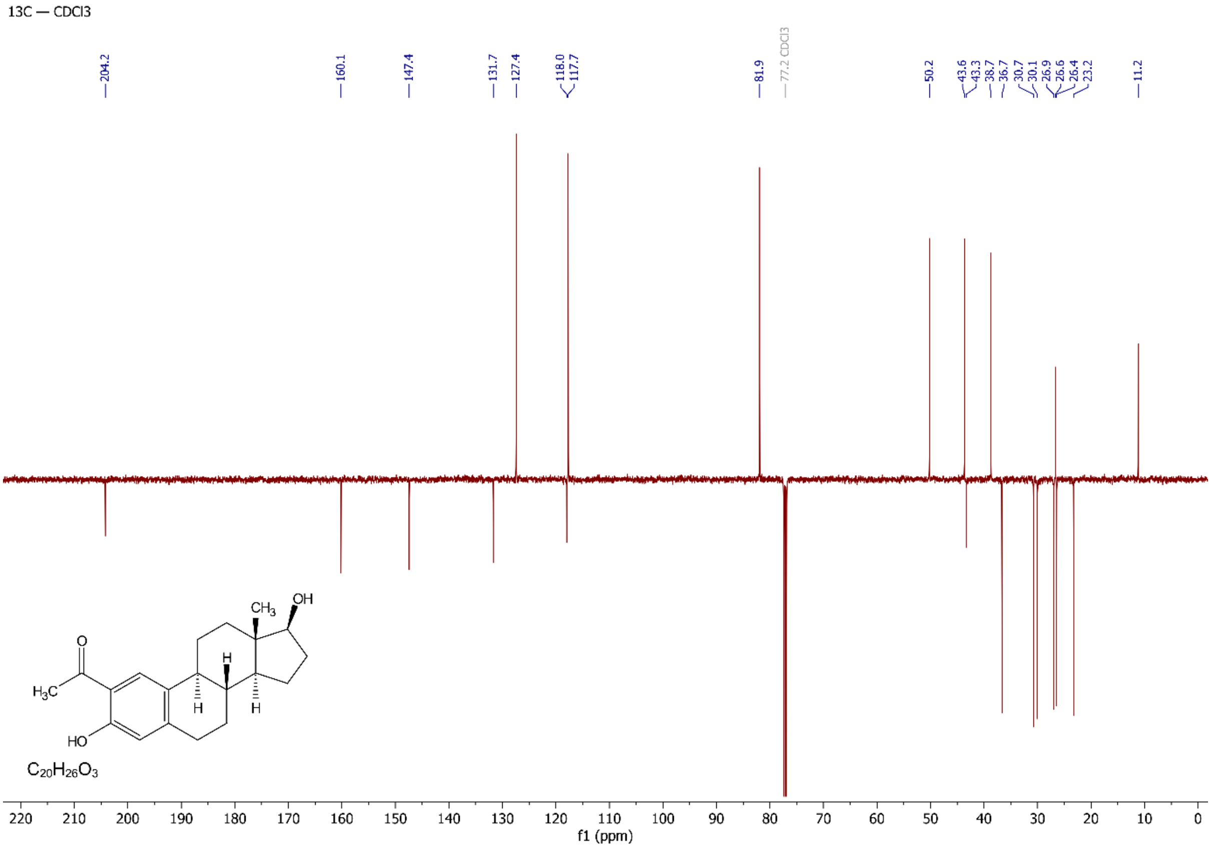

$^1H$ NMR spectrum of compound **2c-**17Ac ($CDCl_3$, 500 MHz)

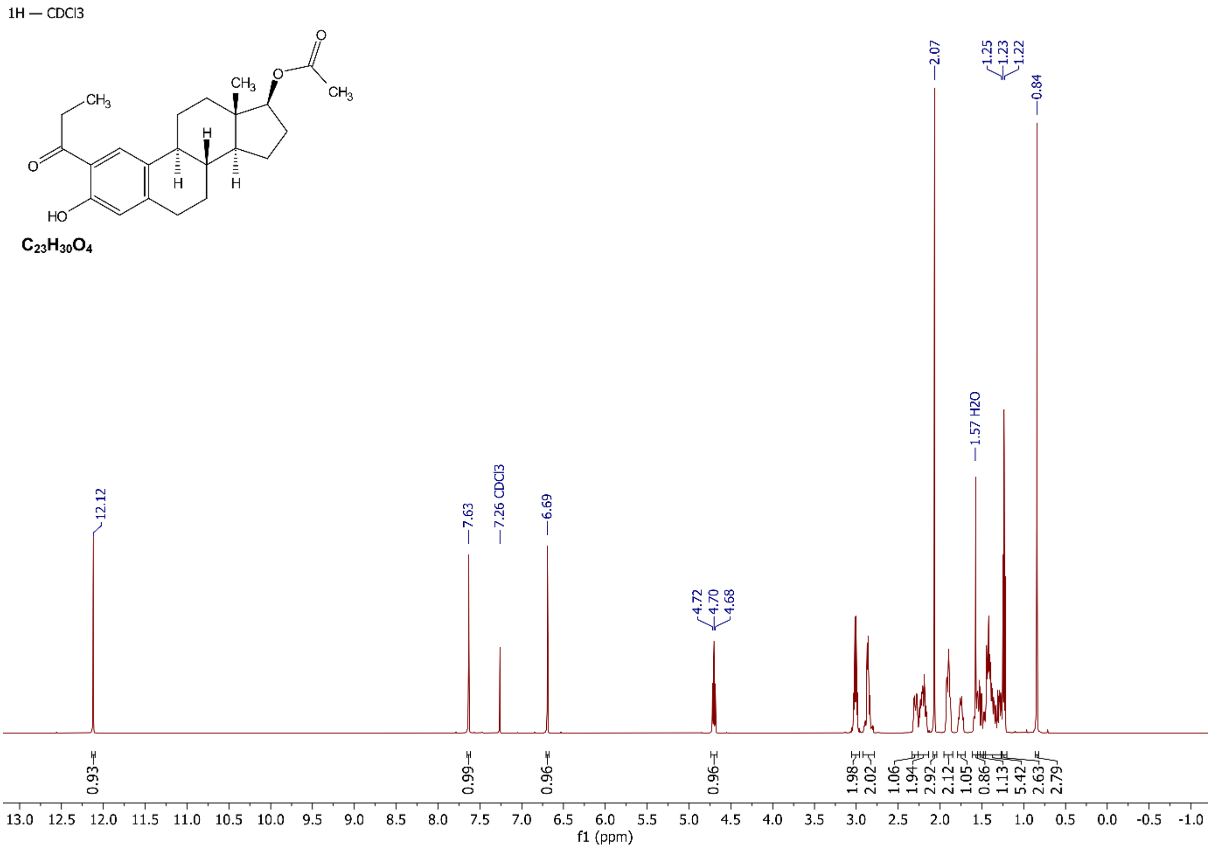


$^{13}C$ NMR spectrum of compound **2c-**17Ac ($CDCl_3$, 125 MHz)

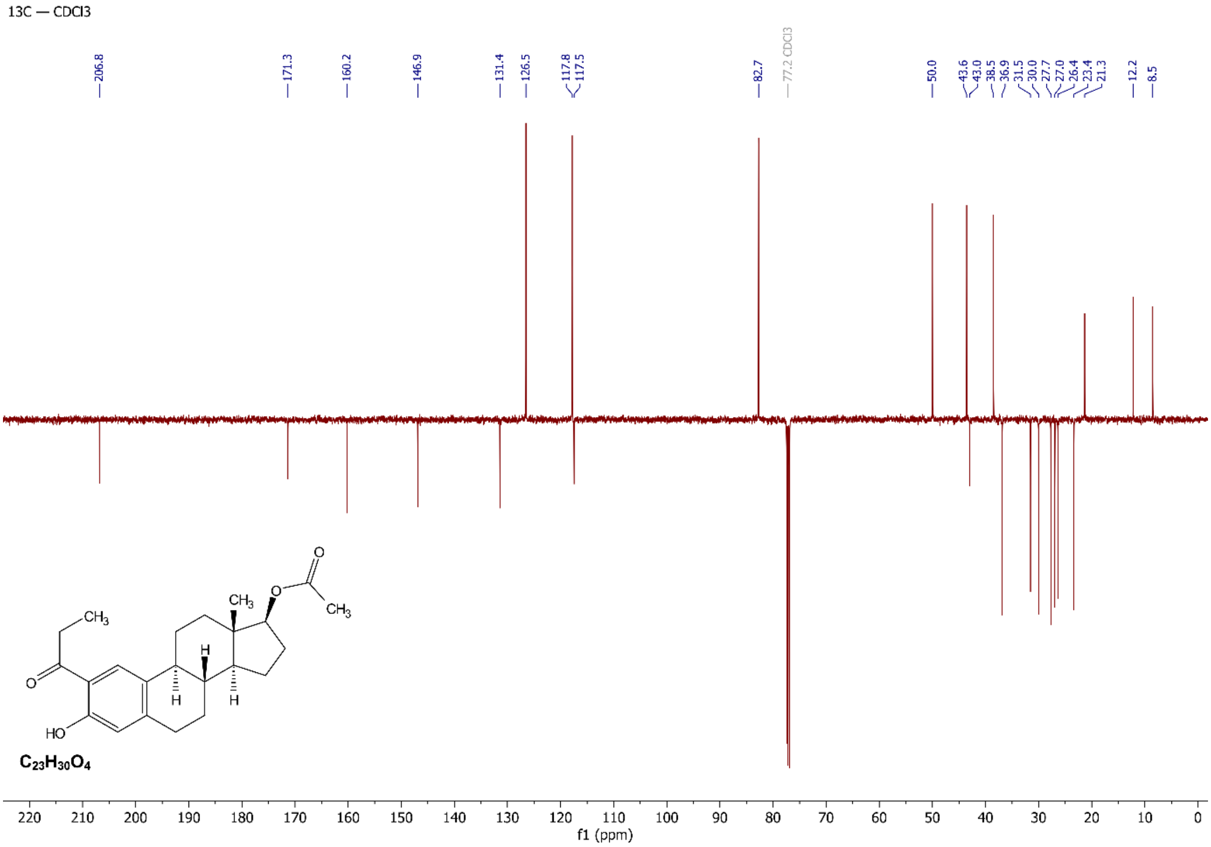

$^{1}$H NMR spectrum of compound **2c** ($CDCl_3$, 500 MHz)

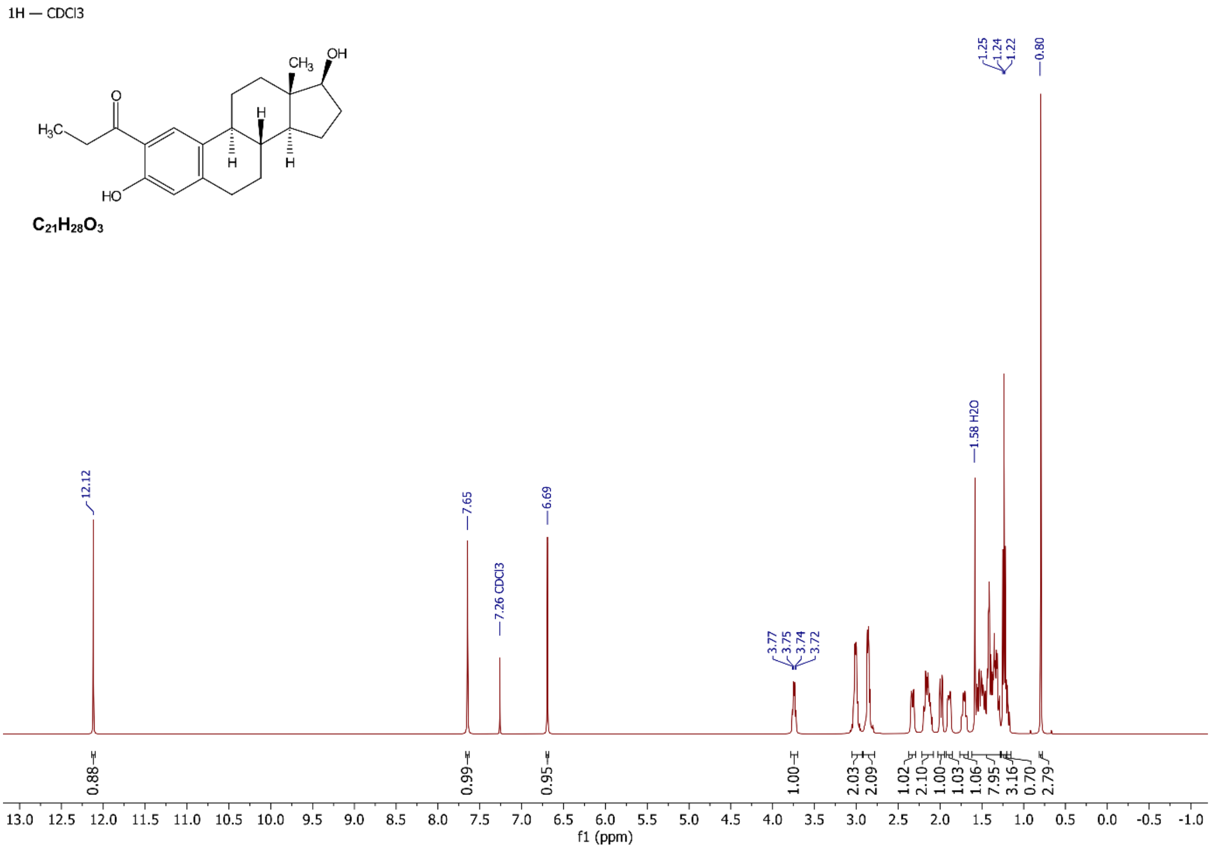


$^{13}$C NMR spectrum of compound **2c** ($CDCl_3$, 125 MHz)

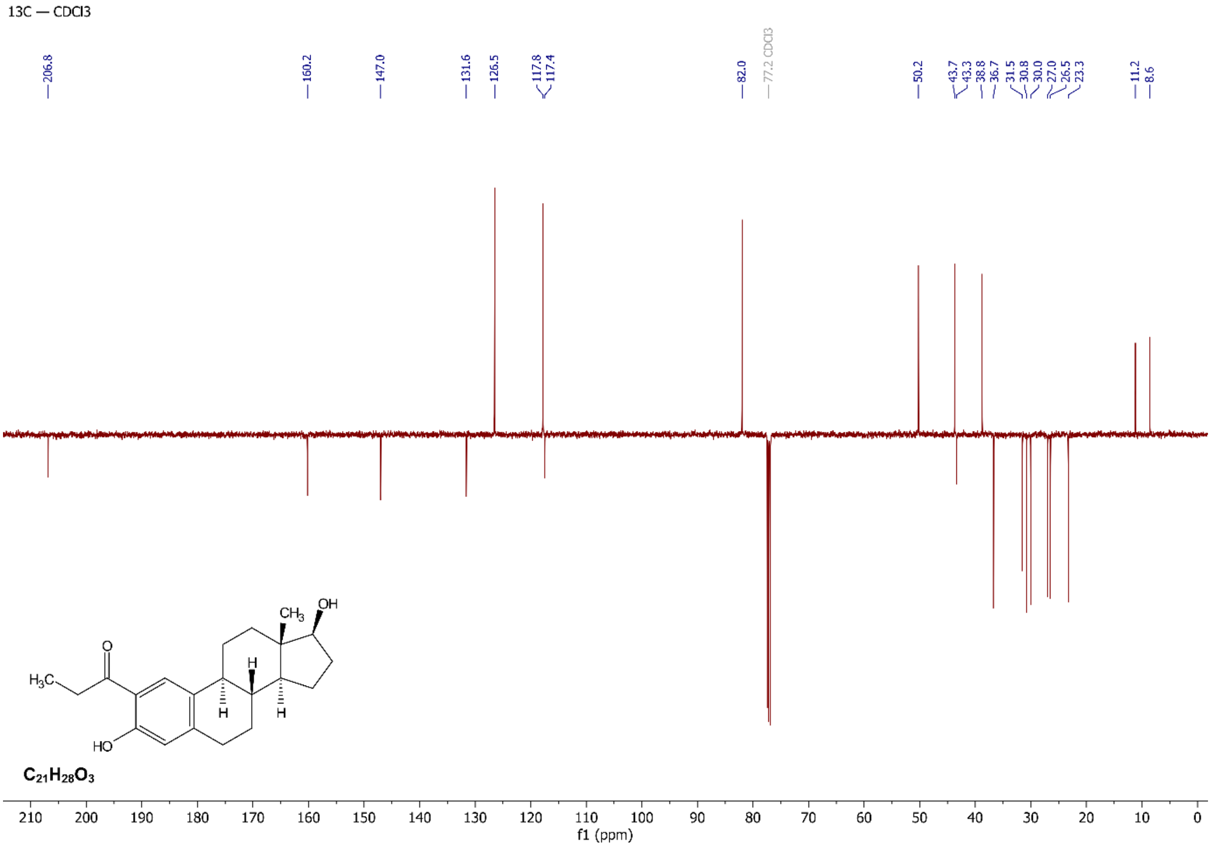

$^{1}$H NMR spectrum of compound **2d-**17Ac (CDCl3, 500 MHz)

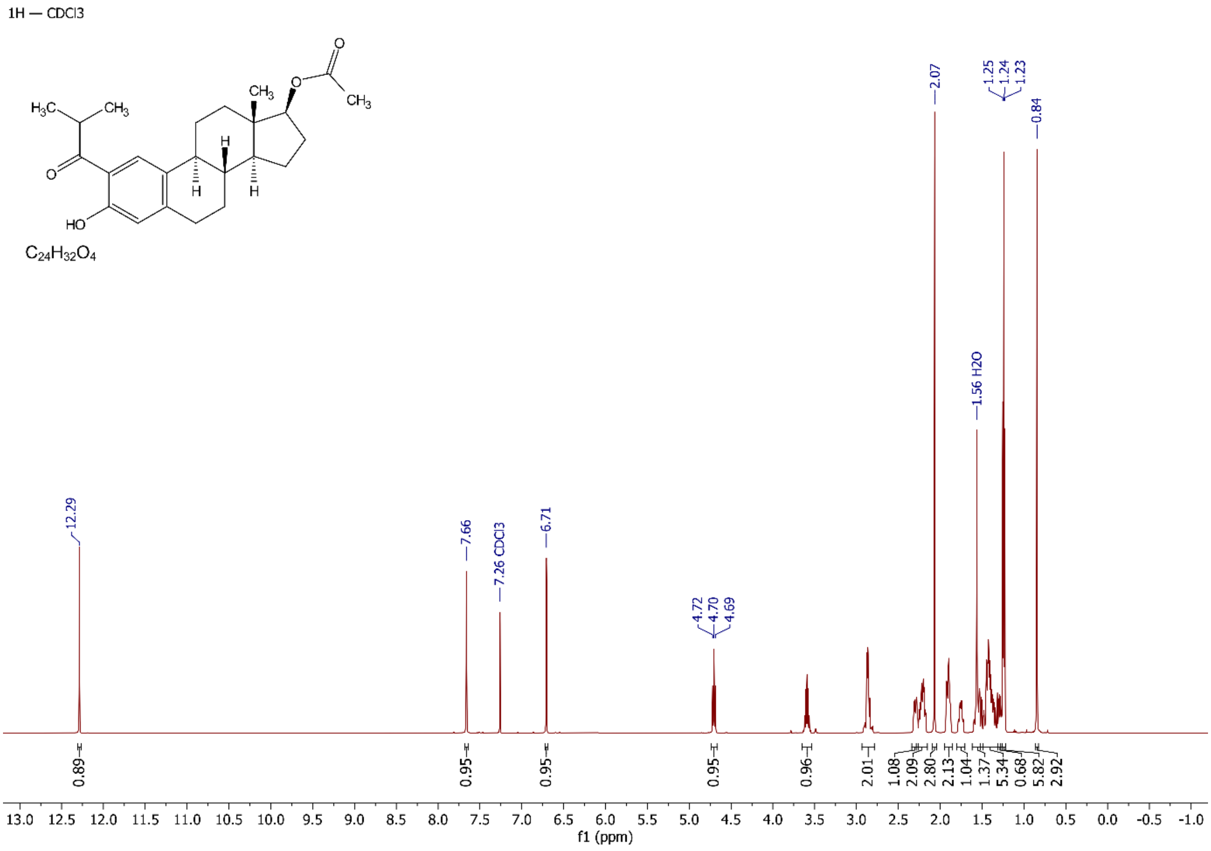


$^{13}$C NMR spectrum of compound **2d-**Ac (CDCl3, 125 MHz)

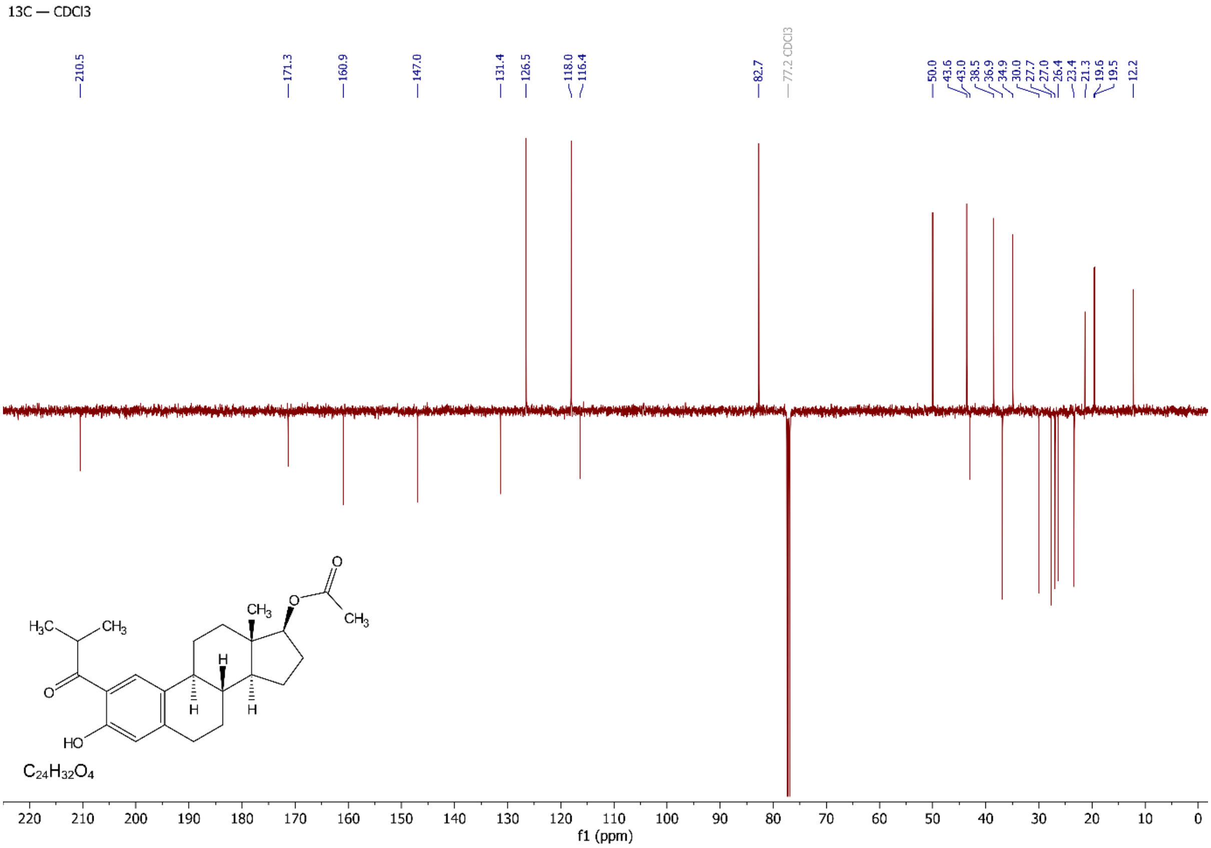

$^{1}H$ NMR spectrum of compound **2d** ($CDCl_3$, 500 MHz)

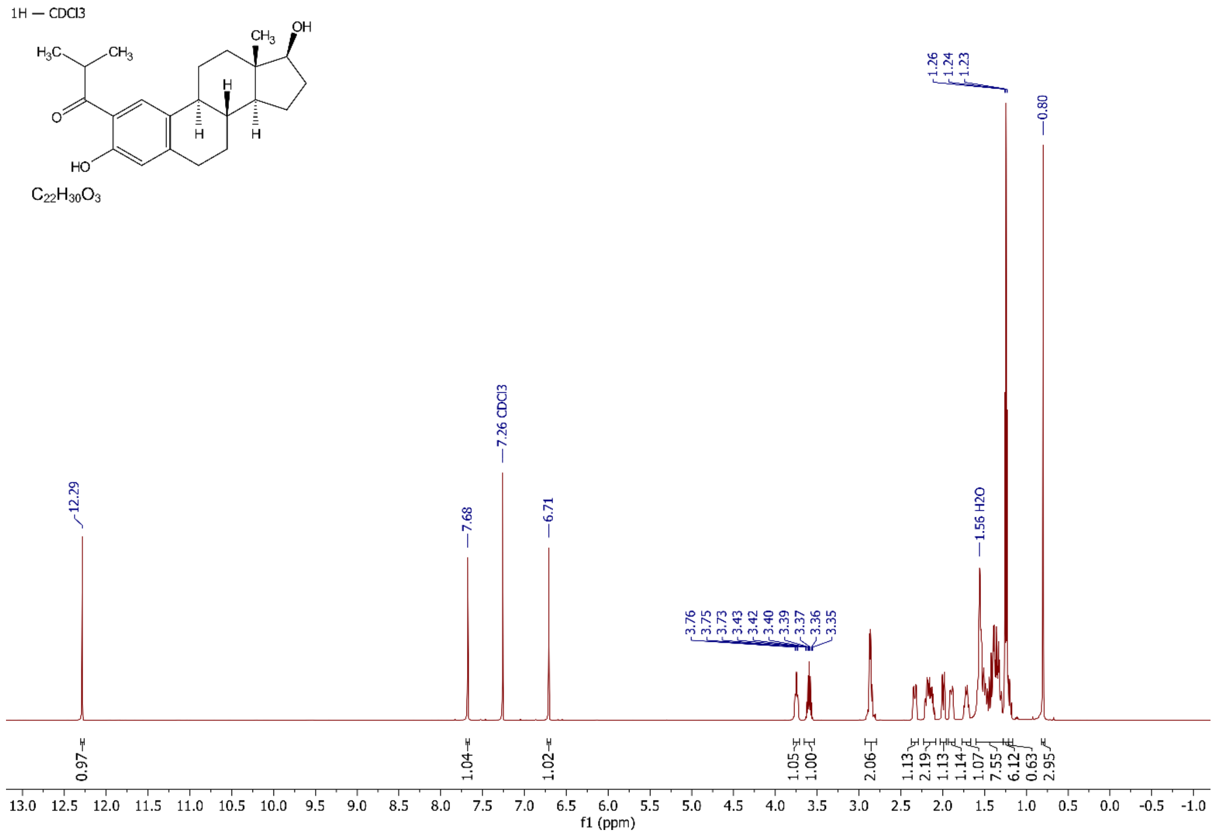


$^{13}C$ NMR spectrum of compound **2d** ($CDCl_3$, 125 MHz)

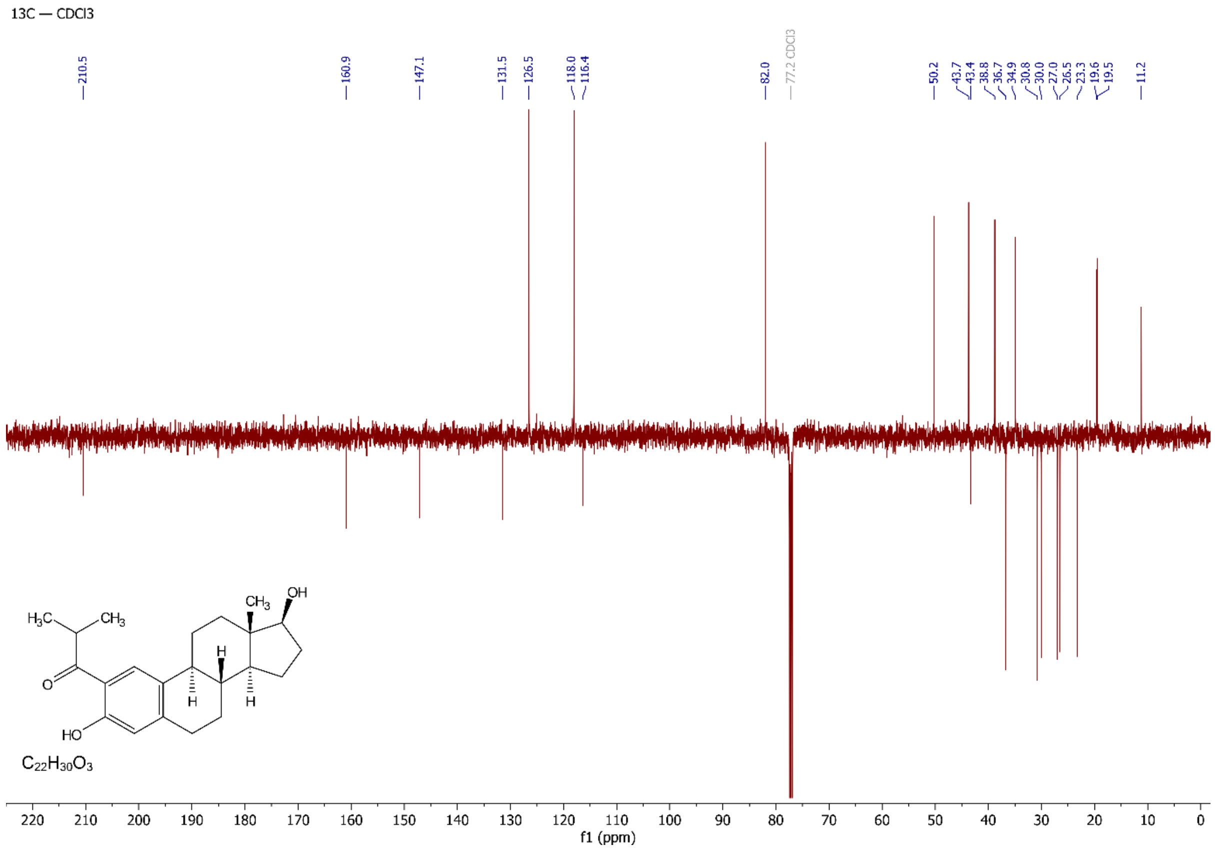

$^1$H NMR spectrum of compound **2e-**17Ac ($CDCl_3$, 500 MHz)

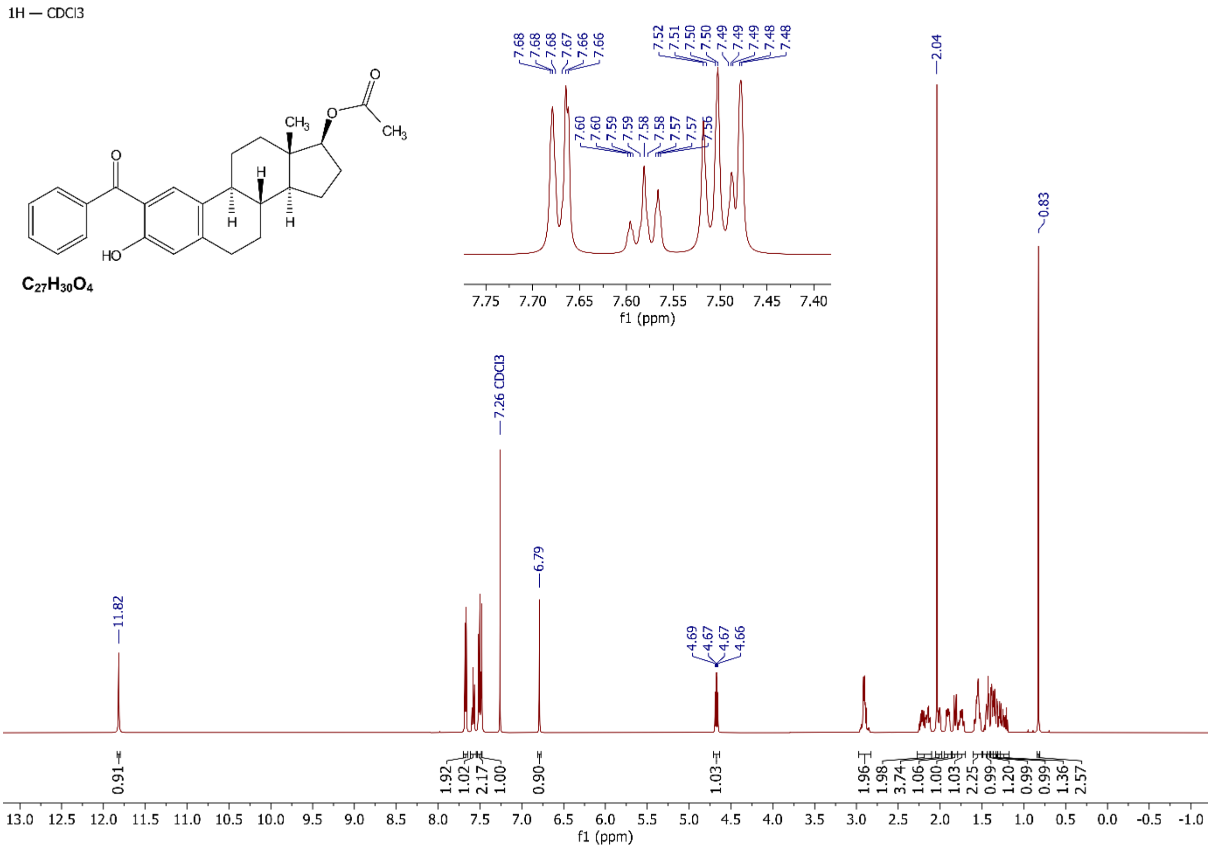


$^{13}$C NMR spectrum of compound **2e-**17Ac ($CDCl_3$, 125 MHz)

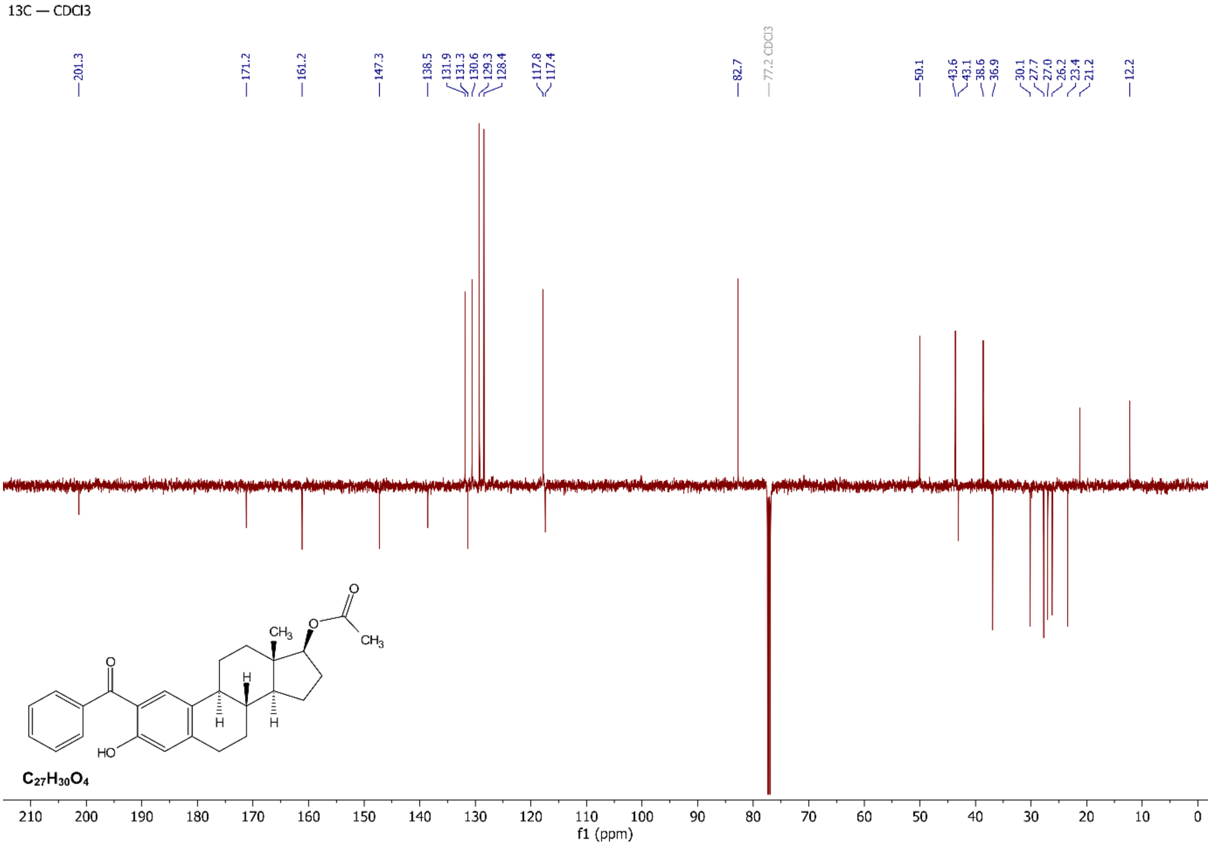

$^{1}H$ NMR spectrum of compound **2e** ($CDCl_3$, 500 MHz)

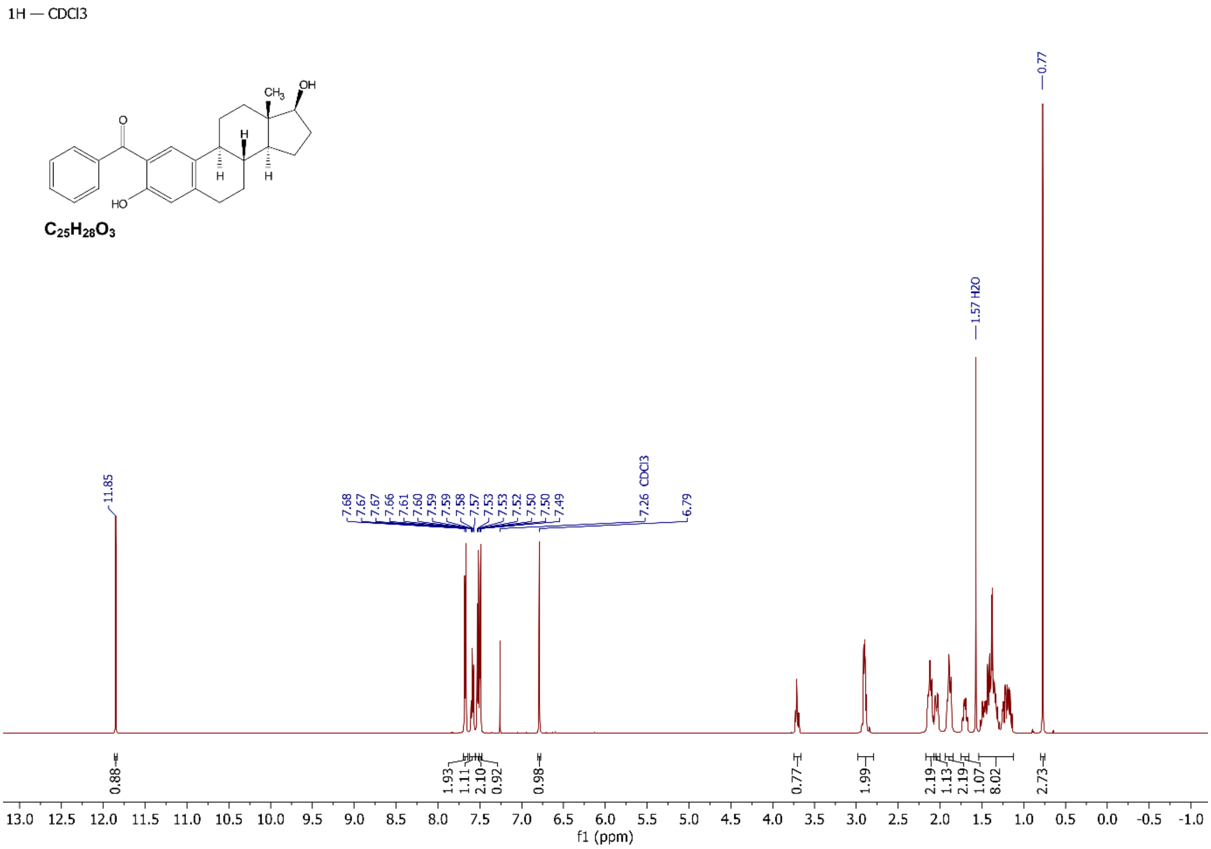


$^{13}C$ NMR spectrum of compound **2e** ($CDCl_3$, 125 MHz)

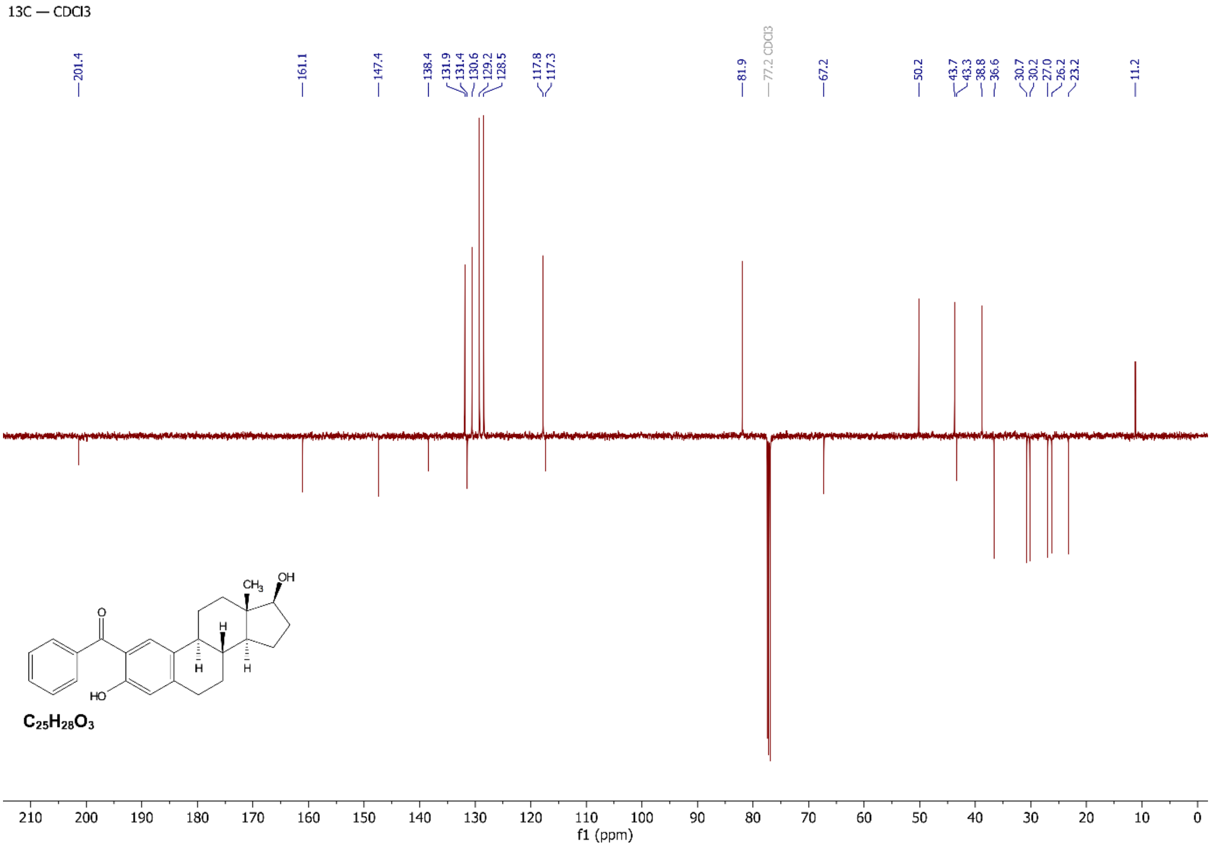

$^{1}$H NMR spectrum of compound **2f** ($CDCl_3$, 500 MHz) 81

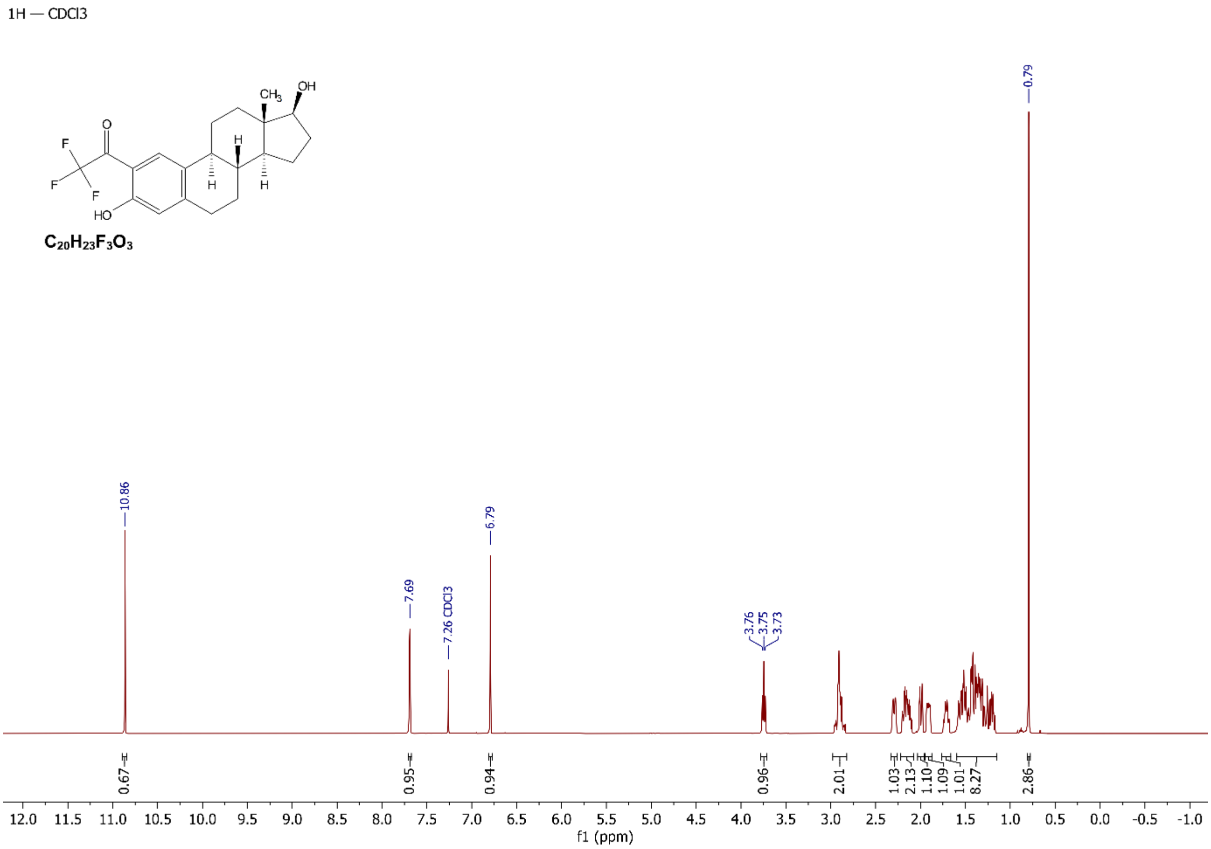


82

$^{13}$C NMR spectrum of compound **2f** ($CDCl_3$, 125 MHz) 83

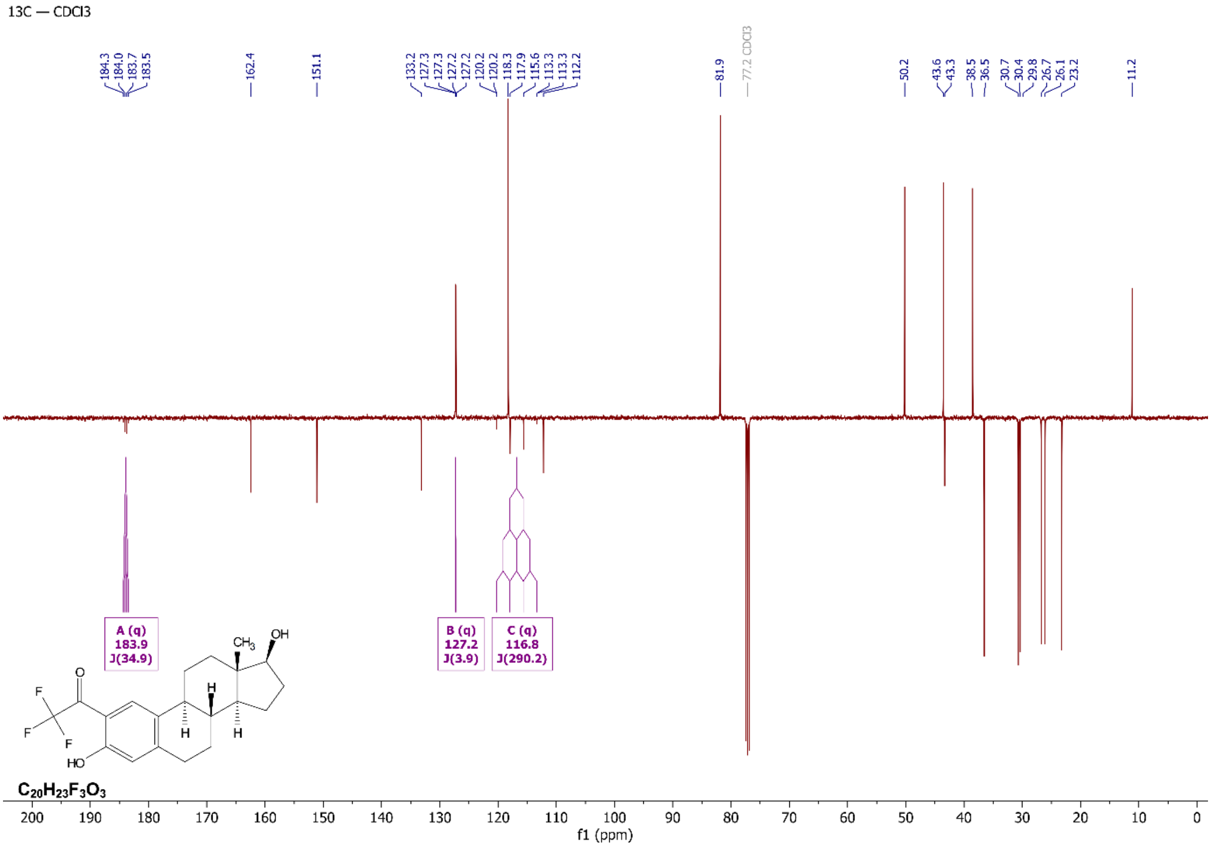


84

$^{1}H$ NMR spectrum of compound **2g** ($CDCl_3$, 500 MHz)

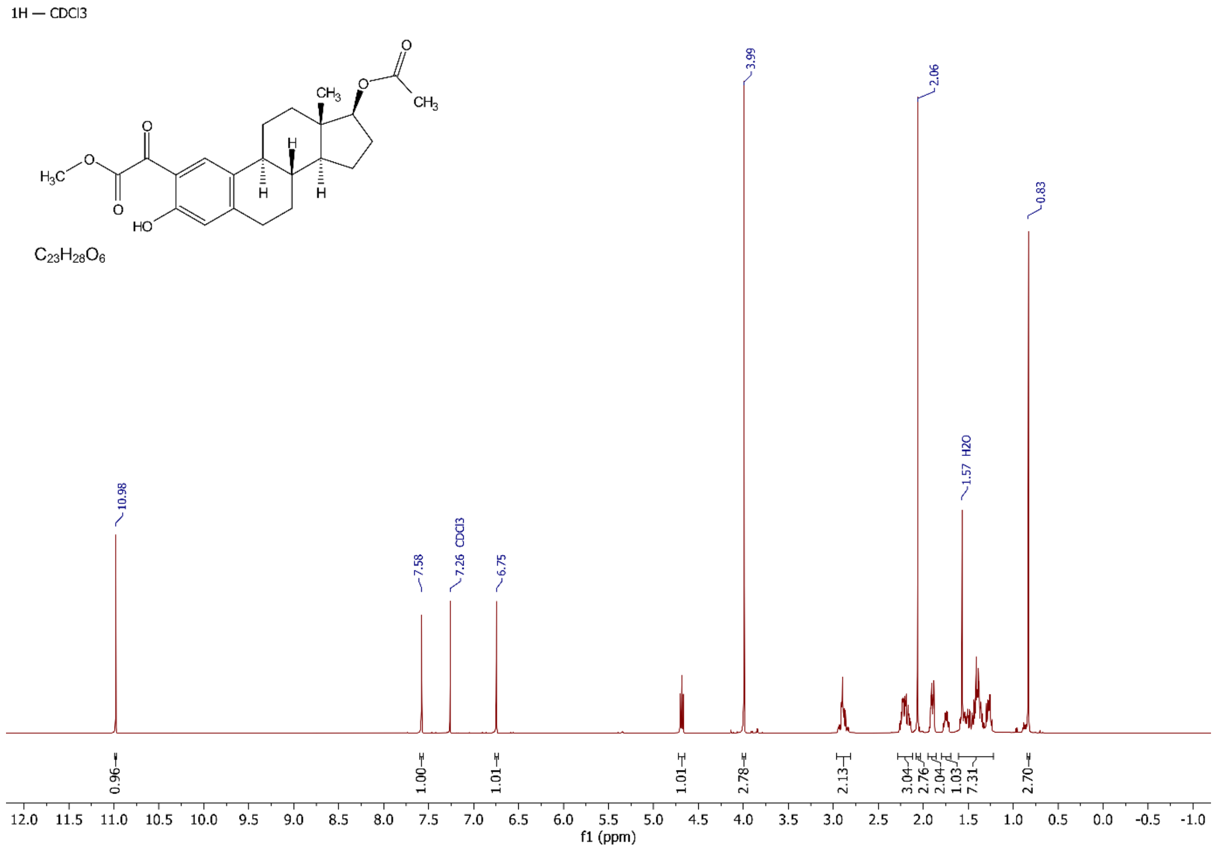


$^{13}C$ NMR spectrum of compound **2g** ($CDCl_3$, 125 MHz)

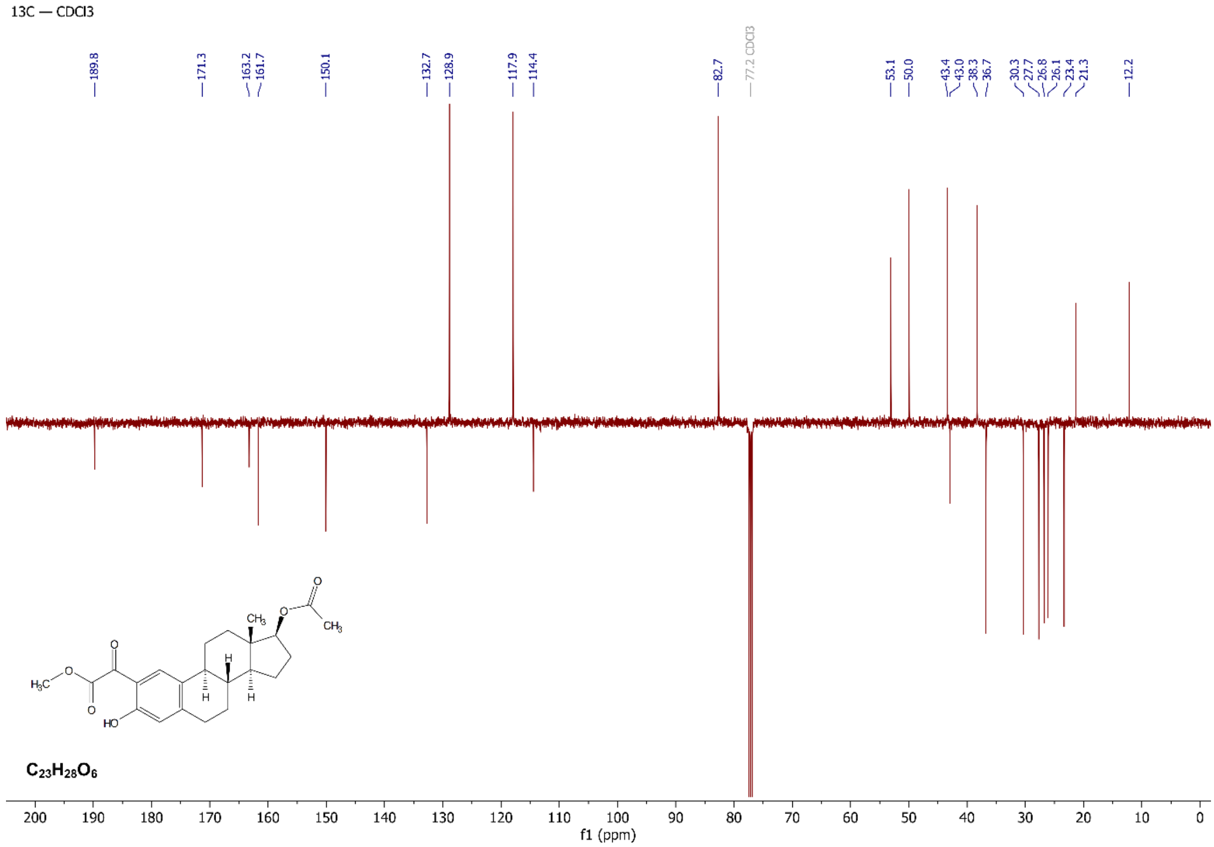

$^{1}H$ NMR spectrum of compound **3a** (DMSO-*d*$_6$, 500 MHz)

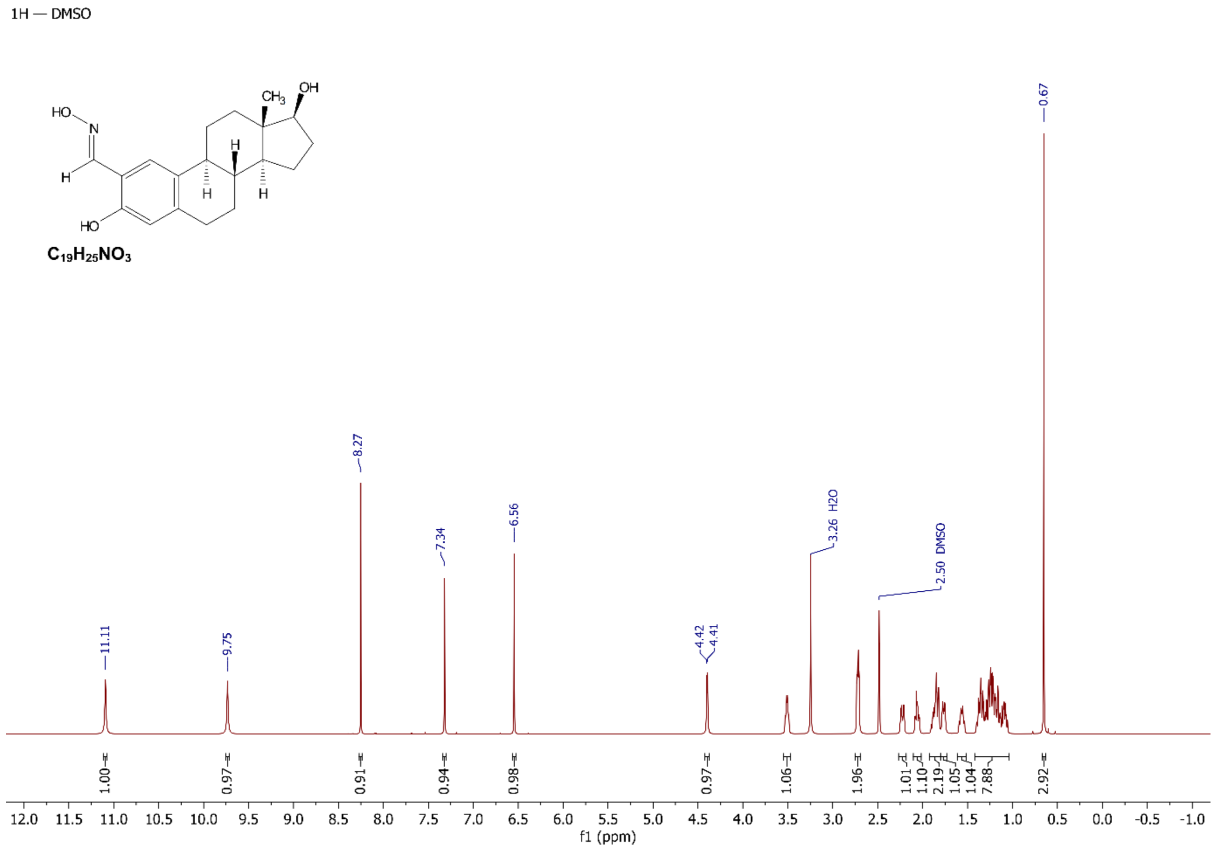


$^{13}C$ NMR spectrum of compound **3a** (DMSO-*d*$_6$, 125 MHz)

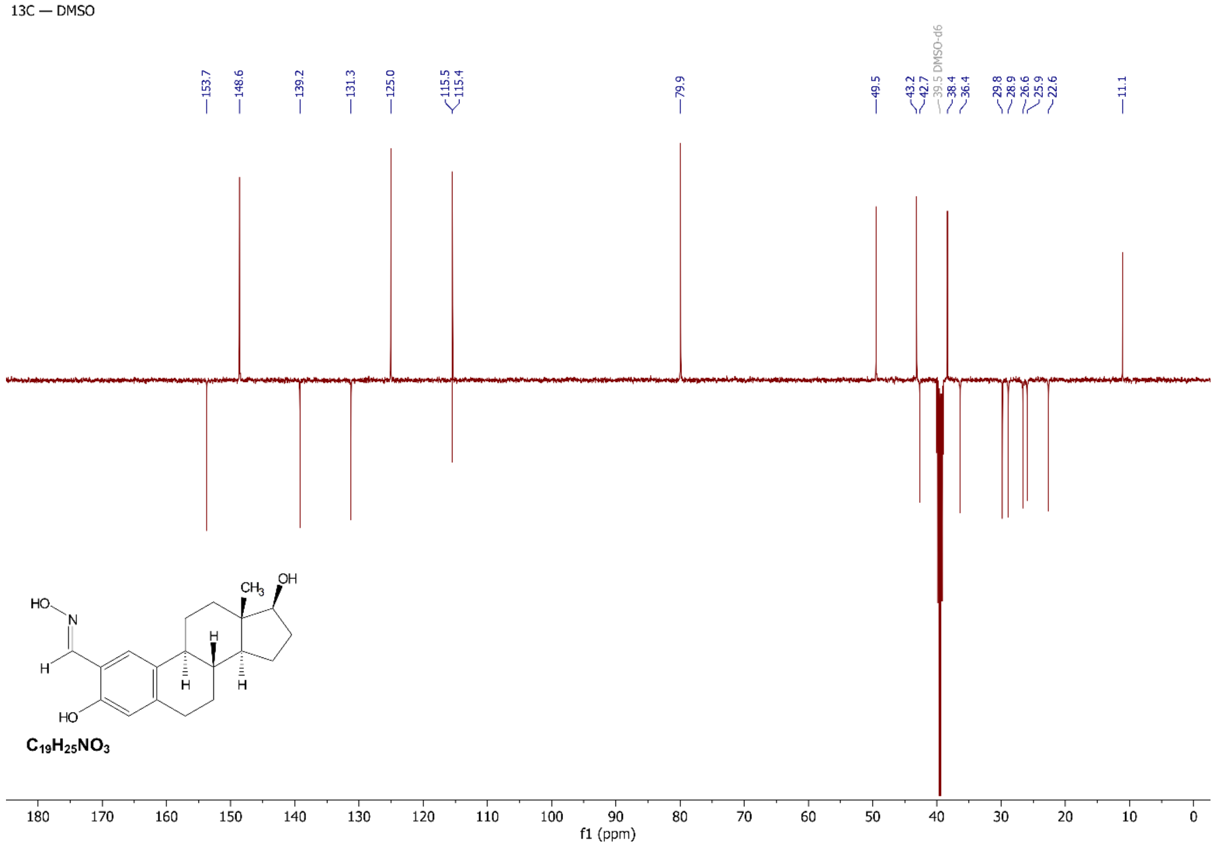

$^{1}H$ NMR spectrum of compound **3b** ($CDCl_3$, 500 MHz)

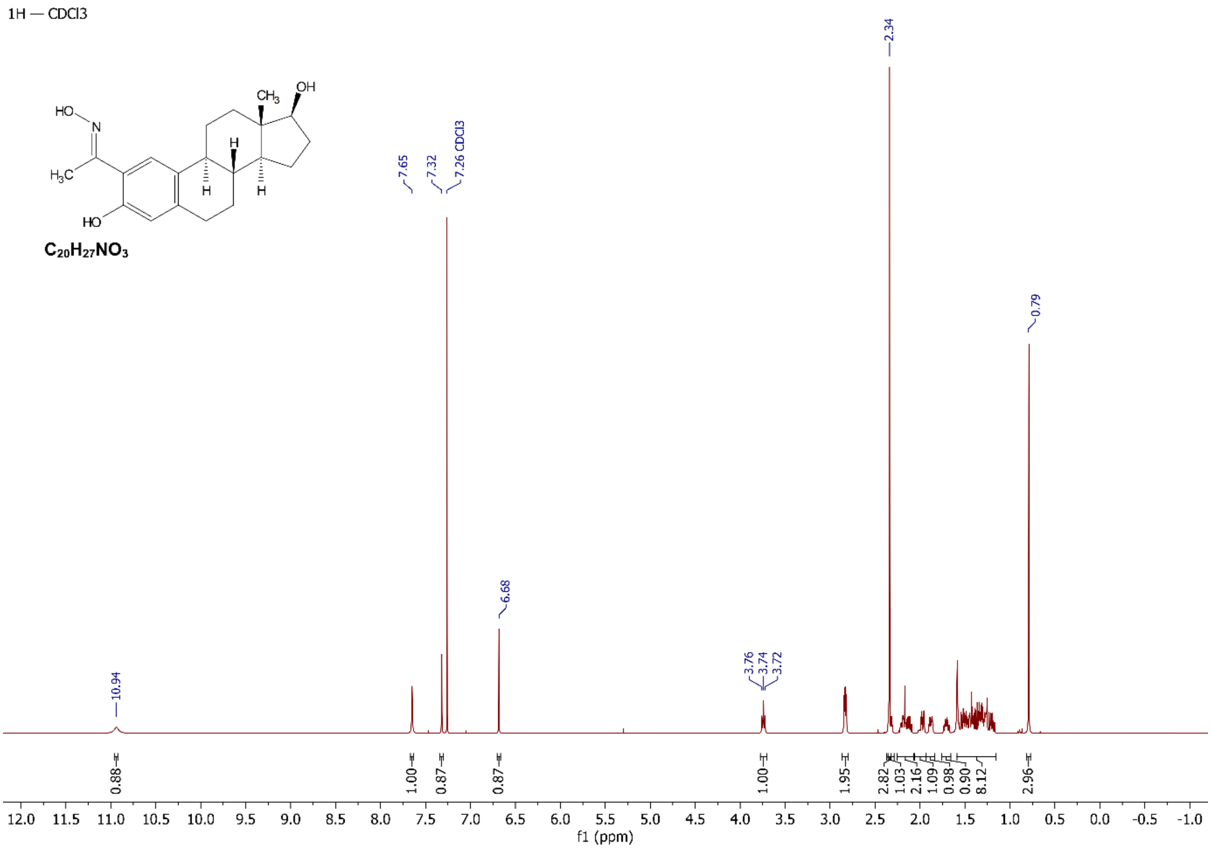


$^{13}C$ NMR spectrum of compound **3b** ($CDCl_3$, 125 MHz)

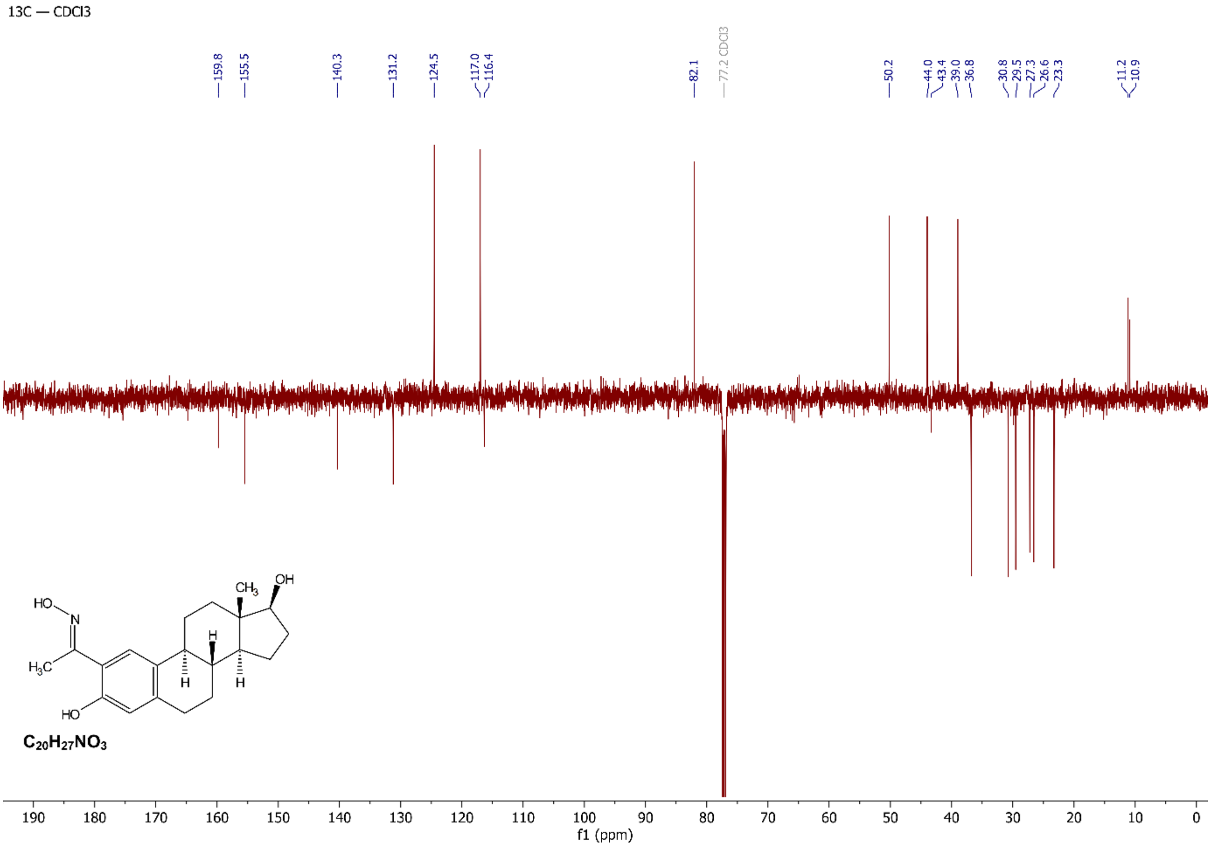

$^{1}$H NMR spectrum of compound **3c** (DMSO-*d*6, 500 MHz)

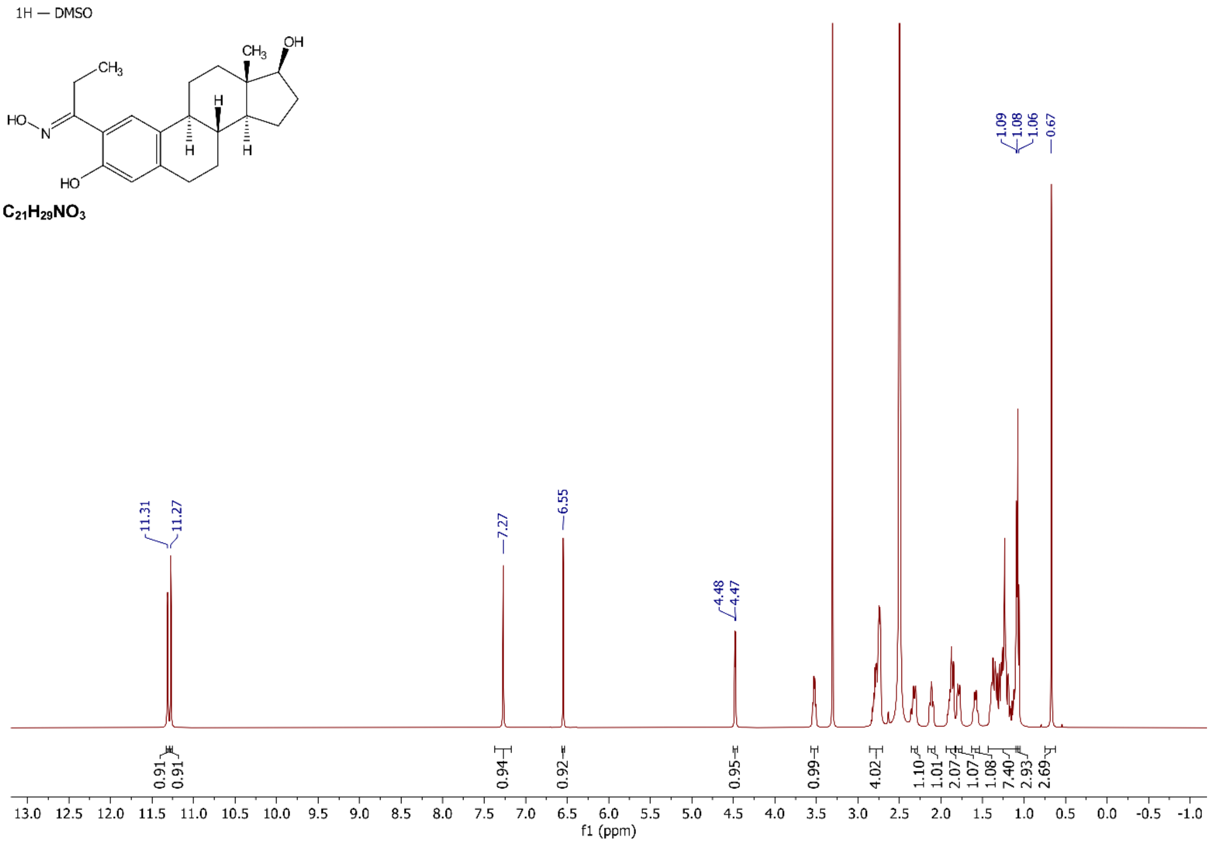


$^{13}$C NMR spectrum of compound **3c** (DMSO-*d*6, 125 MHz)

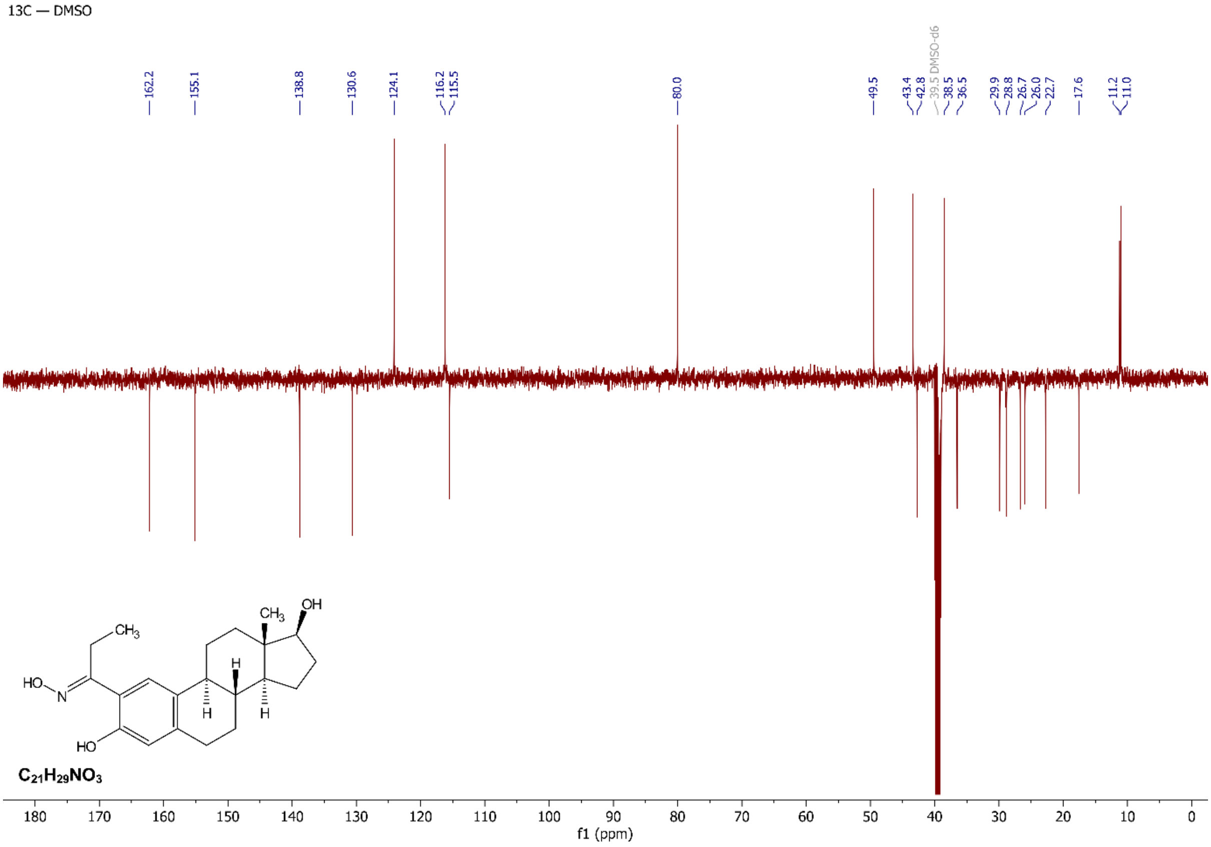

$^{1}$H NMR spectrum of compound **3d-*E*** (DMSO-$d_6$, 500 MHz)

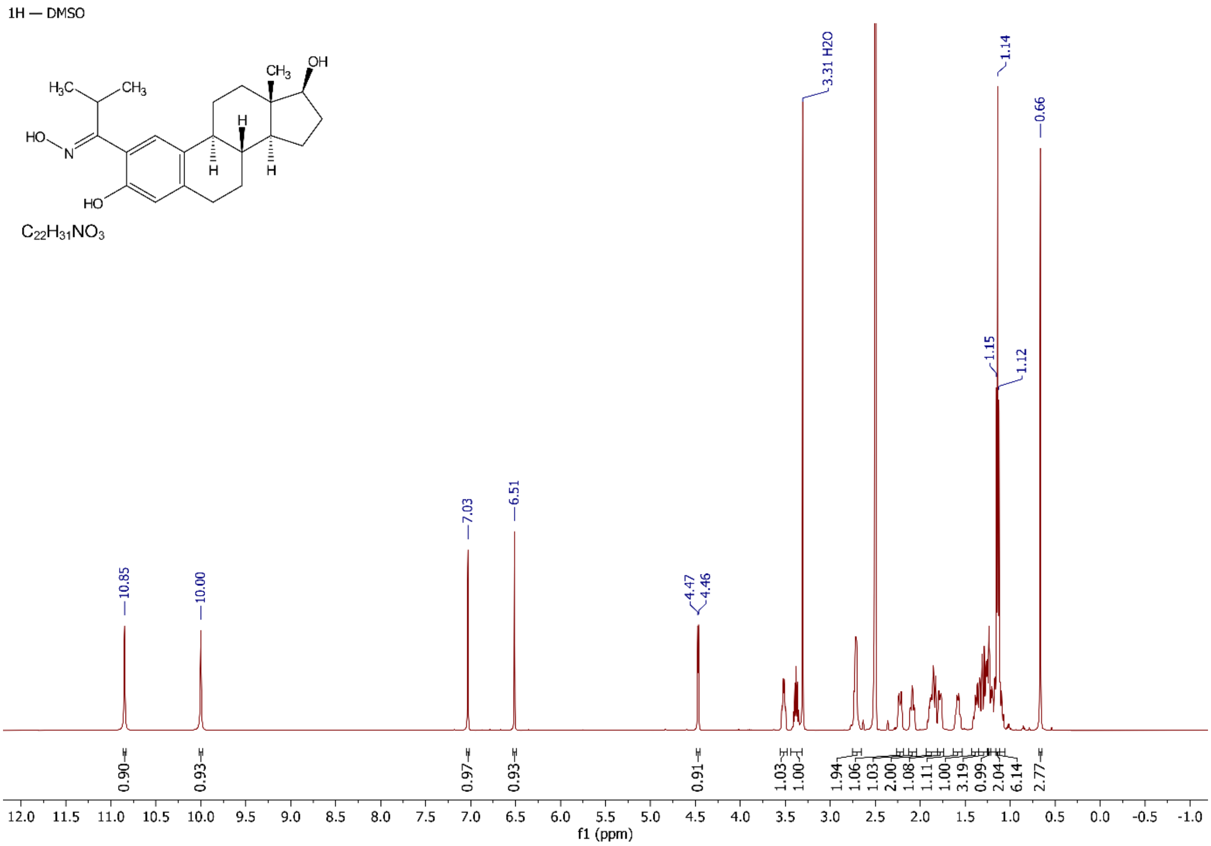


$^{13}$C NMR spectrum of compound **3d-*E*** (DMSO-$d_6$, 125 MHz)

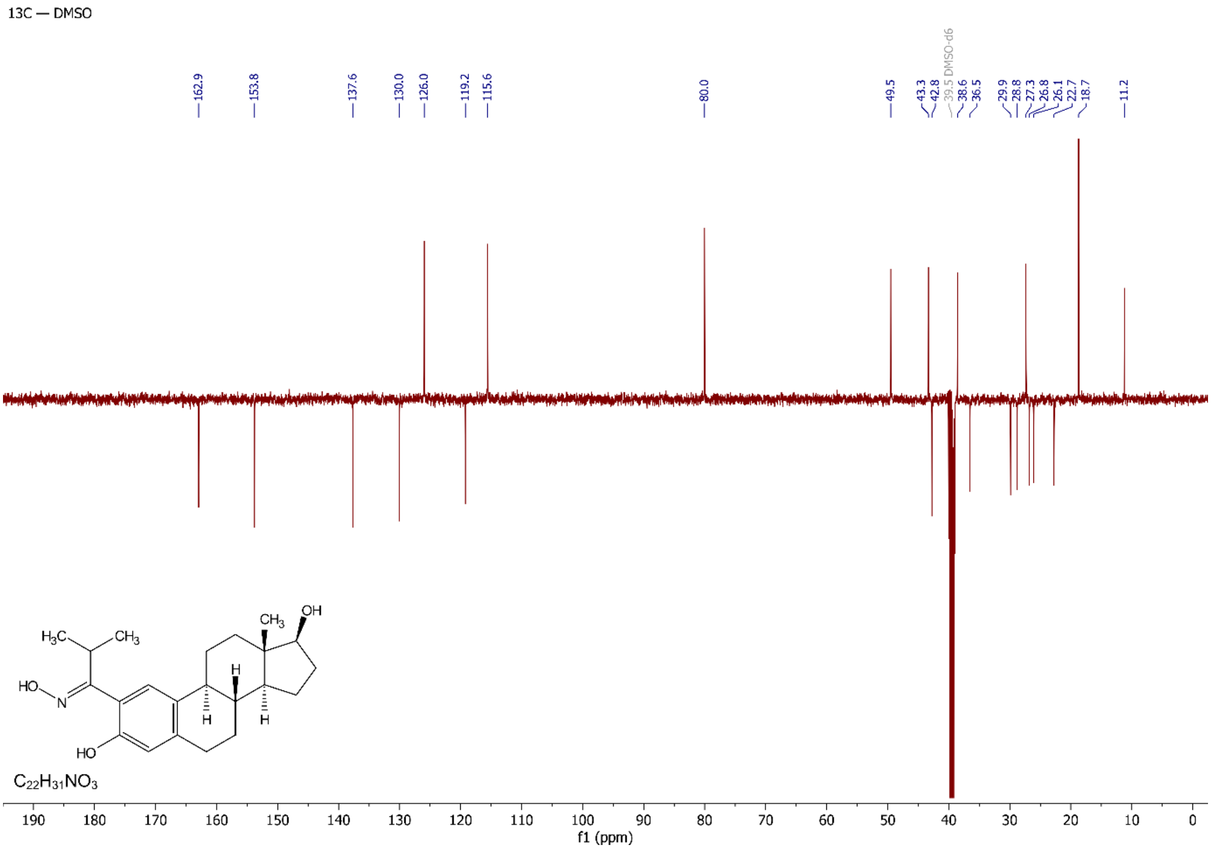

$^{1}H$ NMR spectrum of compound **3d**-*Z* (DMSO-*d*$_6$, 500 MHz)

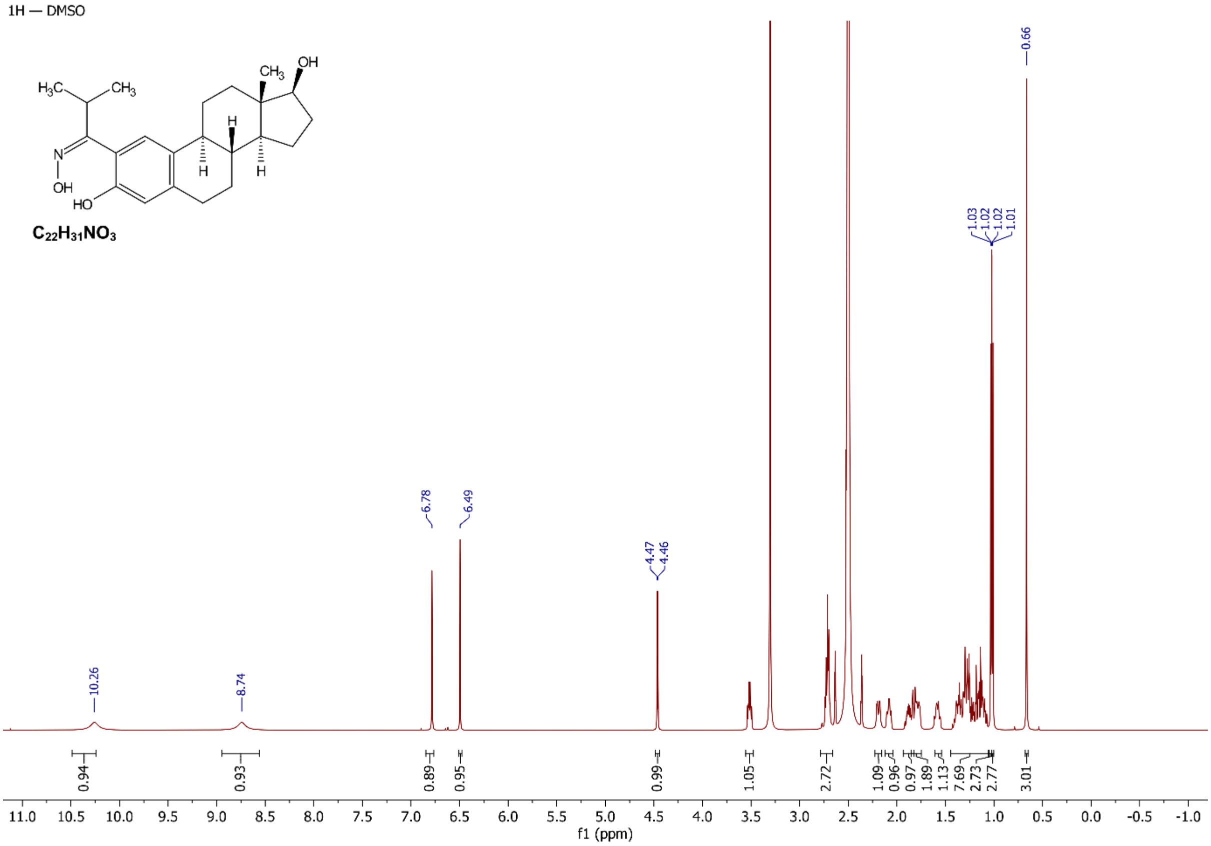


$^{13}C$ NMR spectrum of compound **3d**-*Z* (DMSO-*d*$_6$, 125 MHz)

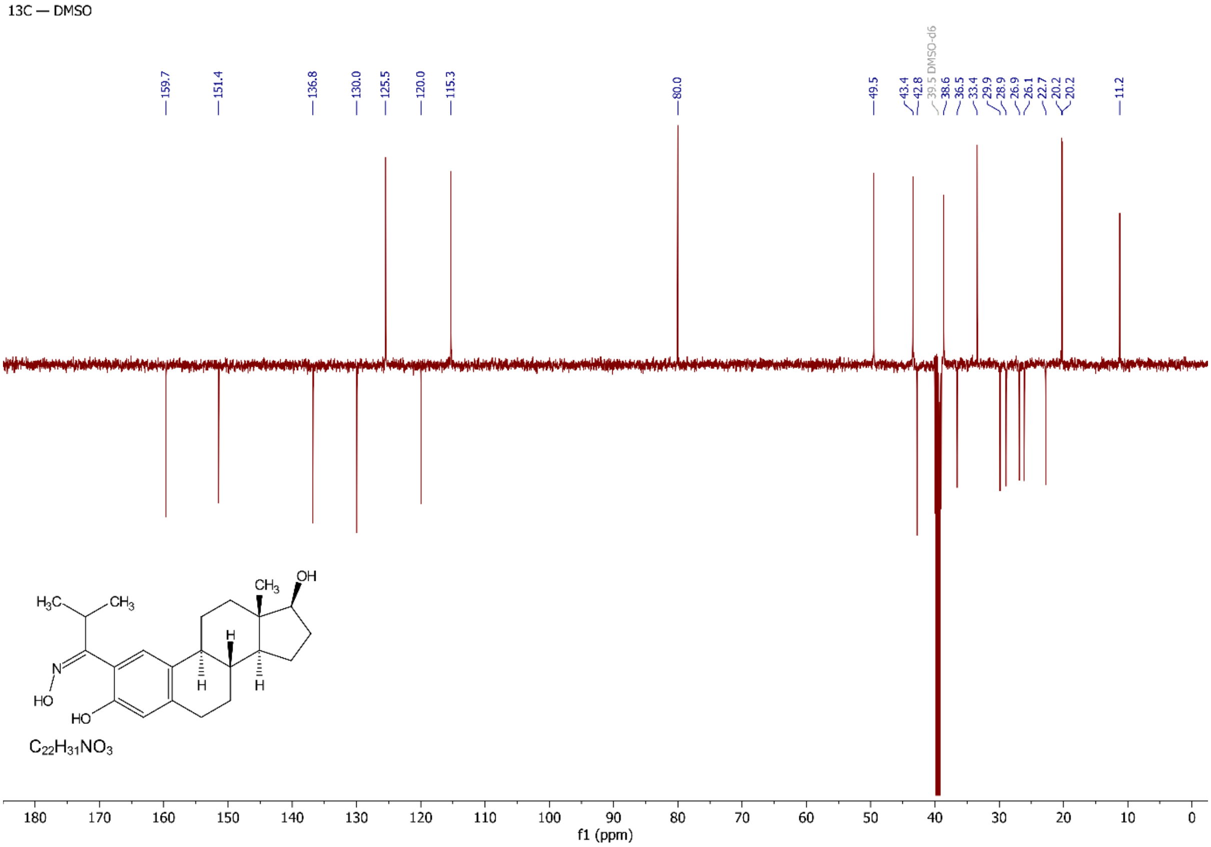

$^{1}H$ NMR spectrum of compound **3e-*E*** (DMSO-*d*$_6$, 500 MHz)

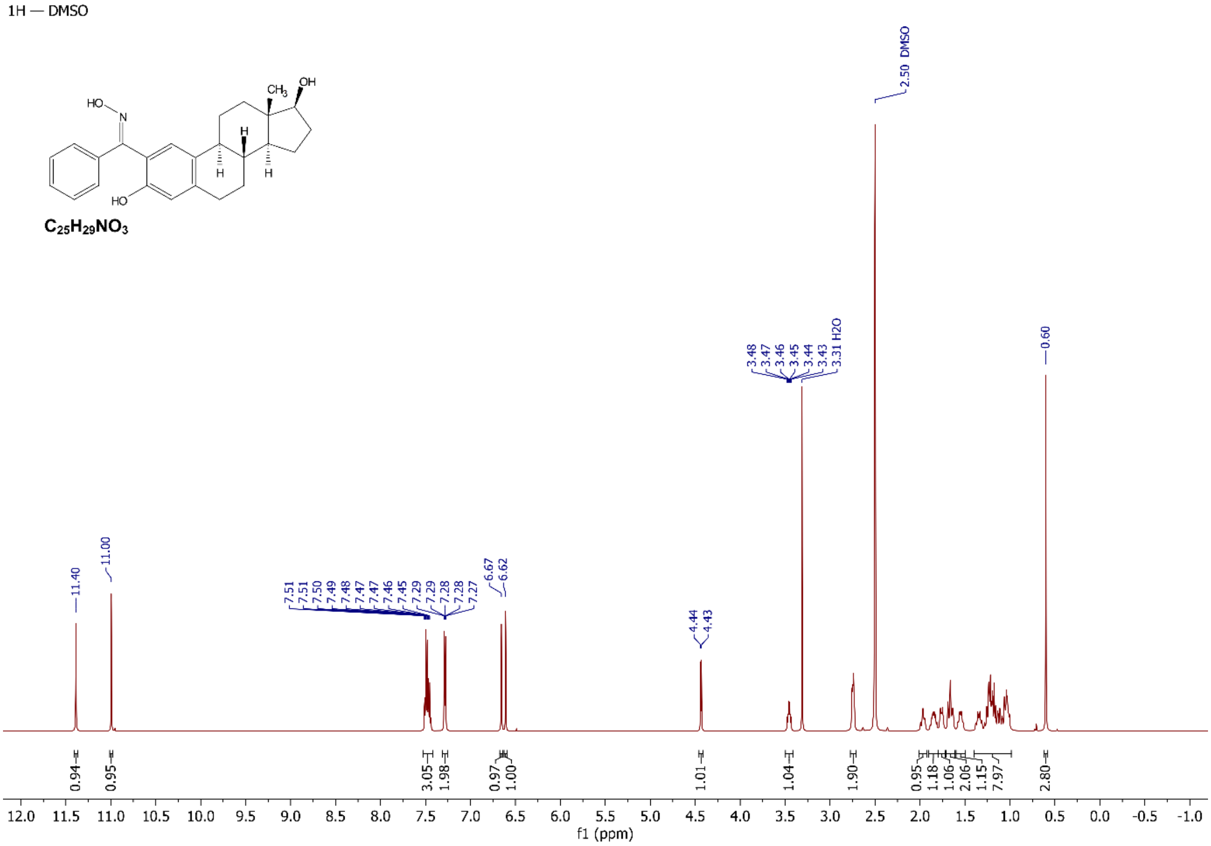


$^{13}C$ NMR spectrum of compound **3e-*E*** (DMSO-*d*$_6$, 125 MHz)

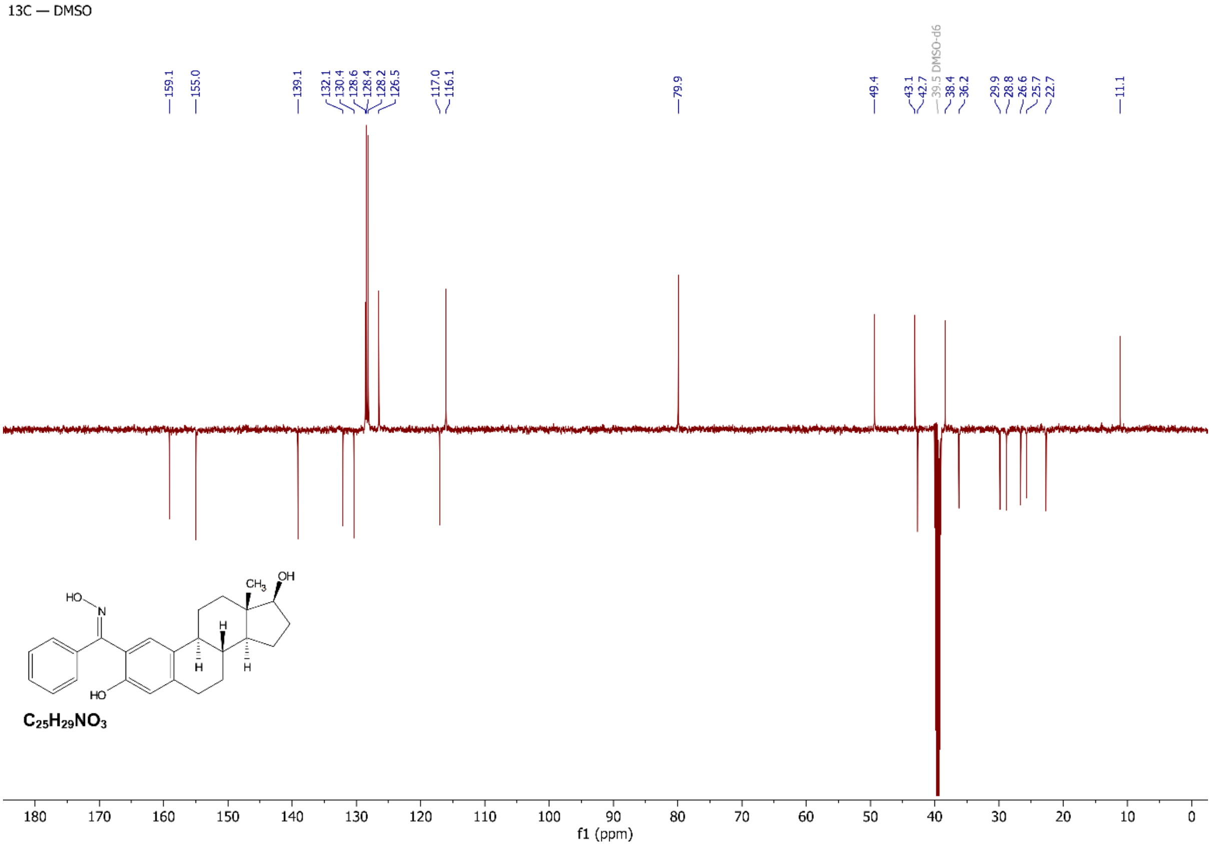

$^{1}$H NMR spectrum of compound **3e-***Z* (DMSO-*d*6, 500 MHz)

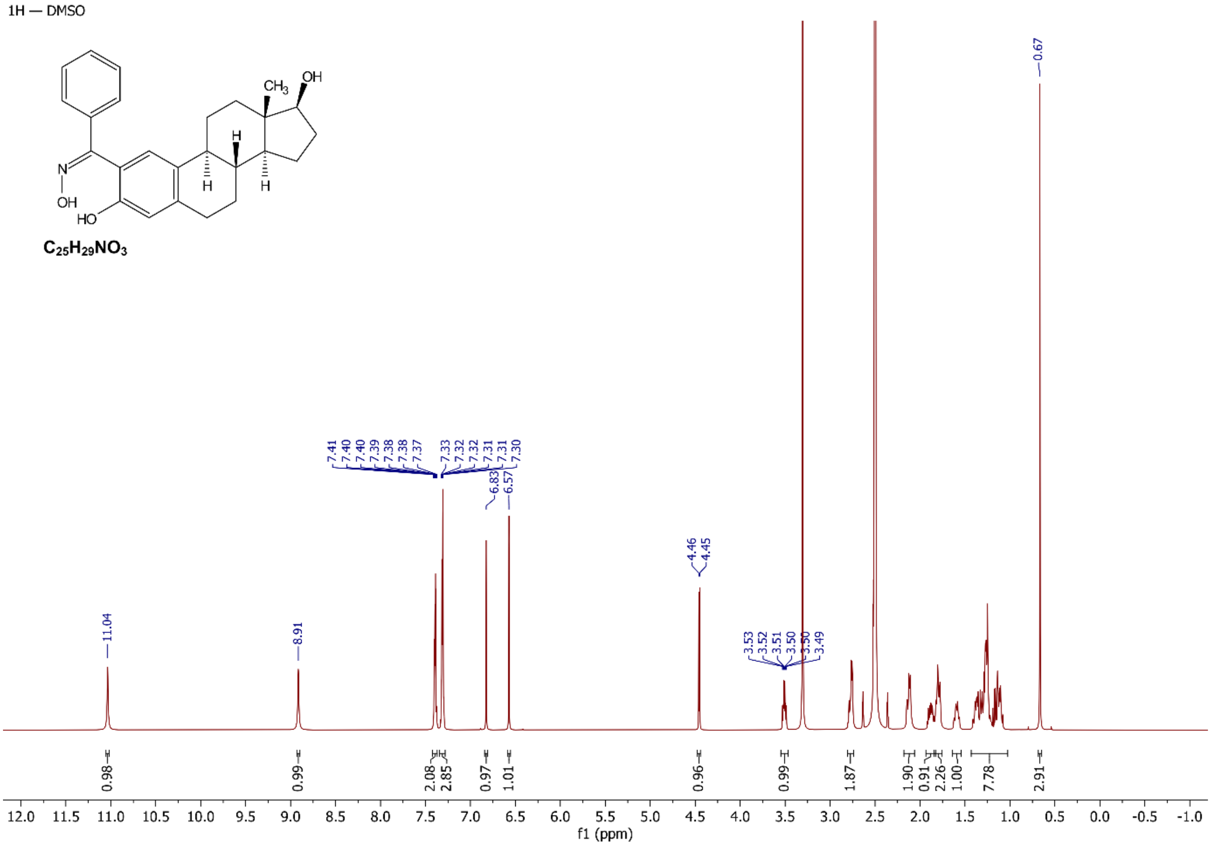


$^{13}$C NMR spectrum of compound **3e-***Z* (DMSO-*d*6, 125 MHz)

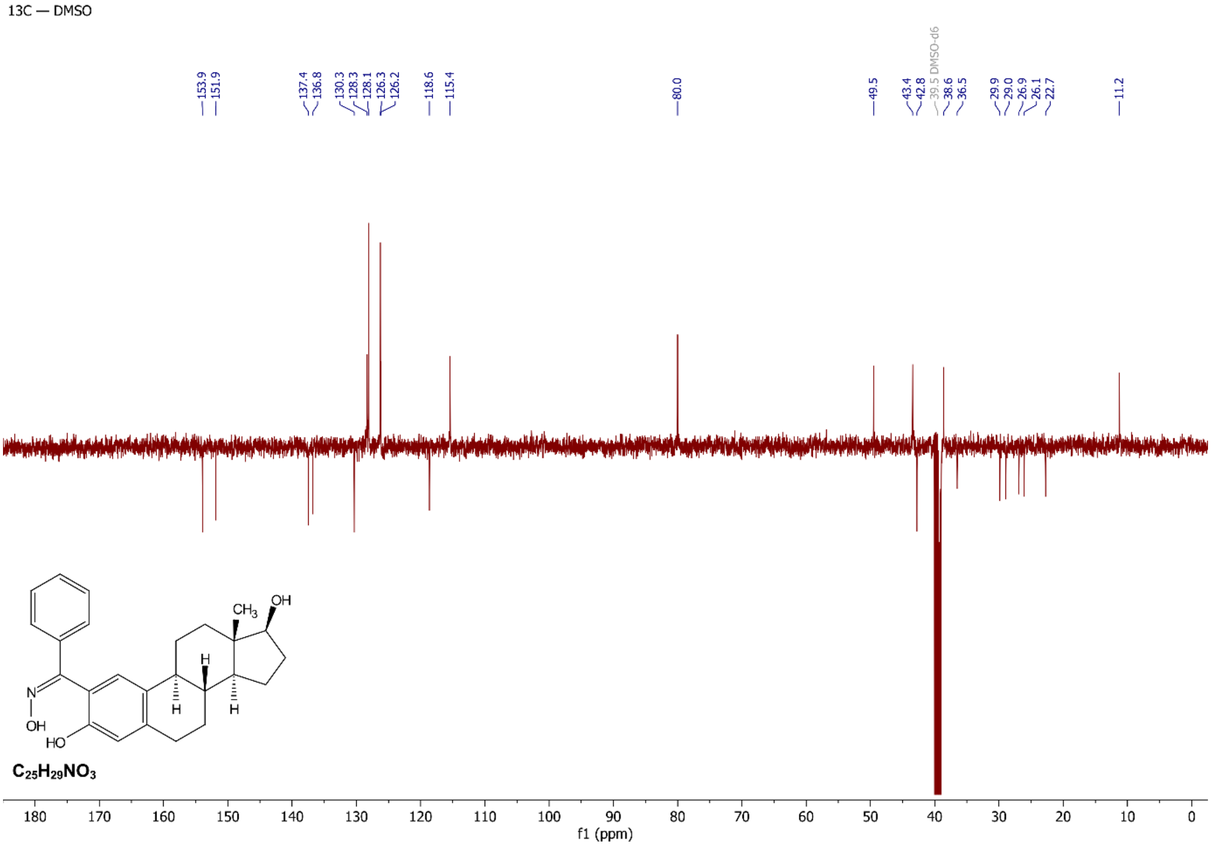

$^{1}$H NMR spectrum of compound **3f** (DMSO-$d_6$, 500 MHz)

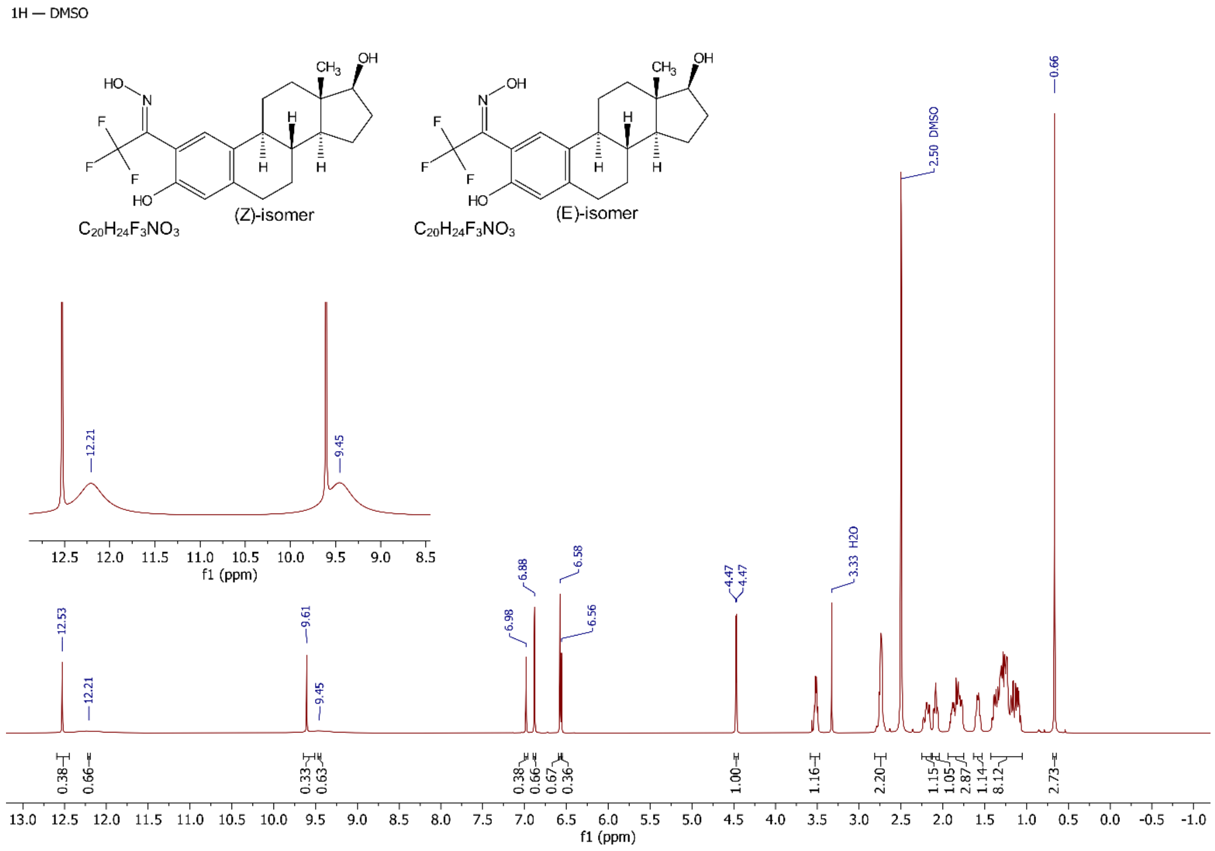


$^{13}$C NMR spectrum of compound **3f** (DMSO-$d_6$, 125 MHz)

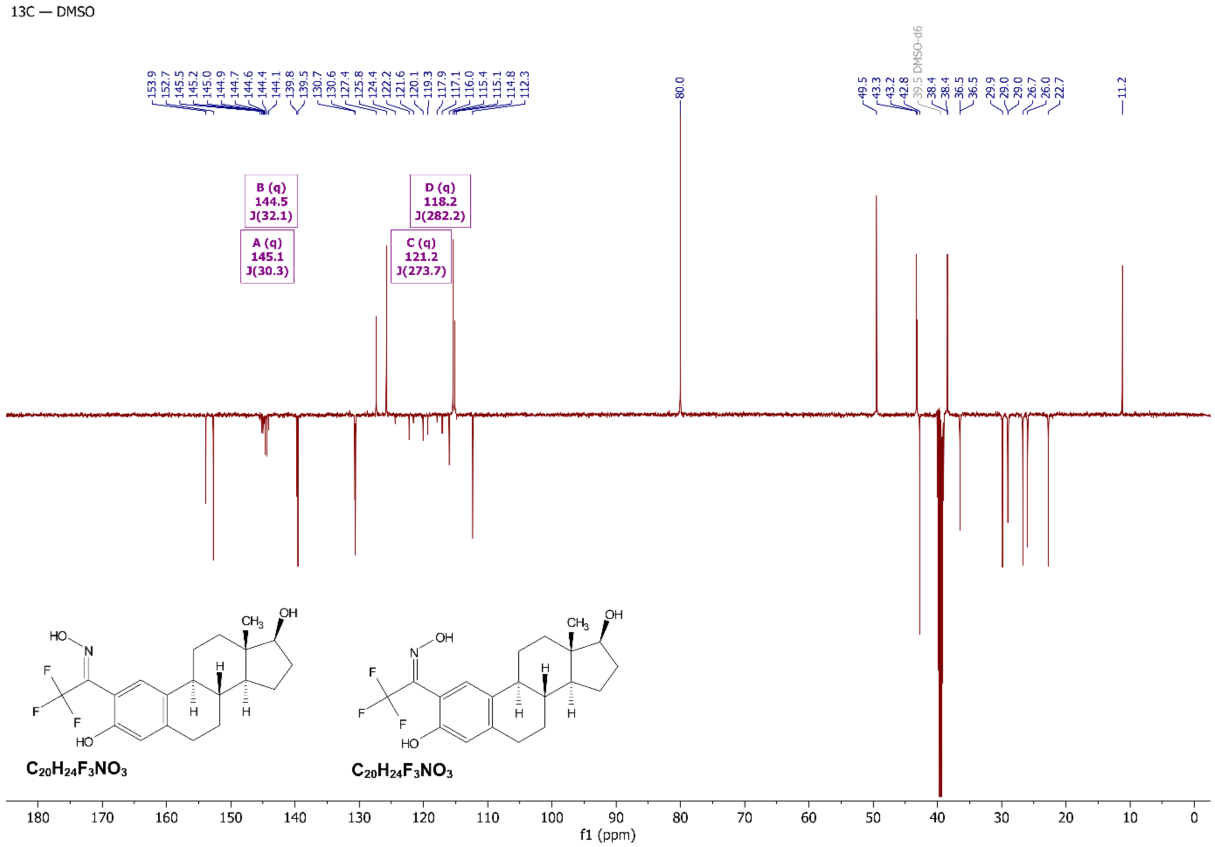

$^{1}$H NMR spectrum of compound **3g** (DMSO-$d_6$, 500 MHz)

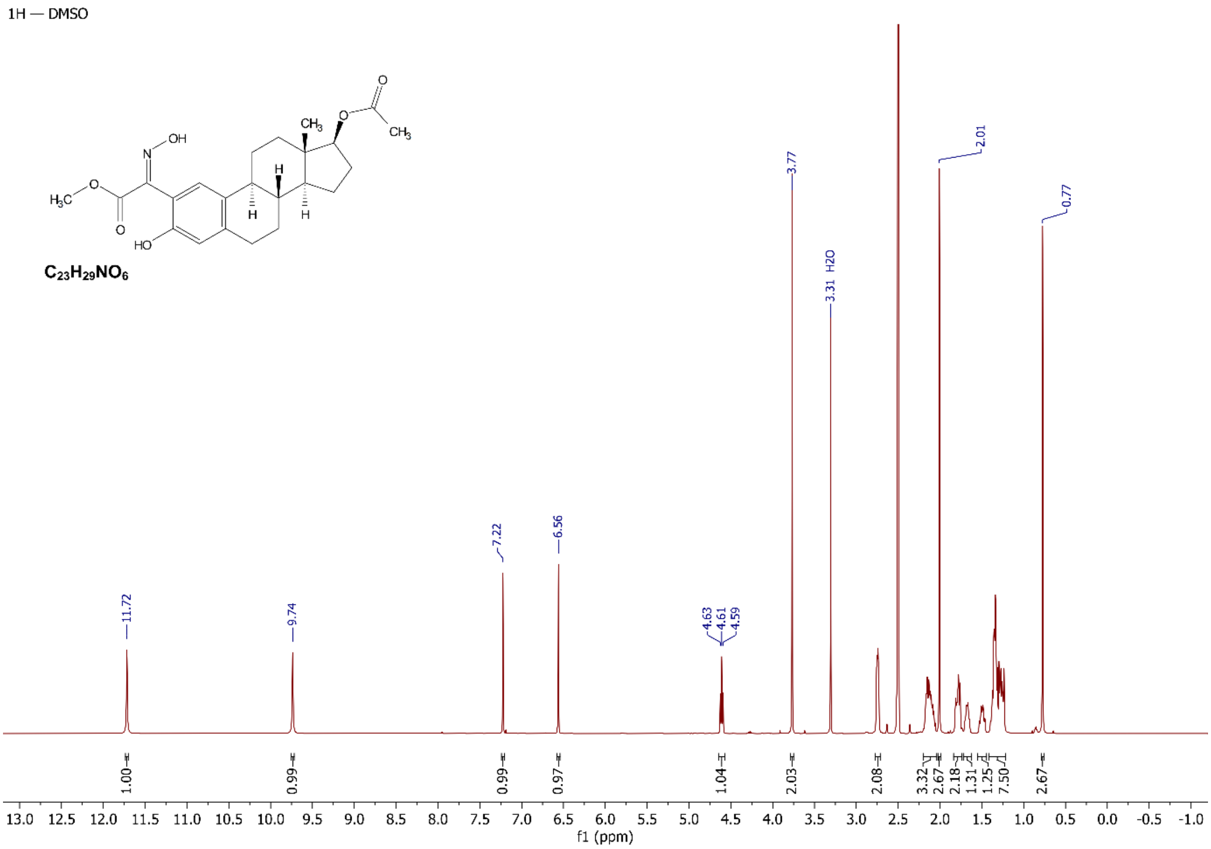


$^{13}$C NMR spectrum of compound **3g** (DMSO-$d_6$, 125 MHz)

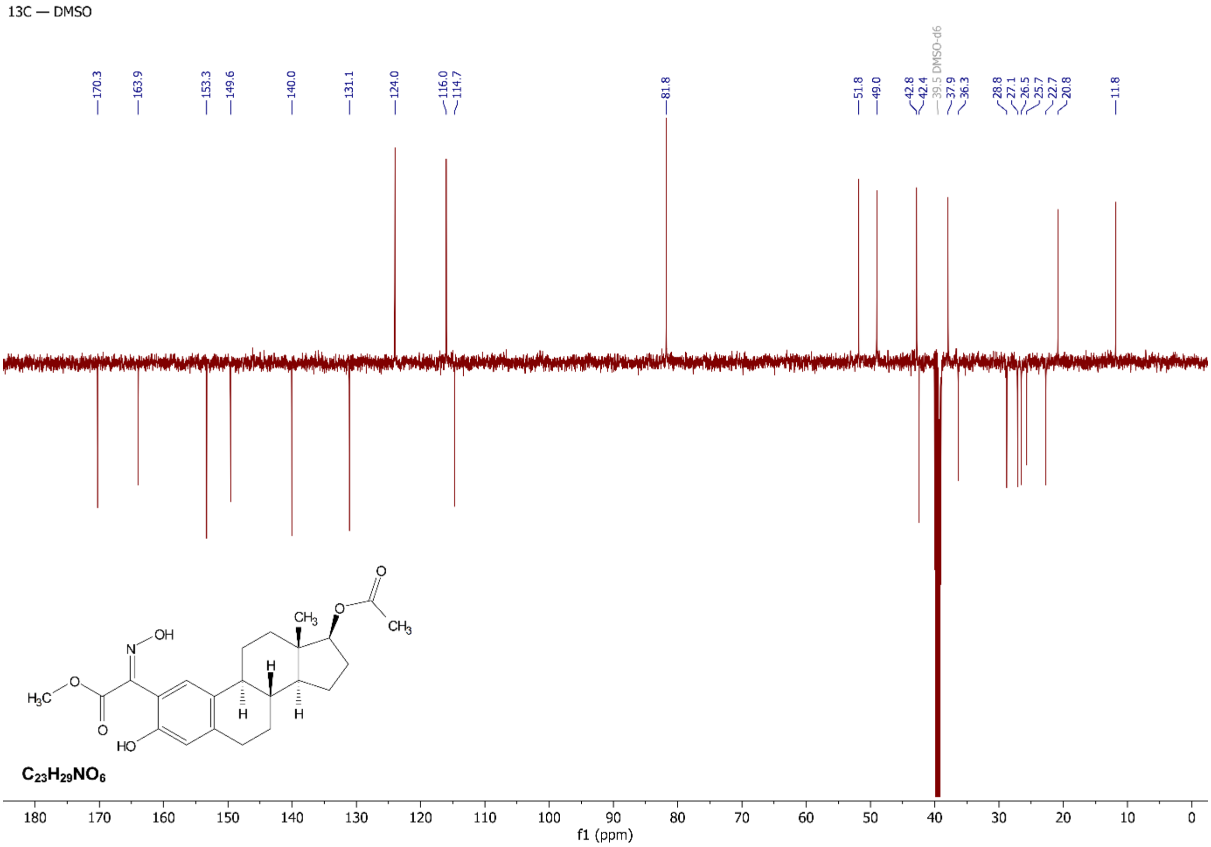

$^1H$ NMR spectrum of compound **4a** ($CDCl_3$, 500 MHz)

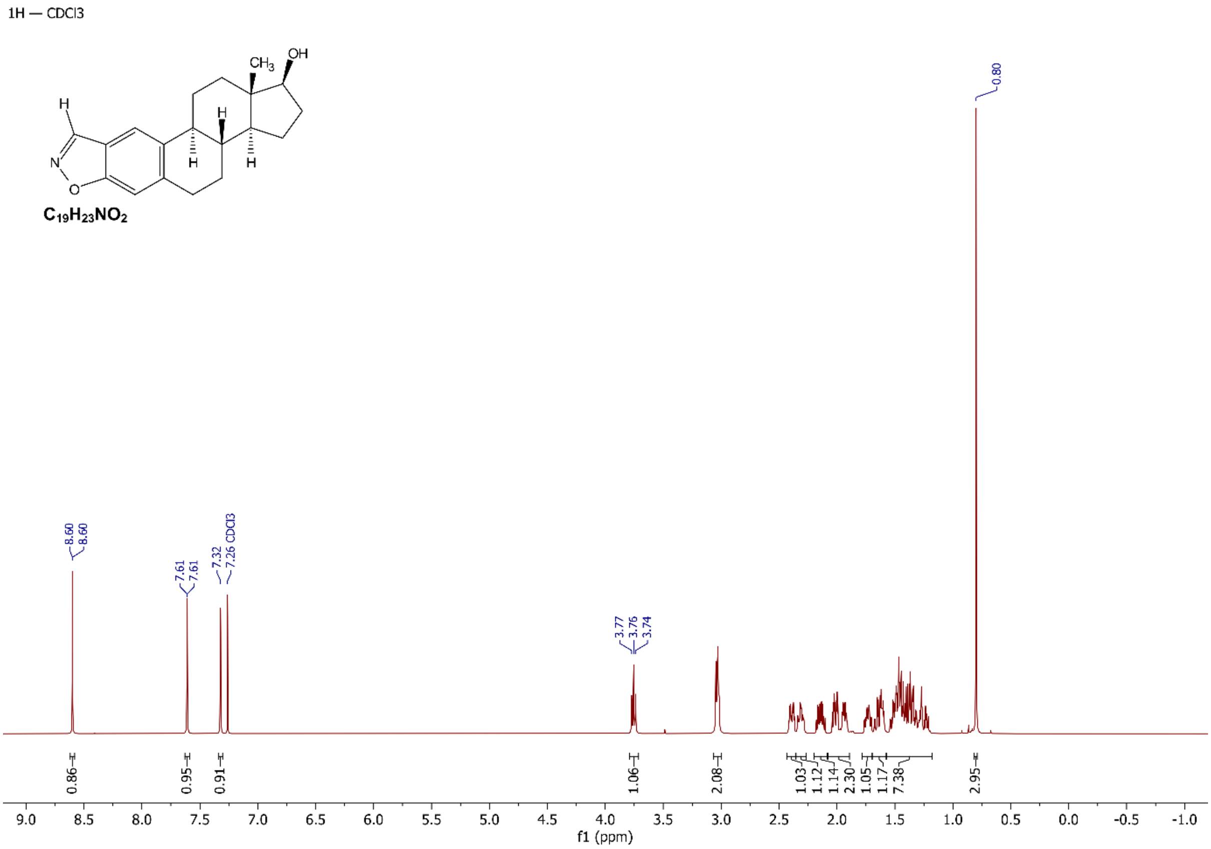


$^{13}C$ NMR spectrum of compound **4a** ($CDCl_3$, 125 MHz)

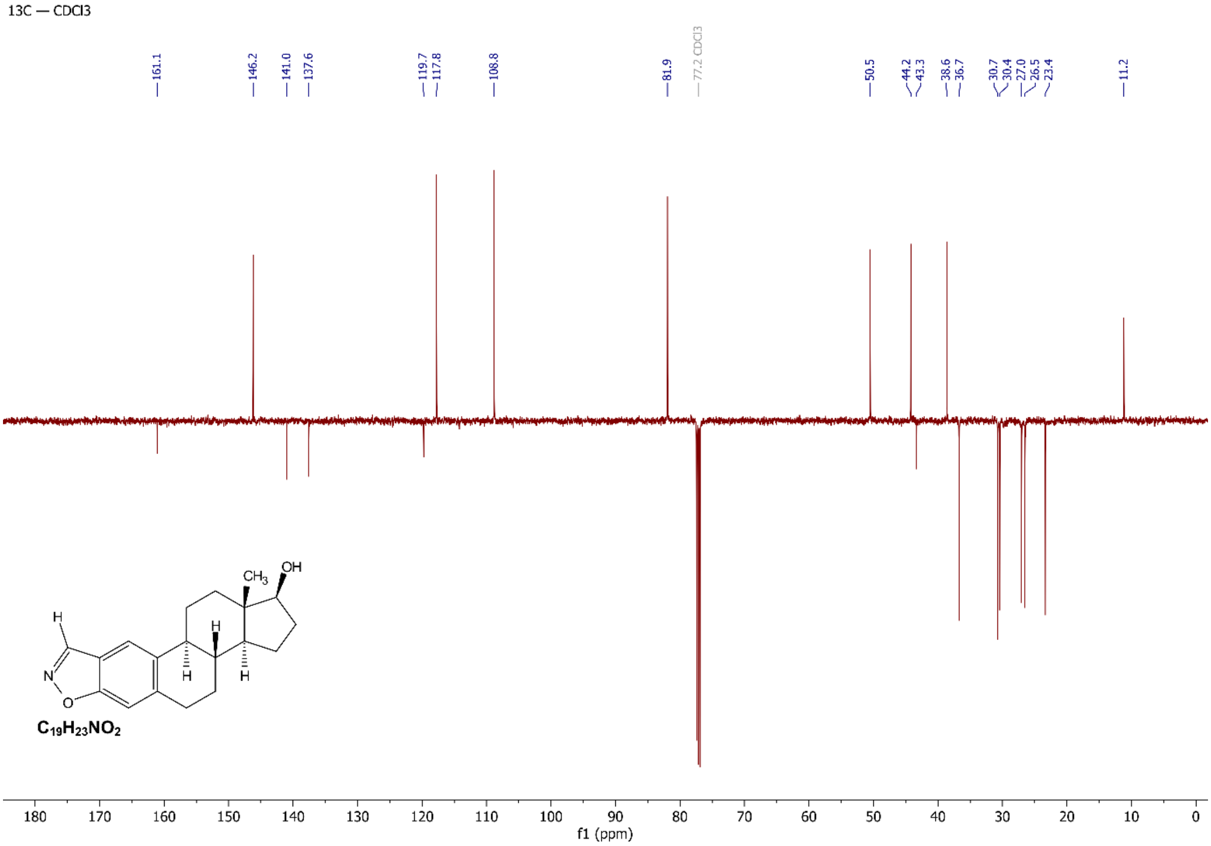

$^{1}$H NMR spectrum of compound **4b** ($CDCl_3$, 500 MHz)

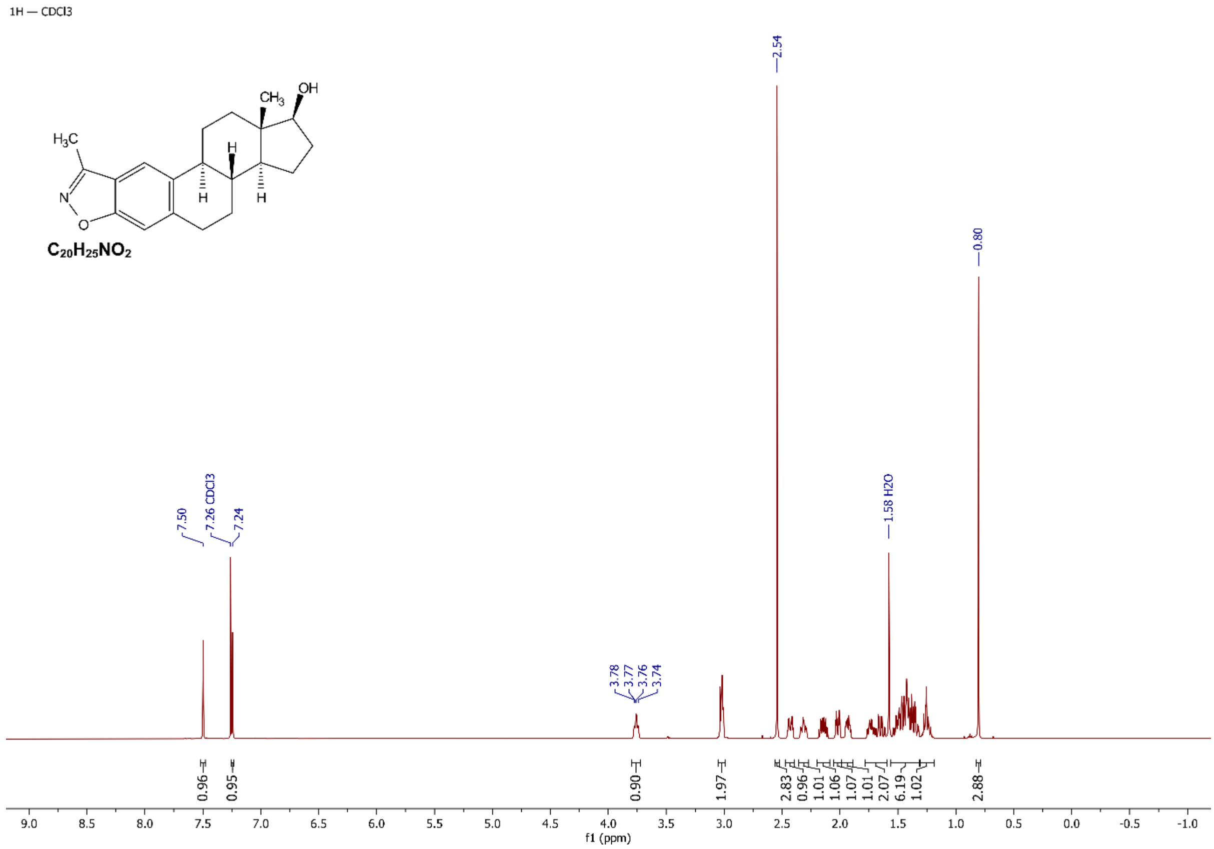


$^{13}$C NMR spectrum of compound **4b** ($CDCl_3$, 125 MHz)

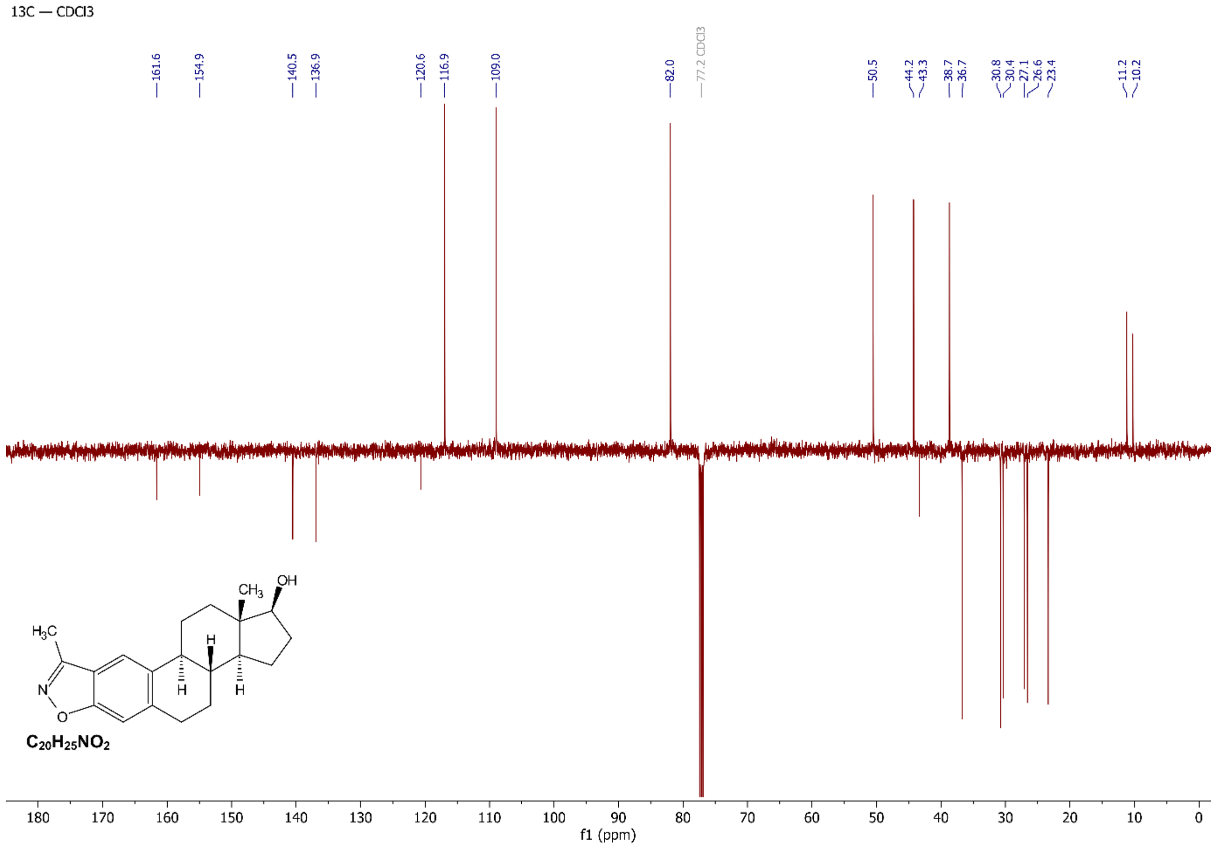

$^{1}$H NMR spectrum of compound **4c** ($CDCl_3$, 500 MHz)

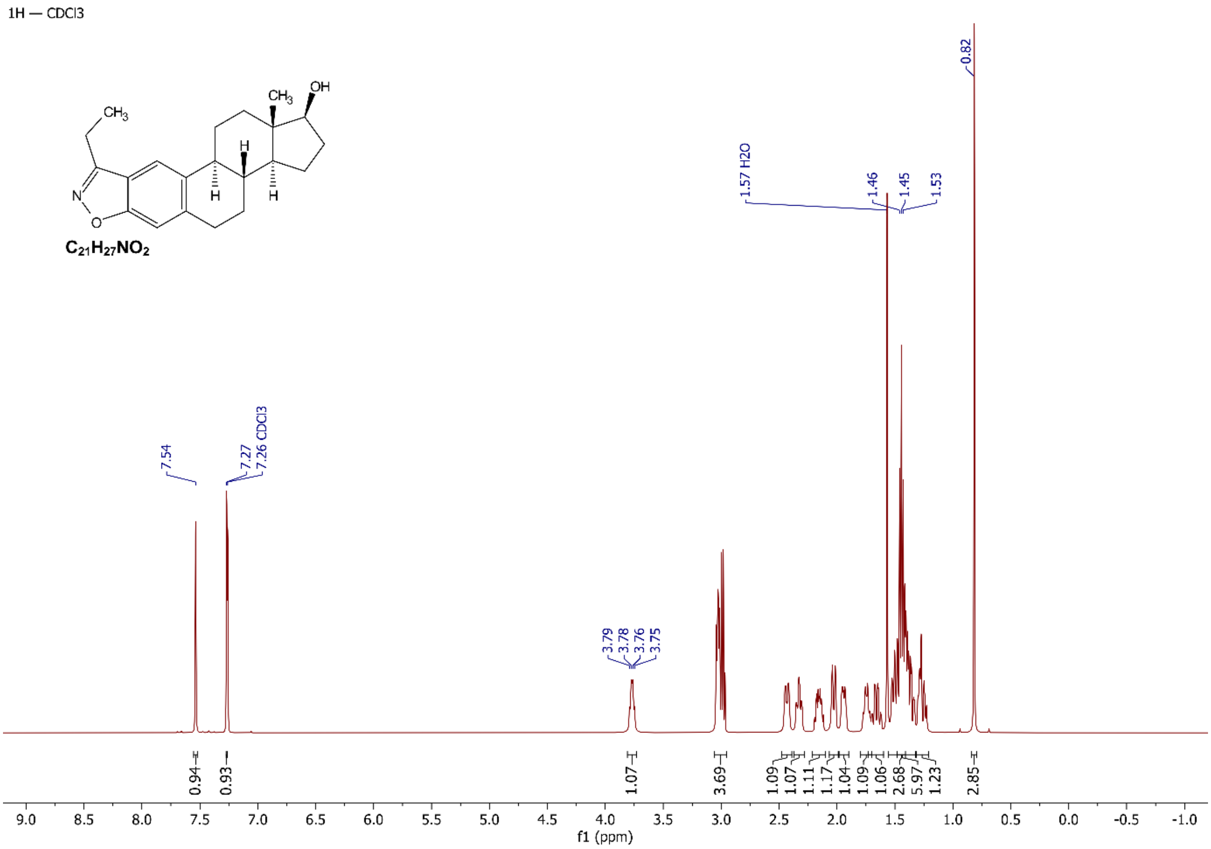


$^{13}$C NMR spectrum of compound **4c** ($CDCl_3$, 125 MHz)

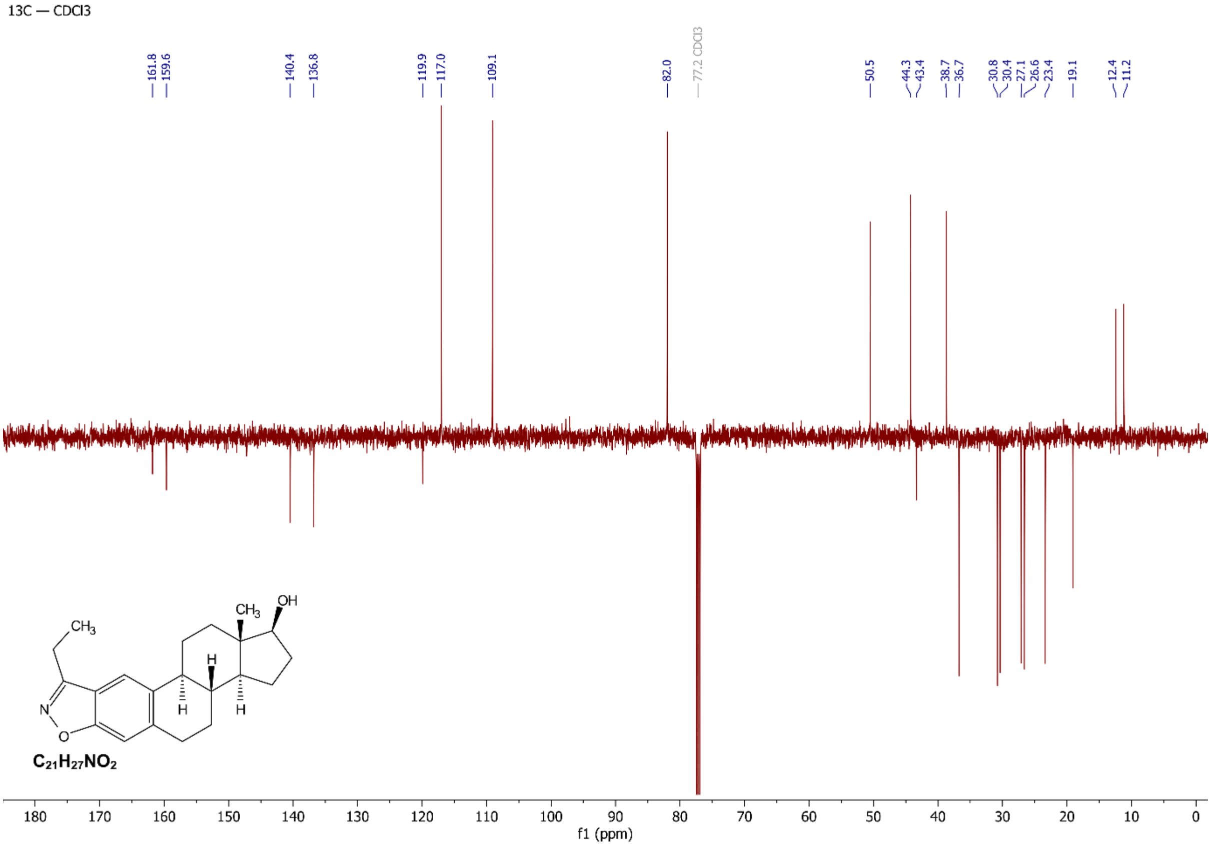

$^{1}H$ NMR spectrum of compound **4d** ($CDCl_3$, 500 MHz)

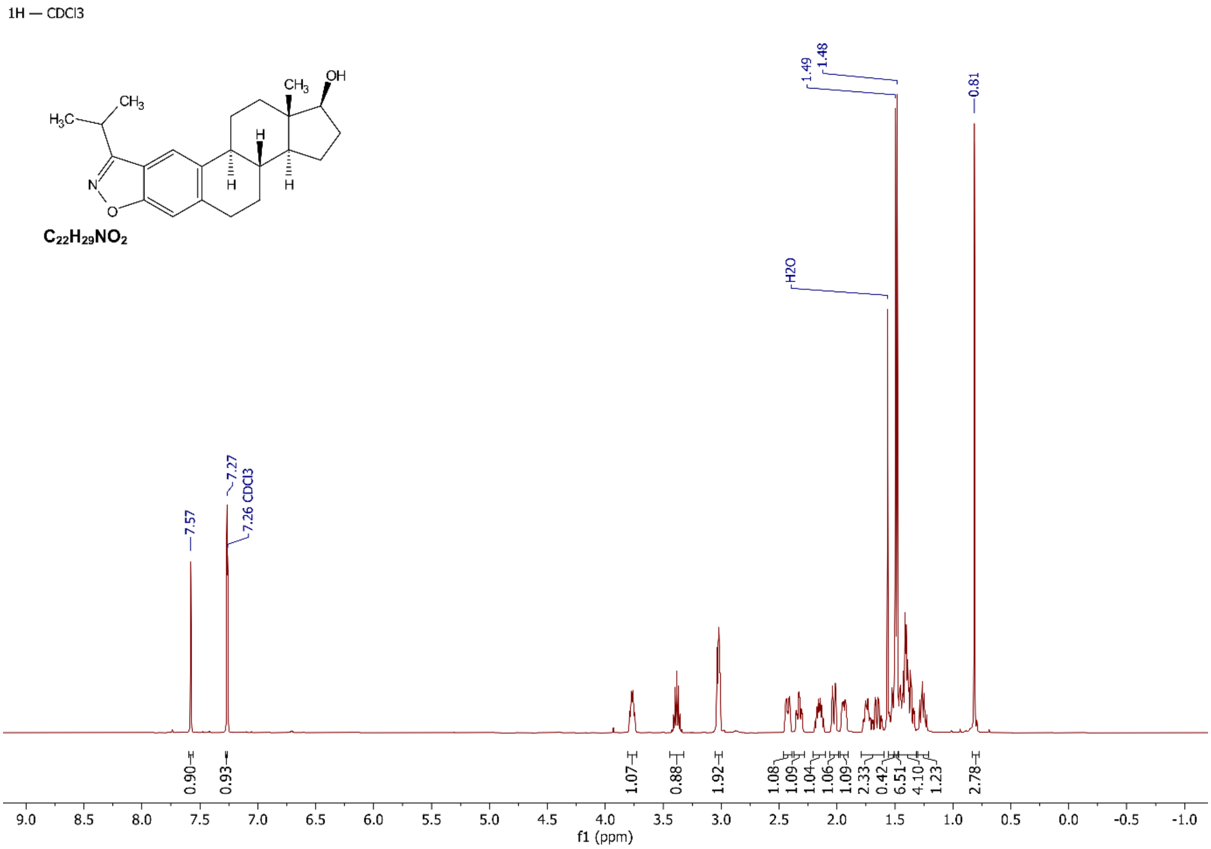


$^{13}C$ NMR spectrum of compound **4d** ($CDCl_3$, 125 MHz)

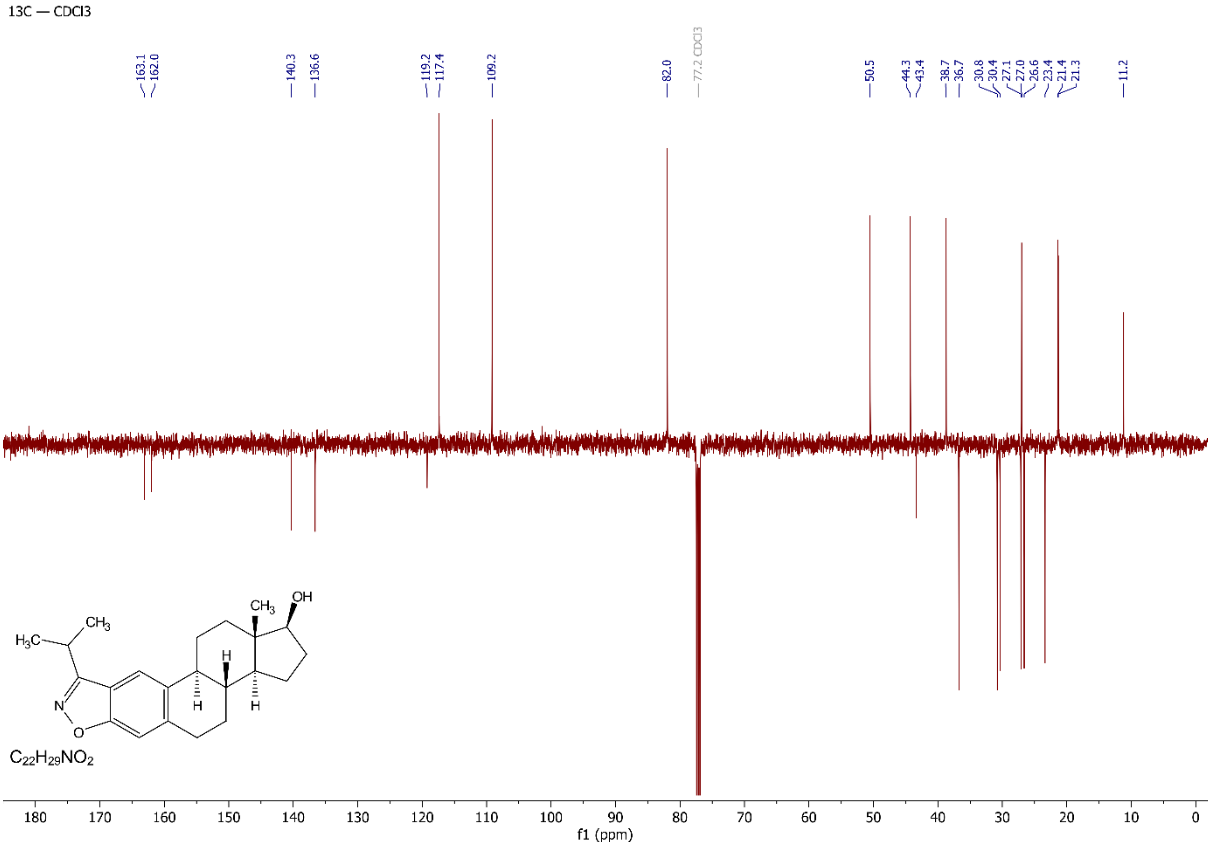

$^1H$ NMR spectrum of compound **4e** ($CDCl_3$, 500 MHz)

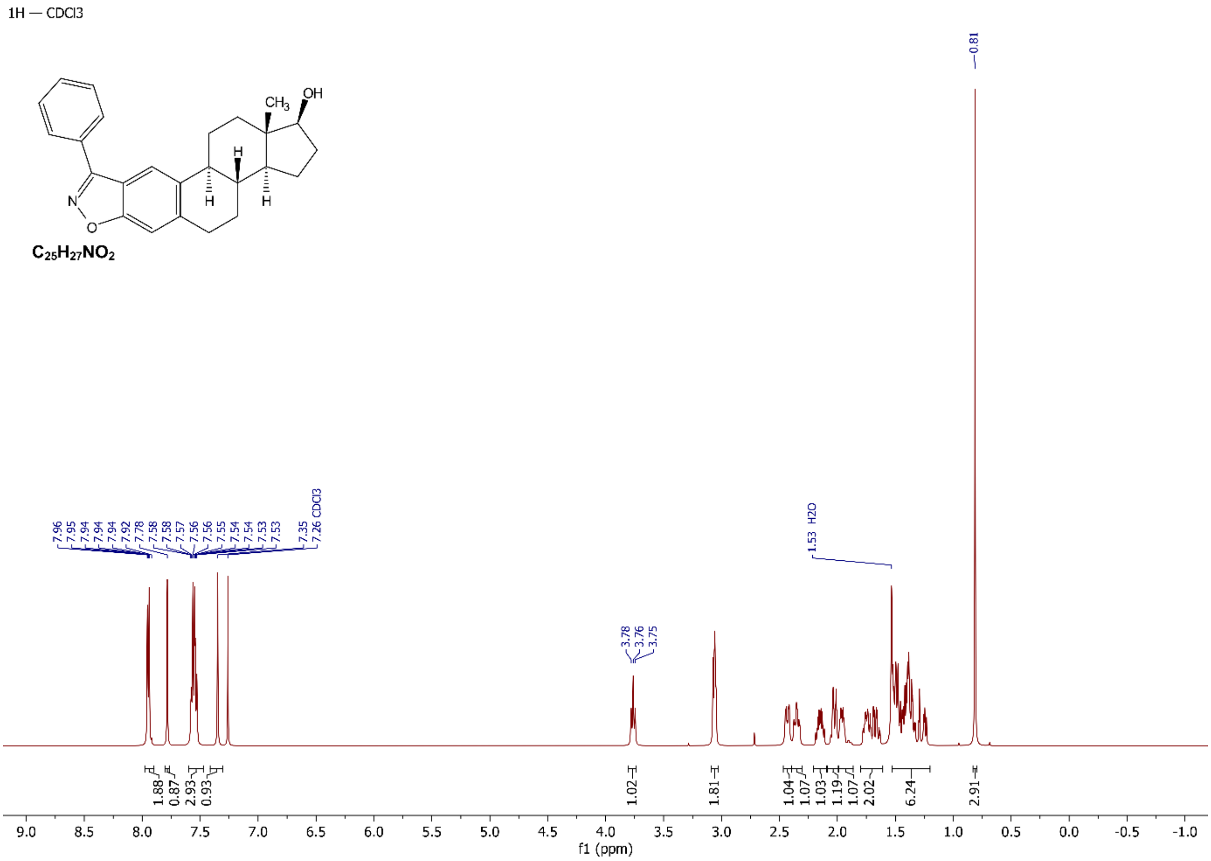


$^{13}C$ NMR spectrum of compound **4e** ($CDCl_3$, 125 MHz)

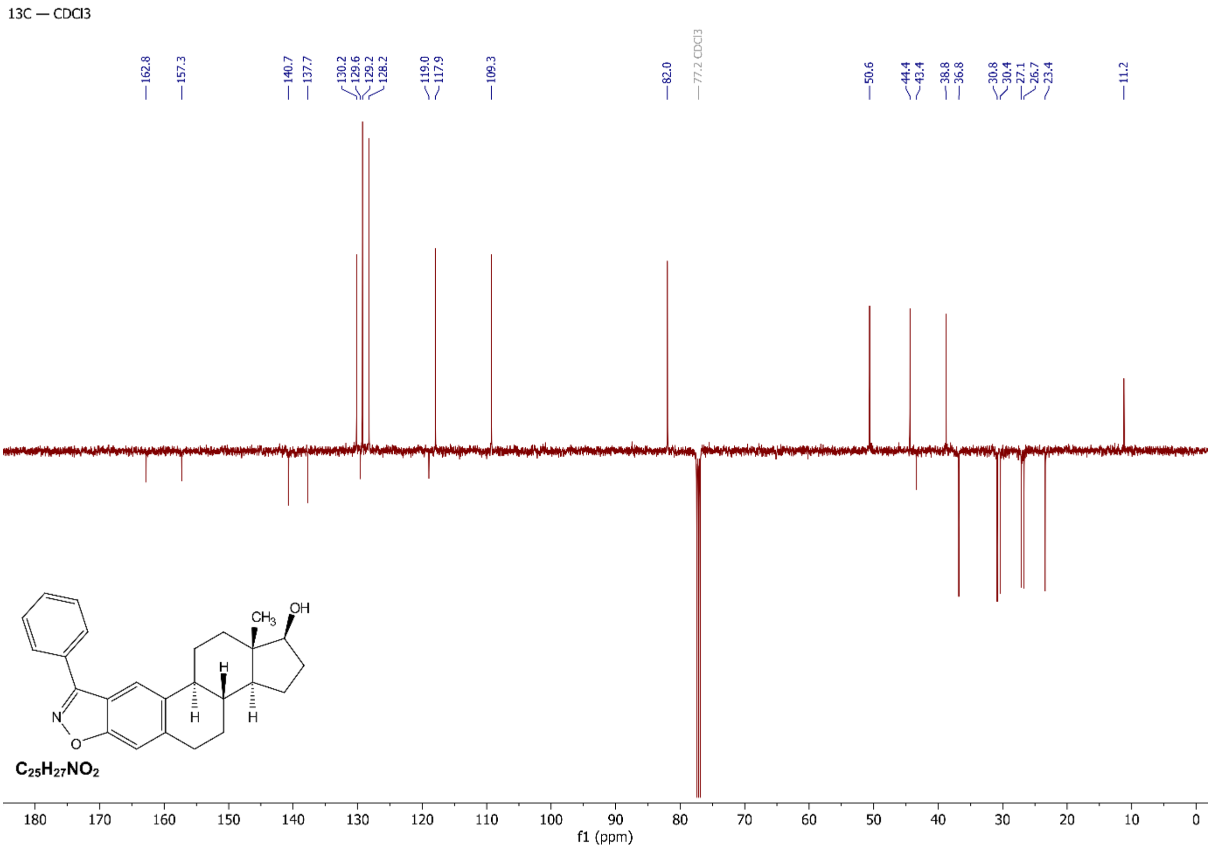

## $^{1}$H NMR spectrum of compound **4f** ($CDCl_3$, 500 MHz)

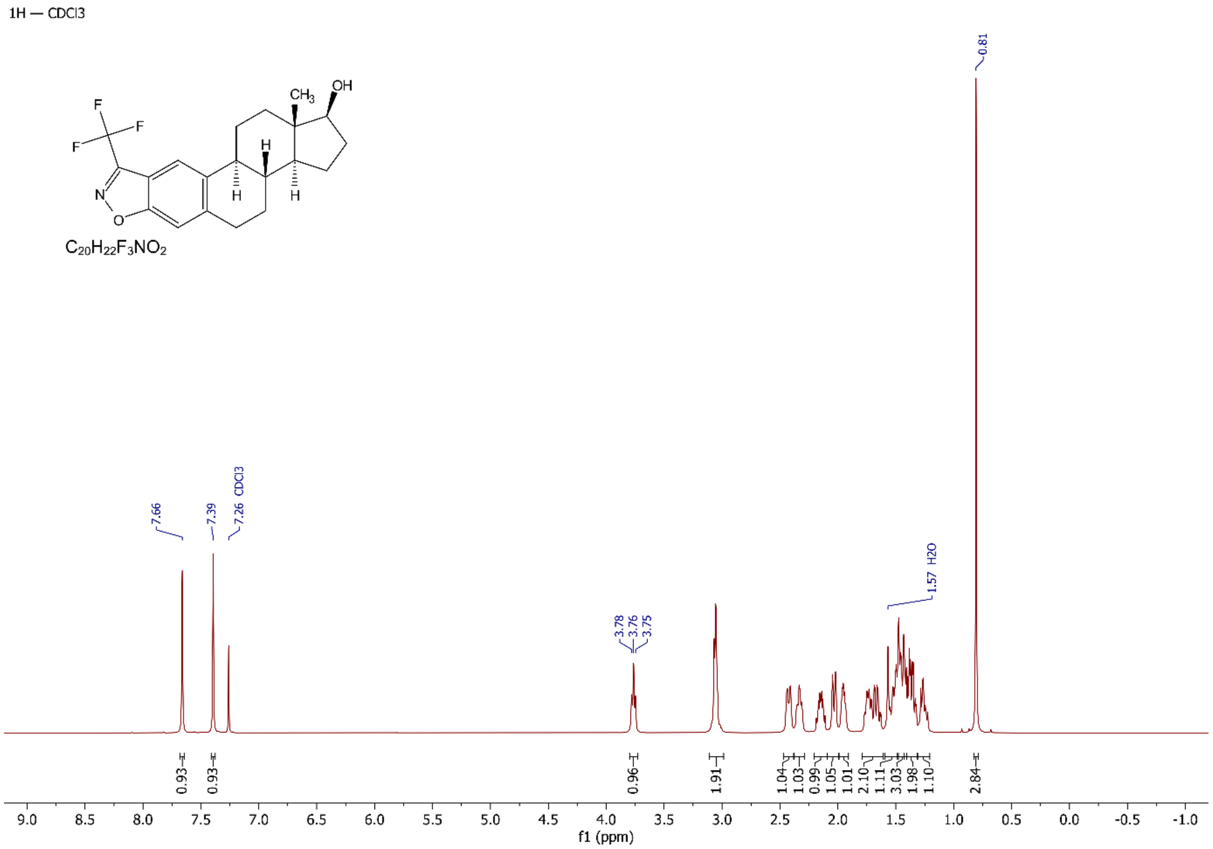


## $^{13}$C NMR spectrum of compound **4f** ($CDCl_3$, 125 MHz)

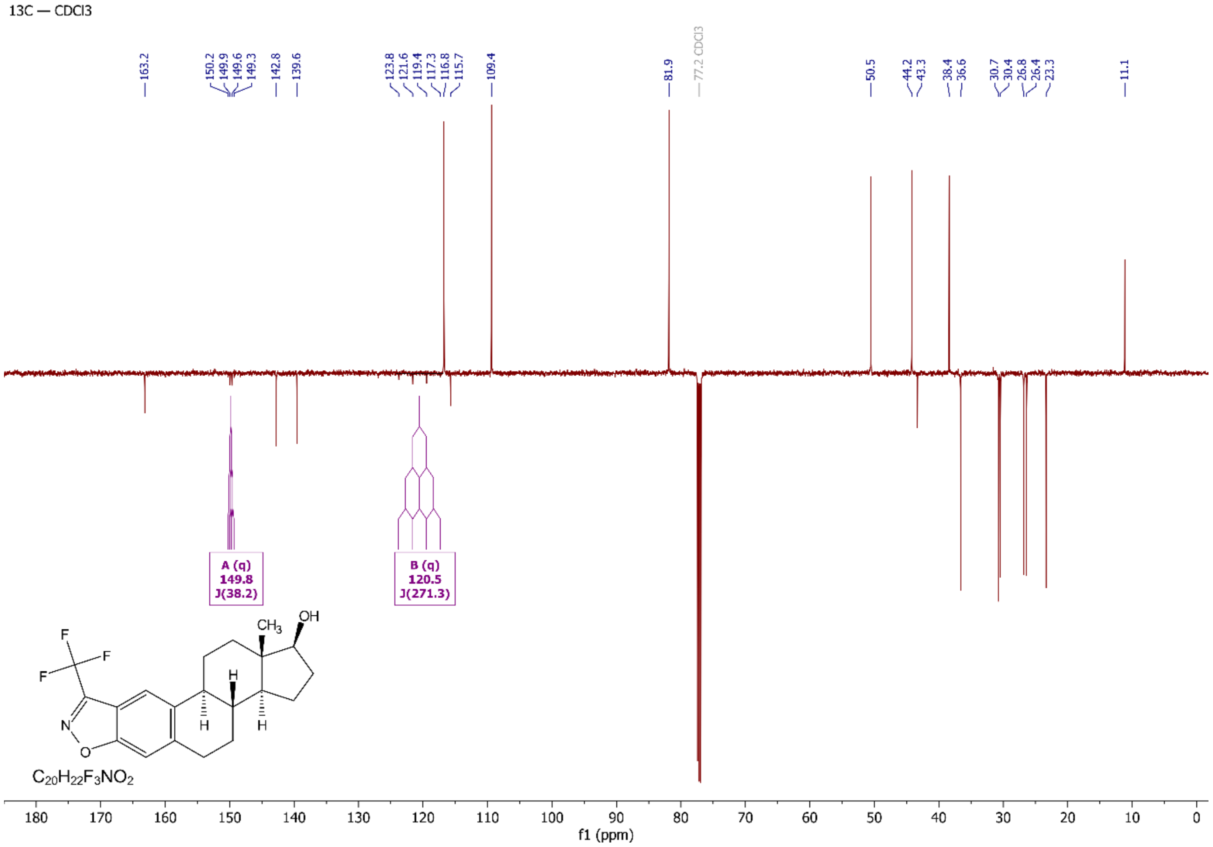

$^{1}$H NMR spectrum of compound **4g** ($CDCl_3$, 500 MHz)

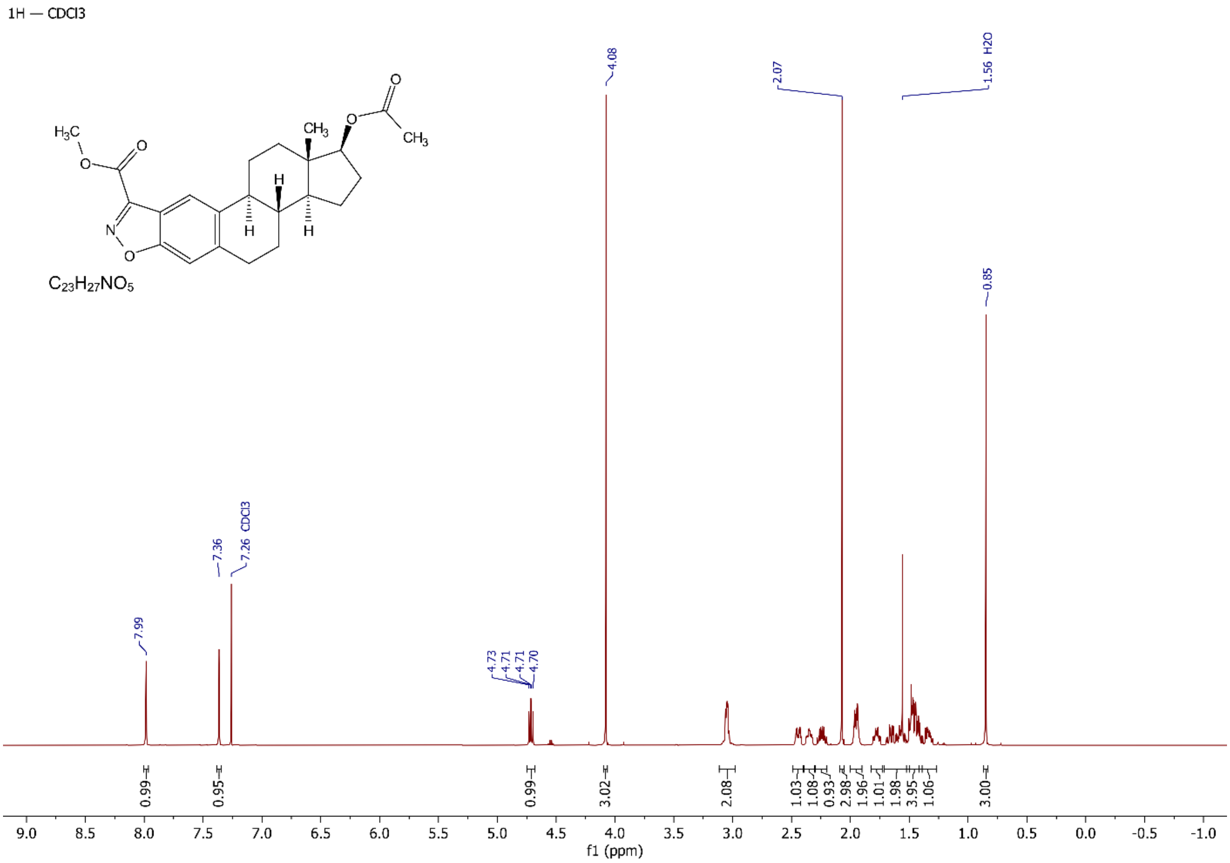


$^{13}$C NMR spectrum of compound **4g** ($CDCl_3$, 125 MHz)

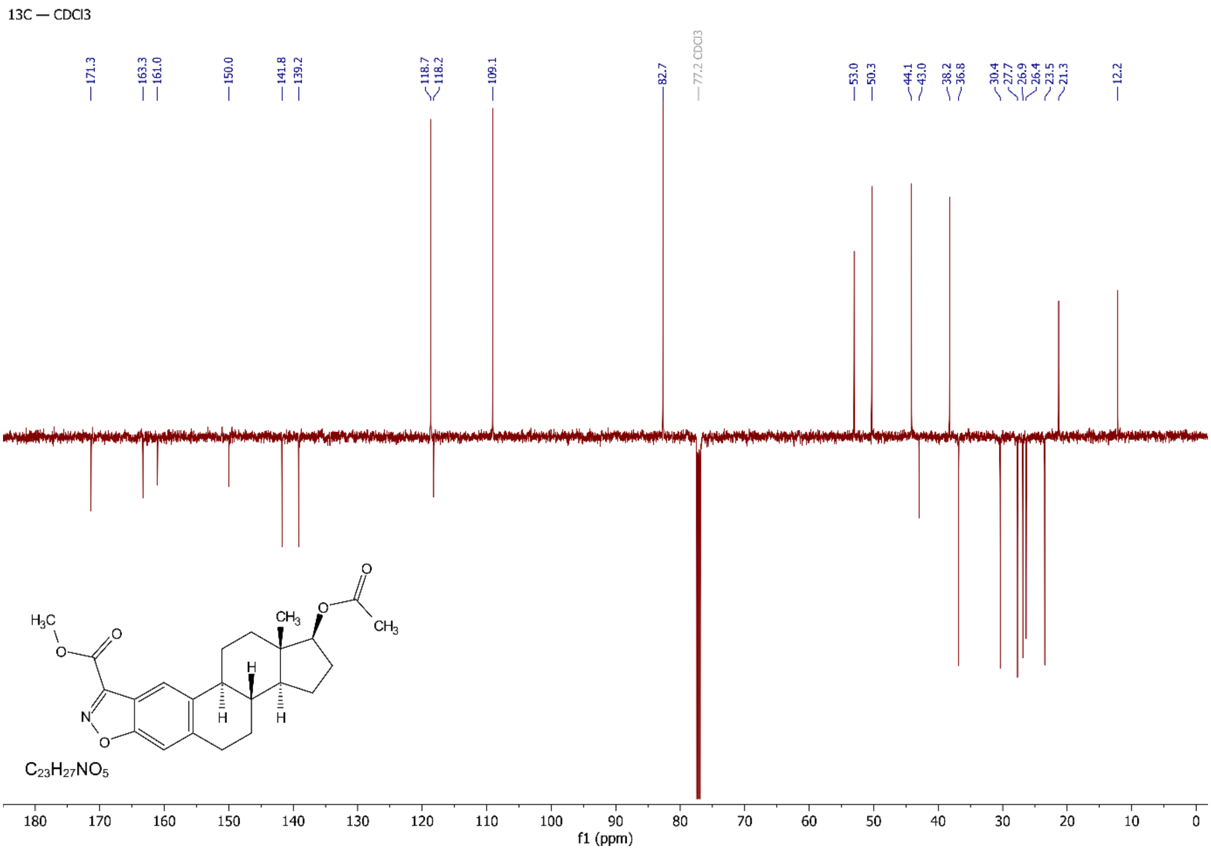

$^{1}$H NMR spectrum of compound **6a** ($CDCl_3$, 500 MHz)

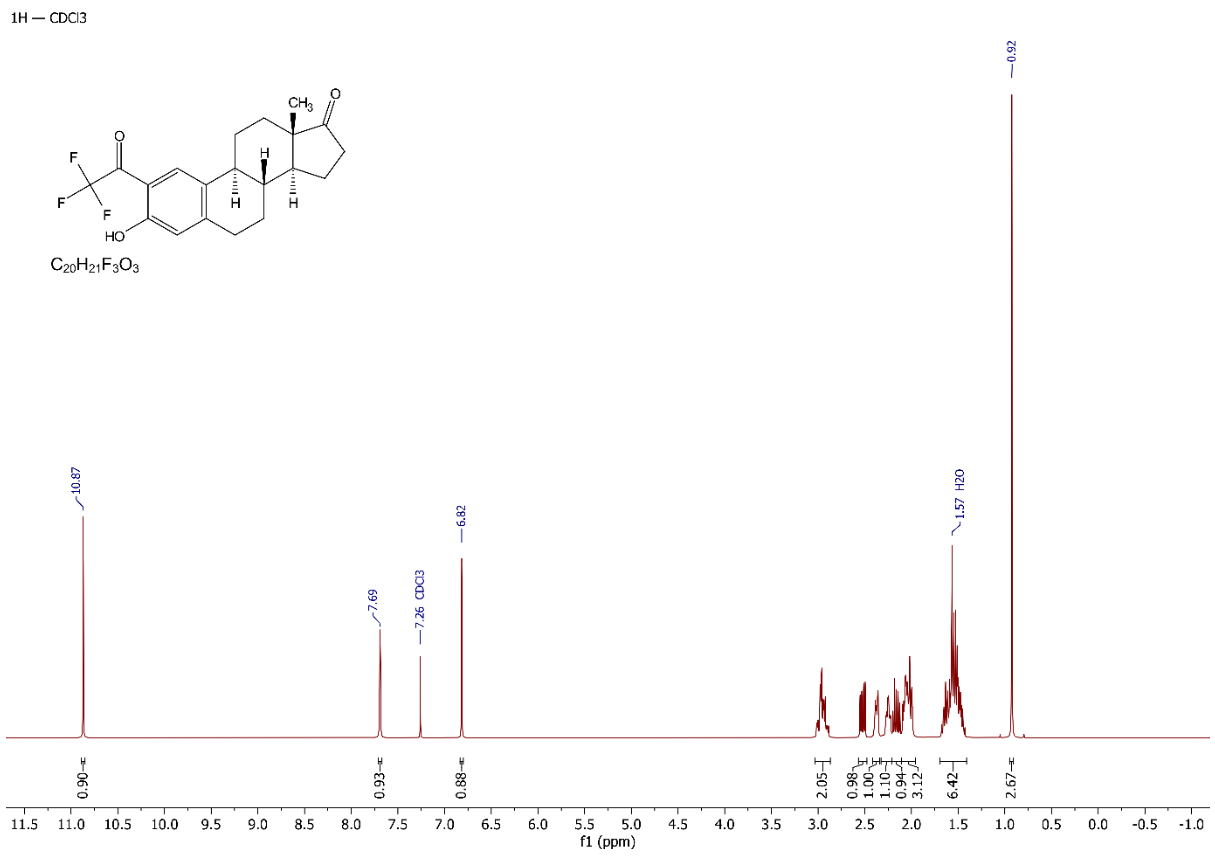


$^{13}$C NMR spectrum of compound **6a** ($CDCl_3$, 125 MHz)

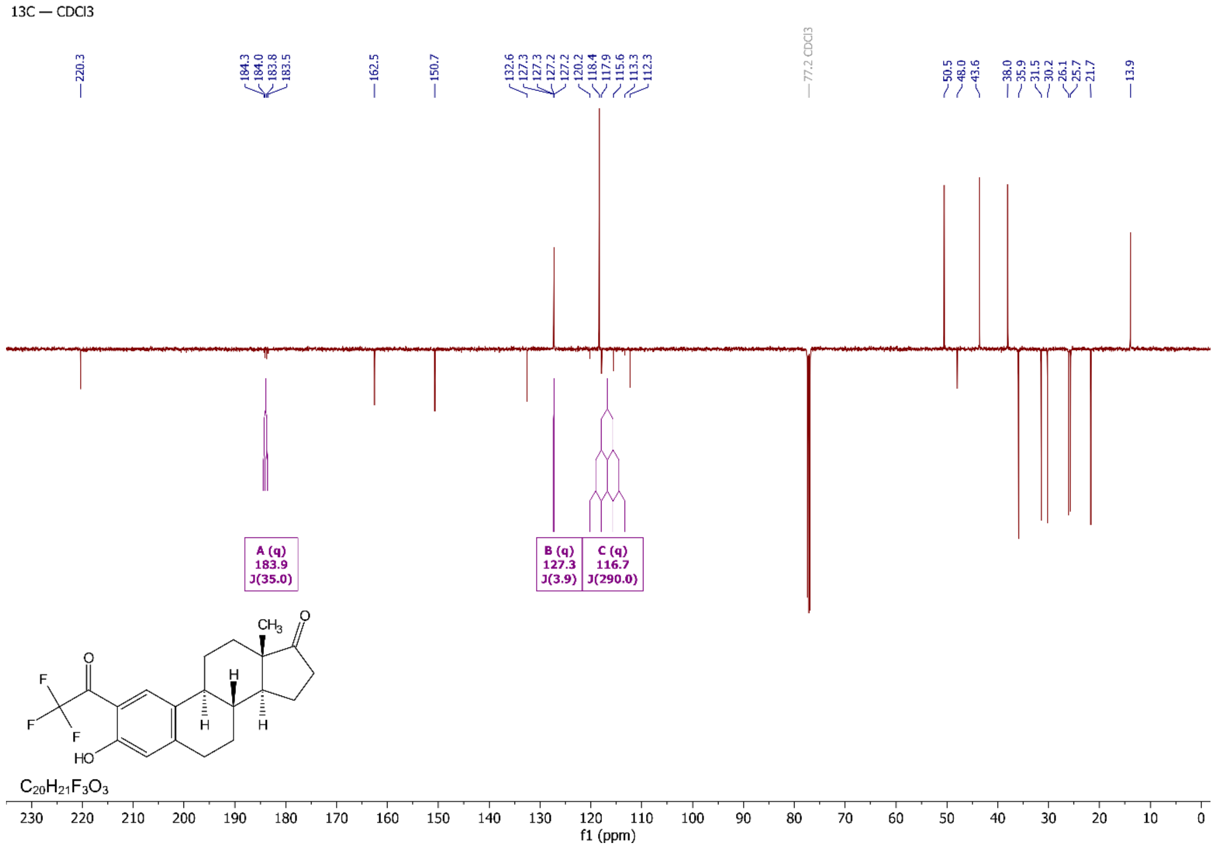

$^1H$ NMR spectrum of compound **6b** (DMSO-*d*$_6$, 500 MHz)

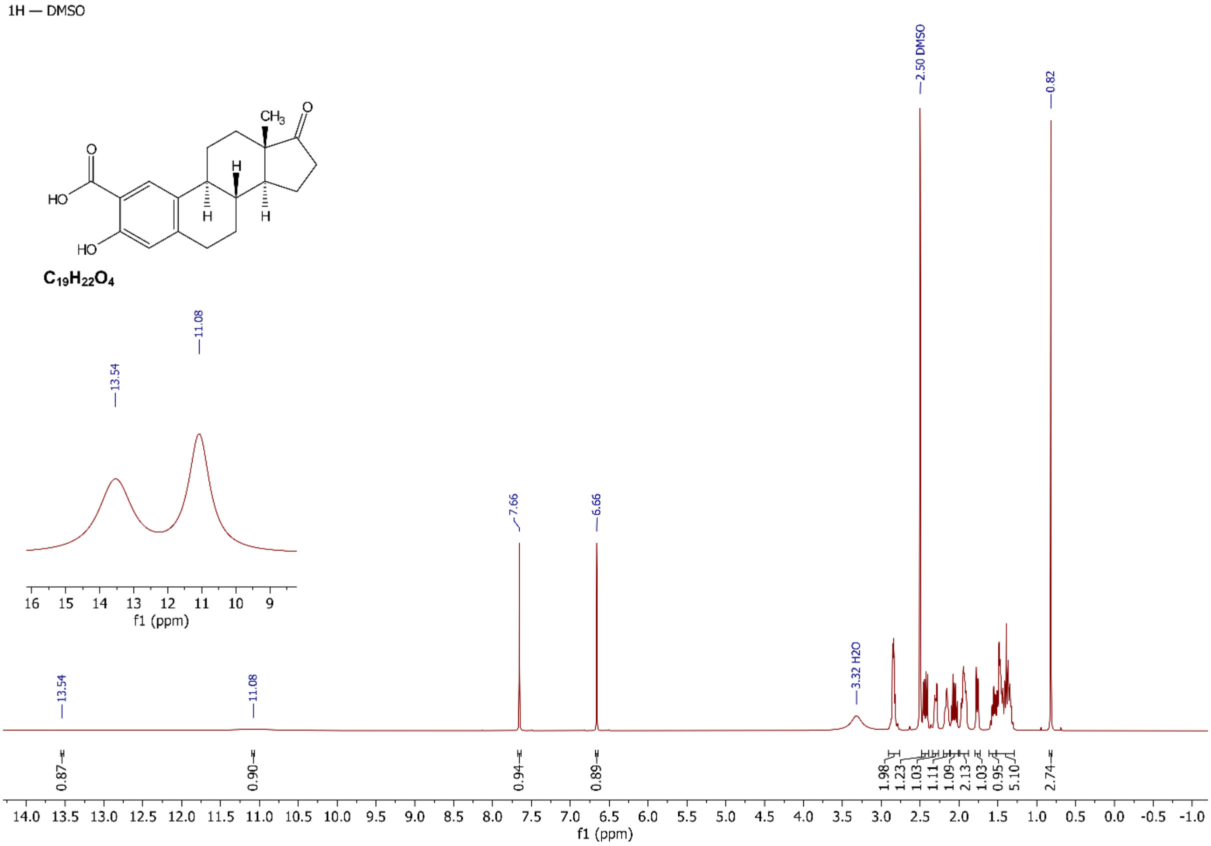


$^{13}C$ NMR spectrum of compound **6b** (DMSO-*d*$_6$, 125 MHz)

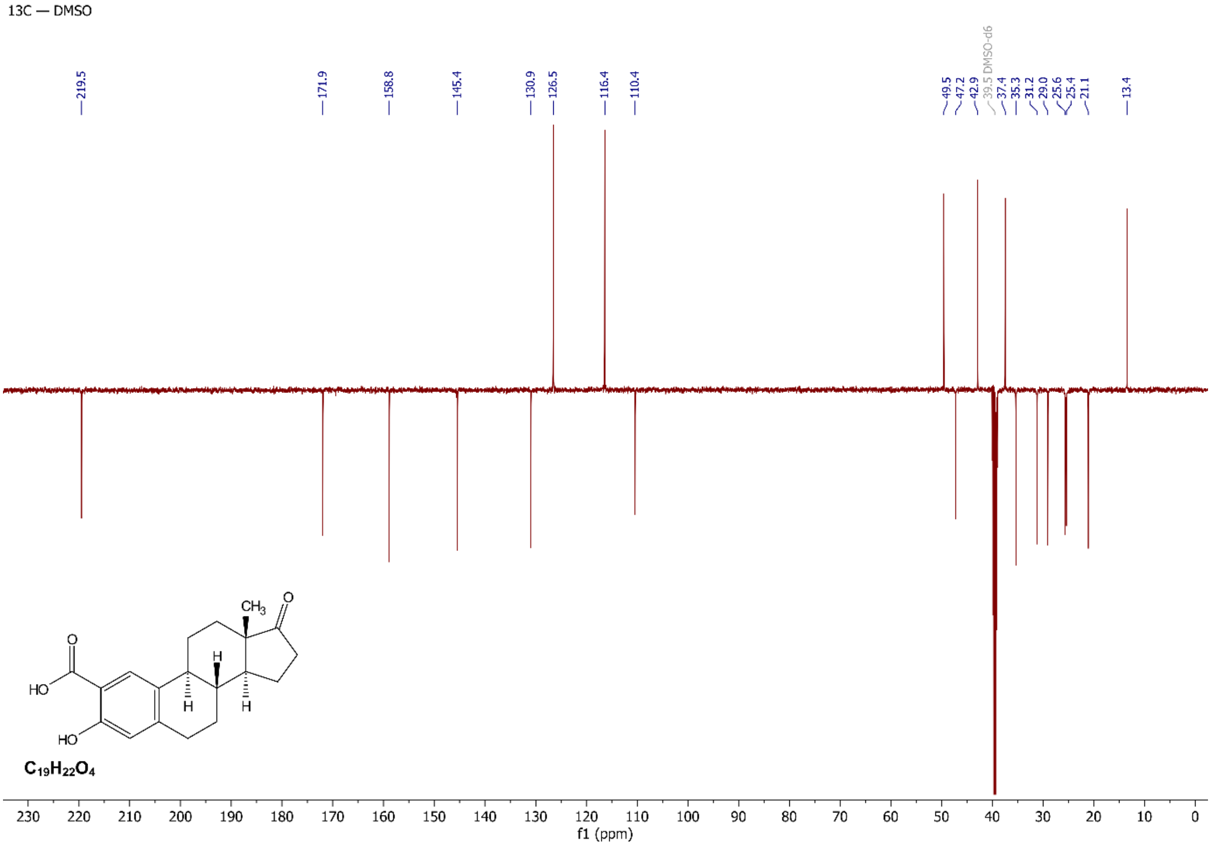

$^{1}$H NMR spectrum of compound **6c** (DMSO-*d*6, 500 MHz)

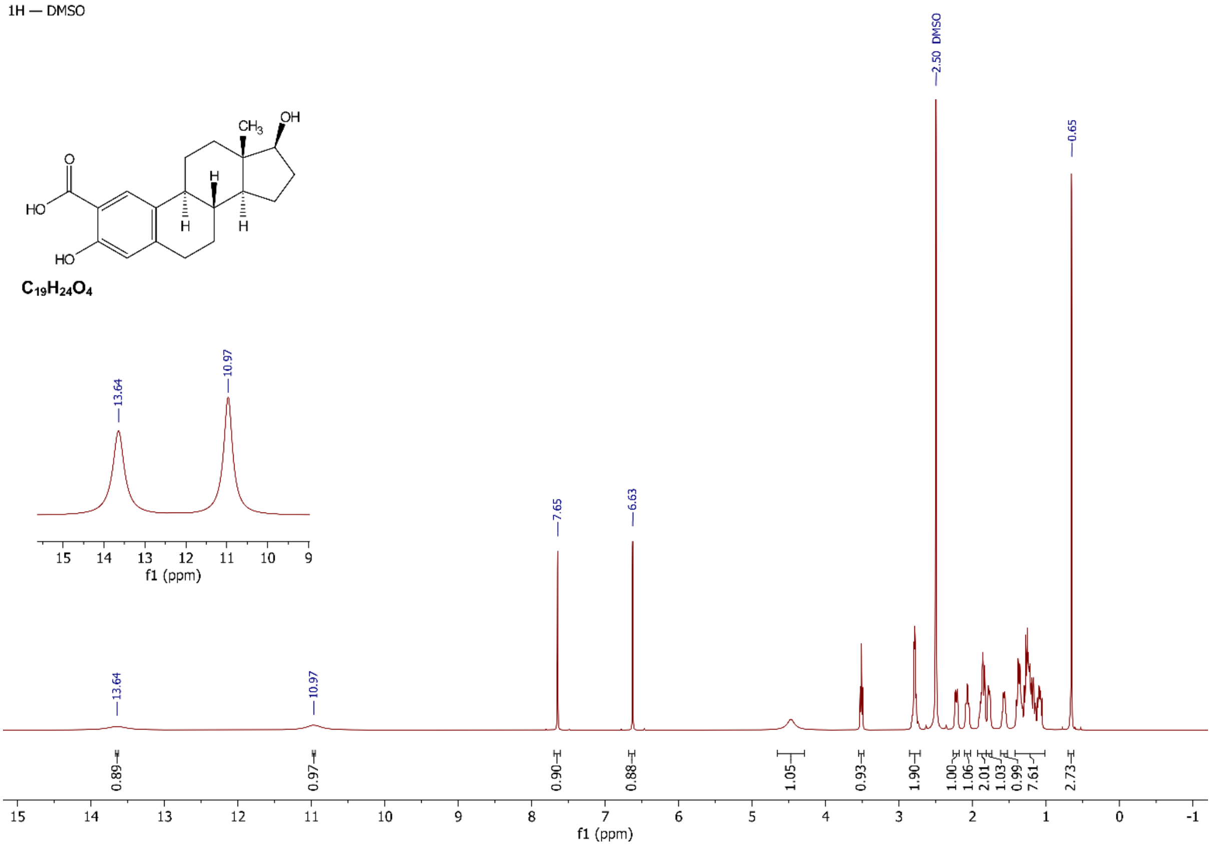


$^{13}$C NMR spectrum of compound **6c** (DMSO-*d*6, 125 MHz)

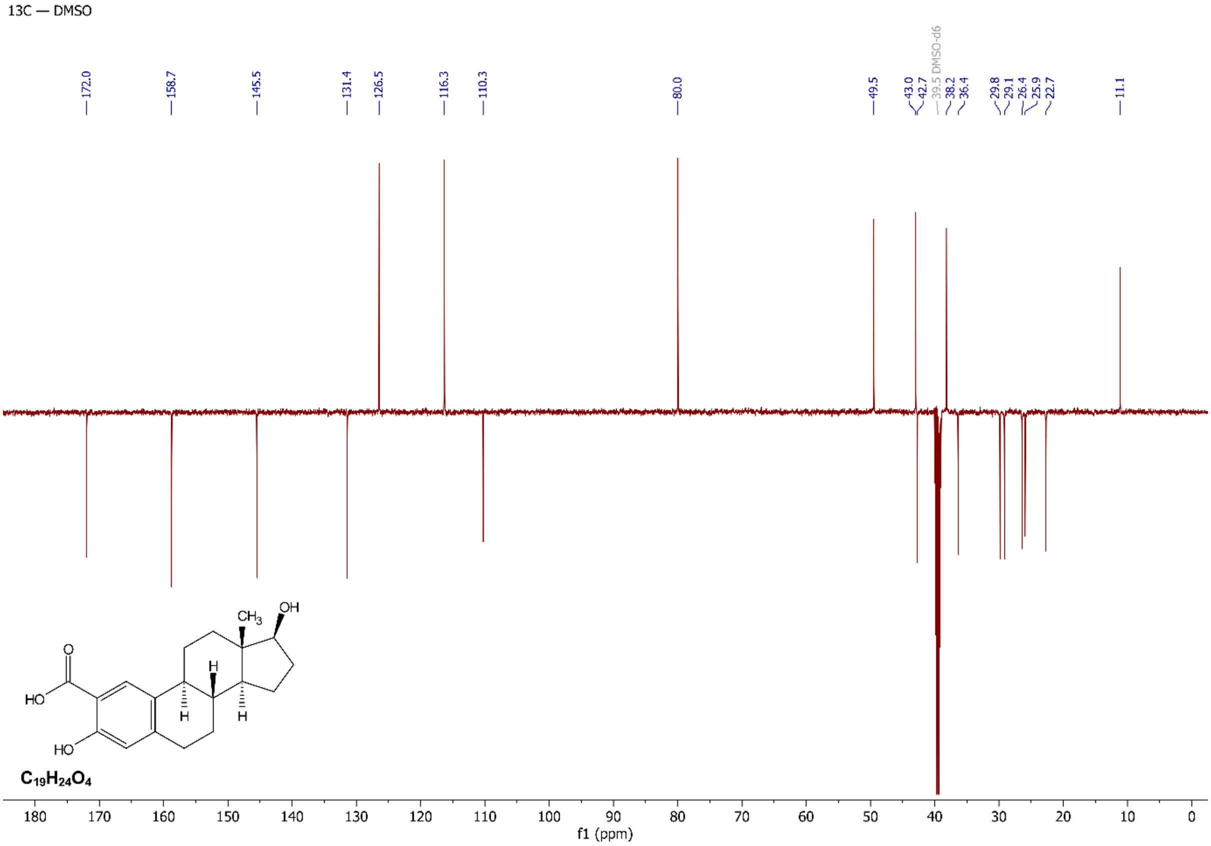

$^{1}$H NMR spectrum of compound **6d** ($CDCl_3$, 500 MHz)

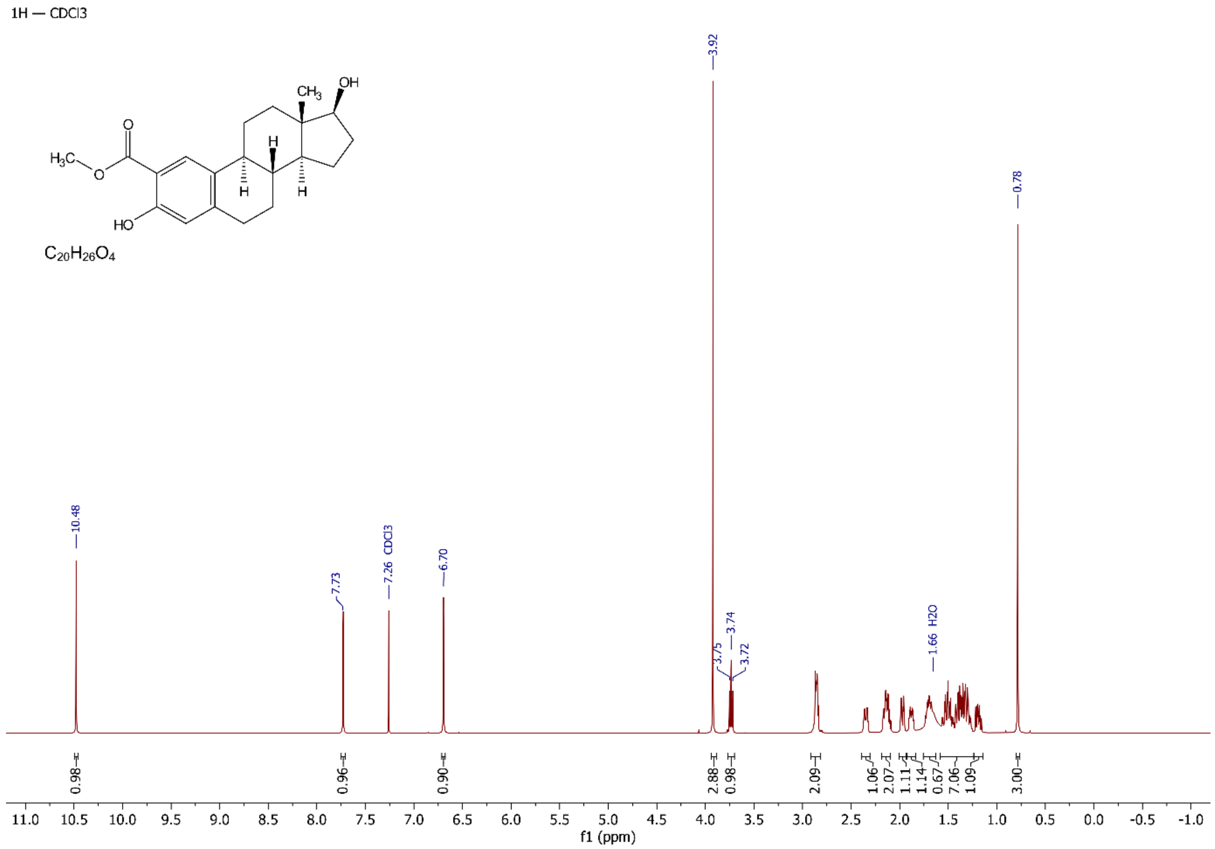


$^{13}$C NMR spectrum of compound **6d** ($CDCl_3$, 125 MHz)

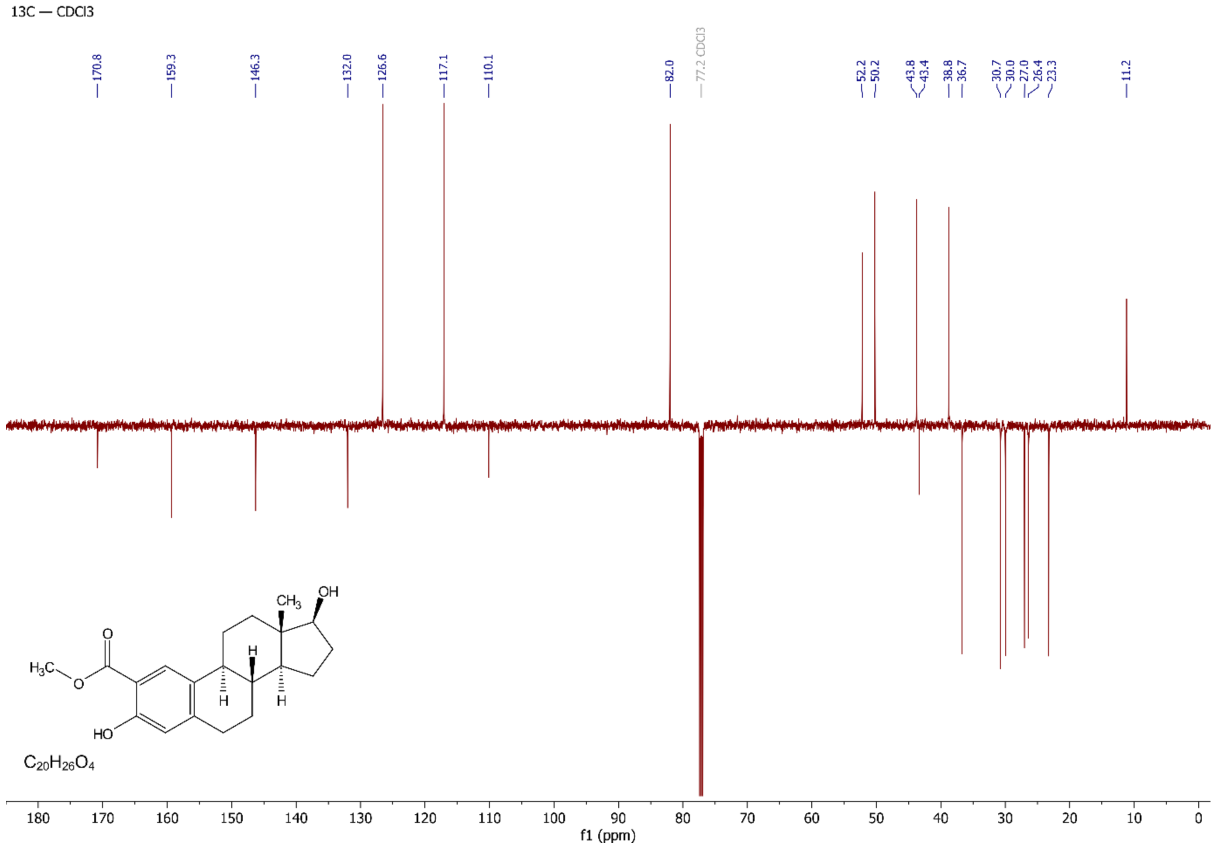

$^{1}H$ NMR spectrum of compound **7** (DMSO-$d_6$, 500 MHz)

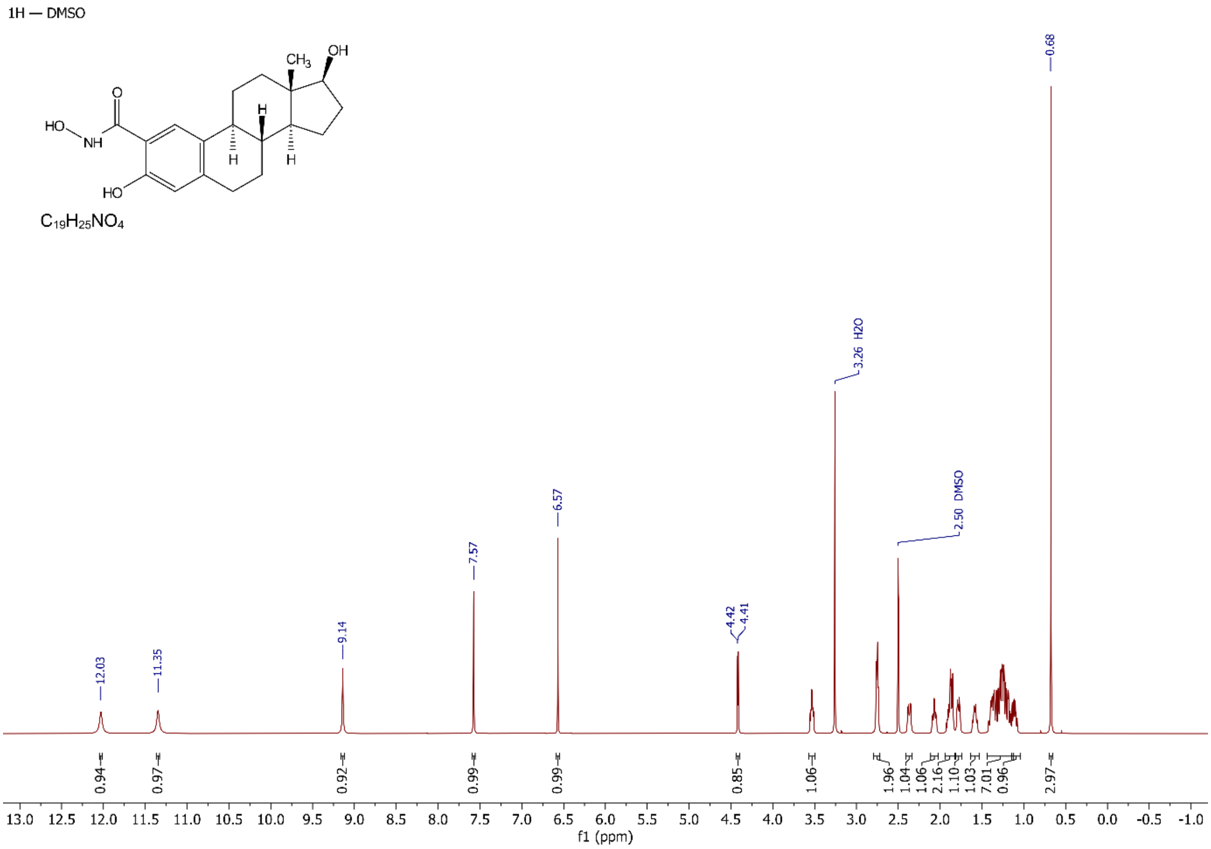


$^{13}C$ NMR spectrum of compound **7** (DMSO-$d_6$, 125 MHz)

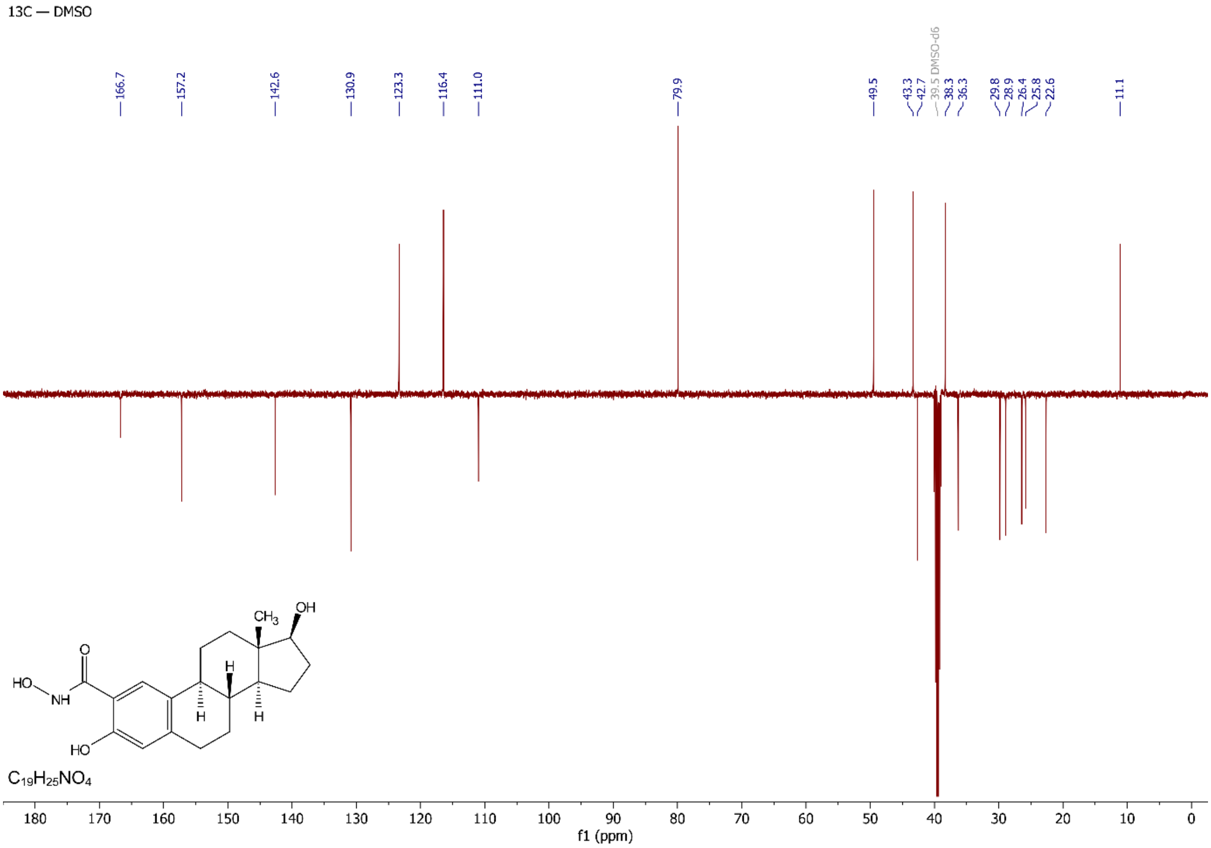

$^{1}$H NMR spectrum of compound **4h** (DMSO-$d_6$, 500 MHz)

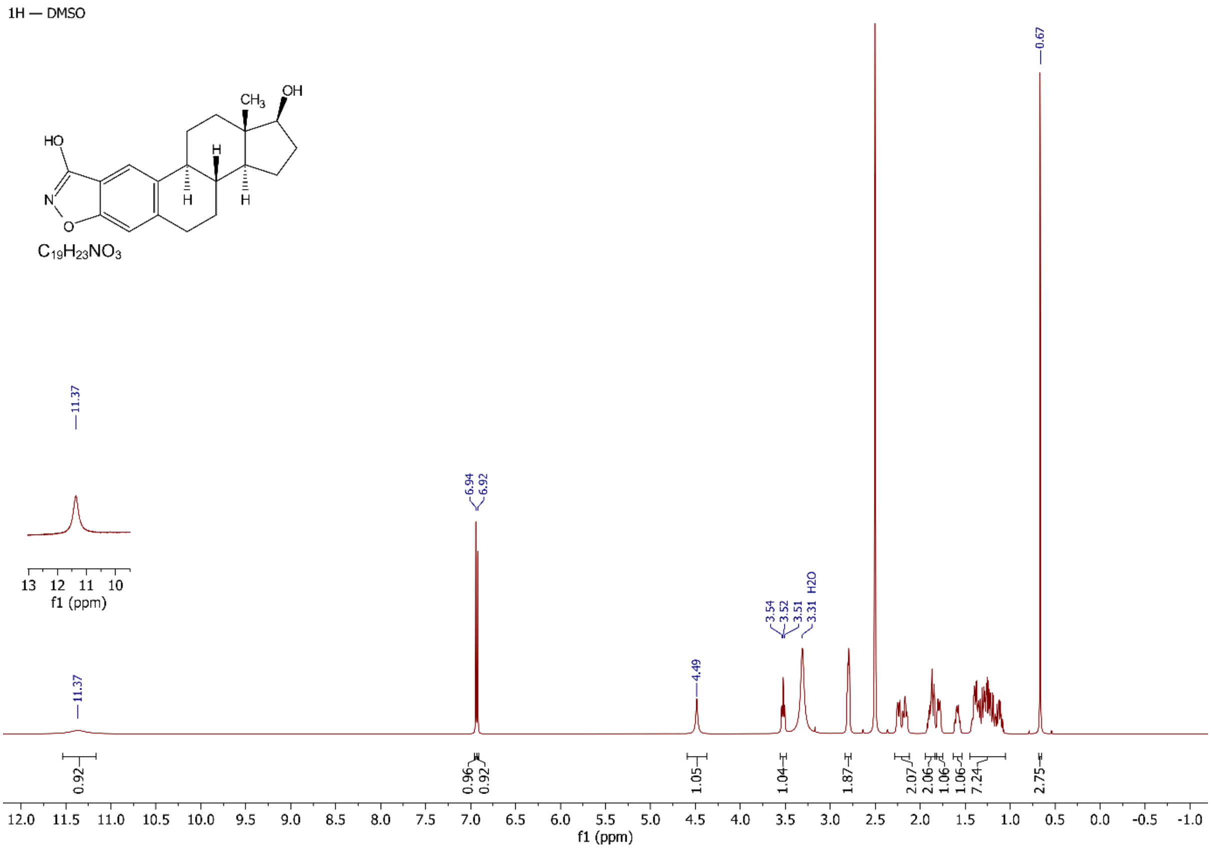


$^{13}$C NMR spectrum of compound **4h** (DMSO-$d_6$, 125 MHz)

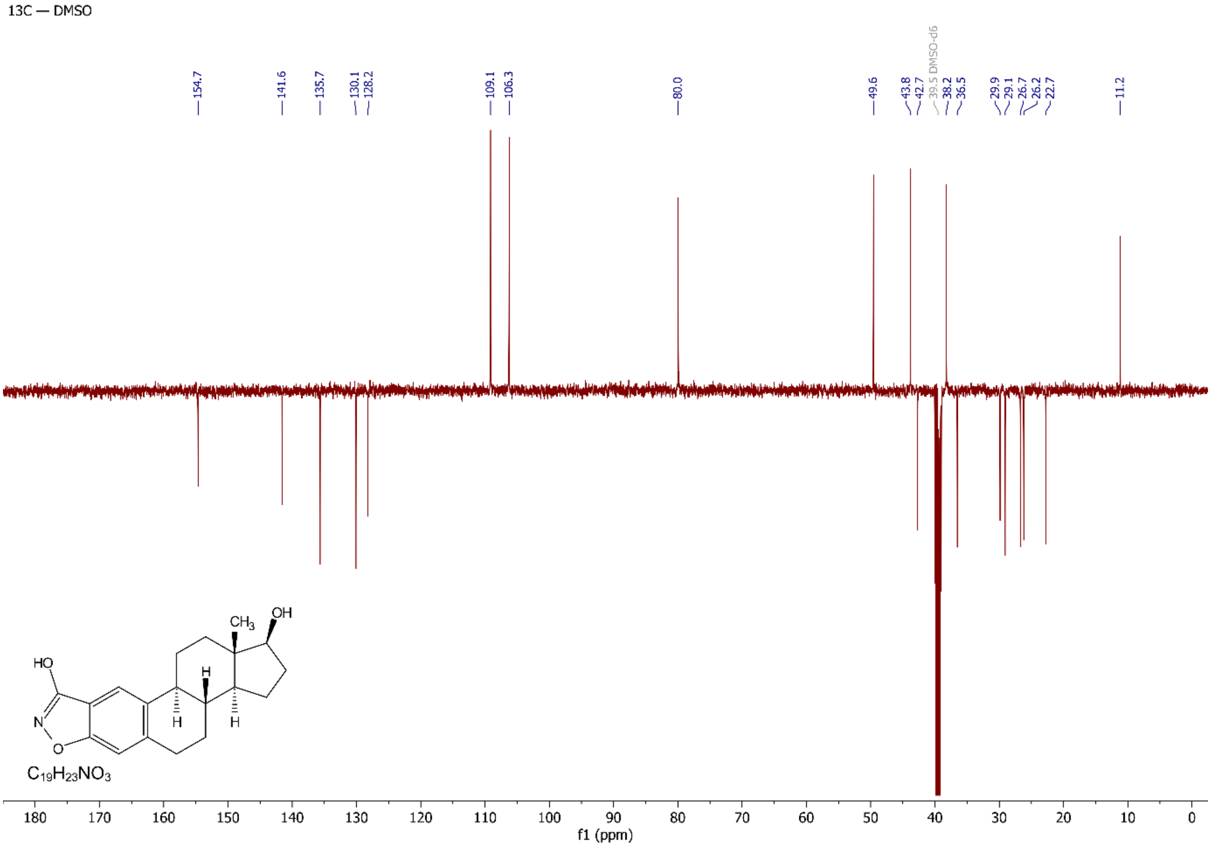

$^{1}H$ NMR spectrum of compound **4i** ($CDCl_3$, 500 MHz)

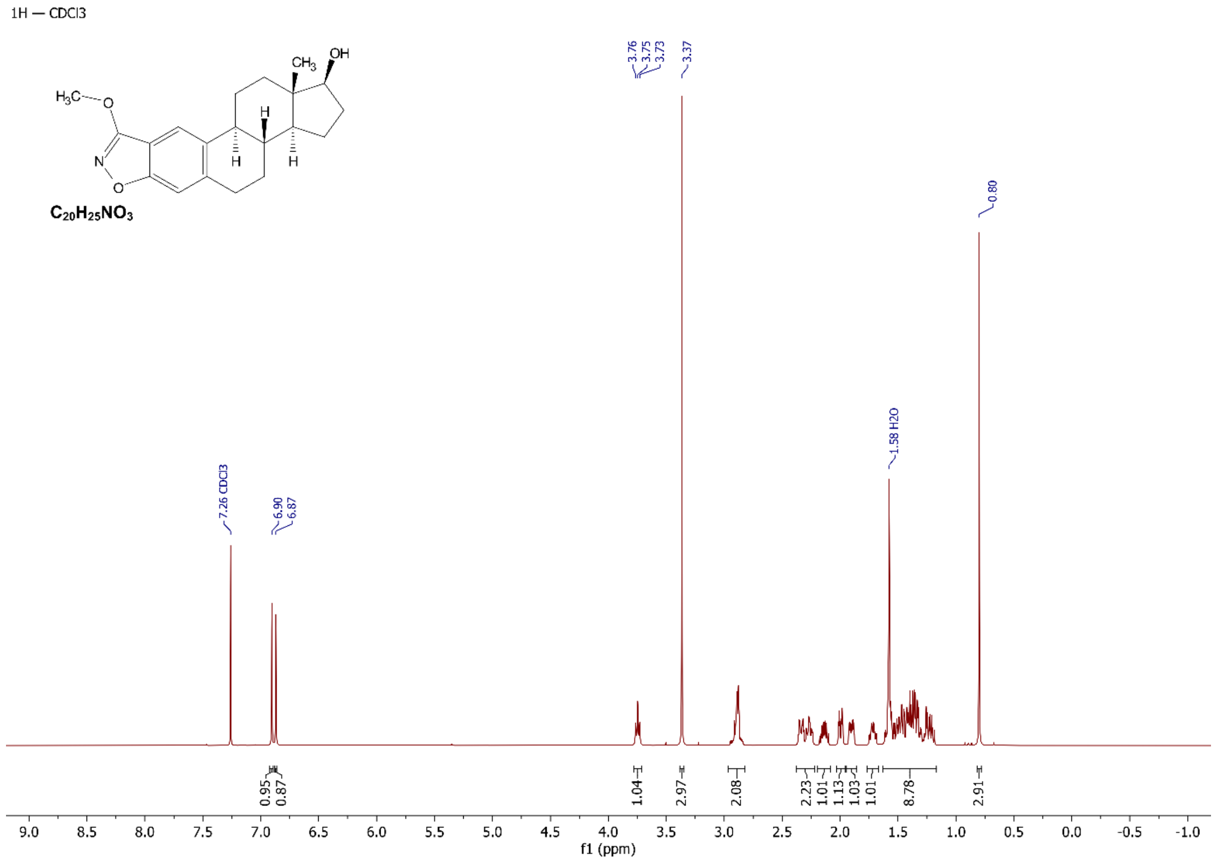


$^{13}C$ NMR spectrum of compound **4i** ($CDCl_3$, 125 MHz)

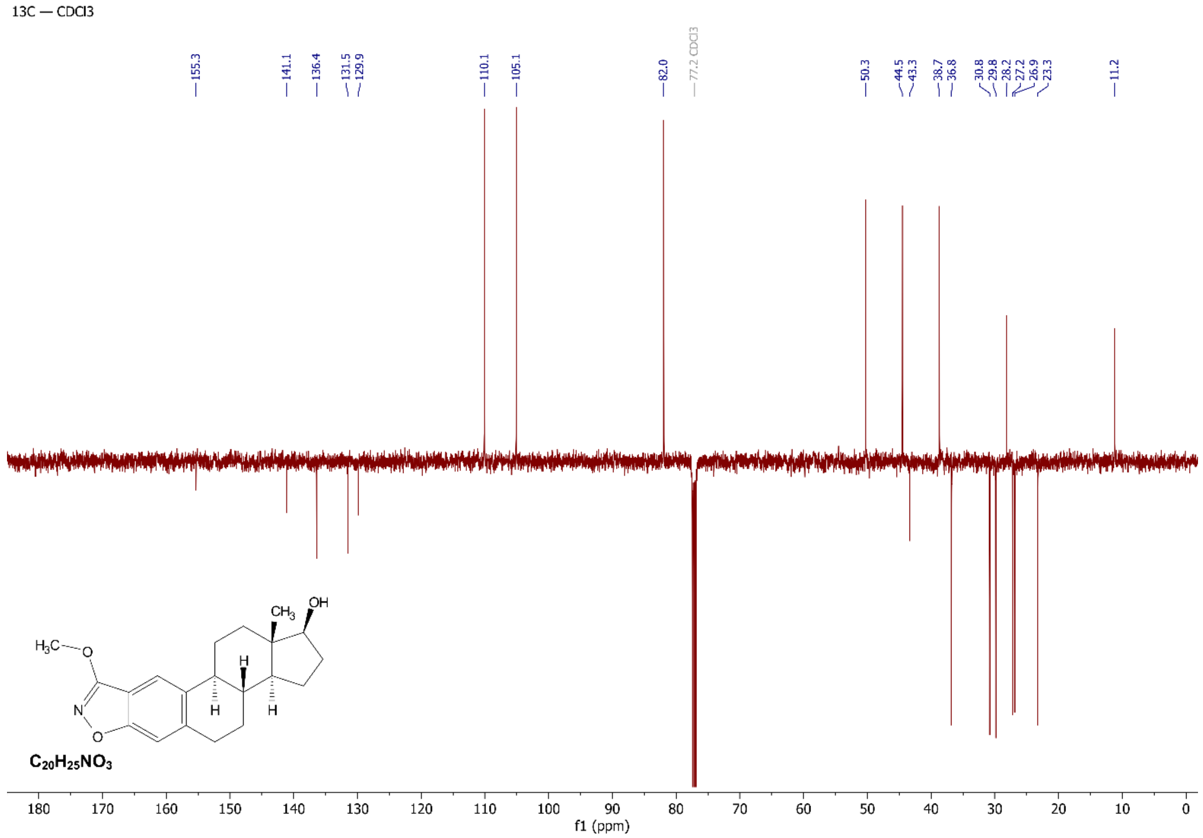

## $^1H$ NMR spectrum of compound **4j** ($CDCl_3$, 500 MHz)

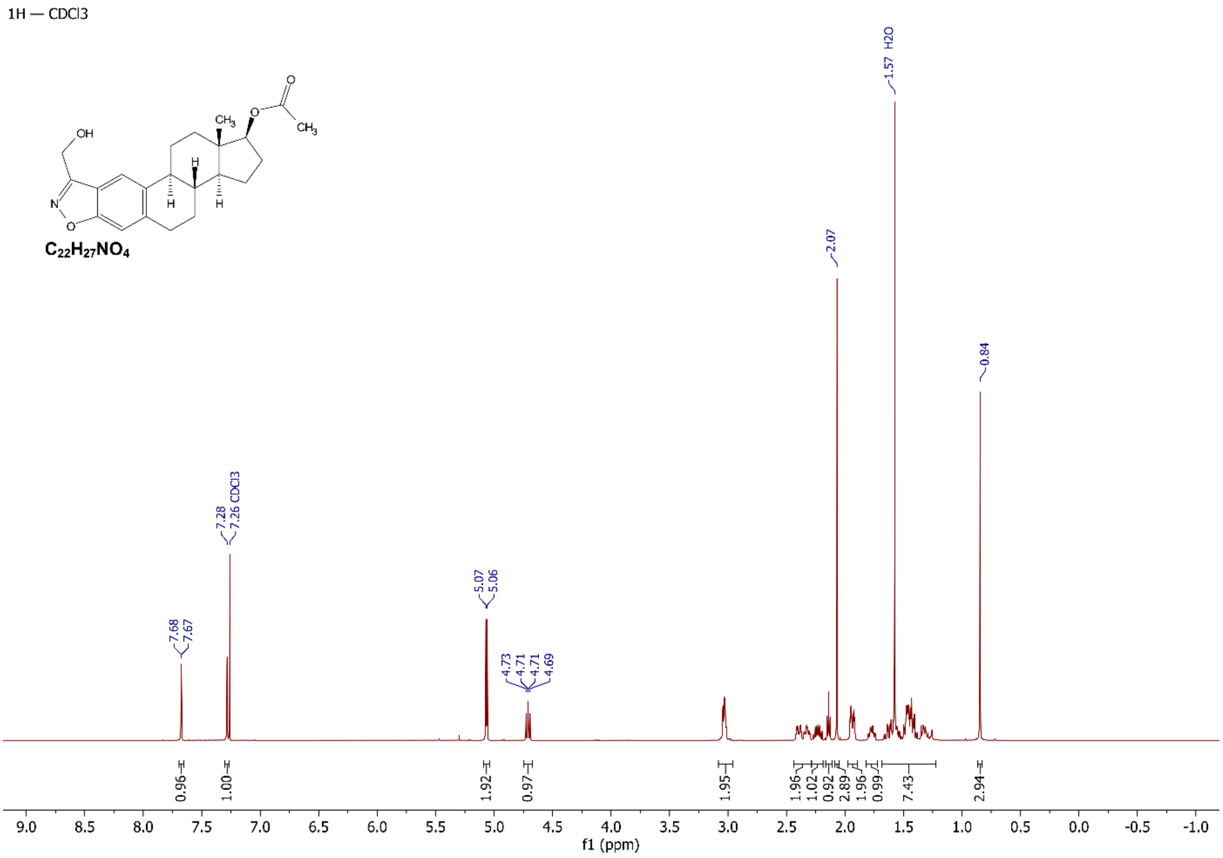


## $^{13}C$ NMR spectrum of compound **4j** ($CDCl_3$, 125 MHz)

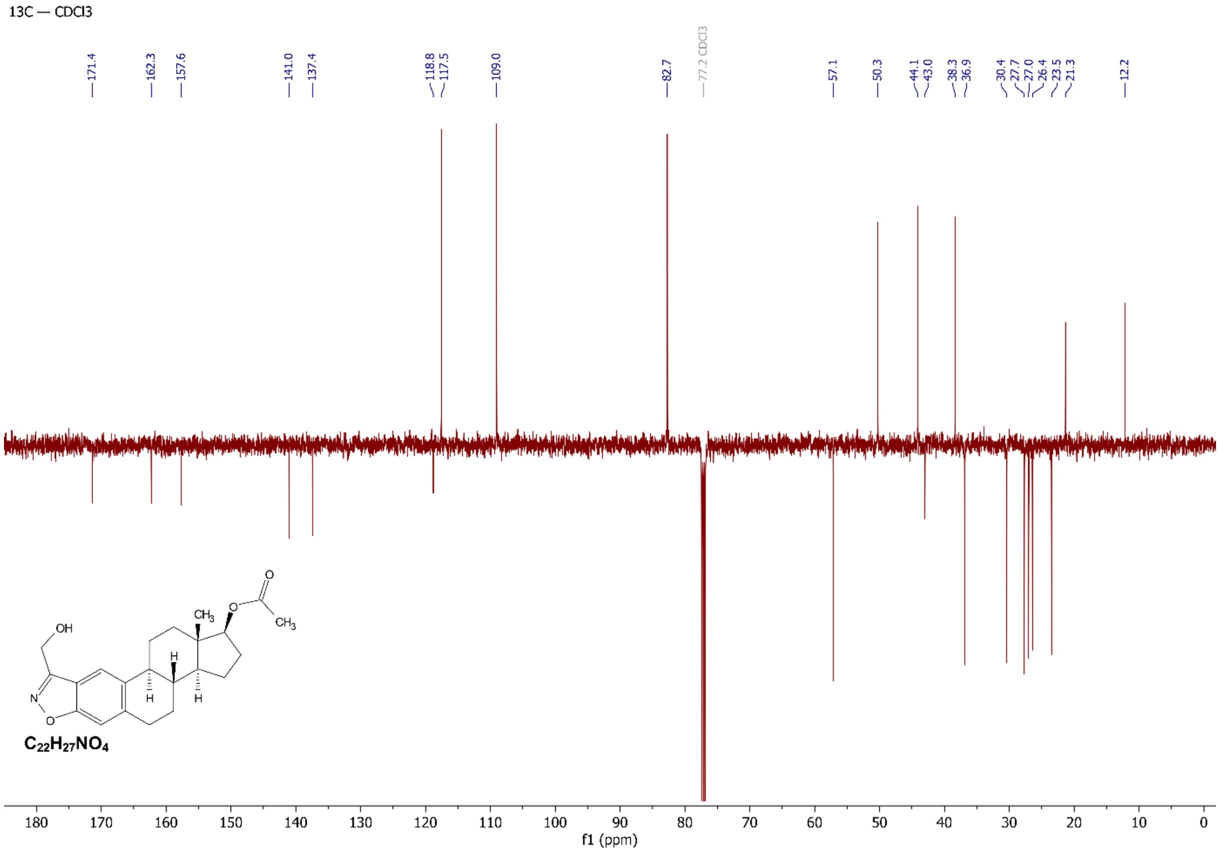

$^{1}H$ NMR spectrum of compound **4k** (DMSO-*d*$_6$, 500 MHz)

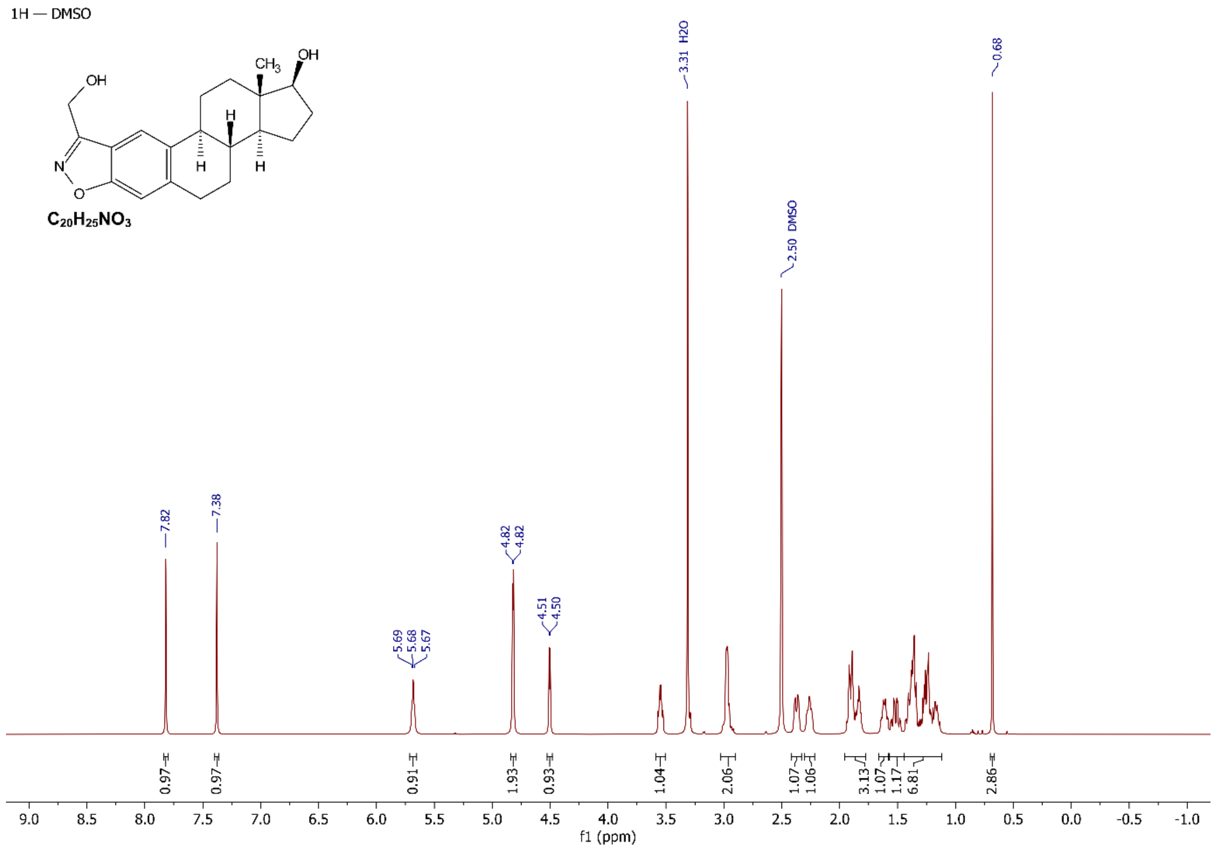


$^{13}C$ NMR spectrum of compound **4k** (DMSO-*d*$_6$, 125 MHz)

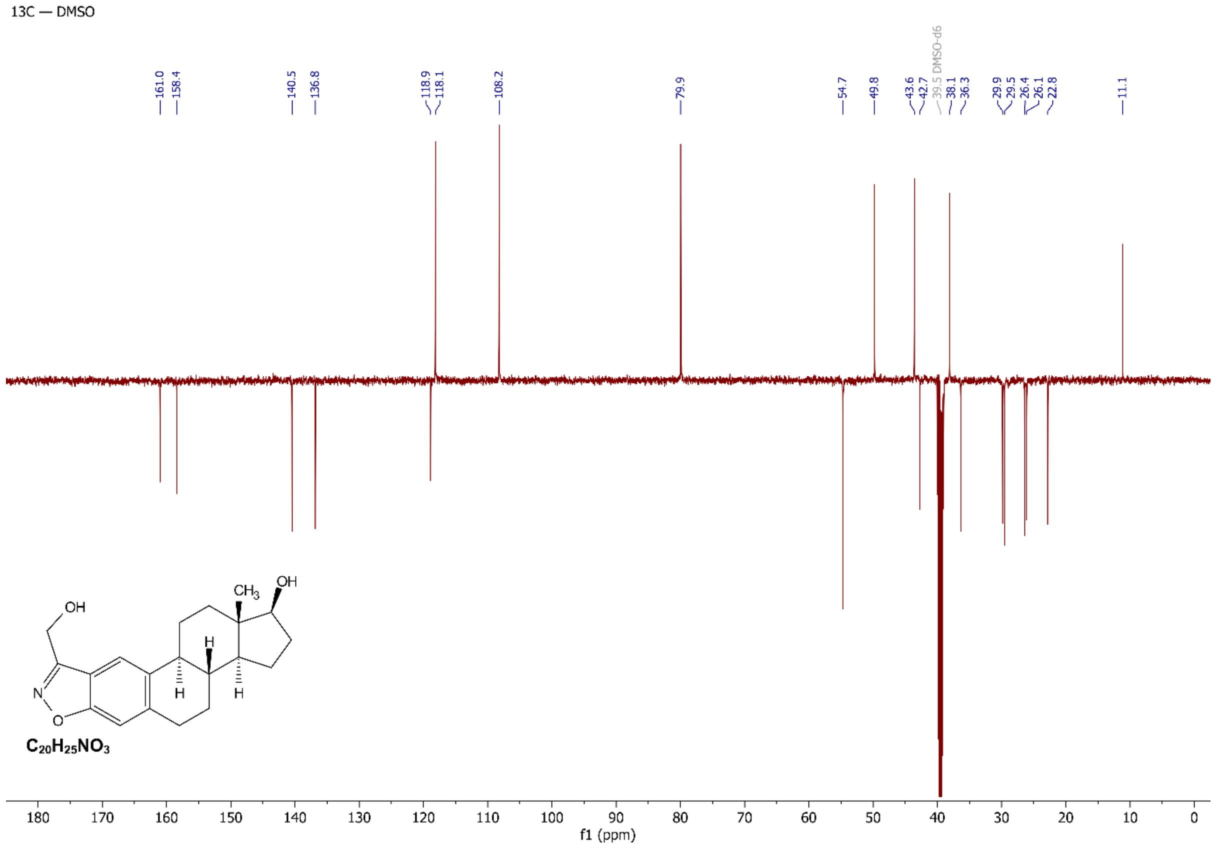

<sup></sup>

## $^{1}$H NMR spectrum of compound **4l** ($CDCl_3$, 500 MHz)

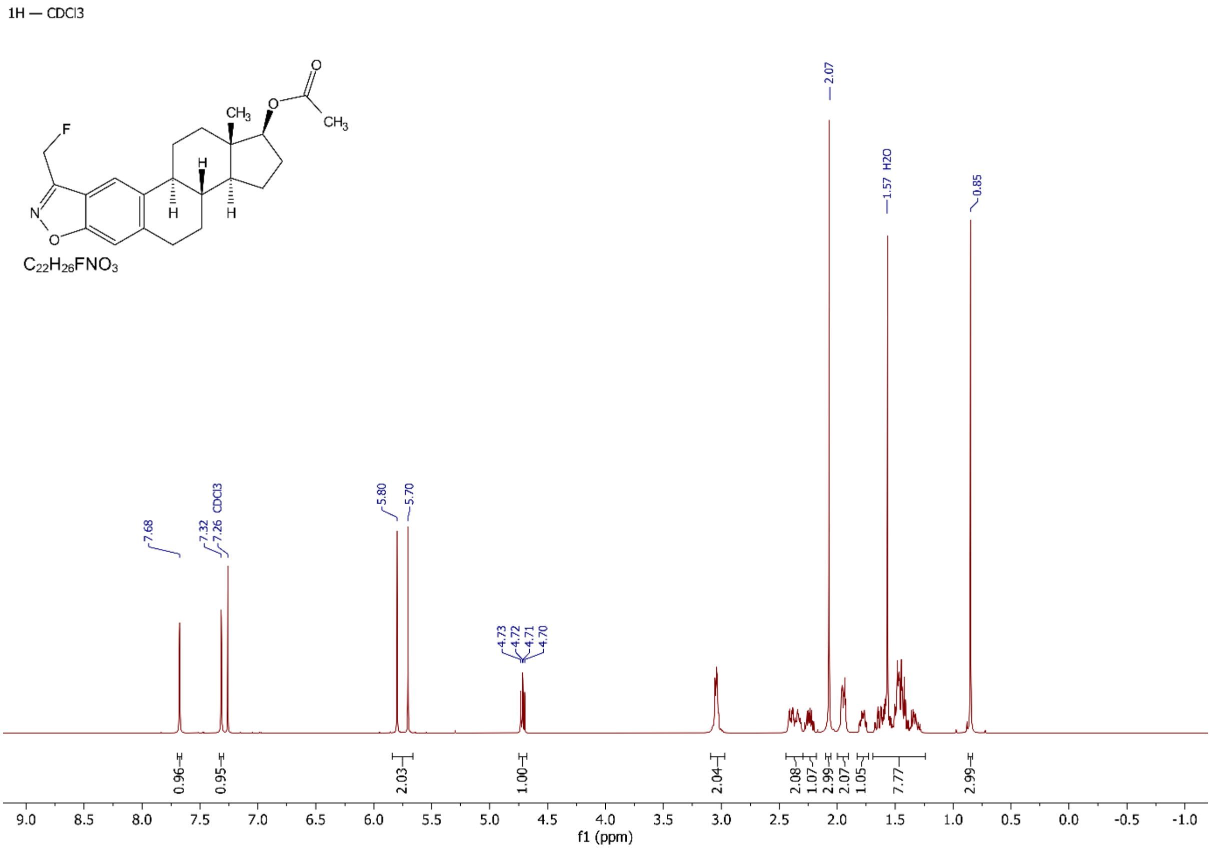


## $^{13}$C NMR spectrum of compound **4l** ($CDCl_3$, 125 MHz)

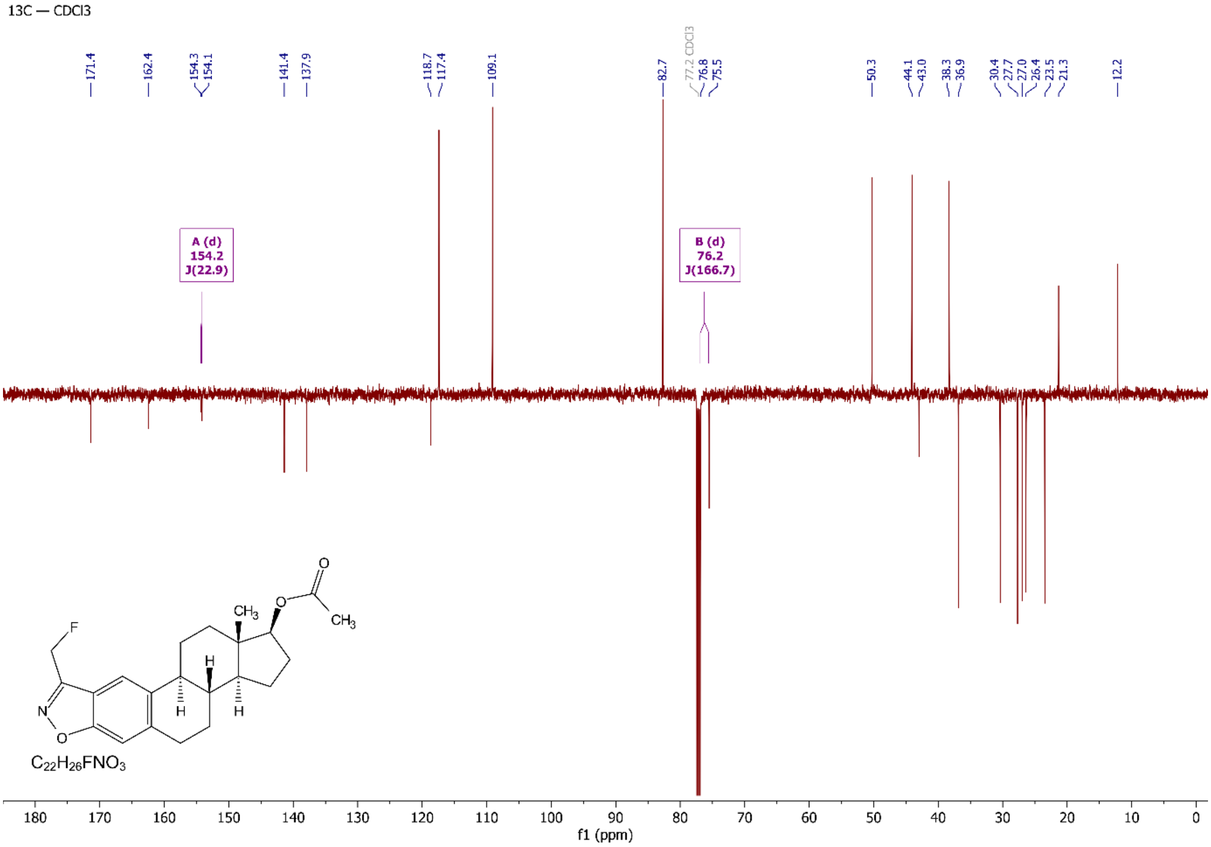

$^{1}$H NMR spectrum of compound **4m** ($CDCl_3$, 500 MHz)

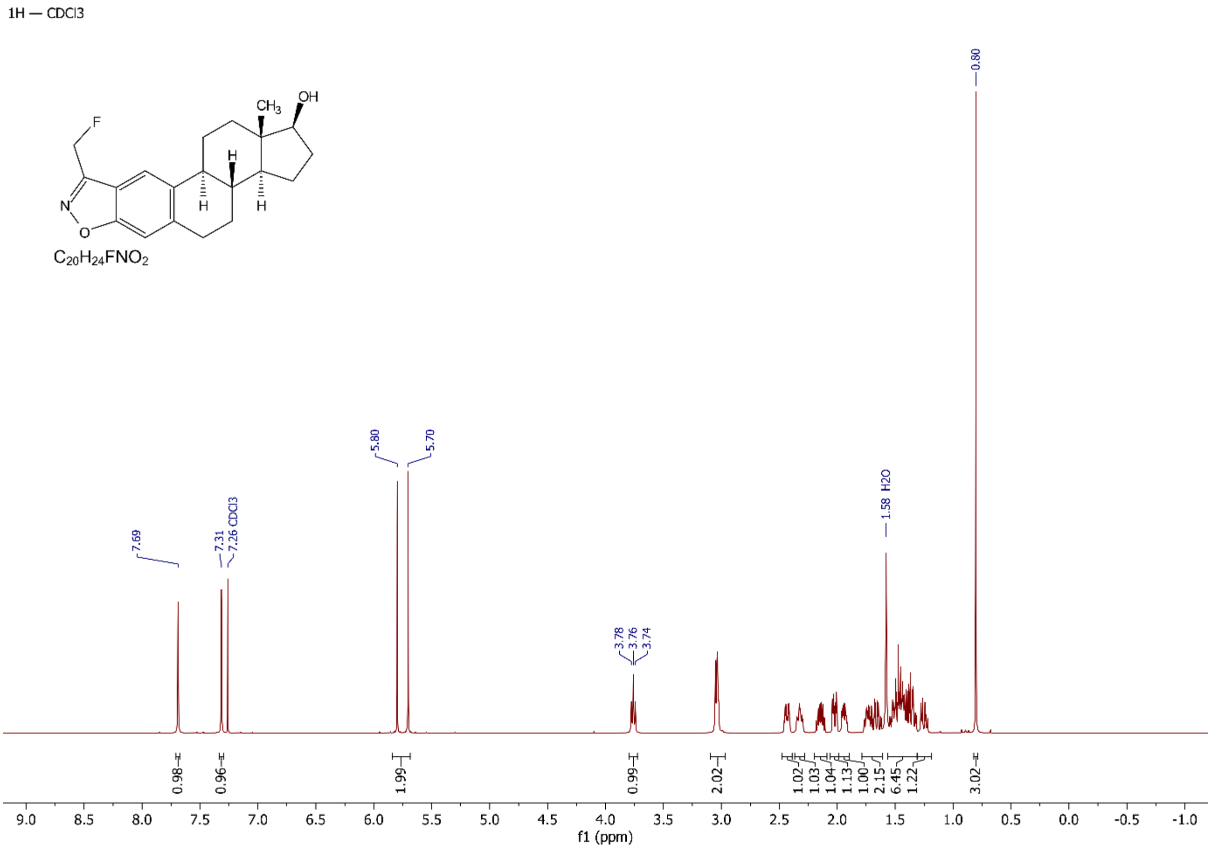


$^{13}$C NMR spectrum of compound **4m** ($CDCl_3$, 125 MHz)

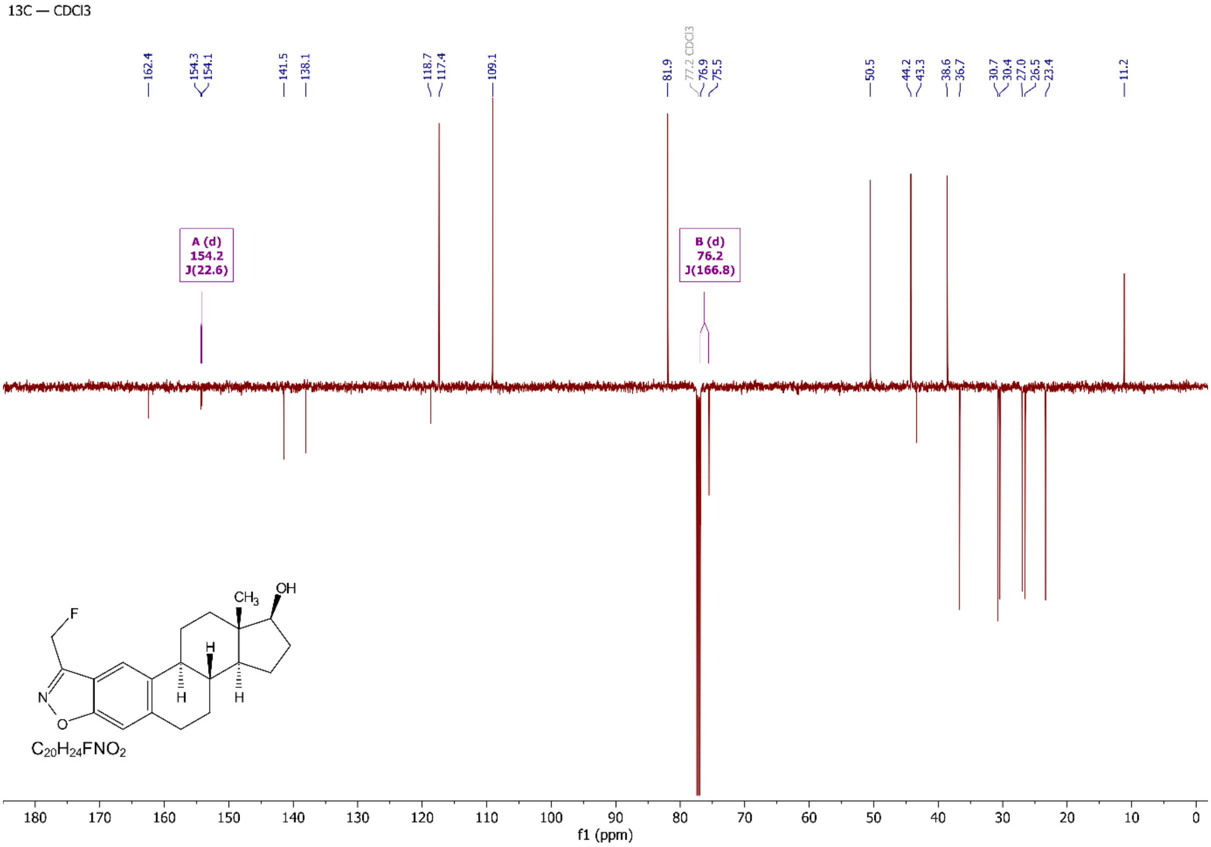

$^{1}$H NMR spectrum of compound **4n** ($CDCl_3$, 500 MHz)

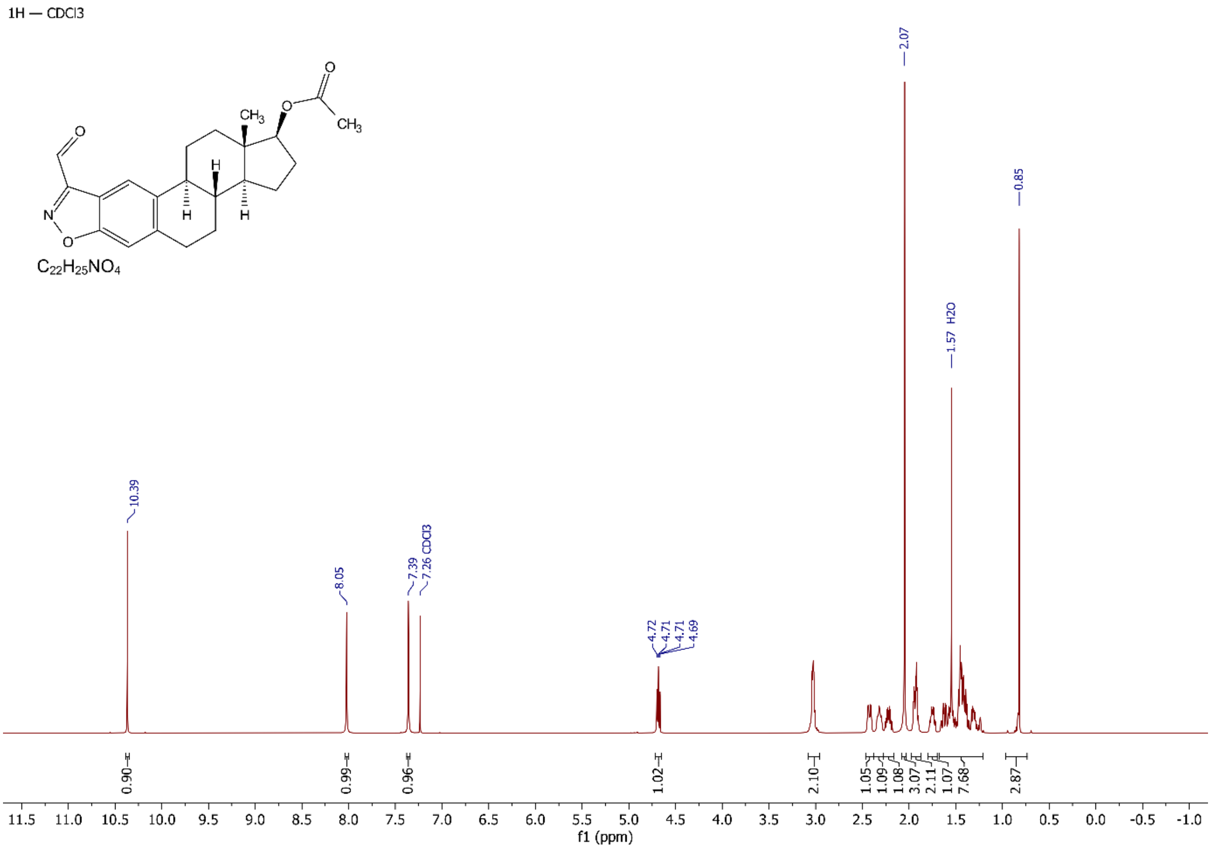


$^{13}$C NMR spectrum of compound **4n** ($CDCl_3$, 125 MHz)

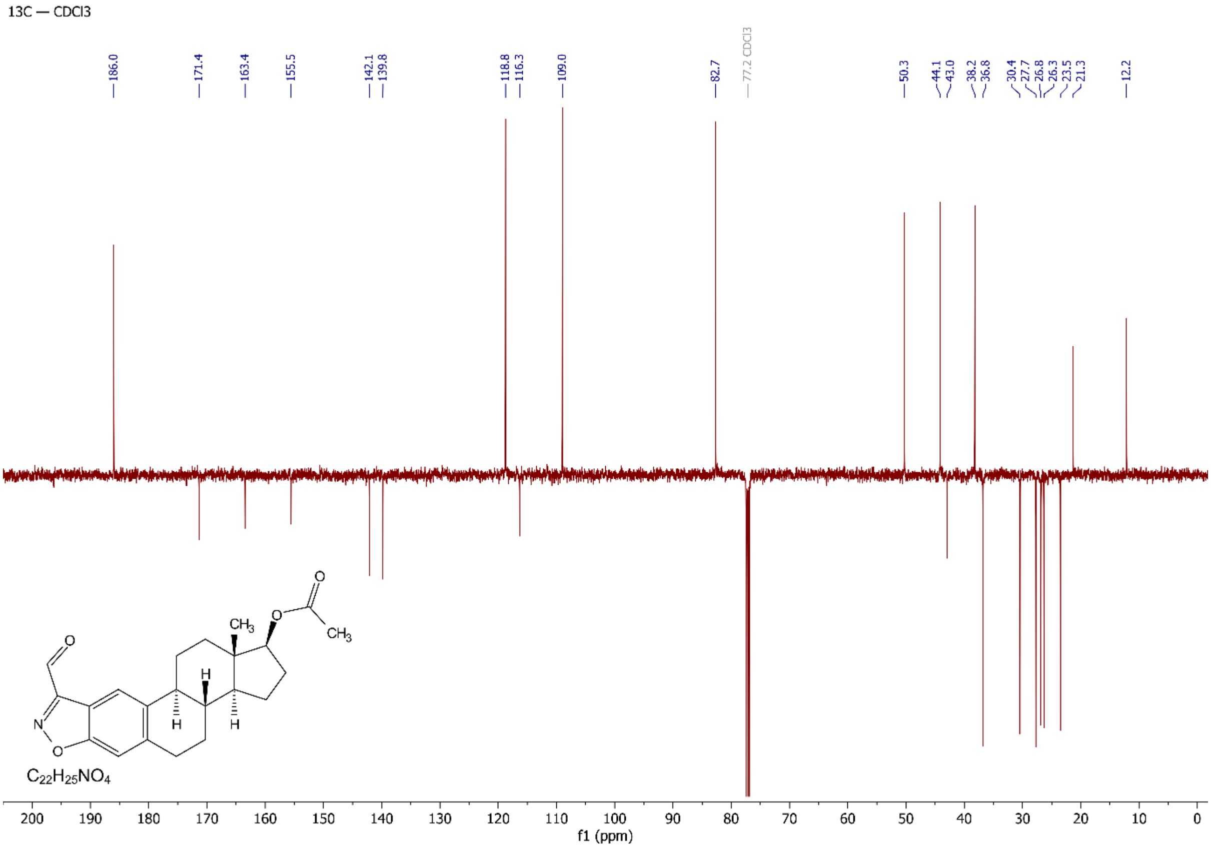

$^{1}H$ NMR spectrum of compound **4o** ($CDCl_3$, 500 MHz)

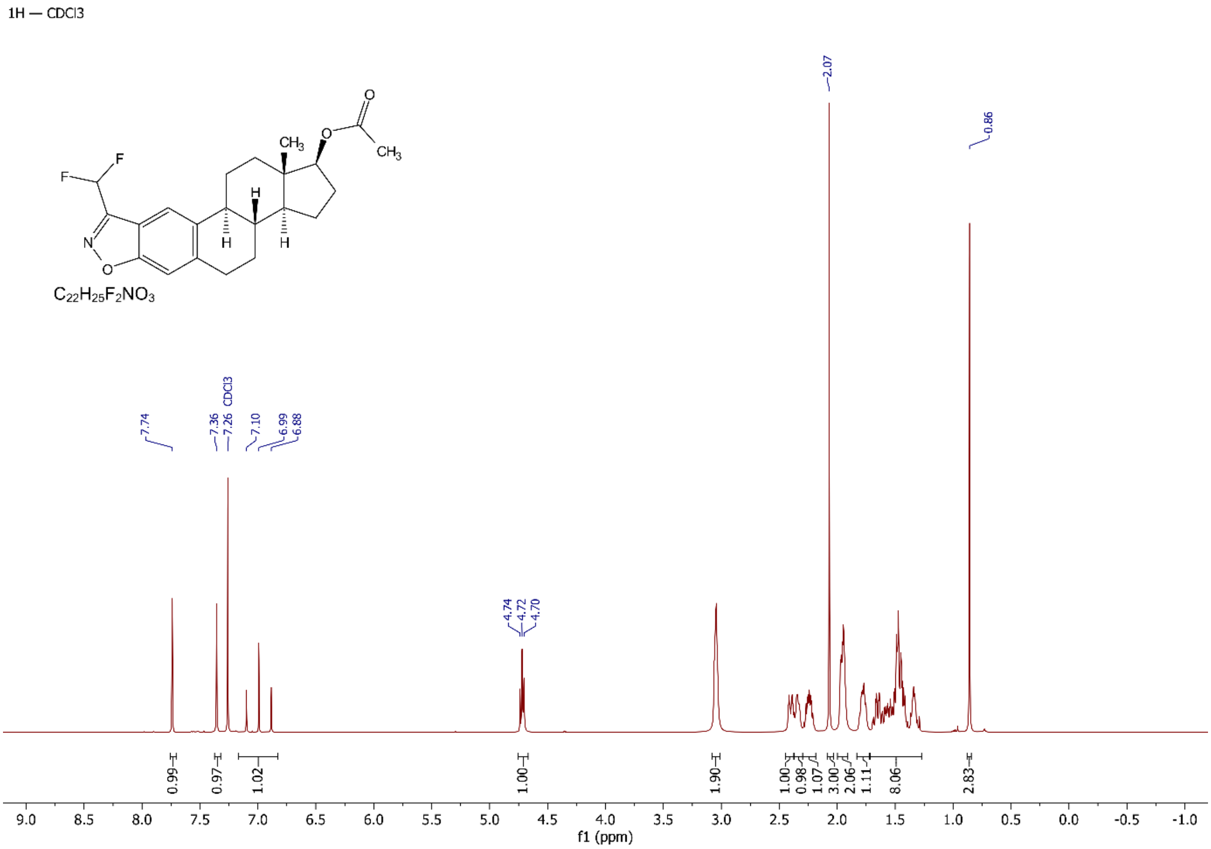


$^{13}C$ NMR spectrum of compound **4o** ($CDCl_3$, 125 MHz)

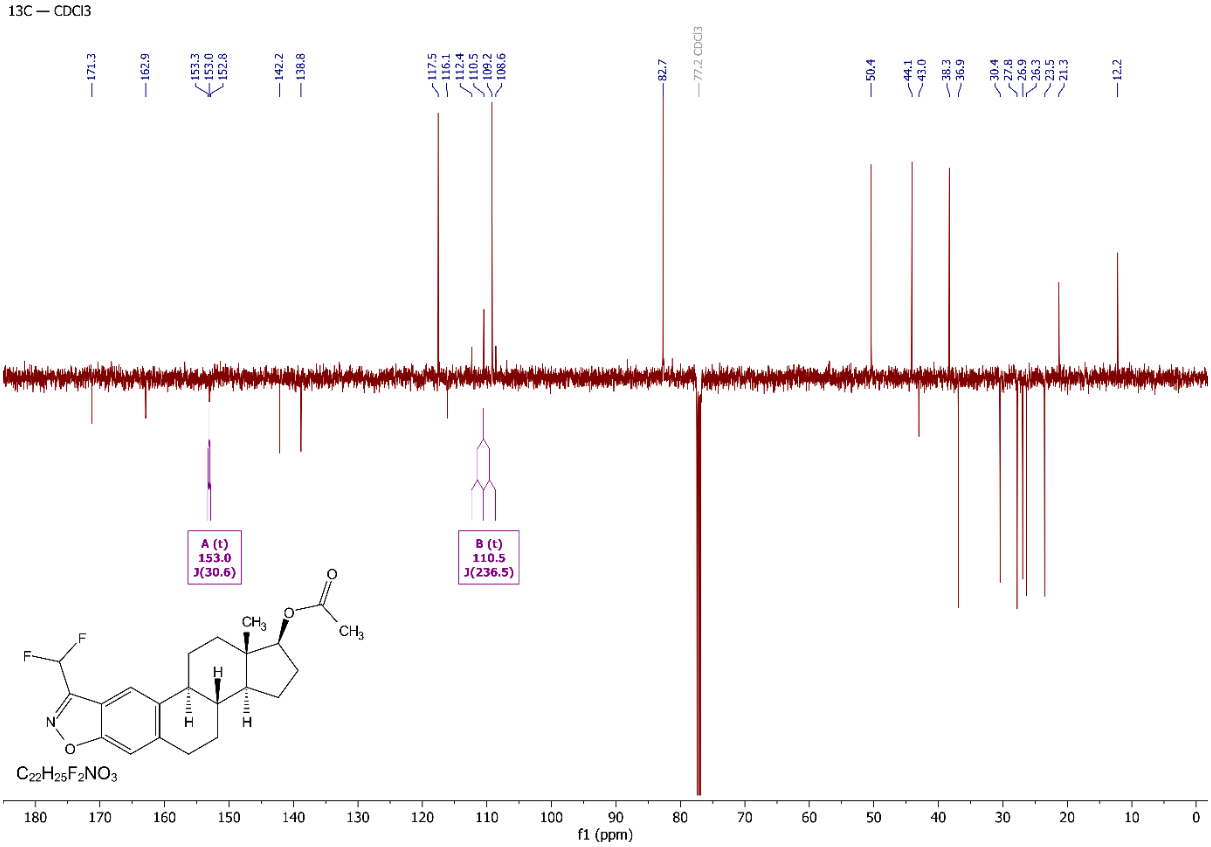

$^1$H NMR spectrum of compound **4p** ($CDCl_3$, 500 MHz)

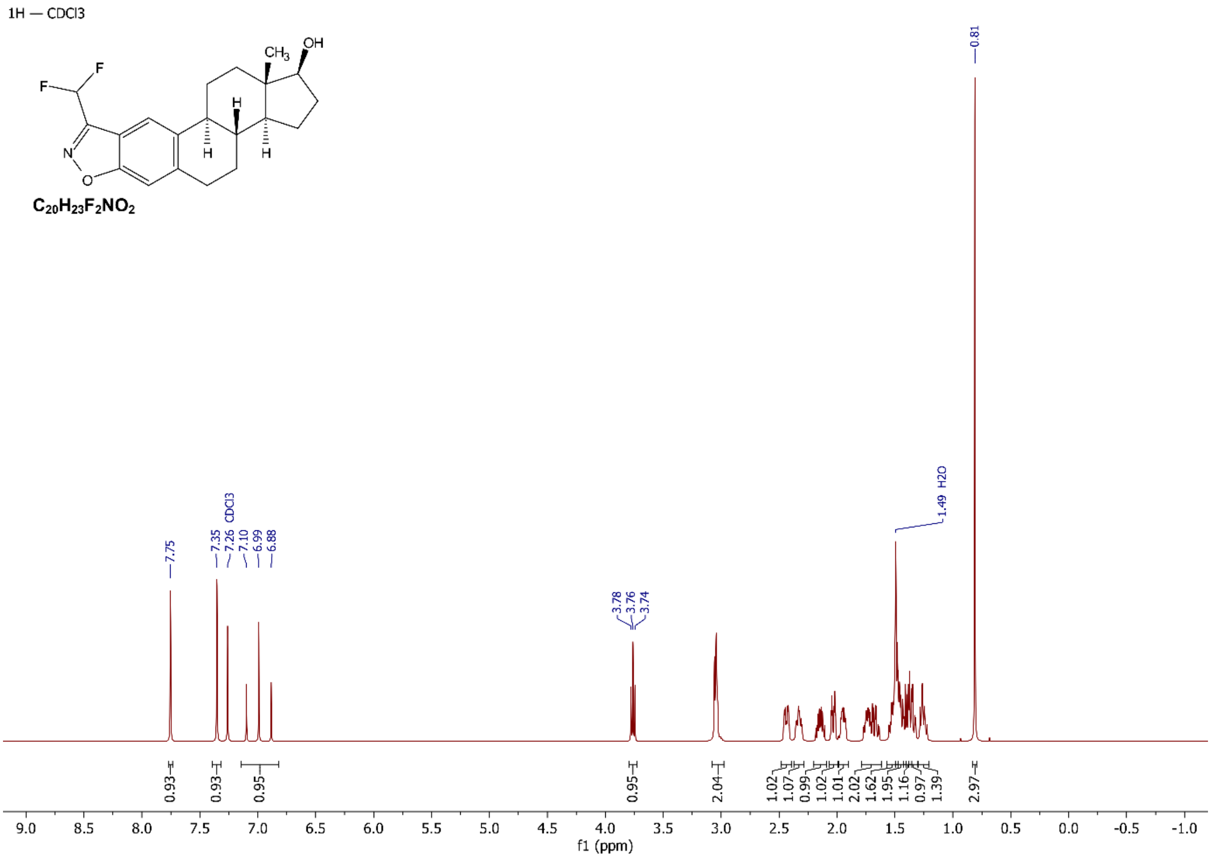


$^{13}$C NMR spectrum of compound **4p** ($CDCl_3$, 125 MHz)

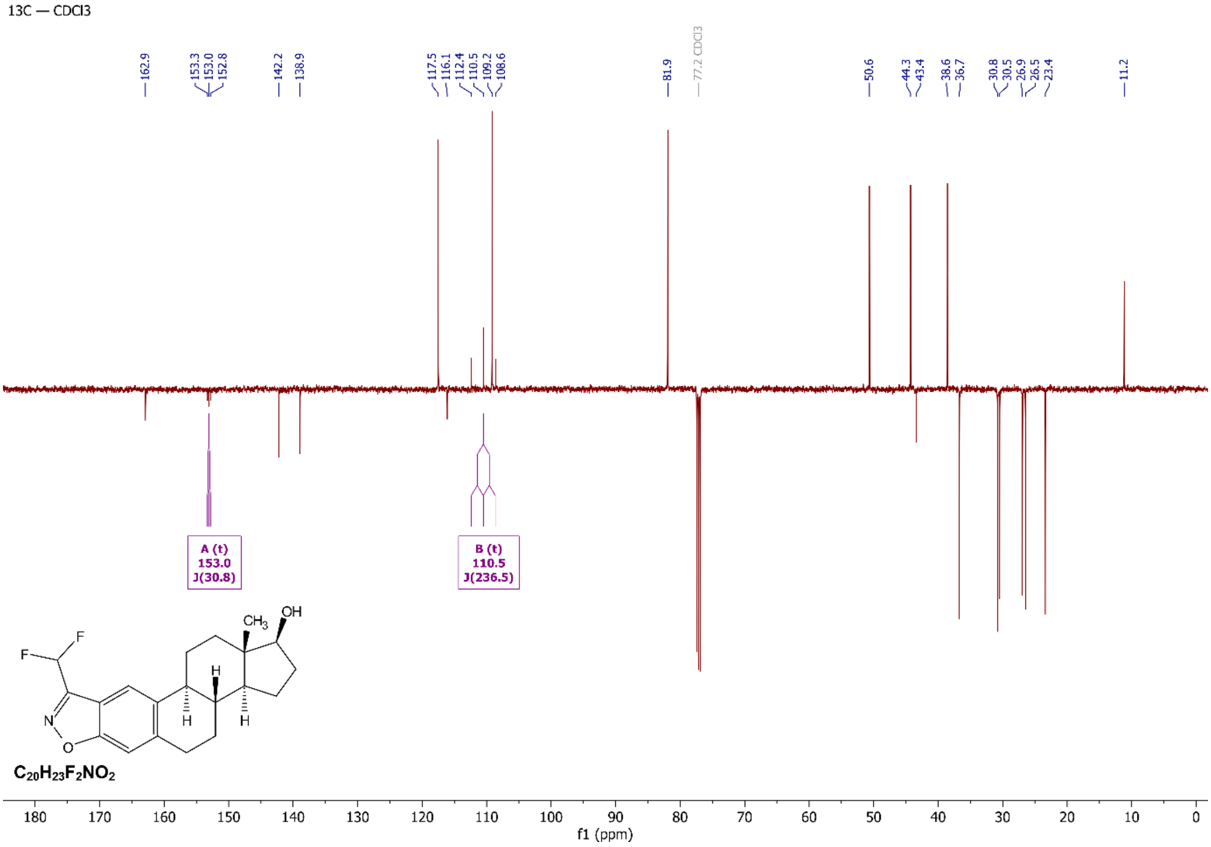

## $^{1}$H NMR spectrum of compound **4q** (DMSO-*d*$_6$, 500 MHz)

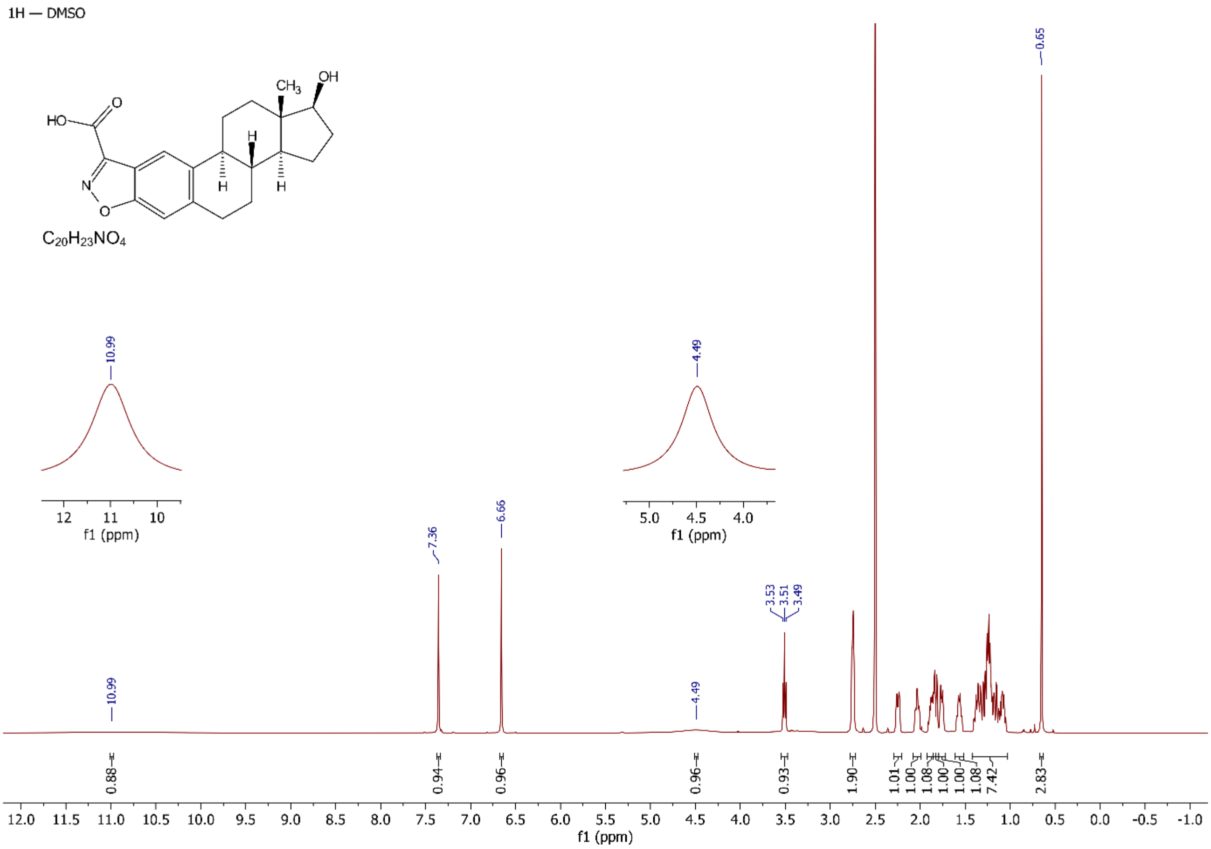


## $^{13}$C NMR spectrum of compound **4q** (DMSO-*d*$_6$, 125 MHz)

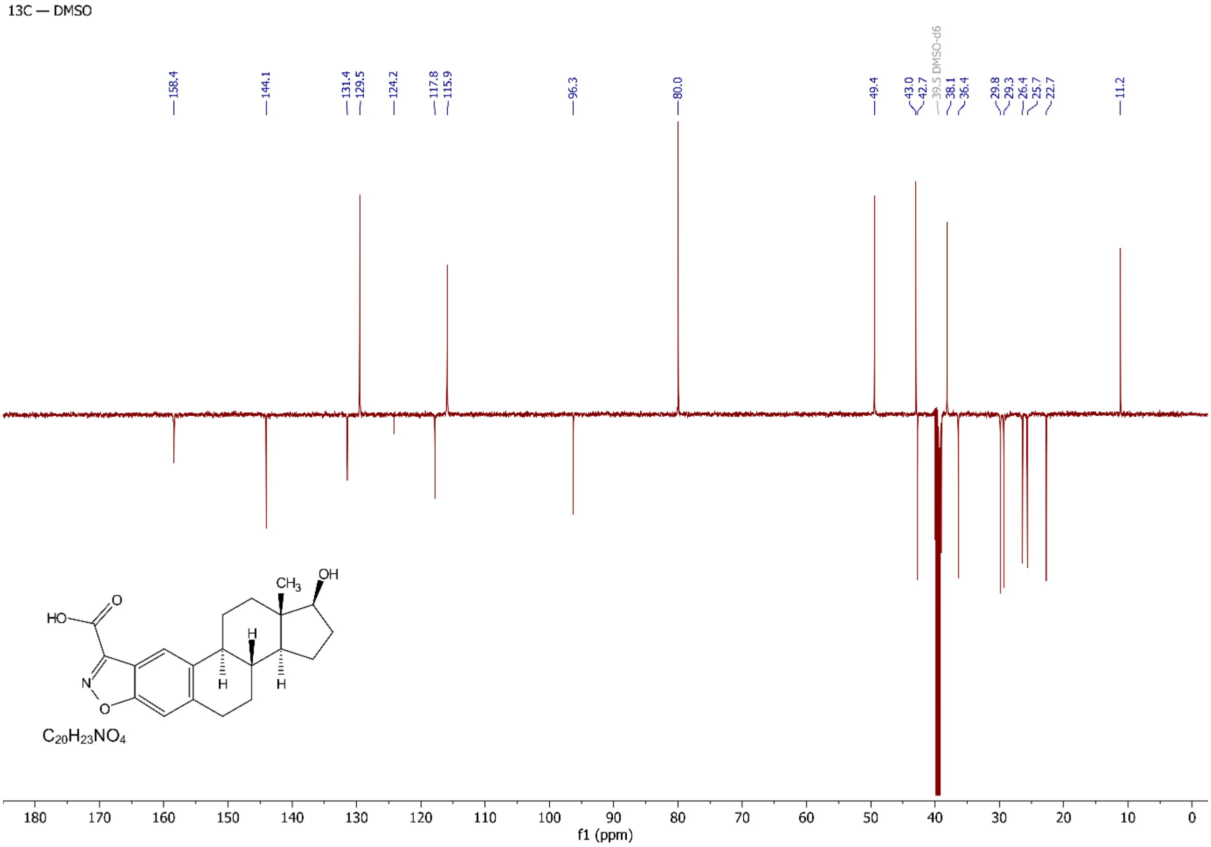

$^{1}$H NMR spectrum of compound **4r** ($CDCl_3$, 500 MHz)

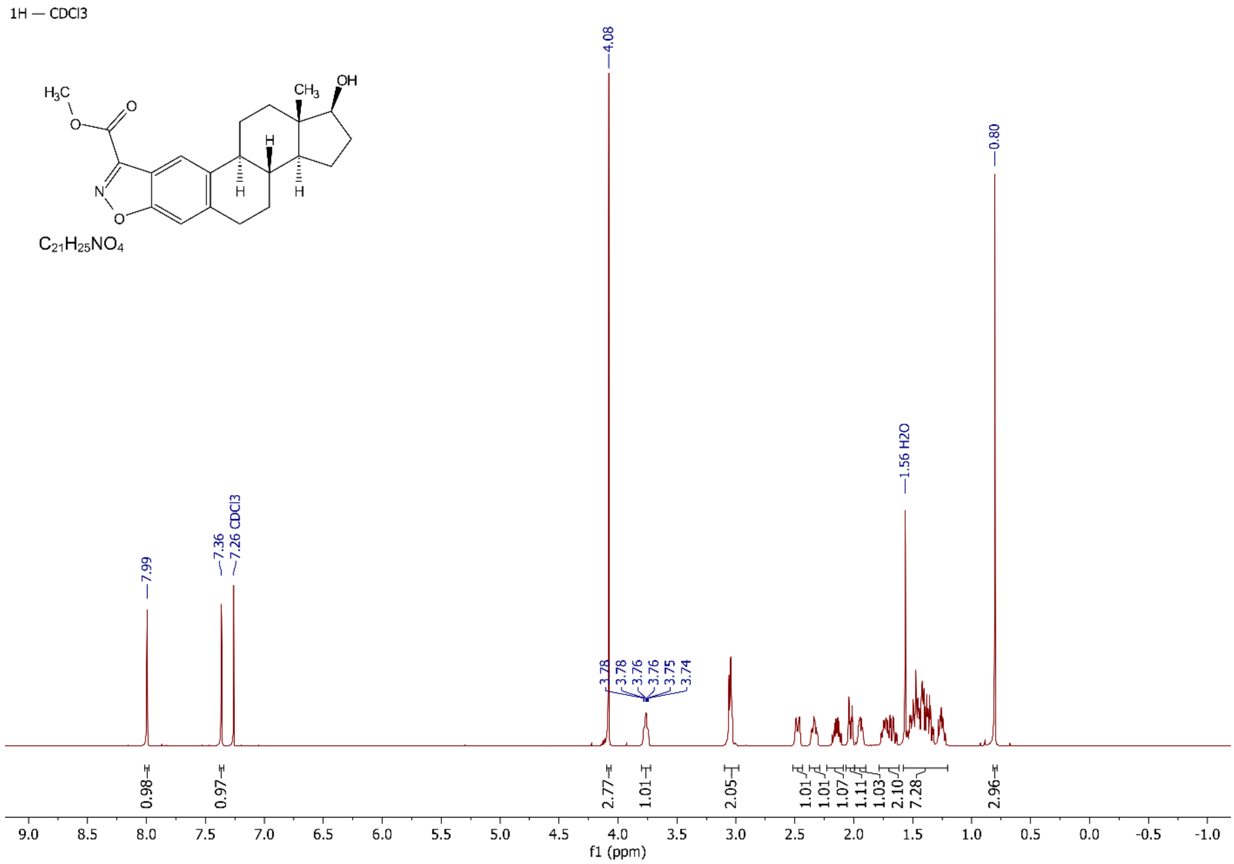


$^{13}$C NMR spectrum of compound **4r** ($CDCl_3$, 125 MHz)

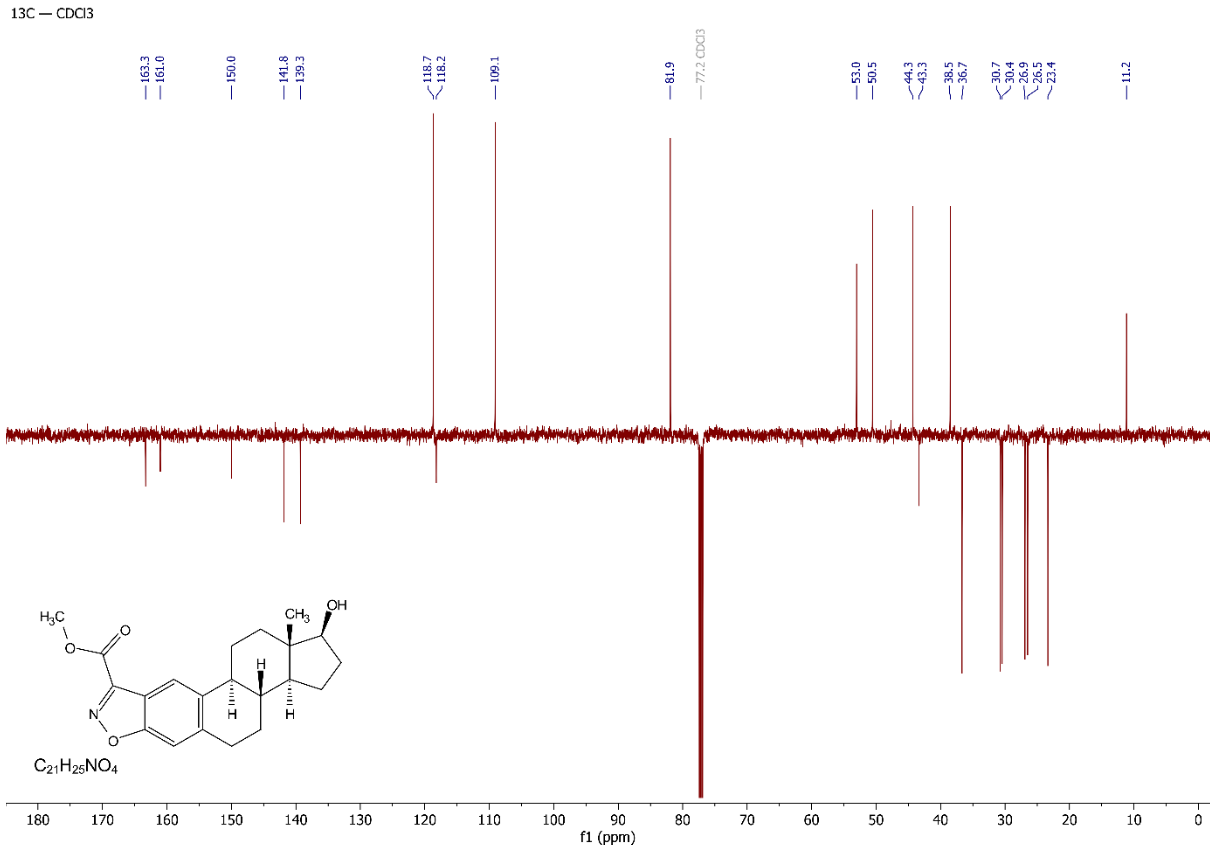

$^{1}H$ NMR spectrum of compound **4s** (DMSO-$d_6$, 500 MHz)

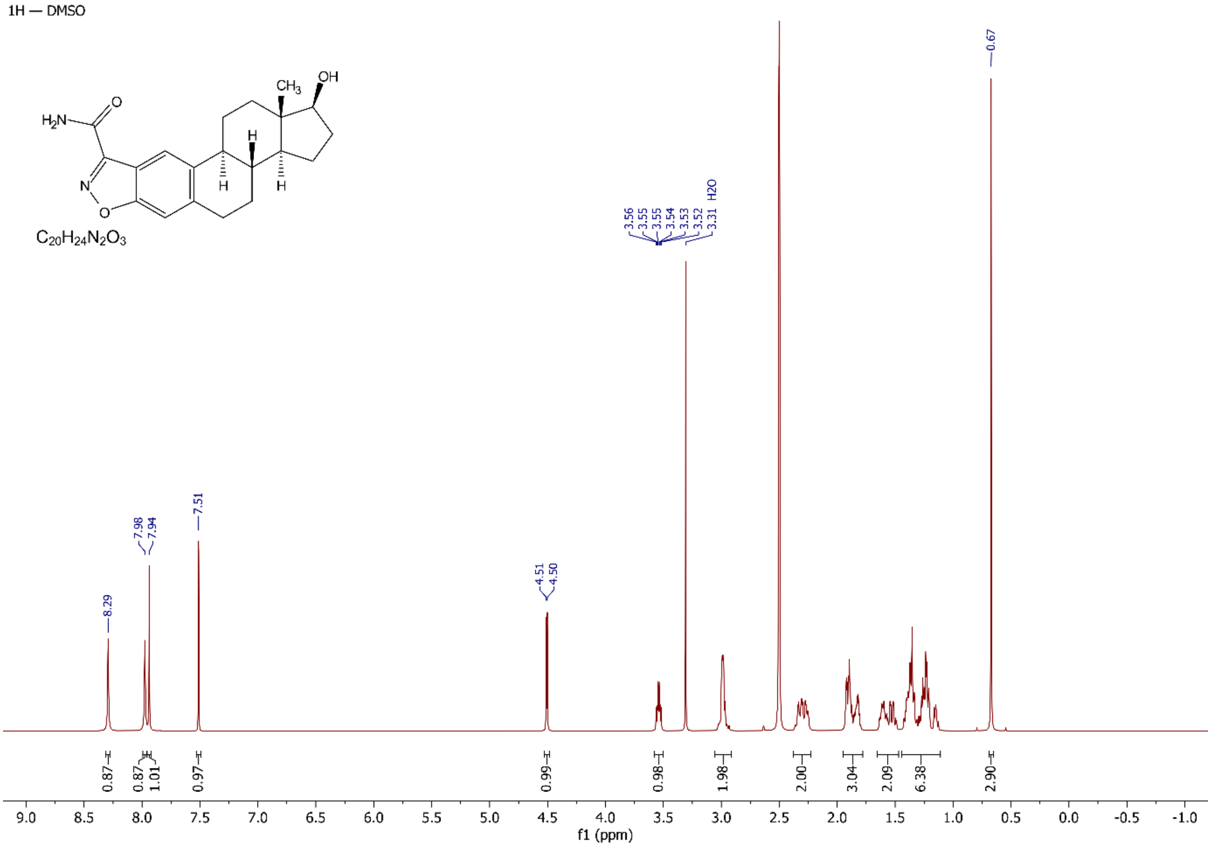


$^{13}C$ NMR spectrum of compound **4s** (DMSO-$d_6$, 125 MHz)

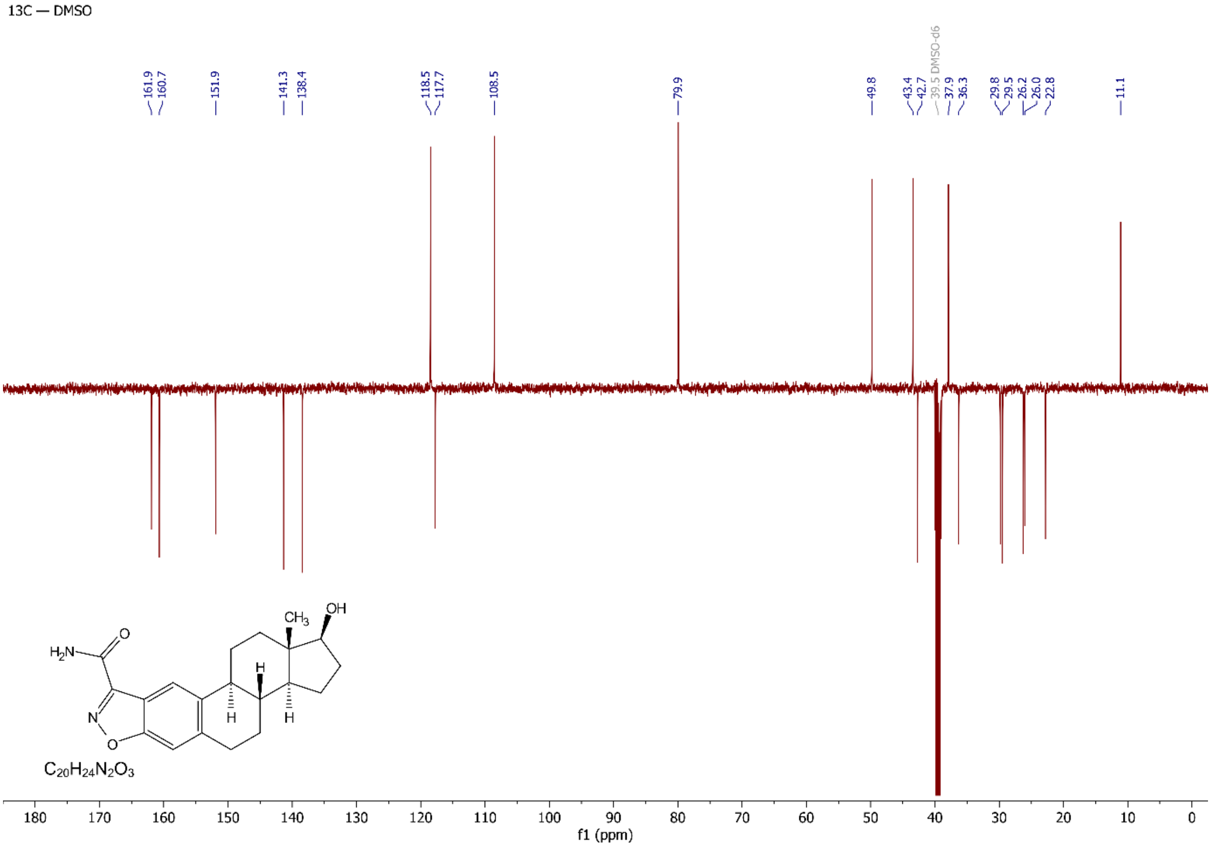

$^{1}H$ NMR spectrum of compound **8** (DMSO-*d*$_6$, 500 MHz)

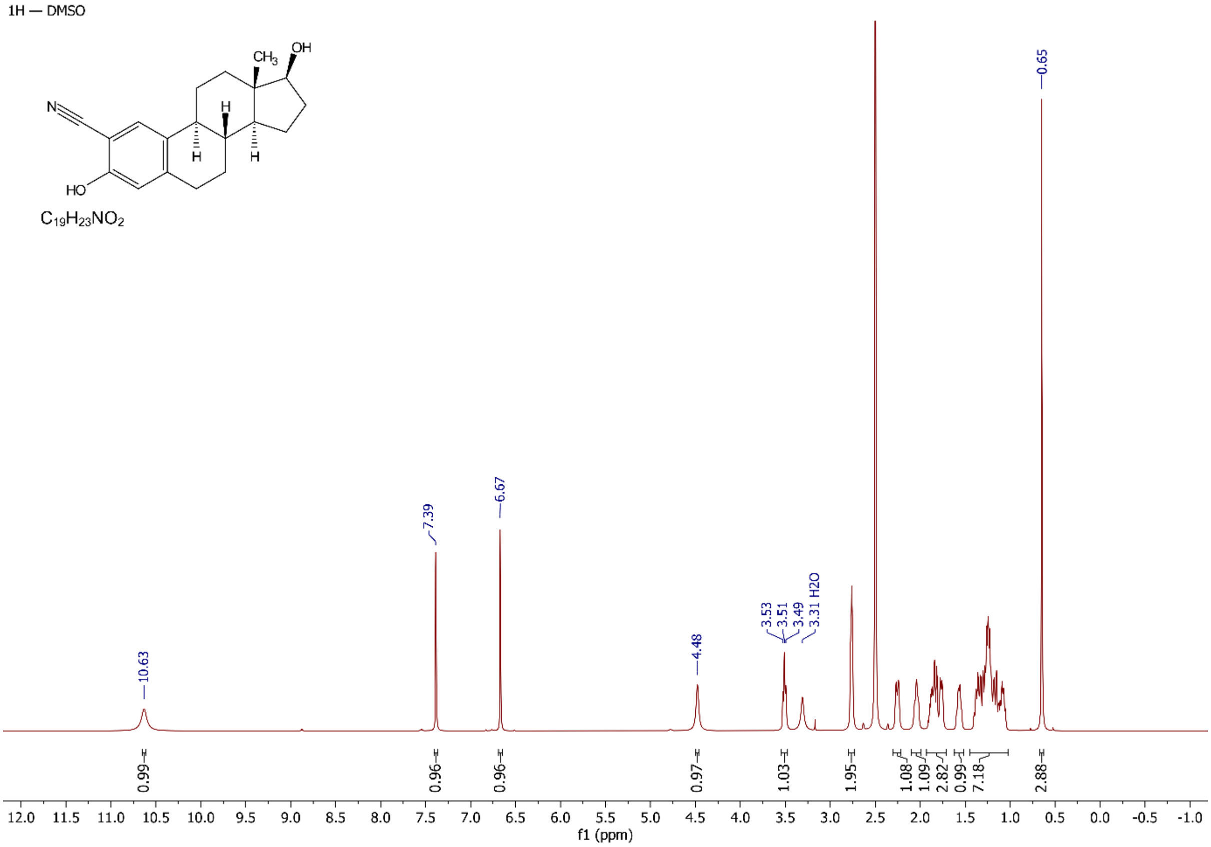


$^{13}C$ NMR spectrum of compound **8** (DMSO-*d*$_6$, 125 MHz)

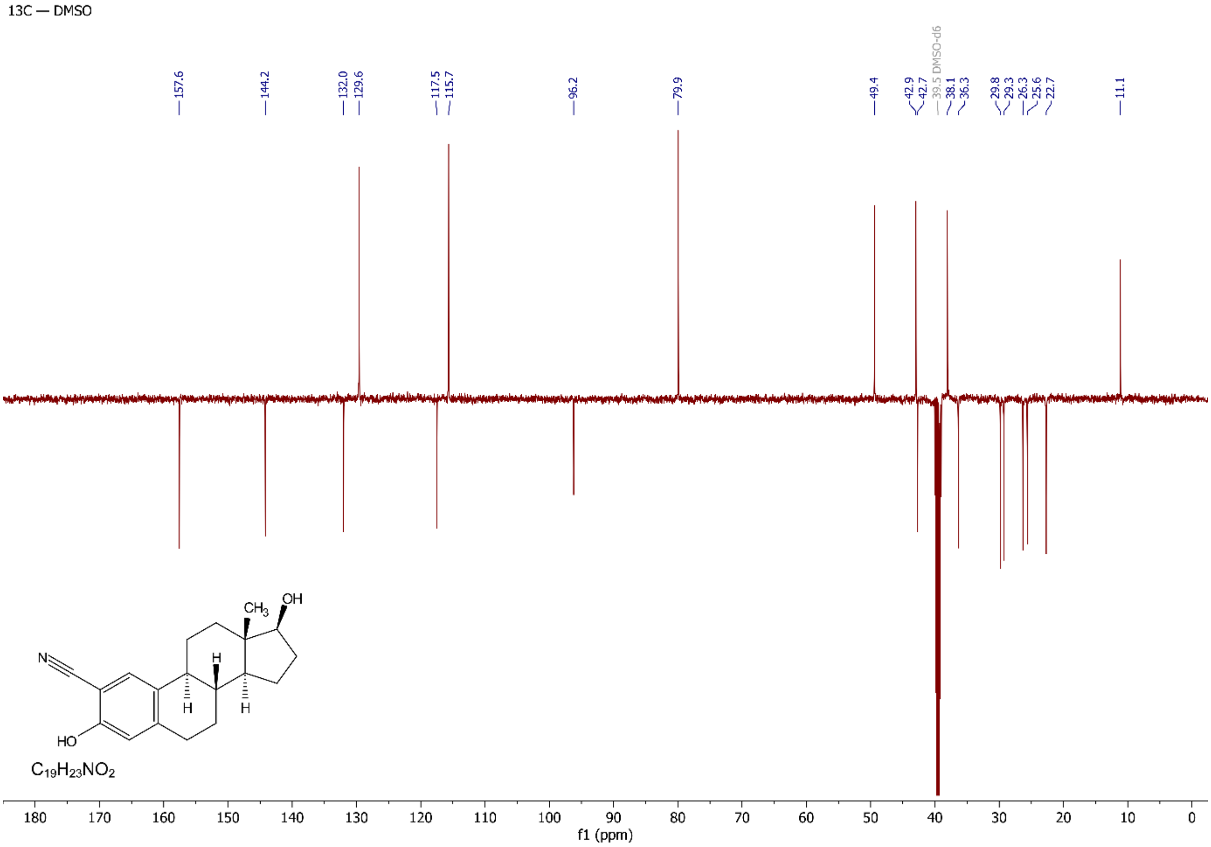

$^{1}H$ NMR spectrum of compound **9** (DMSO-$d_6$, 500 MHz)

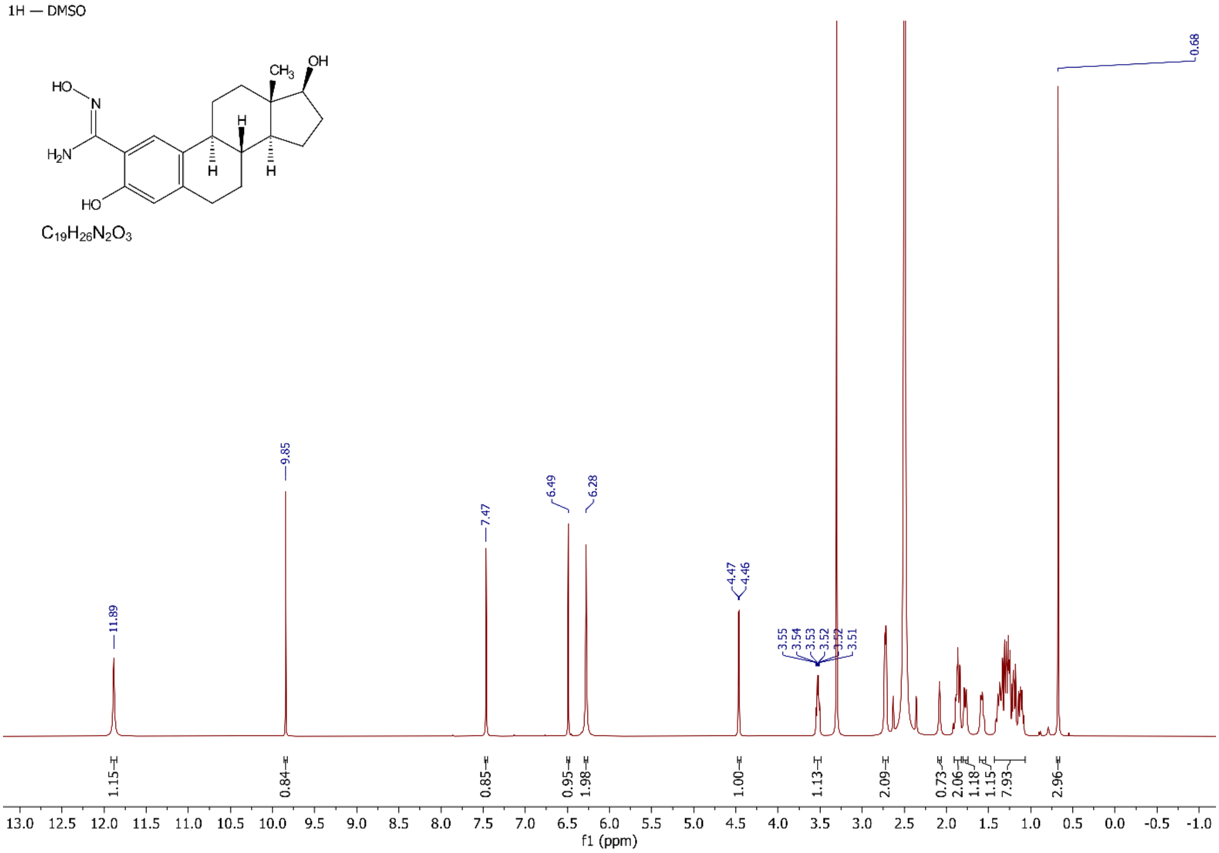


$^{13}C$ NMR spectrum of compound **9** (DMSO-$d_6$, 125 MHz)

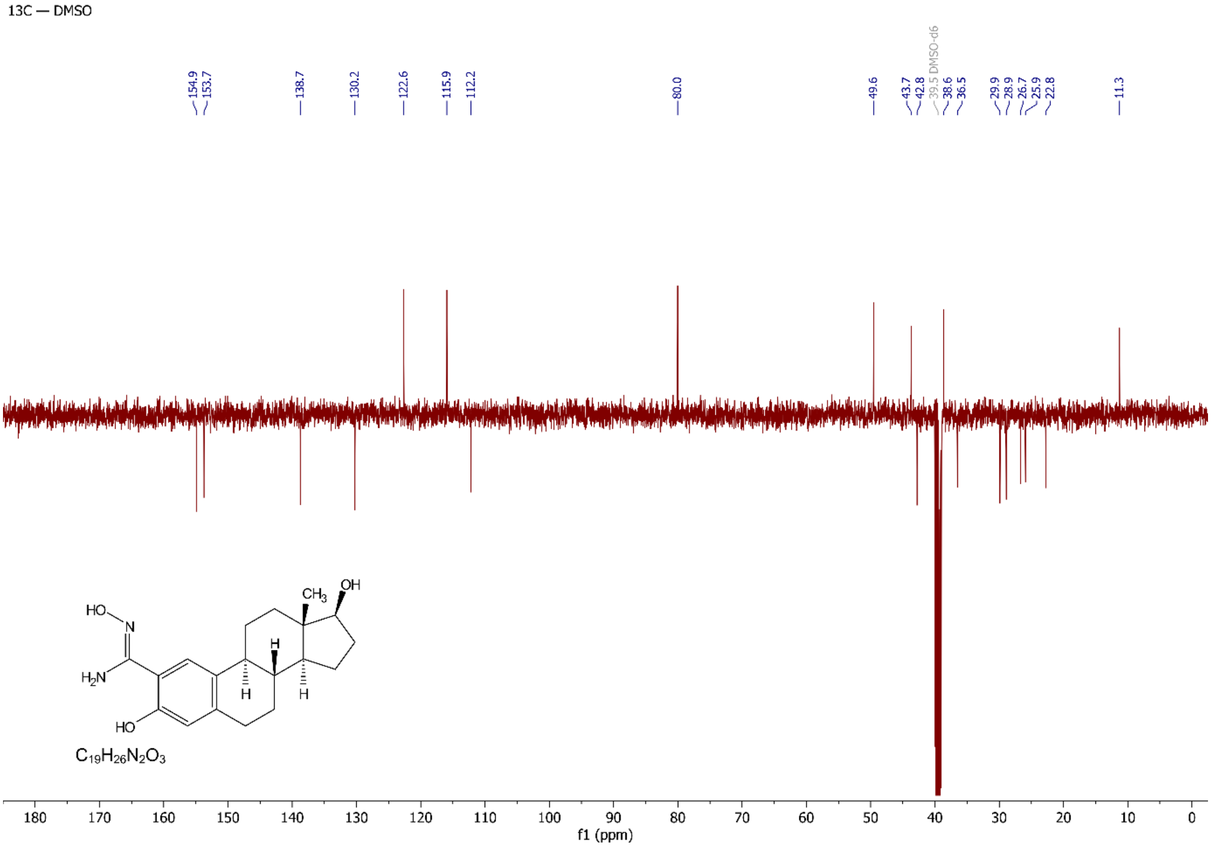

$^{1}$H NMR spectrum of compound **10** (DMSO-$d_6$, 500 MHz)

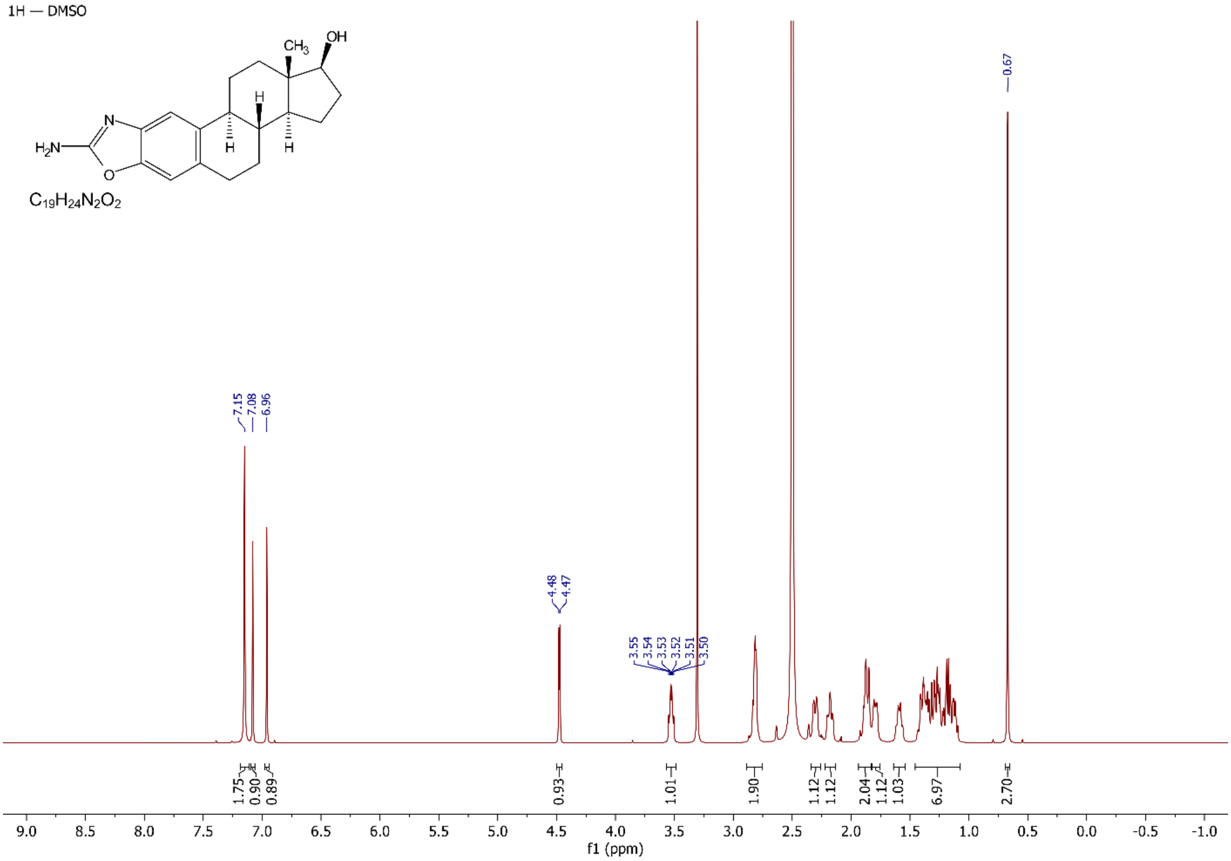


$^{13}$C NMR spectrum of compound **10** (DMSO-$d_6$, 125 MHz)

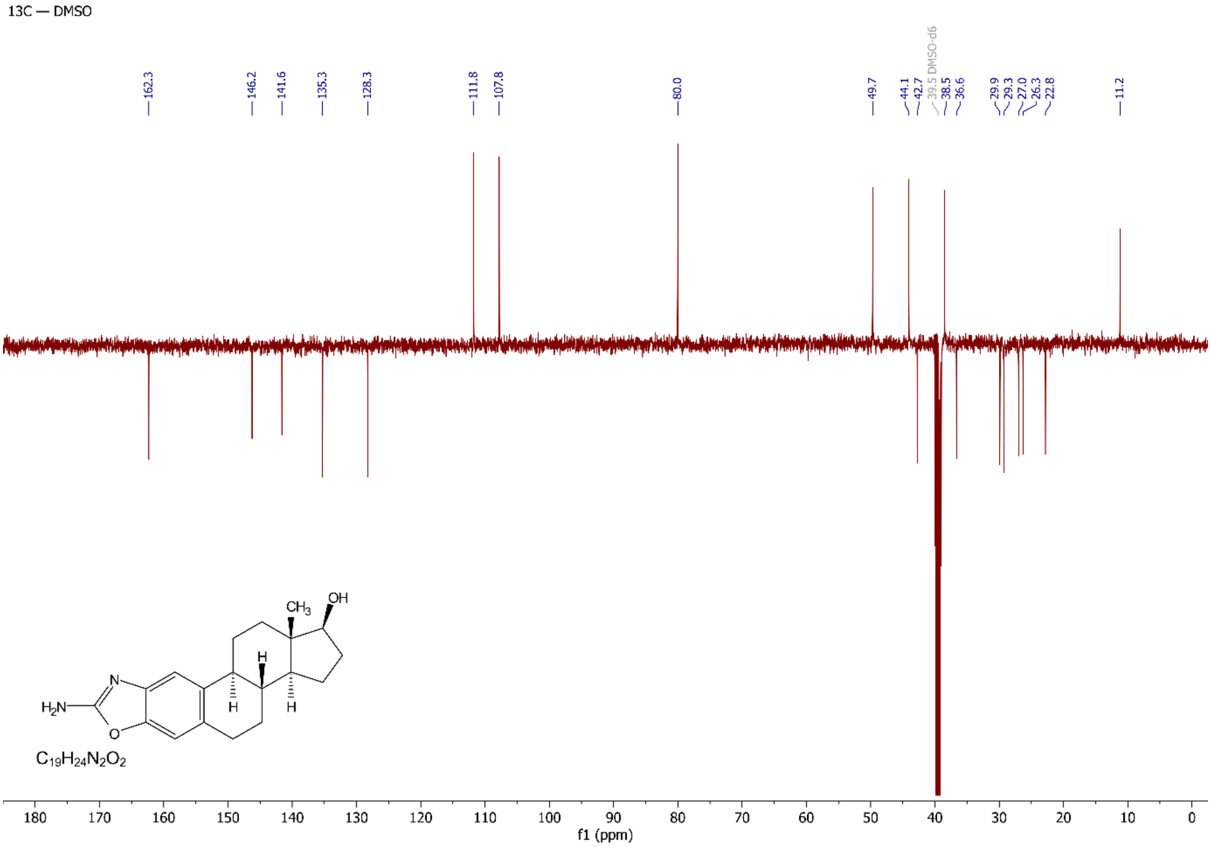

*Predicted pharmacokinetic parameters of the synthesized compounds**

| R | HBD[1] | HBA[2] | Exact mass (Da) | LogP | TPSA[3] (Å$^2$) | Rotatable Bonds |
|---|---|---|---|---|---|---|
| H | 1 | 2 | 297.17 | 3.432 | 46.26 | 0 |
| Me | 1 | 2 | 311.19 | 3.564 | 46.26 | 0 |
| Et | 1 | 2 | 325.20 | 4.264 | 46.26 | 1 |
| $^{i}$Pr | 1 | 2 | 339.22 | 4.807 | 46.26 | 1 |
| Ph | 1 | 2 | 373.20 | 5.466 | 46.26 | 1 |
| $CH_2F$ | 1 | 2 | 329.18 | 3.638 | 46.26 | 1 |
| $CHF_2$ | 1 | 2 | 347.17 | 3.979 | 46.26 | 1 |
| $CF_3$ | 1 | 2 | 365.16 | 4.696 | 46.26 | 1 |
| COOH | 2 | 4 | 341.16 | 3.476 | 83.56 | 1 |
| COOMe | 1 | 3 | 355.18 | 3.622 | 72.56 | 2 |
| $CONH_2$ | 2 | 3 | 340.18 | 2.669 | 89.35 | 1 |
| $CH_2OH$ | 2 | 3 | 327.18 | 2.747 | 66.49 | 1 |
| OH | 2 | 3 | 313.17 | 2.959 | 58.56 | 0 |
| OMe | 1 | 3 | 327.18 | 3.869 | 55.49 | 1 |
| **Ideal Values[4]** | **<5** | **<10** | **<500** | **<5** | **<140** | **<10** |

* Predicted with Chemaxon's Chemicalize software
[1] Hydrogen bond donor
[2] Hydrogen bond acceptor
[3] Topological polar surface area
[4] Based on the Lipinski's and Veber's criteria

*Mean values of primary growth inhibitory screen used for heat map construction*

| | MRC-5 | DU-145 | PC3 | HeLa | MCF-7 |
|---|---|---|---|---|---|
| 2a | 100±9.6 | 100±9.3 | 94±7.7 | 37.2±6.4 | 100±2.5 |
| 2b | 100±6.3 | 99.6±8 | 85.1±8.4 | 96.4±4 | 100±6.9 |
| 2c | 100±10.1 | 88.5±7.4 | 71.1±7.3 | 82.1±7.1 | 82.3±7 |
| 2d | 100±10.1 | 76.9±11.5 | 73.9±7.9 | 100±3.7 | 88±7.9 |
| 2e | 100±9.6 | 100±4.7 | 82.5±6.7 | 87.8±6.8 | 100±6.4 |
| 2f | 100±10.4 | 98.6±11.6 | 79.4±7.8 | 100±9.5 | 100±4.8 |
| 4a | 100±8.6 | 100±7.7 | 83.6±4.2 | 1.4±0.3 | 100±7.2 |
| 4b | 90.1±9.2 | 14.7±4 | 30.2±5 | 10.7±5.1 | 6.5±3.9 |
| 4c | 99.2±10 | 11.5±3.1 | 32.3±3.8 | 3.8±2.5 | 23.6±3.2 |
| 4d | 98.3±11 | 41.1±9.5 | 51.6±9.6 | 17.2±9.5 | 39.4±2.6 |
| 4e | 100±9.3 | 100±5.9 | 64.2±2.9 | 49.8±8.2 | 100±6.6 |
| 4f | 100±8.5 | 100±7.6 | 85.7±8.2 | 100±3.2 | 100±9.2 |
| 4g | 100±5.5 | 100±6.1 | 94.6±9.2 | 31±9.3 | 100±3.7 |
| 4h | 100±10 | 40.8±7.4 | 85.4±5.3 | 0.4±0.4 | 100±4.6 |
| 4i | 100±10 | 100±6.6 | 100±1.9 | 51.7±6.1 | 100±5.4 |
| 4j | 100±8.8 | 75.9±3.4 | 87.3±7 | 100±4 | 98.2±7.6 |
| 4k | 95.6±5.5 | 100±6.7 | 89.7±8 | 48.5±6.6 | 90.5±5.3 |
| 4l | 91.5±6.7 | 73.9±3.2 | 76.1±2.4 | 100±7.1 | 97.2±4.5 |
| 4m | 96.4±5.8 | 73.8±3.9 | 82.8±4.2 | 76.5±7.2 | 89.4±7.4 |
| 4n | 100±5.6 | 83.6±5.6 | 88.4±4.4 | 93.5±7.1 | 100±9.4 |
| 4o | 96.1±10 | 100±5 | 78.2±7.5 | 60.3±1.7 | 100±8.7 |
| 4p | 99.2±8.6 | 78.3±8.9 | 77.4±8.5 | 91.3±5 | 100±6.9 |
| 4q | 100±6.6 | 72.3±5.8 | 85.8±8.3 | 1.5±0.4 | 97.8±7.1 |
| 4r | 100±10.2 | 74.4±8 | 74.9±5.3 | 2.6±1 | 100±3.9 |
| 4s | 100±3.2 | 100±3.6 | 88.3±3.6 | 71.1±4.6 | 100±8.8 |
| 6c | 100±4.7 | 90.1±6 | 94.3±9.4 | 92.7±2.2 | 100±10 |
| 6d | 92.8±7.6 | 67.1±4.6 | 62.6±3 | 22±5.8 | 38.4±4.5 |
| 7 | 100±9.1 | 100±5.1 | 87.9±9.2 | 22.9±10.7 | 100±7.3 |
| 8 | 100±6.2 | 100±5.2 | 94.2±6.4 | 1.6±0.3 | 97.5±5.9 |
| 9 | 99.7±6.8 | 57.4±5.9 | 53.9±5.9 | 16.7±6.7 | 57.1±2.8 |
| Control | 100±9.1 | 100±5.2 | 100±2.6 | 100±6.3 | 99.5±3 |

Mean ± SD values of primary growth inhibitory screen (given as cell viability) used for heat map construction. The compounds were tested in 2.5 μM concentration for 72 h. Control represents viability of cells receiving no treatment.

*Dose-response curves*

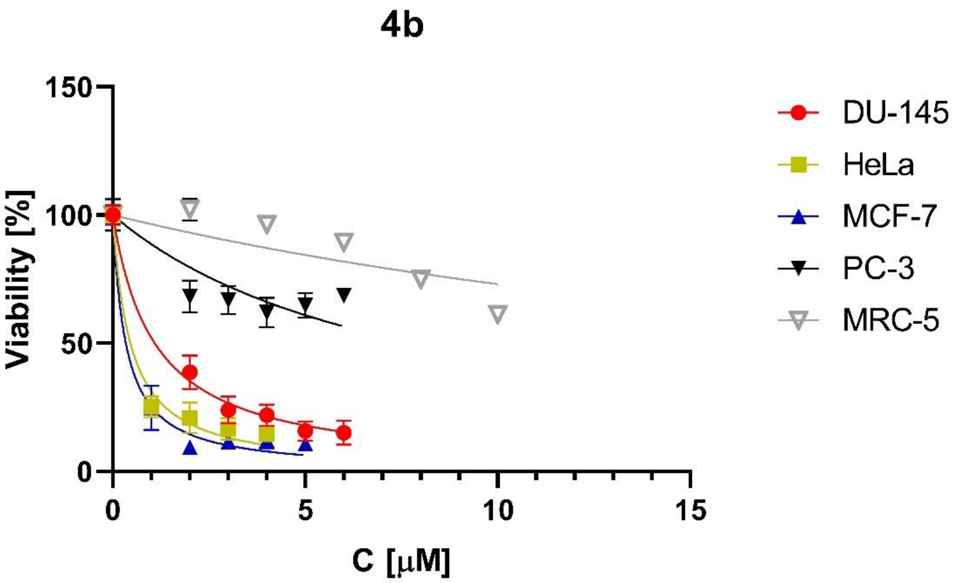


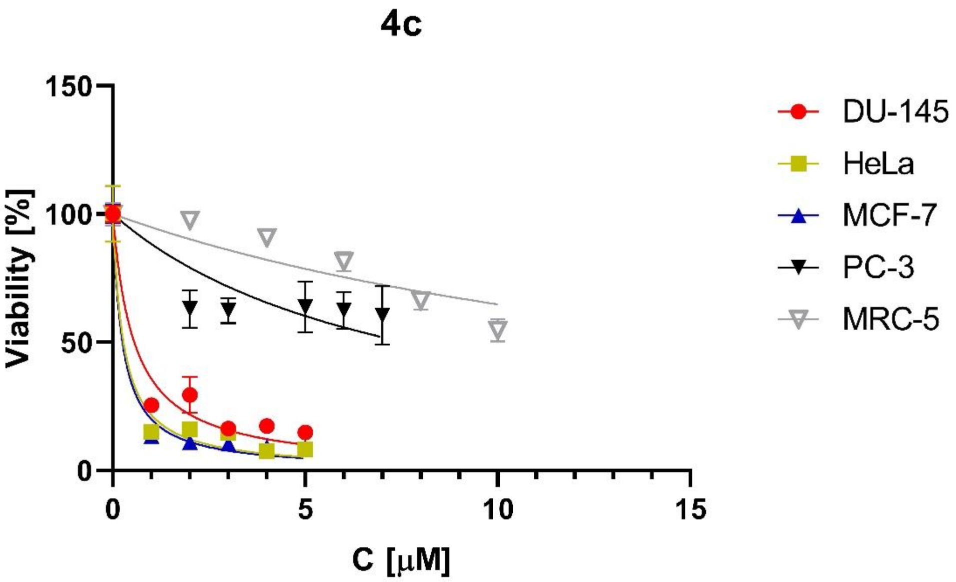

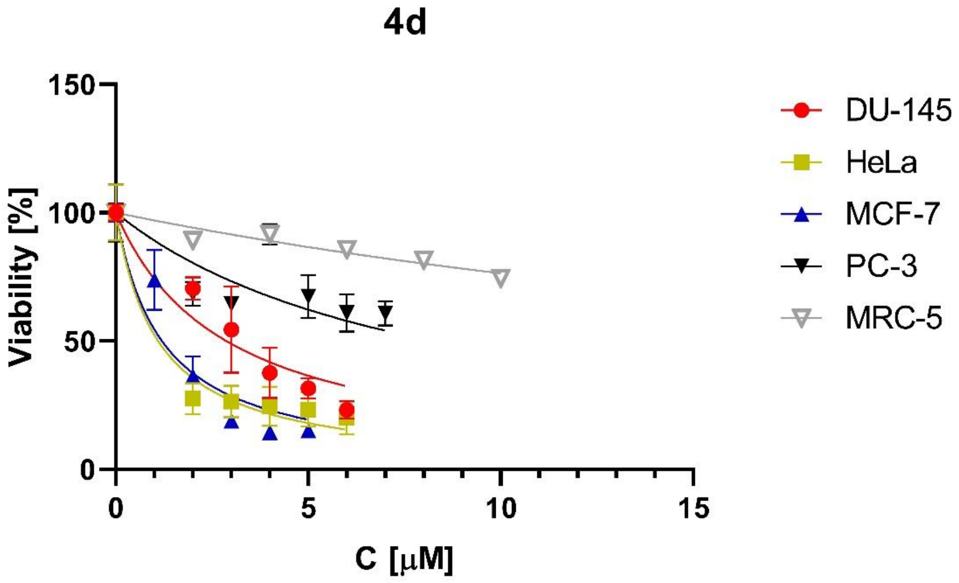


Representative cell viability curves to determine growth inhibition and $IC_{50}$ values following treatments with the selected compounds **4b**, **4c**, **4d** on different cell lines. On X-axis the applied concentrations in μM are represented. Based on the viability data, $IC_{50}$ values were obtained, and represented in Table 1.

*List, sequence and appropriate concentrations of primers used for qRT-PCR.*

| Gene | Forward-primer<br>Reverse-primer | Final concentration [nM] |
|---|---|---|
| *GAPDH* | 5'-TGCACCACCAACTGCTTAGC-3'<br>5'-GGCATGGACTGTGGTCATGAG-3' | 200 |
| *BAX* | 5' TGCTTCAGGGTTTCATCCAG 3'<br>5' GGCGGCAATCATCCTCTG 3' | 200 |
| *Casp-3* | 5' ACATGGCGTGTCATAAAATACC 3'<br>5' CACAAAGCGACTGGATGAAC 3' | 200 |
| *p21* | 5'-CAGCAGAGGAAGACCATGTG-3'<br>5'-GGCGTTTGGAGTGGTAGAAA-3' | 100 |
| *p53* | 5'-CCCTTCCCAGAAAACCTACC-3'<br>5'-CTCCGTCATGTGCTGTGACT-3' | 200 |
